# Climate‐Induced Range Shift and Risk Assessment of Emerging Weeds in Queensland, Australia

**DOI:** 10.1002/ece3.71043

**Published:** 2025-04-02

**Authors:** Olusegun O. Osunkoya, Mohsen Ahmadi, Christine Perrett, Moya Calvert, Boyang Shi, Steve Csurhes, Farzin Shabani

**Affiliations:** ^1^ Invasive Plant & Animal Science Unit, Biosecurity Queensland, Department of Agriculure & Fisheries EcoSciences Precinct Brisbane Queensland Australia; ^2^ Department of Natural Resources Isfahan University of Technology Isfahan Iran; ^3^ College of Arts and Sciences Qatar University Doha Qatar

**Keywords:** climate change, invasive alien species, pest prioritisation, range shift, risk‐assessment, species distribution modelling

## Abstract

Anticipation and identification of new invasive alien species likely to establish, spread and be impactful in a landscape, especially in response to climate change, are consistently a top priority of natural resource managers. Using available global bioclimatic variables limiting plant distributions, we employed maximum entropy (MaxEnt) as a correlative species distribution model to predict the current and future (2041–2060 and 2061–2080) distribution for 54 emerging weed species of different growth forms for the State of Queensland, Australia. Overall, the model predictive performance was excellent, with area under the curve (AUC) and the true skill statistic (TSS) averaging 0.90 and 0.67, respectively. Based on distribution records, the emerging weed species sorted out along environmental (climatic) space—with trees and succulents, each at the two ends of the continuum, while grasses, herbs and shrubs were distributed between the two extremes. Temperature seasonality and minimum temperature of the coldest month were the main driver variables that accounted for differences in climatic preference among the focal species and/or plant growth forms. Range shifts were predicted for many species in response to climate change; overall, habitat range increase will occur more often than range contraction and especially more so in trees compared to all other plant growth forms. Range stability was least in succulent weeds. In general, under climate change, the majority of the invasion hotspot area was projected to remain geographically stable (76.95%). Far northern Queensland (especially the Gulf of Carpentaria and Cape York Peninsula areas) and the coastal communities along the eastern seaboards of the State are the hotspots for emerging invasive alien species to establish and expand/contract in response to climate change. Based on observed and potential ranges, as well as species response to climate change, we derived an index of risk and hence statewide prioritisation watch list for management and policy of the emerging weeds of Queensland.

## Graphical abstract

The paper examined the present distribution of 54 emerging weeds of the State of Queensland, Australia, and through the use of a correlative/environmental model of MaxEnt software, predicted their potential habitat suitability and response (range shift) to climate change scenarios. These distribution traits are then used to generate risk assessments and watch list rankings for the focal weeds that can feed into policy and management in terms of pre‐ and post‐border surveillance, eradication and/or control.

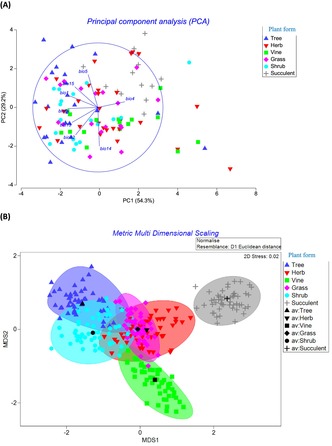

## 1 Introduction

Early detection and rapid response to new invasive organisms can be an effective form of management (Westbrooks 2004; Csurhes 2021; Buddenhagen et al. 2023). Deciding which emerging invasive species to pre‐emptively search for is a critical component of effective early detection and rapid response (Westbrooks 2004; Osunkoya et al. 2022). Consequently, the concept of risk assessment and prioritisation of candidate taxa likely to become invasive (also known as horizon scanning) is attractive and is gaining a wide currency as it is cost‐effective and vital for stopping harmful invasions (Cuhls 2020; Dawson et al. 2022; Buddenhagen et al. 2023). Horizon or environmental scanning warns us about impending change. The horizon scanner is to the future what the lookout is to the sea. Thus, horizon scanning breaks the habit of ignoring the early signs of change. It forces people to look at events around them and report those signs that could have detrimental consequences on the enterprise, not just those that are sure to have impacts (Cuhls 2020; Osunkoya et al. 2022).

The establishment of these new invasive alien species (henceforth IAS) may become even more impactful as they may benefit from changing climatic regimes and anthropogenic opportunities following bushfires, extreme flooding and other disturbances (Westbrooks 2004; O'Donnell et al. 2012; Osunkoya et al. 2014). Contemporary climate change is a primary driver determining future invasive species distribution patterns—be it established or incoming/emerging ones (Jia et al. 2016; Hulme 2017; Bellard et al. 2018). Therefore, understanding how climate change influences the ranges of IAS is important (Bellard et al. 2018; Bradley et al. 2023). The projected increases in atmospheric CO_2_ concentration and changes in temperature and precipitation patterns may alter ecosystem functions, species interactions, demography including age structure and plant distribution (Stocker et al. 2013; Bradley et al. 2023). Temperature and precipitation are considered determinants of many successful range expansions along latitudinal and altitudinal gradients and in multiple ecosystems and several habitat types (Bellard et al. 2016). Increased precipitation has also been linked to woody weed encroachment (Graz 2008; Archer et al. 2017). Many studies of global climate change impact on the geographic distribution of IAS reported varied consequences (Merow et al. 2017; Bradley et al. 2023). For example, several studies predicted that climate change will increase IAS ranges (Dukes and Mooney 1999; Thuiller et al. 2008; Shrestha et al. 2018; Thapa et al. 2018), while others have found opposite trends (Allen and Bradley 2016; Bezeng et al. 2017; Pillet et al. 2022). Thus, projecting future direction and magnitude of invasion impact and/or areas of low or high invasion risk is challenging and largely context dependent—being largely influenced by the IAS traits, ecosystem type invaded and the nature of anthropogenic opportunities (Kriticos et al. 2006; Broennimann et al. 2006; MacLean and Beissinger 2017). Nonetheless, these projections remain essential for making cost‐effective management decisions as they play crucial roles in evaluating the invasion risks posed by introduced species and identifying threats to protected ecosystems. However, introduced species, particularly invasive ones, may not necessarily occupy the same climatic niche in their native and introduced ranges due to changes in their realised or fundamental climatic niche (Gallagher, Beaumont, et al. 2010; Allen and Bradley 2016; Eckert et al. 2020). This phenomenon, referred to as a ‘niche/range shift’, complicates efforts to predict future distributions of IAS.

A large body of literature attempts to explain variation in IAS range size and shifts using ecological and life‐history traits of the species, with predictions that shifts should be greater in species with greater dispersal ability and that are ecological generalists (Estrada et al. 2016; MacLean and Beissinger 2017). Thus, although individual IAS may respond idiosyncratically to climate change, species that share the same ecological properties, such as a similar peculiar photosynthetic pathway, growth/life form (e.g., grasses) or evolutionary history/lineage (e.g., family), might respond in a similar fashion (Thuiller et al. 2005; Thuiller, Lavorel, et al. 2006; Gallagher, Beaumont, et al. 2010). Although this approach is central to the framework of dynamic global vegetation models (Daly et al. 2000; Woodward and Lomas 2004), it has rarely been tested in climate change impact studies involving species distribution models (SDMs) (but see Thuiller, Midgley, et al. 2006). To date, SDM studies concentrate on quantifying species' range changes, but minimal efforts are often made to explore the drivers of the predicted ecological patterns (but see Thuiller et al. 2005; Broennimann et al. 2006).

Although other than climate, many factors contribute to the spread of IAS (particularly human‐assisted dispersal, land disturbance and modification of natural fire regimes), incorporating modelled projections of climate suitability into pest risk assessment and prioritisation systems would provide a useful indicator of future threats and help identify which regions may become hotspots for invasion under future climates (O'Donnell et al. 2012; Duursman et al. 2013; Gallagher, Beaumont, et al. 2010; Shabani et al. 2020; Evans et al. 2024; Szyniszewska et al. 2024). Despite the acknowledgement of climate change and its impact, a shortfall of current determinations of weed threats and prioritisation, such as the Australian Weed Risk Assessment system or the recently completed risk inventory of established weeds of Queensland, Australia (Osunkoya, Froese, Nicol, Perrett, et al. 2019), is that they fail to include potential responses of IAS to climate change, thus reducing the potential efficacy of management prioritisation (Downey et al. 2010; Roger et al. 2015; Jarnevich et al. 2023; Szyniszewska et al. 2024).

Eastern Australia (especially the States of New South Wales and Queensland) is among the five global regions considered most vulnerable to the establishment of new IAS originating mainly from Asia and America via trade, tourism and human traffic (Bellard et al. 2016). In Australia, future projections in response to climate change have been reported for established weeds (e.g., Kriticos et al. 2006; Duursman et al. 2013; Gallagher et al. 2013; O'Donnell et al. 2012; see also http://weedfutures.net/species_list.php). However, at Australia's regional/state levels, there are very few published reports on risk assessments and/or potential impacts of climate change on emerging and incoming (horizon) weed floras (e.g., Waterhouse 2003; Osunkoya, Froese, Nicol, Perrett, et al. 2019; Osunkoya et al. 2020). Though in the past, SDMs of many IAS of Queensland have been produced, such prediction exercises are for established IAS and are often based on modelling software like CLIMATCH, known for its coarse scale and low accuracy as it uses means rather than other climate measurements such as minimums or maximums (Froese 2012; Erickson et al. 2022). CLIMATCH is also unable to model climate change scenarios directly (Erickson et al. 2022; but see Kriticos et al. 2003, 2006). Hence, there is a need to perform a similar exercise on incoming (horizon) weeds and, where applicable, to update predictions for established IAS using improved modelling approaches like ecological niche models (ENMs) (Elith et al. 2010; Adhikari et al. 2022). In this work, we have assembled a cohort of incoming weeds (54 species) in the State of Queensland (henceforth QLD) that are either in low abundance and population foci or are yet to occur in the State but are perceived by stakeholders as detrimental to the region if allowed to establish and flourish. Our aims using the maximum entropy (MaxEnt) model as a well‐performing ENM software and focusing on the northern part of eastern Australia (specifically QLD) are to:

1. Document occurrence records and explore the potential habitat ranges of these identified incoming weeds in Queensland, Australia.
2. Model shift in habitat ranges of these weeds in response to predicted climate change, and subsequently explore the influence of components of environmental variables of temperature and rainfall, as well as species‐specific traits of plant growth form and taxonomic affiliation (lineage) as possible drivers of predicted changes; in line with many previous findings and hypotheses, we predicted an increase in climatically suitable and hence habitat ranges of many of these weed species, but the responses (magnitude and direction) might be context‐dependent (e.g., vary in response to plant growth form and landscape/regional level).
3. Rank the weeds for statewide prioritisation and management/policy actions based on observed and predicted habitat ranges and response to climate change.

## 2 Methods

### 2.1 Study Region

The study area (QLD) lies in the north‐eastern part of Australia (Appendix). Spanning an area of ~1.853 million km^2^, QLD is the second‐largest state by land and the third‐most by population (~5.5 million) and experiences significant climatic and environmental gradients. Mean precipitation ranges from 400 to 780 mm per year; the average minimum annual temperature varies from −10.6°C to 5.4°C, and the average maximum annual temperature varies from 36.0°C to 49.7°C (Australia Bureau of Meteorology, http://www.bom.gov.au/, accessed 8 March 2024); the summer average temperature is 29°C. Taking cognizance of climate change, the average temperature is predicted to increase to over 30°C and 32°C by 2030 and 2070, respectively. A substantial increase in the temperature on the hottest days, as well as a significant spike in the frequency of hot days and the duration of warm spells, is also likely. An increased magnitude of extreme rainfall events is also projected, with high confidence. The mean sea level will increase, and the height of extreme sea‐level events will increase the risk of coastal hazards such as storm and tide inundation (see https://longpaddock.qld.gov.au/qld‐future‐climate/understand‐data/).

Established invasive flora of QLD, just like its native flora, varies across regions but is more similar across local government areas (LGAs) within a given region (Osunkoya, Froese, Nicol, Perrett, et al. 2019; Osunkoya et al. 2021). With state government supervision and oversight, invasive plants and animals are managed at regional levels by local government authorities and natural resource management groups (Osunkoya et al. 2020, 2021). To ensure that new pest species are detected early, reported, and assessed to determine whether management actions (eradication, control or simply placed on a watch list) should be undertaken, Biosecurity Queensland (an agency of QLD Department of Agriculture and Fisheries) maintains a register of potential/incoming weeds (~250 species) into the State (Csurhes 2021; https://www.daf.qld.gov.au/business‐priorities/biosecurity/invasive‐plants‐animals/plants‐weeds). It is from this ‘watch list’ that the 54 focal species in this work were selected. The majority of these watch‐list species are currently of low abundance with few population foci or are yet to arrive in the State but may already be present in neighbouring States of New South Wales (NSW), Northern Territory (NT) and South Australia (SA) (Table 1). In this study, species inclusion for climatic range and risk assessment study is based on (i) statewide consultations with impacted stakeholders (QLD biosecurity officers, landowners and natural resource management groups) who recognise the species' potential to spread and cause environmental, social and economic impacts and (ii) availability of adequate global occurrence records in the species' invaded and native ranges for the predictive modelling exercise. The investigated species cover most plant growth forms (grass: *n =* 9; herb: *n* = 13; shrub: *n* = 9; succulent: *n* = 6; tree: *n* = 10; and vine: *n* = 7).

**TABLE 1:** Investigated emerging (horizon) weed species of QLD, along with their growth form, family affiliation, native origin, time and occurrence records in QLD, and potential range as predicted by MaxEnt software. Superscript symbols on species names indicate the presence of population foci in adjoining States of New South Wales (^a^), Northern Territory (^b^) and South Australia (^c^).

Sp. no.	Species name	Family	Common name	Growth form	Native range	Year of first record in QLD	Occurrence record (actual no. of ~4.5 km × 4.5 km grids of QLD land area infested)		Potential QLD land area infested (%) based on MaxEnt prediction
							Count	As a % of land mass	
1	*Acaciella glauca*	Fabaceae	Redwood	Tree	Southern America	1957	14	0.015	5.64
2	*Acanthospermum australe* ^a^	Asteraceae	Spiny‐bur	Herb	Southern America	1956	4	0.004	6.67
3	*Amphilophium crucigerum* ^a^	Bignoniaceae	Monkeys comb	Vine	Southern America	1955	12	0.013	10.55
4	*Artemisia verlotiorum*	Asteraceae	Chinese mugwort	Herb	Northern America		0	0.000	4.80
5	*Arundo donax* ^a,b,c^	Poaceae	Giant reed	Grass	Asia	1912	47	0.049	0.05
6	*Asparagus retrofractus*, syn *A. africanus*	Liliaceae—Asparagaceae	Ming Asparagus fern	Vine	Africa	2001	2	0.002	0.38
7	*Barleria repens*	Acanthaceae	Small Bush Violet	Shrub	Asia and Africa	2005	54	0.057	3.42
8	*Bignonia magnifica*	Bignoniaceae	Glow vine	Vine	Southern America	2007	17	0.018	4.00
9	*Cabomba caroliniana* ^a,b^	Cabombaceae	Carolina fanwort	Herb	Southern America	1960	58	0.061	0.46
10	*Cenchrus purpureus* ^a,b^	Poaceae	Elephant grass	Grass	Africa	1927	25	0.026	1.85
11	*Cereus hildmannianus* syn *C. uraguayanus* ^a^	Cactaceae	Hedge cactus	Succulent	Southern America	2012	28	0.029	28.55
12	*Ceropegia gigantea* ^a^	Apocynaceae	Lantern flower	Succulent	Africa	1950	11	0.012	11.38
13	*Chromolaena odorata* ^a,b^	Asteraceae	Crucita	Shrub	Southern America	1993	229	0.240	6.26
14	*Coffea arabica* ^a^	Rubiaceae	Coffee	Shrub	Africa	1934	37	0.039	1.65
15	*Coix lacryma‐jobi* ^a^	Poaceae	Job's Tears	Grass	Asia	1930	5	0.005	1.97
16	*Cylindropuntia fulgida* ^a,b,c^	Cactaceae	Boxing glove cactus	Succulent	Southern America	1955	59	0.062	3.14
17	*Dalbergia sissoo* ^b^	Leguminosae—Papilionaceae	Himalaya raintree	Tree	Asia	1912	22	0.023	10.83
18	*Diplachne uninervia* ^a,c^	Poaceae	Mexican sprangletop	Grass	Southern America	1988	15	0.016	0.95
19	*Dyschoriste nagchana*	Acanthaceae	Nagchana Bush Violet	Herb	Africa & Asia	2000	27	0.028	5.56
20	*Echinochloa polystachya* ^a,b^	Poaceae	Aleman Grass	Grass	Southern America	1985	36	0.038	14.78
21	*Elephantopus mollis*	Asteraceae	Elephant's foot/Tobacco weed	Herb	Southern America	1989	56	0.059	2.87
22	*Florestina tripteris* ^a^	Asteraceae	Sticky florestina	Herb	Southern America	1989	16	0.017	40.50
23	*Gliricidia sepium* ^b^	Leguminosae—Papilionaceae	Gliricidia	Tree	Southern America	1960	4	0.004	5.55
24	*Gmelina arborea* ^b^	Lamiaceae	Gamhar	Tree	Asia	1936	6	0.006	12.08
25	*Heteranthera reniformis* ^a^	Pontederiaceae	Kidneyleaf Mud Plantain	Herb	Americas	2007	34	0.036	4.99
26	*Hyparrhenia rufa* ^a,b^	Poaceae	Thatch grass	Grass	Africa	1966	308	0.323	11.68
27	*Indigofera schimperi*	Leguminosae—Papilionaceae	Schimper's indigo	Herb	Africa	1972	7	0.007	7.59
28	*Ipomoea alba* ^a^	Convolvulaceae	Moonflower	Vine	Southern America	1948	20	0.021	7.33
29	*Jatropha curcas* ^b^	Euphorbiaceae	Nutmeg plant	Shrub	Southern America	1924	14	0.015	13.01
30	*Khaya senegalensis* ^b^	Meliaceae	African mahogany	Tree	Africa	1970	10	0.010	2.78
31	*Leonotis nepetifolia* ^a,b^	Lamiaceae	Christmas candlestick	Herb	Asia and Africa	1925	47	0.049	1.94
32	*Manihot glaziovii*	Euphorbiaceae	Ceara rubber tree	Shrub	Southern America	1942	4	0.004	1.72
33	*Mesosphaerum pectinatum*	Lamiaceae	Comb hyptis	Herb	Southern America	1991	22	0.023	3.68
34	*Miconia racemosa*	Melastomataceae	Camasey felpa	Shrub	Southern America	2002	1	0.001	0.20
35	*Mimosa pigra* ^b^	Leguminosae—Mimosaceae	Giant sensitive tree	Shrub	Southern America	2001	5	0.005	11.38
36	*Murraya koenigii* ^a,b^	Rutaceae	Curry Leaf	Tree	Asia	1779	26	0.027	1.82
37	*Neptunia plena* ^a,b^	Leguminosae—Mimosaceae	Bashful Bush	Herb	Southern America	1964	9	0.009	10.86
38	*Opuntia dejecta*	Cactaceae	Prickly pear	Succulent	Central & Southern America	1983	1	0.001	12.78
39	*Opuntia elata* ^a^	Cactaceae	Riverina pear	Succulent	Southern America	2015	15	0.016	0.67
40	*Opuntia sulphurea*	Cactaceae	Sulphur cactus	Succulent	Southern America	1983	5	0.005	0.05
41	*Paspalum mandiocanum* ^a^	Poaceae	Broadleaf Paspalum	Grass	Southern America	2010	44	0.046	1.79
42	*Pithecellobium dulce* ^b^	Fabaceae	Madras thorn	Tree	Southern America	1982	19	0.020	15.20
43	*Praxelis clematidea*	Asteraceae	Praxelis	Herb	Southern America	1993	327	0.343	7.94
44	*Rhodomyrtus tomentosa* ^a^	Myrtaceae	Rose myrtle	Shrub	Asia	1970	9	0.009	0.86
45	*Rotala rotundifolia* ^a^	Lythraceae	Dwarf Rotala	Herb	Asia	1974	15	0.016	1.89
46	*Schizachyrium microstachyum*	Poaceae	Little bluestem	Grass	Southern America	2001	22	0.023	4.48
47	*Setaria parviflora* ^b^	Poaceae	Marsh bristlegrass	Grass	Americas	1930	4	0.004	5.27
48	*Sieruela rutidosperma* ^b^	Cleomaceae	Fringed Spider flower	Herb	Africa	2020	2	0.002	0.93
49	*Spathodea campanulata* ^a,b^	Bignoniaceae	African tulip tree	Tree	Africa	1933	89	0.093	4.37
50	*Stigmaphyllon ciliatum* ^a,b^	Malpighiaceae	Orchid vine	Vine	Southern America	1918	6	0.006	0.67
51	*Syzygium jambos* ^a,b^	Myrtaceae	Rose apple	Tree	Asia	1915	12	0.013	3.26
52	*Thunbergia fragran*	Acanthaceae	White Lady	Vine	Asia and Africa	1974	62	0.065	4.90
53	*Toxicodendron radicans*	Anacardiaceae	Poison ivy	Vine	Northern America	1981	2	0.002	0.05
54	*Ziziphus mucronata*	Rhamnaceae	Buffalo thorn	Tree	Africa		0	0.000	61.25
	Mean					1965	36	0.037	7.21

### 2.2 Species Distribution Modelling Approach

Occurrence data on the global distribution of QLD emerging IAS were obtained from the Global Biodiversity Information Facility Online website (GBIF 2024) and the Atlas of Living Australia (ALA 2024). GBIF provides access to many georeferenced species distribution records, but the data often contain duplicates, uncertainty and ambiguous centroids. To ensure the data are suitable for SDM, pre‐processing and filtering procedures are essential. We implemented pre‐download constraints for the GBIF download process to eliminate duplicates, observations without coordinates, absence records, coordinates with equal latitude and longitude, corrupted coordinates, observations older than 1990 and raster centroid datasets (Zizka et al. 2019). We also spatially filtered the occurrence points to ensure they were at least 5 km apart. This reduced the negative effects of spatial autocorrelation, which can artificially inflate model accuracy or skew parameter estimates during the SDM analysis (Dormann et al. 2007). The correlative SDMs require absence points, or if not available, pseudo‐absence points or so‐called background points. For each species, we selected 10,000 background points randomly inside a buffer of 1000‐km radius around its presence points (Warren et al. 2020) to exclude the model from being fitted with inaccessible areas.

To model the global climatic niche of our focal species, we accessed the 19 available bioclimatic variables (derived from monthly temperature and precipitation records) of the WorldClim dataset (version 2.1) at a spatial resolution of 2.5 arc‐min, ~4.5 × 4.5 km (Fick and Hijmans 2017). To minimise the multicollinearity effect, the initial set of 19 bioclimatic variables was further reduced to eight: (i) annual mean temperature (BIO1), (ii) temperature seasonality (BIO4), (iii) maximum temperature of the warmest month (BIO5), (iv) minimum temperature of the coldest month (BIO6), (v) annual precipitation (BIO12), (vi) precipitation of the wettest month (BIO13), (vii) precipitation of the driest month (BIO14) and (viii) precipitation seasonality (BIO15). We selected these variables to ensure the predictive models were ecologically relevant and statistically sound. Firstly, the chosen climatic variables capture annual ranges, seasonal variability and extremes of temperature and precipitation. Hence, they are crucial in reflecting a species' capacity to adapt to climatic conditions and represent abiotic constraints shaping species distributions, niche evolution and adaptability at a large scale (Gallagher, Beaumont, et al. 2010; Shabani et al. 2020). Secondly, we calculated the variance inflation factor (VIF) of the variables and ensured that they had low multicollinearity (VIF < 10), thus avoiding model overfitting and unreliable parameter estimates.

We invoked the MaxEnt model of the dismo package (Hijmans et al. 2017) to model species distribution. Briefly, MaxEnt (Phillips et al. 2006) is a machine learning algorithm that estimates the probability of occurrence of a species in contrast to the background (pseudo‐absence) environmental conditions. In a landscape, MaxEnt uses a maximal entropy function to estimate the probability of occurrence based on the environmental characteristics of the habitats where the species is known to be present (Elith et al. 2011). We used MaxEnt's default settings, as recommended by Phillips et al. (2006), who found that tuning the default settings for a diverse dataset of 226 species across six regions resulted in good predictive performance. Moreover, Valavi et al. (2022) in a benchmark study showed that MaxEnt model, when fitted with the recommended default setting, ranks among the high‐performing models, with no significant difference with a fine‐tuned MaxEnt model and even better than biomod framework (Thuiller et al. 2009). In addition, since one of our objectives was to compare habitat suitability of different species and their responses to environmental conditions, we opted to follow a consistent modelling approach across all 54 emerging weed species. To optimise the limited availability of occurrence data and using both globally invaded and native range climatic data for each species, we repeated modelling approach based on the cross‐validation method. The data were divided into 10 equal‐sized folds, and training models were constructed by excluding each fold in turn. The excluded folds were then used to assess the accuracy of the training models considering the area under the curve (AUC) of the receiver operating characteristic (ROC) plots. The AUC values are divided as follows: 0.5–0.6, (poor), 0.6–0.7 (fair), 0.7–0.8 (good), 0.8–0.9 (very good) and 0.9–1.0 (excellent). The higher the AUC value, the better the model performed (Phillips et al. 2006). Using the maximum training sensitivity plus specificity (MaxSS) threshold (Liu et al. 2013), we calculated true skill statistic (TSS) to assess classification accuracy of each species' MaxEnt model and subsequently generated a binary habitat suitability map for each species. We also considered the relative importance of the climatic variables in the species' distribution model based on their contribution to the MaxEnt model.

### 2.3 Assessing Response of Emerging Weeds to Climate Change

To model the distribution of the invasive species considering future climate scenarios, we used projections of the bioclimatic variables for 2050 and 2070 (average for 2041–2060 and 2061–2080, respectively) based on five global circulation models (GCMs)—ACCESS‐CM2, BCC‐CSM2‐MR, IPSL‐CM6A‐LR, MIROC6 and MPI‐ESM1‐2‐HR. For each GCM, we chose two shared socioeconomic pathways (SSP1‐2.6 and SSP5‐8.5) of the 6th Assessment Report of the Intergovernmental Panel on Climate Change (IPCC) for incorporating the future climate scenarios into the model projections. The SSP1‐2.6 is the most optimistic scenario, and SSP5‐8.5 ‐is the most pessimistic climate change scenario. For SSP1‐2.6, the assumption is that global warming would increase by 1.7°C between the years 2041 and 2060 and by 1.3°C–2.4°C between 2081 and 2100. In contrast, SSP5‐8.5 assumes that global warming would increase by 2.4°C between the years 2041 and 2060 and by 3.3°C–5.7°C between 2081 and 2100. The narrative description of SSP scenarios is ‘Suitability’ and ‘Fossil‐fuelled Development’ for SSP1‐2.6 and SSP5‐8.5, respectively (Riahi et al. 2017). Although there is a more optimistic scenario known as SSP1‐1.9, in this study, we focused on these two scenarios to assess the impact of climate change on the distribution of the target weed species under sustainability and uncontrolled development pathways. Accordingly, for each time period and each species, we generated 10 future climatic projections (5 GCMs × 2 SSPs); these GCM projections are averaged for each species in this comparative work.

Predictive models often exhibit reduced reliability when extended beyond the training domain (Elith et al. 2010). This is particularly evident in climate change predictions, where fitted models based on current conditions are projected onto novel future climatic conditions. To address this challenge, Elith et al. (2010) proposed measuring the similarity between new environments (future climatic conditions) and those within the training sample using multivariate environmental similarity surface (MESS) analysis. Following this approach, we generated a MESS map after climate change projection and employed negative MESS values, representing dissimilar areas, to mask future projections. Like the current suitability map, we transformed future climatic projections into binary maps using MaxSS threshold. Finally, we calculated two indices based on the binary maps of current and future projections to quantify the proportion of range change: (i) habitat gain, defined as the number of pixels currently unoccupied but predicted to be suitable for occupation in the future, and (ii) habitat loss, defined as the number of pixels currently suitable but predicted to be unsuitable in the future. Using ArcGIS, we created a cumulative invasion risk map (species richness) by overlaying the presence/absence maps for the 54 focal species (Thuiller et al. 2005).

### 2.4 Climatic Niche Comparison Between Plant Growth Forms

Across the 54 species data and grouped by plant growth form, we carried out a principal component analysis (PCA) using the eight a priori identified bioclimatic variables. PCA (following data transformation, normalisation and creation of resemblance matrix formulation using Euclidean distance) discriminates between the ecological niches (e.g., climate) of species or species groups and has been shown to accurately identify niche overlaps, niche differences and shifts (Broennimann et al. 2012; Eckert et al. 2020). For evidence of climatic (niche) overlap or difference among the plant growth forms of our 54 focal species, we used multidimensional scaling (MDS) and analysis of similarities (ANOSIM) options with the PRIMER (v.7) software (Clarke and Gorley 2015). MDS is an ordination technique that is similar to PCA but uses a different resemblance measure and thus allows estimation of similarities (ANOSIM) between groups of data. ANOSIM is analogous to ANOVA, and it compares the mean difference of ranks within and between groups (in our case, between plant growth forms), generating the statistic R (Clarke and Warwick 2001). R values range from −1 to +1, with negative values and values near 0 indicating similarity among groups, while values approaching *R* = 1 are suggestive of a strong dissimilarity (in our case, climatic difference) among groups. We also used a generalised linear model (GLM) to test for the effect of weed origin (continent) on species distribution in QLD.

### 2.5 Weed Species Ranking for Biosecurity Risk Assessment and Prioritisation

Across the 54 focal species, we performed normalisation on our three species distribution parameters, i.e., on current distribution (derived from ALA/GBIF, etc.), (ii) potential distribution and (iii) range shift (the latter two as predicted by MaxEnt model) in QLD. To achieve this, we converted each parameter to a common range (0–1) using Min–Max scaling, defined as

$$X_{1}=\frac{X-X_{\min}}{X_{\max}-X_{\min}}$$

We then summed these three parameters to derive an index of invasion risk and, consequently, a statewide risk assessment for the focal species (see also Lohr et al. 2015; Osunkoya, Froese, Nicol, Perrett, et al. 2019). We chose the summation approach as the three distribution parameters were found not to be significantly correlated with each other and thus contribute independently to the risk score (FAO 2004; USDA‐APHIS‐PPQ 2004). In the risk assessment procedure, inclusion of potential distribution provides information on how far a weed can spread, while range shift (if any) will standardise such spread prediction in response to climate change (Lozano et al. 2024; Szyniszewska et al. 2024). For example, a particular climate change scenario could render previously unsuitable climates favourable for certain species (Mainka and Howard 2010; Lozano et al. 2024). Ideally, the potential impact of the focal species should be an important inclusion variable in the weed prioritisation exercise, but we lack such data for many species (see also Rockwell‐Postel et al. 2020; Lozano et al. 2024; and the Section 4) and hence was not included.

## 3 Results

### 3.1 General Pattern

With multiple species (54), two future scenarios, two emission pathways, 5GCMs, and multiple ways to analyse the data, we selected a subset of results for this paper (i.e., presented a summary of the full range of outcomes and their averages). This approach allows for an overview of incoming (horizon) weeds and climate change impacts on their realised and potential habitat suitability in the eastern part of Australia with a focus on the QLD. Additional analyses and species‐by‐species results and maps for all scenarios can be found in Appendix S2.

Most of the focal horizon weed species are from the Americas (Mexico, Central and South America, 34/54 species = 63%) (Table 1). The remaining species are evenly distributed between Asia and Africa (~17% each), with none from Europe. No plant taxon (e.g., family) dominates the list. As expected, the majority of our 54 species are of recent incursions, as indicated by their current small population size and extent (i.e., low number of 4.5 × 4.5 km pixels infested in QLD, Table 1, Appendix S2 and S3). The degree to which different species have already spread in Queensland varies significantly. Noteworthy are low occurrence records (< 0.02% of total area currently infested) for *Acanthospermum australe* , *Asparagus retrofractus*, *Coix lacryma‐jobi* , *Gliricidia sepium* , *Manihot glaziovii* , *Miconia racemosa* , *Mimosa pigra* , *Opuntia dejecta* , *Opuntia sulphurea* , *Setaria parviflora* , *Sieruela rutidosperma* and *Toxicodendron radicans* to no records for *Artemisia verlotiorum* and *Ziziphus mucronata*. On the other hand, there were moderate to high current distribution records for *Praxelis clematidea*, *Hyparrhenia rufa* and *Chromolaena odorata* (0.2%–0.4% of total QLD areas currently infested).

### 3.2 Species Distribution and Climatic Requirements

**FIGURE 1:**
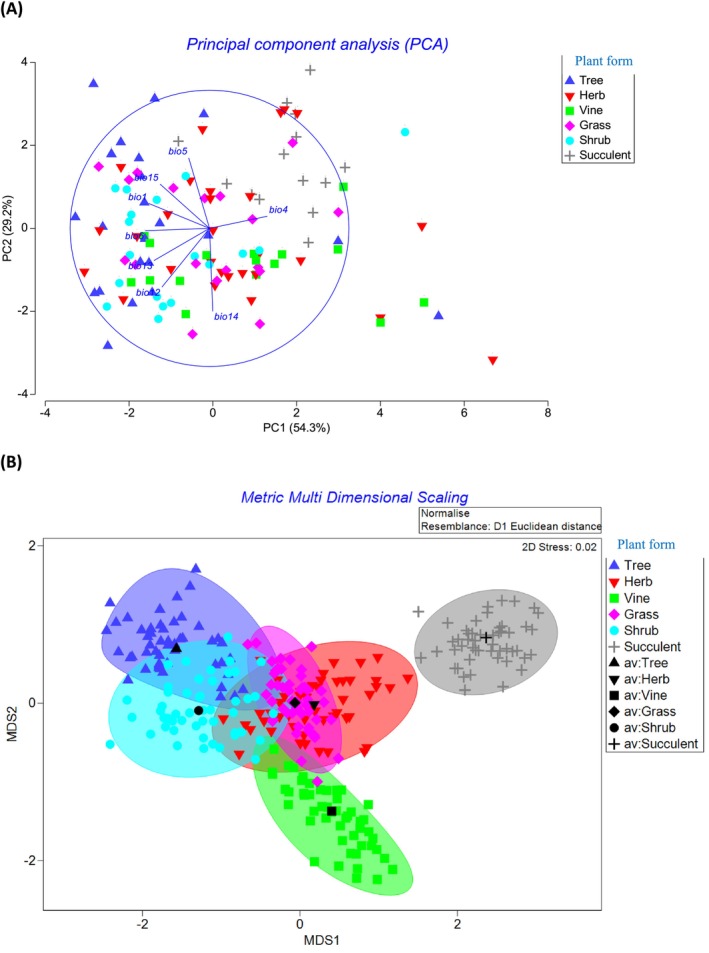
Ordination of 54 emerging invasive alien species of QLD, Australia based on global bioclimatic data of four precipitation and four temperature‐related variables that are derived from their invaded and native ranges. Species have been grouped by plant growth form. (A) Species ordination using principal component analysis (PCA) and (B) ordination using metric multidimensional scaling (MDS) and bootstrap averaging technique to indicate the centroid point (black symbol) for each plant group. The direction and magnitude of influence of the climatic variables (BIO1, BIO4, BIO5, BIO6, BIO12, BIO13, BIO14, BIO15 and BIO16) are indicated on the biplot.

Of the eight bioclimatic variables used, temperature seasonality (BIO4) was the main predictor of habitat suitability for 34 out of 54 (63%) focal emerging IAS, making an overall mean value of 35.14% contribution to the total variation in the dataset (Figure 1A, Appendix S3). Other major contributors, in decreasing order, are minimum temperature of the coldest month (BIO6‐18.96%) and precipitation seasonality (BIO15‐10.32%). Precipitation of the wettest month (BIO13) made the least contribution (4.07%). Note, however, that the relative contribution of the bioclimatic variables varied significantly across the focal species and was largely unaffected (*p* > 0.05) by plant growth form (Appendix S3).

Ordination of focal species distribution based on their bioclimatic data in QLD indicated that Axes I and II—having captured 54% and 29%, respectively, of the total variation in the dataset are—enough to explain the extent of the spatial variation in the dataset (Figure 1A). Axis I was majorly a temperature axis—with temperature seasonality (BIO4), minimum temperature of the coldest month (BIO6), annual mean temperature (BIO1) and precipitation of the wettest month (BIO13) making the greatest contribution to the variation in distribution and habitat requirements of the 54 focal species. Axis II was both precipitation and temperature gradients—mainly explained by precipitation of the driest month (BIO14) and maximum temperature of the wettest month (BIO5) acting in opposing directions. Hence, an increase in temperature seasonality (BIO4) tends to favour the establishment of succulents relative to any other plant growth forms. In contrast, an increase in the minimum temperature of the coldest month (BIO6), an increase in annual mean temperature (BIO1) and to some extent increases in precipitation seasonality (BIO15) and precipitation of the wettest month (BIO13) favour the occurrence of trees (Figure 1, Appendix S4). A combination of increasing annual precipitation (BIO12) and increasing precipitation of the driest month (BIO14) tends to favour the establishment of vines. Climatic requirements of herbs and grasses (and to a limited extent that of shrub) appeared diffused. In all, succulent plants appeared to have habitat requirements (i.e., climatic variables) that are distinctly different from those of other growth forms—a trend confirmed by both ANOSIM (Global *R* = 0.202, *p* = 0.001) and bootstrap averaging estimation technique (Figure 1B).

### 3.3 Model Fit, Species Range Size and Range Shift

Total global occurrence samples for the 54 focal emerging (horizon) weeds varied between species (Appendix S3) but were generally high, with an overall mean of 918 and 229 as training and test samples, respectively. Note that there are extreme cases of low (e.g., *Acaciella glauca* [23 samples] and *Artemisia verlotiorum* [34 samples]) and very high total samples (e.g., *Arundo donax* [7996 samples], *Chromolaena odorata* [4315 samples] and *Toxicodendron radicans* [10,639 samples], Appendix S3). The AUC and TSS values varied among species but were consistently high across all species (mean AUC = 0.90 and mean TSS = 0.67) (Appendix S3).

**FIGURE 2:**
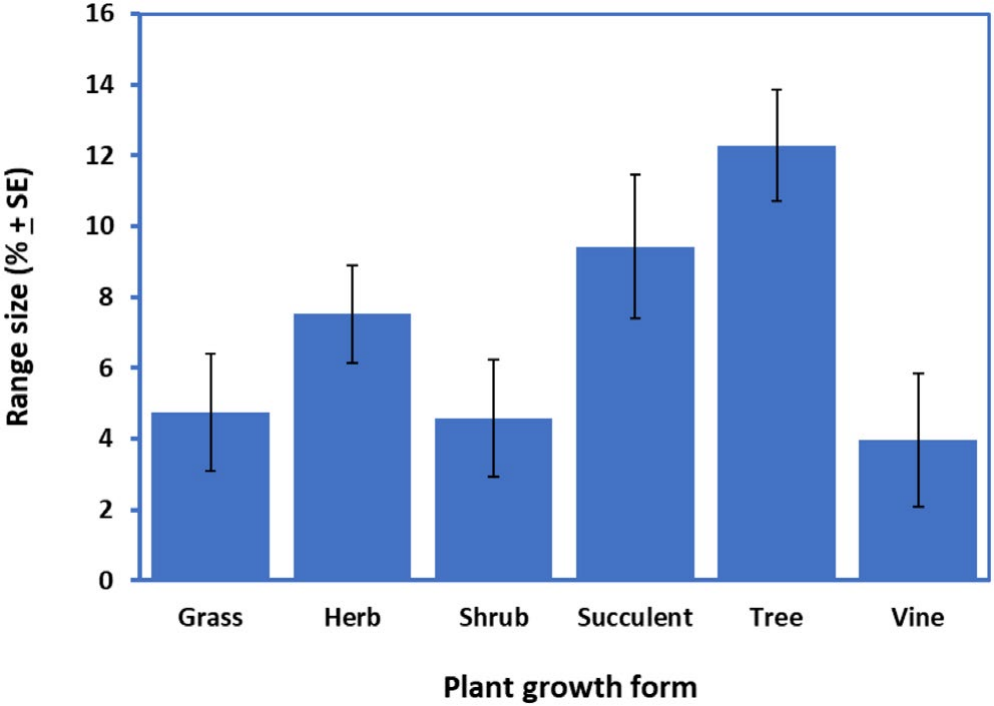
Range size (potential, and as a % of QLD area) by plant growth form of the 54 emerging weeds of QLD. Data have been pooled across species.

Under the current climatic conditions, *Ziziphus mucronata* (a tree), *Florestina tripteris* (a herb) and *Cereus hildmannianus* (a succulent) have the highest extent of climatically suitable habitats in QLD (61.3%, 40.5% and 28.5%, respectively), while *Arundo donax* (a grass), *Opuntia sulpurea* (a succulent) and *Toxicodendron radicans* (a vine) had the least (all < 0.05%) (Table 1). Across all our 54 tested species, the mean area of current suitable habitats (potential) based on the eight climatic variables was 7.21% ± 0.69% of QLD. However, the ranges varied significantly (*p* < 0.05) among species and between plant growth forms, being of the order: tree (12.3%), succulents (9.4%) > herb (7.5%) ≥ shrubs (4.6%) ≥ grass (4.8%) ≥ vines (3.98%) (Figure 2).

In response to climate change (Table 2), the highest positive range changes, i.e., range shifts (300%–900% increase from current size), were predicted for *Opuntia elata* , *Murraya koenigii* , *Dalbergia sissoo* , *Khaya senegalensis* and *Sieruela rutidosperma* and the lowest/neutral changes (< 5% change) were for *Miconia racemosa* , *Praxelis clematidea*, *Cereus hildmannianus* , *Diplachne uninervia* , *Florestina tripteris* , *Spathodea campanulata* , *Ziziphus mucronata* and *Cenchrus purpureus*. Significant losses in response to climate change (> 60% from current size) were predicted for *Artemisia verlotiorum*, *Opuntia sulphurea* , *Cylindropuntia fulgida* , *Asparagus retrofractus*, *Arundo donax* , *Heteranthera reniformis* and *Paspalum mandiocanum* (Table 2).

**TABLE 2:** Response of Queensland emerging weeds to climate change (lost, gain, shift and stability) based on MaxEnt predictions. Range dynamics have been pooled across future timelines, GCMs and RCP scenarios. Overall predictions are based on mean range shift values.

Sp. No.	Species name	Climate change effect															
		Range lost as a percentage of current (potential) size			Range gain as a percentage of current (potential) size			Range shift (gain to loss, % of current [potential] size)			Range stability (future habitat size after loss, % of current [potential] size)			Potential range size after climate change effect by 2050/2070 (% of QLD)			Overall prediction in range size relative to current (potential) condition
		Mean (%)	95% CI		Mean (%)	95% CI		Mean (%)	95% CI		Mean (%)	95% CI		Mean (%)	95% CI		
			Lower	Upper		Lower	Upper		Lower	Upper		Lower	Upperer		Lower	Upper	
1	*Acaciella glauca*	17.3	8.5	26.0	1.0	−69.4	71.4	−16.3	−87.2	54.6	4.7	4.1	5.2	4.7	2.4	7.1	Reduction
2	*Acanthospermum australe*	31.2	22.5	40.0	1.9	−68.5	72.3	−29.4	−100.	41.5	4.6	4.1	5.1	4.7	2.4	7.1	Reduction
3	*Amphilophium crucigerum*	0.1	−8.7	8.8	21.8	−48.6	92.1	21.7	−49.2	92.6	10.5	10.0	11.1	12.8	10.5	15.2	Gain
4	*Artemisia verlotiorum*	61.6	52.8	70.3	0.0	−70.4	70.4	−61.6	−132	9.3	1.8	1.3	2.4	1.8	−0.5	4.2	Reduction
5	*Arundo donax*	63.0	54.3	71.7	1.5	−68.9	71.9	−61.5	−132	9.4	0.0	−0.5	0.5	0.0	−2.3	2.4	Reduction
6	*Asparagus retrofractus*, syn *A. africanus*	90.3	81.6	99.0	1.3	−69.1	71.7	−89.0	−159	−18.1	0.0	−0.5	0.6	0.0	−2.3	2.4	Reduction
7	*Barleria repens*	15.0	6.2	23.7	9.9	−60.5	80.3	−5.1	−76.0	65.8	2.9	2.4	3.4	3.2	0.9	5.6	Reduction
8	*Bignonia magnifica*	29.9	21.2	38.7	0.2	−70.2	70.6	−29.7	−100	41.2	2.8	2.3	3.3	2.8	0.5	5.2	Reduction
9	*Cabomba caroliniana*	27.0	18.2	35.7	30.4	−40.0	100.8	3.4	−67.4	74.3	0.3	−0.2	0.9	0.5	−1.9	2.8	Gain
10	*Cenchrus purpureus*	14.5	5.8	23.3	8.2	−62.2	78.6	−6.3	−77.2	64.6	1.6	1.1	2.1	1.7	−0.6	4.1	Reduction
11	*Cereus hildmannianus* syn *C. uraguayanus*	6.3	−2.5	15.0	2.0	−68.4	72.4	−4.3	−75.2	66.6	26.8	26.2	27.3	27.3	25.0	29.7	Reduction
12	*Ceropegia gigantea*	31.1	22.4	39.9	2.2	−68.2	72.5	−29.0	−99.9	41.9	7.8	7.3	8.4	8.1	5.7	10.4	Reduction
13	*Chromolaena odorata*	0.0	−8.7	8.7	74.5	4.1	144.9	74.5	3.6	145.4	6.3	5.7	6.8	10.9	8.6	13.3	Gain
14	*Coffea arabica*	35.1	26.4	43.8	0.3	−70.1	70.7	−34.8	−105	36.1	1.1	0.6	1.6	1.1	−1.3	3.4	Reduction
15	*Coix lacryma‐jobi*	32.9	24.2	41.6	0.0	−70.4	70.4	−32.9	−103	38.0	1.3	0.8	1.8	1.3	−1.0	3.7	Reduction
16	*Cylindropuntia fulgida*	87.7	78.9	96.4	0.1	−70.3	70.5	−87.5	−158.	−16.6	0.4	−0.1	0.9	0.4	−2.0	2.8	Reduction
17	*Dalbergia sissoo*	0.0	−8.7	8.7	256.0	185.6	326.4	256.0	185.1	326.9	10.8	10.3	11.3	38.5	36.2	40.9	Gain
18	*Diplachne uninervia*	59.3	50.5	68.0	37.9	−32.5	108.2	−21.4	−92.3	49.5	0.4	−0.1	0.9	0.8	−1.6	3.1	Reduction
19	*Dyschoriste nagchana*	6.2	−2.5	14.9	19.3	−51.1	89.7	13.1	−57.8	84.0	5.2	4.7	5.7	6.3	3.9	8.6	Gain
20	*Echinochloa polystachya*	0.0	−8.7	8.7	49.2	−21.2	119.5	49.2	−21.7	120.1	14.8	14.3	15.3	22.0	19.7	24.4	Gain
21	*Elephantopus mollis*	31.9	23.1	40.6	0.2	−70.2	70.6	−31.7	−102	39.2	2.0	1.4	2.5	2.0	−0.4	4.3	Reduction
22	*Florestina tripteris*	12.0	3.2	20.7	7.7	−62.7	78.1	−4.3	−75.2	66.6	35.7	35.1	36.2	38.8	36.4	41.1	Reduction
23	*Gliricidia sepium*	0.0	−8.7	8.7	57.0	−13.4	127.4	57.0	−13.9	127.9	5.5	5.0	6.1	8.7	6.3	11.1	Gain
24	*Gmelina arborea*	0.0	−8.7	8.7	12.2	−58.2	82.6	12.2	−58.7	83.1	12.1	11.6	12.6	13.5	11.2	15.9	Gain
25	*Heteranthera reniformis*	65.9	57.1	74.6	0.0	−70.4	70.4	−65.8	−136	5.1	1.7	1.2	2.2	1.7	−0.7	4.1	Reduction
26	*Hyparrhenia rufa*	0.0	−8.7	8.7	27.3	−43.1	97.7	27.3	−43.6	98.2	11.7	11.2	12.2	14.9	12.5	17.2	Gain
27	*Indigofera schimperi*	2.5	−6.3	11.2	15.3	−55.1	85.7	12.8	−58.1	83.7	7.4	6.9	7.9	8.6	6.2	10.9	Gain
28	*Ipomoea alba*	1.2	−7.5	9.9	18.6	−51.8	89.0	17.4	−53.5	88.3	7.2	6.7	7.8	8.6	6.3	11.0	Gain
29	*Jatropha curcas*	0.0	−8.7	8.7	22.4	−48.0	92.8	22.4	−48.5	93.3	13.0	12.5	13.5	15.9	13.6	18.3	Gain
30	*Khaya senegalensis*	0.0	−8.7	8.7	244.3	173.9	314.7	244.3	173.4	315.2	2.8	2.3	3.3	9.6	7.2	11.9	Gain
31	*Leonotis nepetifolia*	43.7	35.0	52.5	4.9	−65.5	75.2	−38.9	−109	32.0	1.1	0.6	1.6	1.2	−1.2	3.5	Reduction
32	*Manihot glaziovii*	15.3	6.6	24.1	8.2	−62.1	78.6	−7.1	−78.0	63.8	1.5	0.9	2.0	1.6	−0.8	4.0	Reduction
33	*Mesosphaerum pectinatum*	18.7	10.0	27.5	10.3	−60.1	80.6	−8.5	−79.4	62.4	3.0	2.5	3.5	3.4	1.0	5.7	Reduction
34	*Miconia racemosa*	14.9	6.2	23.7	6.6	−63.8	77.0	−8.4	−79.3	62.5	0.2	−0.3	0.7	0.2	−2.2	2.5	Reduction
35	*Mimosa pigra*	0.0	−8.7	8.7	58.8	−11.6	129.2	58.8	−12.1	129.7	11.4	10.9	11.9	18.1	15.7	20.4	Gain
36	*Murraya koenigii*	0.0	−8.7	8.7	475.7	405.3	546.1	475.7	404.8	546.6	1.8	1.3	2.3	10.5	8.1	12.8	Gain
37	*Neptunia plena*	0.0	−8.7	8.7	73.5	3.1	143.9	73.5	2.6	144.4	10.9	10.3	11.4	18.8	16.5	21.2	Gain
38	*Opuntia dejecta*	26.5	17.8	35.3	13.6	−56.8	83.9	−13.0	−83.9	57.9	9.4	8.9	9.9	11.1	8.8	13.5	Reduction
39	*Opuntia elata*	0.5	−8.3	9.2	297.2	226.9	367.6	296.8	225.9	367.7	0.7	0.1	1.2	2.6	0.3	5.0	Gain
40	*Opuntia sulphurea*	93.5	84.8	102.2	0.0	−70.4	70.4	−93.5	−164	−22.6	0.0	−0.5	0.5	0.0	−2.4	2.4	Reduction
41	*Paspalum mandiocanum*	67.1	58.4	75.9	0.1	−70.3	70.5	−67.0	−137	3.9	0.6	0.1	1.1	0.6	−1.8	2.9	Reduction
42	*Pithecellobium dulce*	0.0	−8.7	8.7	45.3	−25.1	115.7	45.3	−25.6	116.2	15.2	14.7	15.7	22.1	19.7	24.4	Gain
43	*Praxelis clematidea*	7.8	−0.9	16.5	8.7	−61.7	79.0	0.9	−70.0	71.8	7.3	6.8	7.8	8.0	5.6	10.4	Gain
44	*Rhodomyrtus tomentosa*	1.3	−7.4	10.1	82.8	12.4	153.1	81.4	10.5	152.3	0.8	0.3	1.4	1.6	−0.8	3.9	Gain
45	*Rotala rotundifolia*	34.8	26.1	43.6	2.2	−68.2	72.6	−32.6	−103.5	38.3	1.2	0.7	1.8	1.3	−1.1	3.6	Reduction
46	*Schizachyrium microstachyum*	39.1	30.4	47.8	0.2	−70.2	70.6	−38.9	−109	32.0	2.7	2.2	3.2	2.7	0.4	5.1	Reduction
47	*Setaria parviflora*	0.0	−8.7	8.7	98.3	27.9	168.7	98.3	27.4	169.2	5.3	4.7	5.8	10.4	8.1	12.8	Gain
48	*Sieruela rutidosperma*	0.0	−8.7	8.7	151.4	81.0	221.8	151.4	80.5	222.3	0.9	0.4	1.5	2.3	0.0	4.7	Gain
49	*Spathodea campanulata*	10.2	1.4	18.9	10.0	−60.4	80.4	−0.2	−71.1	70.7	3.9	3.4	4.4	4.4	2.0	6.7	Reduction
50	*Stigmaphyllon ciliatum*	15.3	6.6	24.1	6.7	−63.7	77.1	−8.7	−79.6	62.2	0.6	0.0	1.1	0.6	−1.7	3.0	Reduction
51	*Syzygium jambos*	19.8	11.0	28.5	2.6	−67.8	73.0	−17.2	−88.1	53.7	2.6	2.1	3.1	2.7	0.3	5.1	Reduction
52	*Thunbergia fragran*	5.5	−3.2	14.2	21.6	−48.8	92.0	16.1	−54.8	87.0	4.6	4.1	5.1	5.7	3.3	8.0	Gain
53	*Toxicodendron radicans*	28.9	20.2	37.6	1.1	−69.3	71.5	−27.8	−98.7	43.1	0.0	−0.5	0.6	0.0	−2.3	2.4	Reduction
54	*Ziziphus mucronata*	3.8	−5.0	12.5	0.9	−69.5	71.3	−2.9	−73.8	68.0	58.9	58.4	59.5	59.5	57.1	61.8	Reduction
	Mean	21.6	12.9	30.4	42.6	−27.7	113.0	21.0	−49.9	91.9	6.4	5.9	7.0	8.5	6.2	10.9	Gain

**FIGURE 3:**
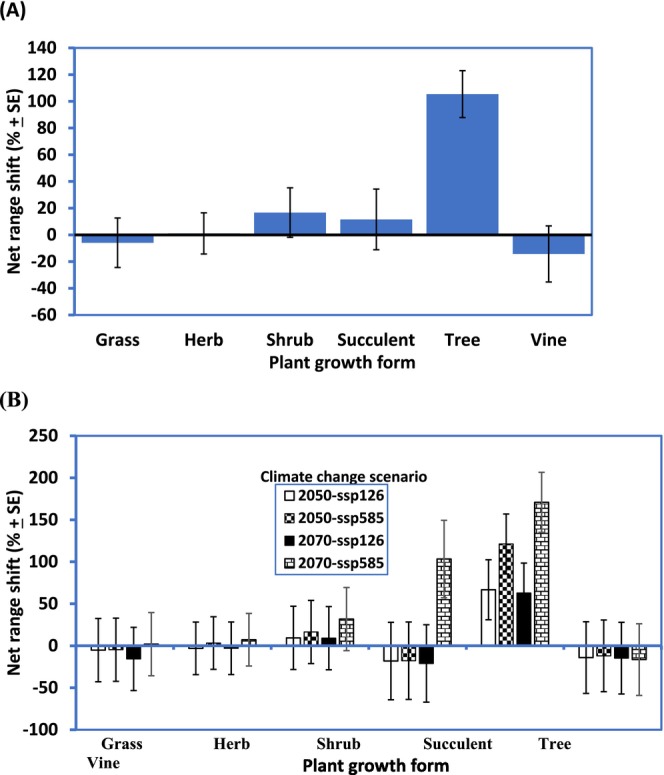
Range shift by growth form (gain–lost) as a % of potential susceptible habitat area of QLD in response to climate change for 54 emerging weeds of the State. For each plant growth form, data have been pooled across species and time scenarios (A) and across species only (B). Analyses based on GLM two‐way ANOVA.

**FIGURE 4:**
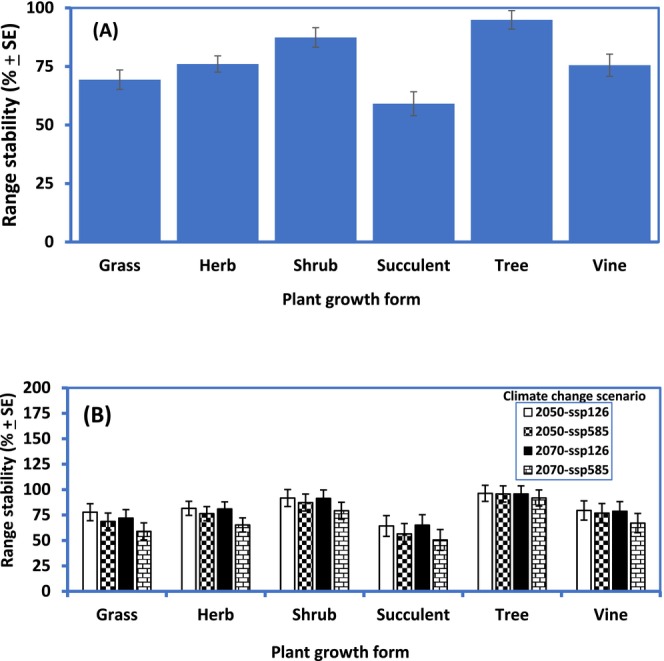
Range stability by growth form (as a % of potential susceptible habitat area QLD) in response to climate change for 54 emerging weeds of the State. For each plant growth form, data have been pooled across species and time scenarios (A) and across species only (B). Analyses based on GLM two‐way ANOVA.

In all, in response to climate change, we found minimal/no changes in size ranges (< 5% change) in 7/54 species (13.96%) (species numbered 7, 9, 11, 22, 43, 49 and 54 in Table 2), negative changes in 25/54 species (46.3%) and positive changes in 22/54 (40.7%) in our focal emerging IAS (Table 2). Range shift was driven more by precipitation variables than changes in temperature (Table 3); in contrast, range stability was driven by temperature variables. The current or potential range sizes have no significant effect (*p >* 0.05) on the prediction of range shift in response to climate change. We found that plant growth form influenced range shift and range stability in response to climate change, to the extent that the greatest positive changes and stability are in trees (Figures 3 and 4). Range shifts in shrubs and succulents, though positive, were non‐significant as their 95% confidence interval bracketed the zero line (Figure 3). In contrast, predicted habitat suitability of grasses and vine will marginally decrease. For trees, the majority of the expansion will be in the Northwest (e.g., in the Gulf of Carpentaria area), in Far North QLD (especially Cape York Peninsula areas) and along the eastern coastal habitats of the State, except for *Ziziphus mucronata* where the increase in habitat suitability is wide—covering both central and southern parts of the State (Appendix S2). The expansion of shrubs also mirrored that of trees—being along the coastal habitats of the eastern side of QLD (e.g., *Chromolaeana odoratum*) and in the Gulf of Carpentaria area (e.g., *Jatropha curcas* and *Mimosa pigra* ). Few of the grasses that will expand their ranges significantly (e.g., *Echinochloa polystachya* [49% expansion] and *Setaria parviflora* [98% expansion]) are predicted to do so mainly within the coastal habitats of the Gulf of Carpentaria and in FNQLD (Table 2, Appendix S2). Despite their current, wider distribution spanning many regions of QLD, some grasses— *Arundo donax* , *Diplachne uninervia* and *Paspalum mandiocanum* —are exceptionally noted to decrease significantly in their habitat ranges (retreating southerly to coastal fringes of the State) in response to climate change (Appendix S2). Vine expansion, if any, in response to climate change will be limited to the coastal eastern fringes of the State, except for *Amphilophium crucigerum* that is additionally predicted to proliferate in Far North Queensland, especially in the Cape York Peninsula area (Appendix S2).

**TABLE 3:** Summary GLM ANOVA of the influence of eight bioclimatic variables of temperature and precipitation (treated as covariates) as drivers of range shift and range stability across 54 emerging weeds of QLD, Australia. Significant effects are in bold and italics. Note that roughly the same trends were observed when data were distilled to individual climate change scenarios and time periods.

Source of variation	Range shift					Range stability				
	Type III sum of squares	df	Mean square	*F*	Sig.	Type III sum of squares	df	Mean square	*F*	Sig.
bio1_AnnMean_temp	19,206.88	1	19,206.88	2.211	0.139	3022.644	1	3022.644	51.692	***< 0.001***
bio4_Temp_seasonality	5149.10	1	5149.10	0.593	0.442	544.461	1	544.461	9.311	***0.003***
bio5_Max_Temp_Warmest_Mth	104,636.10	1	104,636.10	12.047	***< 0.001***	308.34	1	308.34	5.273	***0.023***
bio6_Min_Temp_Colodest_Mth	5718.32	1	5718.32	0.658	0.418	3165.266	1	3165.266	54.131	***< 0.001***
bio12_Ann_Precipitn	169,025.40	1	169,025.40	19.46	***< 0.001***	37.292	1	37.292	0.638	0.425
bio13_Precip_Wettest_Mth	233,843.76	1	233,843.76	26.922	***< 0.001***	91.243	1	91.243	1.56	0.213
bio14_Precip_Driest_Mth	59,612.73	1	59,612.73	6.863	***0.009***	0.474	1	0.474	0.008	0.928
bio15_Precipt_Seasonality	28,511.24	1	28,511.24	3.283	***0.071***	18.845	1	18.845	0.322	0.571
Error	1,797,965.75	207	8685.83			12,104.189	207	58.474		
Total	3,062,198.98	216				29,753.343	216			
*R* ^2^ = 0.394						*R* ^2^ = 0.418				

**FIGURE 5:**
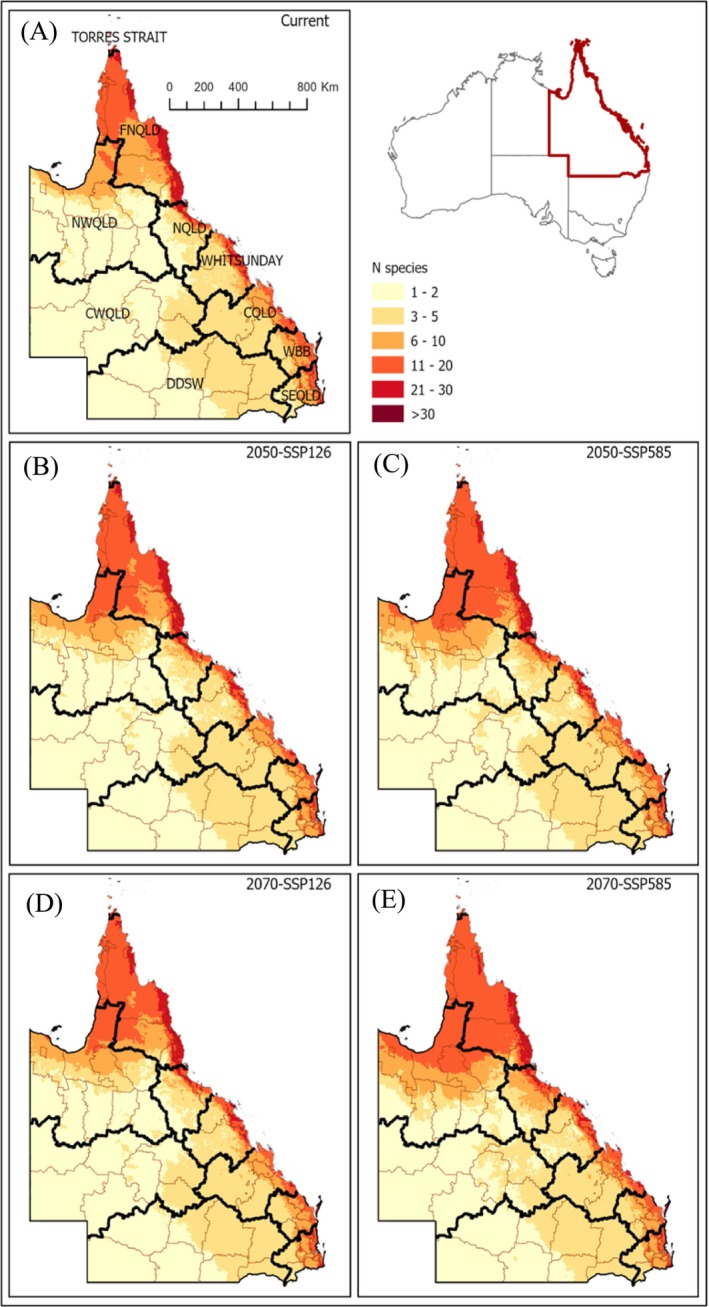
Predicted invasion hotspots using species richness (count per pixel) of 54 emerging weeds across the Queensland landscape: Habitat suitability based on the current situation (A) and in response to time and climate change scenarios (B–E). Local government boundaries are indicated in thin lines, and the 10 Regional Organisations of Council (ROC) groupings are in thick lines. For the meaning of ROC abbreviations, see Appendix S1.

**FIGURE 6:**
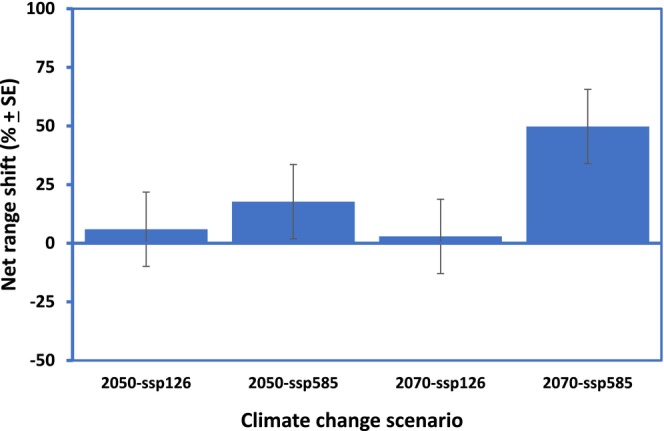
Range shift (as a % of potential susceptible area) of QLD, Australia emerging weed species in response to climate change scenarios. Data have been pooled across the 54 species and are based on GLM one‐way ANOVA.

Overall, and irrespective of climate change scenario and/or plant growth form, the model predicted an increase in habitat range relative to current conditions (positive change: 40.94% ± 7.32%) than a decrease (negative change: 23.05% ± 1.76%), thus suggesting a net gain of 17.89% ± 8.03% for the emerging (horizon) weeds of QLD. Despite the above climate change dynamics, most invasion hotspot areas are projected to remain geographically stable (76.95% ± 1.78%) (Figures 4 and 5)—mainly in the Gulf of Carpentaria, Far North Queensland and along the eastern coast of the state. The model also predicted that the highest magnitudes of range shifts will be for SSP5‐8.5 (16.35%–47.9%) and the lowest in SSP1‐2.6 (2.1%–5.09%) scenarios (Figure 6). For most species studied, close to half of the predicted climatic optimum shifts for 2070 would have occurred by 2050, especially under SSP5‐8.5 (Figures 5 and 6). After accounting for the influence of plant growth form, we detected no effect of geographical origin (i.e., continent) on current and potential habitat suitability nor on range shift dynamics (e.g., for potential habitat suitability: Two‐way GLM ANOVA: *F*_5,12_ = 1.21; *p* = 0.37; for range shift: Two‐way GLM ANOVA: *F*_5,12_ = 0.45; *p* = 0.82).

### 3.4 Species Ranking for Biosecurity Risk Assessment and Prioritisation

The risk ranking (prioritisation) of emerging weeds of QLD based on normalisation and summation of (i) current distribution, (ii) model prediction of potential distribution and (iii) species range shift in response to climate change is given in Table 4, with *Hyparrhenia rufa* , *Praxelis clematidea*, *Ziziphus mucronata* and *Chromolaena odorata* topping the list. Overall, and irrespective of climate change scenarios, species current distribution made the greatest contribution to the risk ranking (Pearson correlation value, *r* = 0.693; *p* < 0.001), followed by potential distribution (*r* = 0.579; *p* < 0.001) and the least by range shift in response to climate change (*r* = 0.40; *p* < 0.001). The ranking of species did not vary significantly among climate change scenarios (*F*_3,91_ = 0.57; *p* = 0.63) but was marginally affected by plant growth form (*F*_5,91_ = 6.71; *p* = 0.07) in the order: trees and succulents > shrubs and herbs > grasses > vines. Note that though trees made up a large proportion of the top 20 prioritised species on the list (7/20), the top of the list is not dominated by a particular plant growth form. We detected no significant association (via correlation analyses, *p* > 0.05) between assigned risk (prioritisation) score and weed arrival time, or between risk score and current range size.

**TABLE 4:** Risk‐based scores of prioritised emerging invasive plant species of Queensland, Australia, ordered along decreasing invasiveness values. Prioritisation is based on the summation of extents of (i) current distribution, (ii) potential distribution and (iii) range shift in response to climate change scenarios.

Rank	Species	Common name	Growth form	Mean risk value	95% confidence interval	
					Upper	Lower
1	*Hyparrhenia rufa*	Thatch grass	Grass	1.26	1.189	1.331
2	*Praxelis clematidea*	Praxelis	Herb	1.23	1.159	1.302
3	*Ziziphus mucronata*	Buffalo thorn	Tree	1.098	1.026	1.169
4	*Chromolaena odorata*	Crucita	Shrub	0.977	0.906	1.049
5	*Florestina tripteris*	Sticky florestina	Herb	0.806	0.735	0.878
6	*Murraya koenigii*	Curry Leaf	Tree	0.688	0.616	0.759
7	*Cereus hildmannianus*	Hedge cactus	Succulent	0.648	0.576	0.719
8	*Dalbergia sissoo*	Himalaya raintree	Tree	0.602	0.53	0.673
9	*Echinochloa polystachya*	Aleman Grass	Grass	0.501	0.43	0.572
10	*Opuntia elata*	Riverina pear	Succulent	0.455	0.384	0.526
11	*Pithecellobium dulce*	Madras thorn	Tree	0.452	0.38	0.523
12	*Spathodea campanulata*	African tulip tree	Tree	0.443	0.372	0.515
13	*Khaya senegalensis*	African mahogany	Tree	0.422	0.35	0.493
14	*Thunbergia fragrans*	White Lady	Vine	0.386	0.314	0.457
15	*Neptunia plena*	Bashful Bush	Herb	0.379	0.307	0.45
16	*Jatropha curcas*	Nutmeg plant	Shrub	0.378	0.306	0.449
17	*Mimosa pigra*	Giant sensitive tree	Shrub	0.36	0.289	0.432
18	*Amphilophium crucigerum*	Monkeys comb	Vine	0.331	0.259	0.402
19	*Gmelina arborea*	Gamhar	Tree	0.328	0.256	0.399
20	*Barleria repens*	Small Bush Violet	Shrub	0.316	0.244	0.387
21	*Opuntia dejecta*	Prickly pear	Succulent	0.299	0.227	0.37
22	*Ipomoea alba*	Moonflower	Vine	0.298	0.227	0.37
23	*Setaria parviflora*	Marsh bristlegrass	Grass	0.297	0.226	0.368
24	*Ceropegia gigantea*	Lantern flower	Succulent	0.29	0.219	0.362
25	*Cabomba caroliniana*	Carolina fanwort	Herb	0.288	0.217	0.359
26	*Dyschoriste nagchana*	Nagchana Bush Violet	Herb	0.286	0.215	0.358
27	*Elephantopus mollis*	Elephant's foot	Herb	0.286	0.215	0.357
28	*Sieruela rutidosperma*	Fringed Spider flower	Herb	0.273	0.202	0.345
29	*Gliricidia sepium*	Gliricidia	Tree	0.26	0.189	0.331
30	*Indigofera schimperi*	Schimper's indigo	Herb	0.258	0.187	0.33
31	*Cylindropuntia fulgida*	Boxing glove cactus	Succulent	0.244	0.172	0.315
32	*Leonotis nepetifolia*	Christmas candlestick	Herb	0.236	0.165	0.308
33	*Rhodomyrtus tomentosa*	Rose myrtle	Shrub	0.223	0.152	0.295
34	*Acaciella glauca*	Redwood	Tree	0.219	0.147	0.29
35	*Heteranthera reniformis*	Kidneyleaf Mud Plantain	Herb	0.219	0.148	0.29
36	*Mesosphaerum pectinatum*	Comb hyptis	Herb	0.219	0.147	0.29
37	*Coffea arabica*	Coffee	Shrub	0.205	0.134	0.276
38	*Schizachyrium microstachyum*	Little bluestem	Grass	0.201	0.13	0.272
39	*Cenchrus purpureus*	Elephant grass	Grass	0.2	0.129	0.271
40	*Paspalum mandiocanum*	Broadleaf Paspalum	Grass	0.196	0.125	0.267
41	*Acanthospermum australe*	Spiny‐bur	Herb	0.192	0.12	0.263
42	*Bignonia magnifica*	Glow vine	Vine	0.187	0.116	0.259
43	*Arundo donax*	Giant reed	Grass	0.183	0.111	0.254
44	*Syzygium jambos*	Rose apple	Tree	0.173	0.101	0.244
45	*Rotala rotundifolia*	Dwarf Rotala	Herb	0.144	0.072	0.215
46	*Diplachne uninervia*	Mexican sprangletop	Grass	0.14	0.068	0.211
47	*Manihot glaziovii*	Ceara rubber tree	Shrub	0.133	0.062	0.204
48	*Stigmaphyllon ciliatum*	Orchid vine	Vine	0.12	0.049	0.192
49	*Artemisia verlotiorum*	Chinese mugwort	Herb	0.116	0.045	0.188
50	*Coix lacryma‐jobi*	Job's Tears	Grass	0.114	0.043	0.186
51	*Miconia racemosa*	Camasey felpa	Shrub	0.098	0.026	0.169
52	*Toxicodendron radicans*	Poison ivy	Vine	0.079	0.007	0.15
53	*Asparagus retrofractus*	Ming Asparagus fern	Vine	0.023	−0.049	0.094
54	*Opuntia sulphurea*	Sulphur cactus	Succulent	0.022	−0.049	0.093

## 4 Discussion

Where feasible, the early detection and control of invasive plants before they become widely established can be cost‐effective and highly desirable. A crucial factor influencing any decisions to commit to control operations is knowledge of the risk posed by the full suite of potentially invasive species. This knowledge facilitates ranking and prioritisation of targets, and the efficacy of such a prioritisation exercise is maximised when climate change and other anthropogenic disturbances are also taken into consideration (Jarnevich et al. 2023; Szyniszewska et al. 2024). ENMs, as applied in this study using the MaxEnt model, are traditionally calibrated using environmental data from both native and invaded ranges and then projected onto other regions/continents to predict areas likely open to invasions (Phillips et al. 2006; Elith et al. 2010). Similarly, Shabani and Kumar (2015) showed that utilising complete distribution data, including both native and exotic occurrences, is the preferred approach when the objective is to map the future distribution of invasive species. An essential consideration when projecting distribution models to new data is the dissimilarity of environmental conditions, particularly for invasive species prone to range shifts in novel environments (Elith et al. 2010). To address this issue, we implemented the multivariate environmental similarity surface (MESS) analysis in our climate change projections. This approach enabled us to exclude novel habitats that are likely to result in extrapolation due to their distinct climatic conditions compared to the species' current range (Elith et al. 2010). Our use of this correlative modelling approach and incorporation of various climate change scenarios strengthened the utility of the prioritisation exercise undertaken and the ensuing watch list ranking generated (Downey et al. 2010; Jarnevich et al. 2023).

In all, it appeared that increased tolerance to certain abiotic factors will encourage IAS establishment, range stability and/or expansion and plant group differentiation. For succulents, these are extreme values of BIO4, BIO5 and BIO12, i.e., increased seasonality of temperature, increased maximum temperature of the warmest month and decreased annual rainfall (Figure 1, Appendix S4). This is not surprising as all our focal succulents (except *Ceropegia gigantea*) are from deserts of South and North America where extreme temperature and aridity are the order of the day (Pillet et al. 2022). The habitat envelopes of our focal succulents in the invaded ranges of QLD appeared geographically wide and varied (Appendix S2). However, the prediction of marginal range shift and lowest range stability (Figures 3 and 4) for succulents (compared to the other growth forms) was surprising as expected future climates with hotter and drier climates are usually predicted to favour species with Crassulacean acid metabolism (CAM)—the photosynthetic pathway of most succulents (Pillet et al. 2022). Contributory factors to these observed retreat trends for succulents, except for the SSP5‐8.5 (2070) scenario, could be the following: (i) differences in habitat availability/occupied between the invaded ranges of QLD and their native ranges as matching climatic conditions of the native range may be lacking in the invaded range of QLD (Gallagher, Beaumont, et al. 2010; Gallagher, Hughes, et al. 2010; Pillet et al. 2022) and (ii) increased intensity of rainfall and humidity (due to climate change) despite higher temperature as succulents tend to thrive better in hotter and drier, rather than wetter climates (Pillet et al. 2022). Only *Opuntia elata* was predicted to thrive and expand (297% increase relative to current condition). The projected range gain for this species can be attributed solely to the higher climatic suitability in the future, which favours its ecological niche and climatic requirements. Though not considered in the study, other contributory factors to the projected success of *Opuntia elata* may be attributed to a combination of its succulency, CAM metabolism, highly competitive ability, multiple reproductive strategies (e.g., clonal reproduction for local dominance and persistence) and human‐induced propagule pressure like nursery sale (Barbosa et al. 2017).

The habitat range/climatic envelope of emerging invasive trees in QLD appeared opposite to that of the invasive succulents (Figure 1). Interestingly, trees were predicted to have the largest positive range shift of all the plant growth forms examined. Aside from the expectation that similar but opposing climatic reasons adduced for succulents will play out for trees, there is evidence that trees have a much larger dispersal range (Clark 1998; Higgens et al. 2003; Broennimann et al. 2006). These factors coupled with the greatest affinity for increased preference for more than half of the environmental variables used in the model (increasing annual mean temperature (BIO1), increasing minimum temperature of the coldest month (BIO6), increasing precipitation of the wettest month (BIO13) and increasing precipitation seasonality (BIO15)), most of which aligned with future climate change scenarios (Stocker et al. 2013; Shabani et al. 2020), ensure that trees, as a focal group, can establish, thrive, spread much faster and hence exhibit the highest range and associated dynamics (size, stability and shift) than any other plant group now and into the future (Figure 4, Appendix S4). Additionally, in our analyses, we observed that range shift/gain in response to climate change is driven more by precipitation than by temperature variables (Table 3) and trees were the winners (Figure 3). In general, QLD being a subtropical region, experiences higher volatilities (extremities) in rainfall than in temperature (http://www.qld.gov.au/environment/climate/climate‐change/); this observed climatic dynamics could have also contributed to the predicted proliferation of invasive trees at the expense of other plant growth forms. Further studies could explore this conclusion in greater detail.

The remaining plant growth forms (grasses, vine, shrub and herb) did not show signs of significant range shift or preference for any environmental variable. Note, however, for grass species that were predicted to significantly expand their ranges ( *Echinochloa polystachya* , *Hyparrhenia rufa* and *Setaria parviflora* , with 49%, 27% and 98% increase, respectively; see Table 2), the main climate drivers were tolerance to increasing temperature seasonality (BIO4) and increasing minimum temperature of the coldest month (BIO6). Our finding for *Setaria parviflora* is similar to that reported by Chuine et al. (2012) for the same species. Suitable climate space for some of the tested grasses (e.g., *Arundo donax* , *Diplachne uninervia* and *Paspalum mandiocanum* ) with known infestations in subtropical and tropical areas of the state will contract towards the coast (Appendix S2), suggesting that climatic conditions in inland areas may become less suitable by 2050/2070 for invasive grasses (see also Gallagher et al. 2013). It is also likely that this latter set of invasive grasses lacks the physiological characteristics for tolerance to increasing temperatures (Barbosa 2016) as the climate warms up. It is fair to conclude that generality cannot be drawn as per the influence of a particular set of climatic variables on range shifts of IAS or plant growth form because responses are species and landscape (i.e., context) specific.

Overall, the model predicted that ~7.2% of QLD area will be climatically suitable for our 54 focal species, which is projected to increase to 8.4% in response to climate change. Most of the positive range shift in QLD is predicted to occur along the eastern coastlines and the Gulf area of FNQLD and NWQLD (Figure 5). This finding, coupled with the proximity of FNQLD (and indeed the whole top end of Australia) to neighbouring oceanic islands and the States of Papua New Guinea (PNG) and Indonesia, will suggest that Biosecurity QLD must continue to step up its surveillance in the regions, as these are vulnerable pathways of introduction of IAS. The ecological systems in these two top end regions of the state (i.e., NWQLD and FNQLD) are unique: the interior of NWQLD consists of vast Mitchell grasslands (the xerophytic *Astrebla* spp.) interspersed with *Acacia* trees and *Eucalyptus* and *Melaleuca* woodlands (tropical savannah). The Gulf country (of FNQLD and NWQLD) needs to be protected from range‐expanding IAS, especially alien trees. Thus, from the results of the habitat suitability work, we can expect global climate change to increase the capacity of alien plant species to invade, thrive and expand into these areas while lowering native community resistance to invasion by disrupting the dynamic equilibria that maintain native communities (see Kriticos et al. 2003; Ngugi and Neldner 2024).

With climate change, the majority of invasion hotspot areas for emerging weeds were projected to remain geographically stable by 2050 (Figures 4 and 5). Invasion hotspots in Australia (O'Donnell et al. 2012) and the eastern USA (Evans et al. 2024) indicated similar geographic stability despite differences in regions and invasive species investigated (see also Barbosa 2016; Lopes et al. 2023; Puchałka, Paź‐Dyderska, Jagodziński, et al. 2023 for similar reports on invasive plant species of Neotropics and Europe). Together, these findings suggest that stability in invasion hotspots may be a general pattern in response to climate change expected in the 21st century. Thus, current invasive plants (whether emerging or established) will not disappear with climate change, but invasion risk reduction for some regions of the state (e.g., inland areas contiguous to coastal communities of the SE and central QLD despite their increasing human population relative to FNQLD and the Gulf area of NWQLD) offers opportunities for coordinated conservation and restoration efforts in the intermediate future. In short, areas predicted to no longer be suitable for invasive plants can be targeted for adaptive management via active restoration using desired native plants (Bradley et al. 2023).

### 4.1 Biosecurity Risk Assessment and Management

Very few studies have combined SDM and climate change scenarios in the scanning, risk assessment and prioritisation of newly emerging weeds (e.g., Westbrooks 2004; Roger et al. 2015; Hulme 2017); not doing so results in suboptimal risk assessment and prioritisation (Downey et al. 2010; Jarnevich et al. 2023; Szyniszewska et al. 2024). To derive an index of prioritisation for the 54 emerging weed species of QLD, we used a simple summation procedure combining current occurrence records with predicted potential range and mean response to two climate change scenarios. Other aggregation procedures (e.g., different weightage assignment) might optimise the index better, but this is still highly debatable (see Caton et al. 2018; Osunkoya, Froese, and Nicol 2019). Our derived risk index information will be useful in management decisions for (i) pre‐emptive ‘watch‐listing’ where species are not yet present in QLD, (ii) local eradication where populations are still very small and removal is technically and economically feasible or (iii) preventive containment, if the emerging weed species has spread to the point where complete eradication is no longer feasible. Our specific recommendation on each species is made along with the rankings generated (Table 4). In general, the top four species with prioritisation index values > 1 ( *Hyparrhenia rufa* , *Praxelis clematidea* and *Chromolaena odorata* )—being already present with large, multiple population foci (exception is *Ziziphus mucronata*), are challenging to manage and strategic containment of dispersal, or pre‐emptive development of biological control agents, may be the only viable control options. Unlike the other 53 species assessed, one species, *Z. mucronata*, appears to be currently absent from Queensland, as we were unable to confirm its presence from herbarium data. If this is in fact the case, it can be moved onto a pre‐border watch list. Z. *mucronata* is still considered high risk, as it was assigned a high prioritisation score due to its predicted large potential habitat range (61% of QLD) and minimal change (high stability) in response to climate change. The remaining species (especially those ranked in the bottom 15, e.g., *Acanthospermum australe* , *Opuntia sulpurea—*see Table 4) are candidates for listing as ‘restricted biosecurity matter’ under the Queensland Biosecurity Act 2014. This would not only prohibit sale but also impose a clear legal obligation on landowners to take all reasonable steps to reduce the risk of these species on their land and, thereby, reduce the risk of dispersal.

Our prioritisation list is important as it enables efforts to be targeted at highest risk species, particularly in cases where targets are being actively sold for horticulture (garden trade) or habitat restoration. However, this statewide list needs to be reviewed periodically, especially in view of the fact that potential preventative impacts (be it ecological, cultural, human‐health related or economic) and identified pathways have not been incorporated into the prioritisation index but are known to be strong drivers of management decisions (Rockwell‐Postel et al. 2020; Bradley et al. 2023; Osunkoya et al. 2022). Impact data are currently being compiled using CABI (2024) Online website and Global Invasive Species Database (GISD 2018), but we noticed a dearth of impact information for many of our focal species (see also Rockwell‐Postel et al. 2020; Lozano et al. 2024). No doubt, the area investigated (QLD) is huge—spanning an area ~1.853 million km^2^, and hence management decisions like that of established IAS may have to be locally/regionally specific (Osunkoya, Froese, and Nicol 2019; Osunkoya et al. 2020). The fine‐tuning (drilling) of the findings reported in this work to local government and regional levels is an aspect we are currently exploring to make the findings and management decisions more context and locally applicable (Osunkoya et al. 2020). We hope this assessment will be used to prioritise preventative and control actions for QLD emerging weeds; a similar one called the ‘invasive range expanders listing tool’ already exists in America (Allen and Bradley 2016).

### 4.2 Caveats on Using SDMs for Introduced Weed Species

The results of the SDMs for introduced weed species in QLD, Australia highlight key ecological and methodological challenges. While our SDMs effectively identified areas of habitat suitability, the inability of the models to capture all occurrence points for certain species, for example, Giant Reed ( *Arundo donax* ), warrants further scrutiny. This discrepancy underscores the complexities inherent in modelling invasive species with dynamic ecological niches and varied dispersal mechanisms (Elith et al. 2010; Tingley et al. 2014). The case of *A. donax* exemplifies how niche dynamics and dispersal strategies influence model outcomes. Some of our modelled weed species have become naturalised in diverse regions across Australia, facilitated by both natural and anthropogenic dispersal mechanisms. The observed distribution of these species suggests a significant niche shift following their introduction, wherein the species have expanded their ecological tolerance and adapted to novel environmental conditions, enabling them to thrive in habitats distinct from their native range (Gallien et al. 2012; Guisan et al. 2017). The observed niche shift has important implications for understanding the invasion biology of weed species in Australia. These species' ability to exploit a broader range of environmental conditions may be attributed to factors such as phenotypic plasticity, genetic variability and biotic interactions in the introduced range (Davidson et al. 2011; Richardson and Pyšek 2011). The limitations of the standard threshold‐based approach to binarising habitat suitability maps highlight the need for tailored modelling strategies for invasive species with complex ecological behaviours. For species like *A. donax* , which exhibit niche shifts, conventional SDMs based on global occurrence data may fail to capture the full extent of their potential distribution in the introduced range. Therefore, it is essential to integrate additional methodologies to enhance the accuracy and ecological relevance of the models. One promising approach involves examining the overlap between native and introduced niches to quantify the extent of niche shift (Broennimann et al. 2012). Reciprocal modelling, wherein models are trained using occurrence data from one range (native or introduced) and evaluated in the other, can provide valuable insights into the ecological adaptability of invasive species (Tingley et al. 2014). This technique can help determine whether the environmental conditions in the introduced range are entirely novel or represent a subset of the species' native niche. Furthermore, incorporating variables related to human activity, such as land use and transport networks, may improve model performance for garden escapee weeds like *A. donax* that are heavily influenced by anthropogenic factors (Gallien et al. 2012).

Overall, SDMs and their predictions possess inherent limitations, including (i) issues with lack of niche saturation (equilibrium) and shifting environmental niches in invaded ranges for many species, (ii) use of a limited number of climate change scenarios, especially of GCM, (iii) lack of explicit consideration of species biological traits, migration rate and/or role of anthropogenic scenarios and (iv) suboptimal performance and inferences where other machine learning tools (e.g., mechanistic model of CLIMEX or Random Forest) are not used simultaneously (for a full treatise on the above, see Gallagher, Beaumont, et al. 2010; Elith et al. 2010; Adhikari et al. 2022; Bradley et al. 2023). In view of the above caveats, our modelling and analyses should be interpreted in the context of data limitations and our assumptions. Our goal, as shown in this study, is to draw generalisations rather than provide details on invasive species effects on a case‐by‐case basis. Nonetheless, in view of the increased uncertainty of species responses to combinations of novel landscapes and climates and imperfection with meteorological forecasting, we recommend that our models are re‐evaluated and revised throughout the lifetime of their projections and, where also feasible, incorporate novel physiological data (Elith et al. 2010).

## 5 Conclusion

We have used the MaxEnt model to predict the potential distribution of 54 emerging weeds in the State of QLD, Australia. With the AUC and TSS values obtained (AUC > 0.9 and TSS > 0.6), the models and predictions performed well. We showed that the potential range and sensitivity of a given species or plant growth form to global environmental changes do not depend upon its geographical origin, making it impossible to use place of origin (country or continent) to forecast, a priori, the performance of emerging weed species in their new/invaded ranges and/or their responses to climate change (see also Gallagher, Hughes, et al. 2010; Osunkoya et al. 2020, 2021 for similar findings for established weeds). The model indicates that the direction and magnitude of shift in species distribution differed among species and plant growth types. Overall, we found evidence that trees are range shifters with the greatest capacity in range dynamics (size, expansion and stability)—possibly in conformity that this group migrates fastest and disperses the furthest (Clark 1998; Higgens et al. 2003), though other plausible explanations, such as propagule pressure or relocation propensity beyond their historical native environmental range (Gallagher, Beaumont, et al. 2010), could also suffice. The remaining plant growth forms (grasses, herbs, shrubs, vines and succulents) showed minor and non‐significant range shifts in response to climate change. Overall, the MaxEnt model shows that climate change is likely to increase the habitat suitability of many (but not all) incoming invasive weeds, especially in the far north, northwest and along coastal fringes of the eastern part of the State. This trend reinforces the hypothesis that warming temperatures will expand the suitable habitats of many invasive plants northward and eastward of the Australian continent (O'Donnell et al. 2012; Gallagher et al. 2013; Bellard et al. 2016). Based on current occurrence and model predictions of range dynamics in response to climate change, we have ranked these species (risk assessment/prioritisation) for policy and proactive management and advocate for re‐assessment in later years as more data on potential impact and pathways become readily available.

## Author Contributions

**Olusegun O. Osunkoya:** conceptualization (lead), data curation (equal), formal analysis (equal), funding acquisition (lead), investigation (equal), methodology (equal), project administration (lead), resources (equal), software (equal), supervision (lead), validation (equal), visualization (lead), writing – original draft (lead), writing – review and editing (lead). **Mohsen Ahmadi:** conceptualization (supporting), data curation (equal), formal analysis (equal), funding acquisition (supporting), investigation (equal), methodology (equal), project administration (supporting), resources (supporting), software (equal), supervision (supporting), validation (lead), visualization (supporting), writing – original draft (supporting), writing – review and editing (equal). **Christine Perrett:** conceptualization (supporting), data curation (lead), formal analysis (supporting), funding acquisition (supporting), investigation (supporting), methodology (supporting), project administration (supporting), resources (supporting), software (supporting), supervision (supporting), validation (supporting), visualization (supporting), writing – original draft (supporting), writing – review and editing (supporting). **Moya Calvert:** conceptualization (supporting), data curation (supporting), formal analysis (supporting), funding acquisition (supporting), investigation (equal), methodology (equal), project administration (supporting), software (supporting), supervision (supporting), validation (supporting), visualization (equal), writing – original draft (supporting), writing – review and editing (supporting). **Boyang Shi:** conceptualization (supporting), data curation (supporting), formal analysis (supporting), funding acquisition (supporting), investigation (supporting), methodology (equal), project administration (supporting), resources (supporting), software (supporting), supervision (supporting), validation (supporting), visualization (supporting), writing – original draft (supporting), writing – review and editing (lead). **Steve Csurhes:** conceptualization (supporting), data curation (supporting), formal analysis (supporting), funding acquisition (equal), investigation (equal), methodology (supporting), project administration (supporting), resources (supporting), software (supporting), supervision (supporting), validation (equal), writing – original draft (equal), writing – review and editing (lead). **Farzin Shabani:** conceptualization (equal), data curation (lead), formal analysis (lead), funding acquisition (supporting), investigation (equal), methodology (lead), project administration (supporting), resources (supporting), software (lead), supervision (lead), validation (lead), visualization (lead), writing – original draft (equal), writing – review and editing (supporting).

## Conflicts of Interest

The authors declare no conflicts of interest.

## Supporting information


**Appendix S1.** Map of Australia and its seven States/Territories (inset) and The State of Queensland (main diagram) (TAS, Tasmania; VIC, Victoria; NSW, New South Wales; QLD, Queensland; SA, South Australia; NT, Northern Territory; WA, Western Australia). For Queensland, the local government boundaries are indicated in thin lines and the 10 Regional Organisations of Councils’ groupings in thick lines. SEQLD, Southeast Queensland; DDSW, Darling Downs Southwest Queensland; WBB, Wide Bay Burnett; CQLD, Central Queensland; CWQLD, Central West Queensland; NQLD, North Queensland; NWQLD, Northwest Queensland; and FNQLD, Far North Queensland (illustration extracted from figure 1 of Osunkoya, Froese, Nicol Perrett et al. 2019).
**Appendix S2.** Spatial distribution of 54 emerging weed species in QLD based on (i) actual occurrence points, (ii) potential range, (iii, iv) response to climate change scenarios in 2050 and (v, vi) response to climate change scenarios in 2070. Note that species are organised by plant growth forms—grass (9 species)–herb (13 species)–shrub (9 species)–tree (10 species)–succulent (6 species)–vine (7 species).
**Appendix S3.** Number of training and test samples, area under the curve (AUC), true skill statistics (TSS) and relative contribution of eight chosen climatic variables influencing MaxEnt predictions of habitat suitability for 54 emerging weed species of QLD. The most important variables are in italics and bold font.
**Appendix S4.** Relative contribution of climatic variables by plant growth form.

## Data Availability

All codes will be freely available. Data are also provided as Supporting Information.
