# Supplementary material for: Climate‐Induced Range Shift and Risk Assessment of Emerging Weeds in Queensland, Australia
Source: Ecol Evol. 2025 Apr 2;15(4):e71043. doi: 10.1002/ece3.71043 (PMC11964613; doi:10.1002/ece3.71043)
Supplement: Supplementary file 1 — Appendix S1. Map of Australia and its seven States/Territories (inset) and The State of Queensland (main diagram) (TAS, Tasmania; VIC, Victoria; NSW, New South Wales; QLD, Queensland; SA, South Australia; NT, Northern Territory; WA, Western Australia). For Queensland, the local government boundaries are indicated in thin lines and the 10 Regional Organisations of Councils’ groupings in thick lines. SEQLD, Southeast Queensland; DDSW, Darling Downs Southwest Queensland; WBB, Wide Bay Burnett; CQLD, Central Queensland; CWQLD, Central West Queensland; NQLD, North Queensland; NWQLD, Northwest Queensland; and FNQLD, Far North Queensland (illustration extracted from figure 1 of Osunkoya, Froese, Nicol Perrett et al. 2019). Appendix S2. Spatial distribution of 54 emerging weed species in QLD based on (i) actual occurrence points, (ii) potential range, (iii, iv) response to climate change scenarios in 2050 and (v, vi) response to climate change scenarios in 2070. Note that species are organised by plant growth forms—grass (9 species)–herb (13 species)–shrub (9 species)–tree (10 species)–succulent (6 species)–vine (7 species). Appendix S3. Number of training and test samples, area under the curve (AUC), true skill statistics (TSS) and relative contribution of eight chosen climatic variables influencing MaxEnt predictions of habitat suitability for 54 emerging weed species of QLD. The most important variables are in italics and bold font. Appendix S4. Relative contribution of climatic variables by plant growth form. [file ECE3-15-e71043-s001.docx]

**Appendices**

Climate-Induced Range Shift and Risk Assessment of Emerging Weeds in Queensland, Australia

**By**

Olusegun O. Osunkoya, Mohsen Ahmadi, Christine Perrett, Moya Calvert, Boyang Shi, Steve Csurhes, & Farzin Shabani

**Appendix S1:** Map of Australia and its seven States/Territories (inset) and The State of Queensland (main diagram) (TAS- Tasmania; VIC- Victoria; NSW- New South Wales; QLD- Queensland; SA- South Australia; NT- Northern Territory; WA- Western Australia). For Queensland, the local government boundaries are indicated in thin lines and the ten Regional Organisations of Councils’ groupings in thick lines. SEQLD- Southeast Queensland; DDSW Darling Downs Southwest Queensland; WBB- Wide Bay Burnett; CQLD Central Queensland; CWQLD- Central West Queensland; NQLD- North Queensland; NWQLD- Northwest Queensland, and FNQLD- Far North Queensland) (Illustration extracted from Figure 1 of Osunkoya et al., 2019b)

Appendix S2: Spatial distribution of 54 emerging weed species in QLD based on (i) actual occurrence points, (ii) potential range, (iii, iv) range response to climate change scenarios in 2050 and (v, vi) range response to climate change scenarios in 2070. Note that species are organised by plant growth form – Grass (9)-Herb (14)-Shrub (8)-Tree (10)-Succulent (6)-Vine (7)

1. GRASS- 9 species
2. (ii) (iii) (iv) (v) (vi)


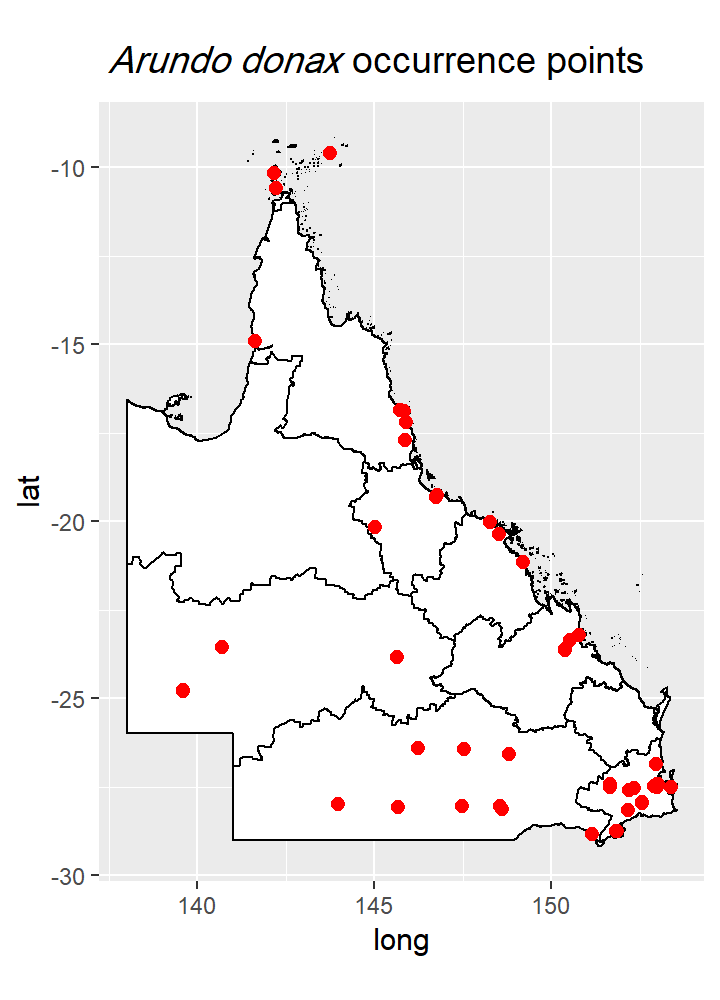

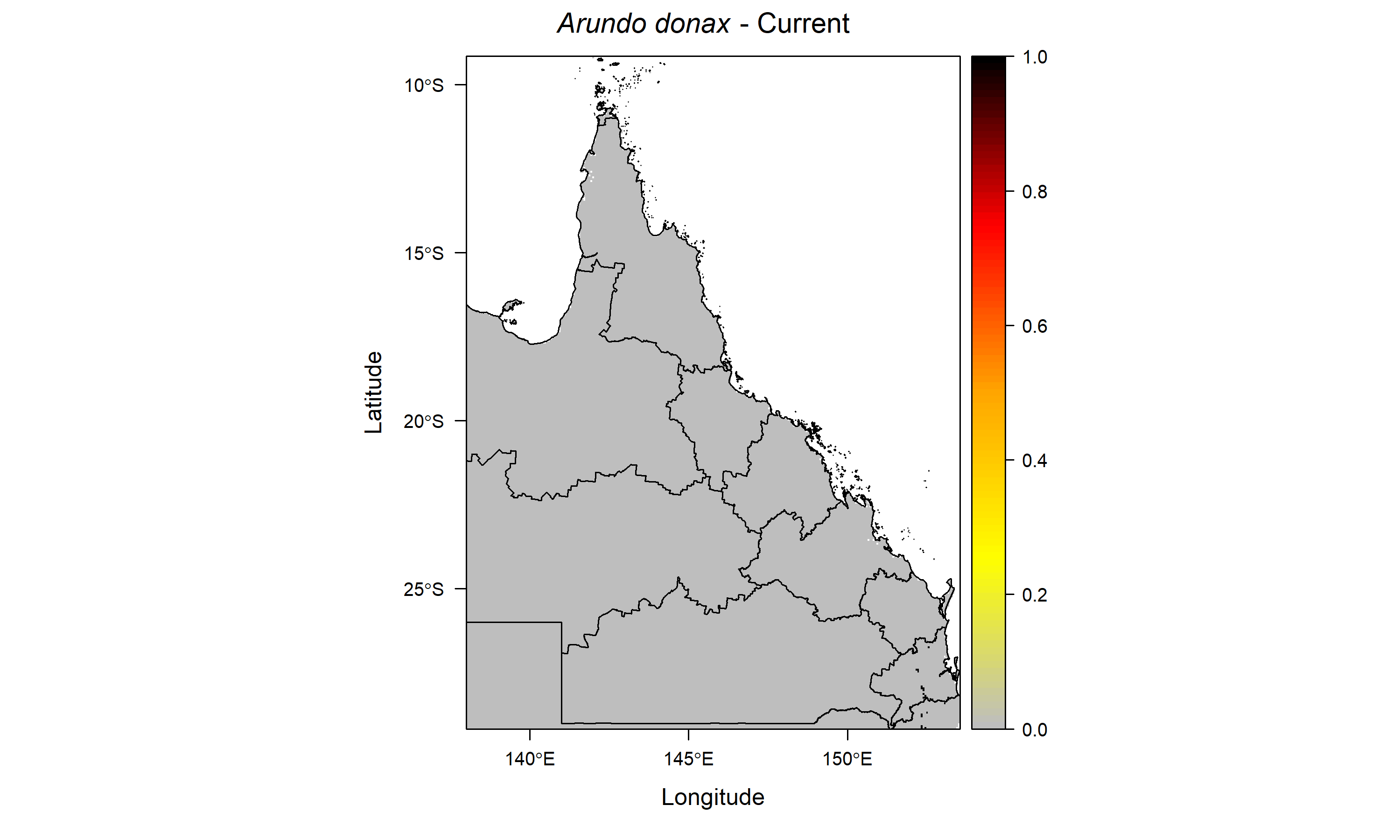

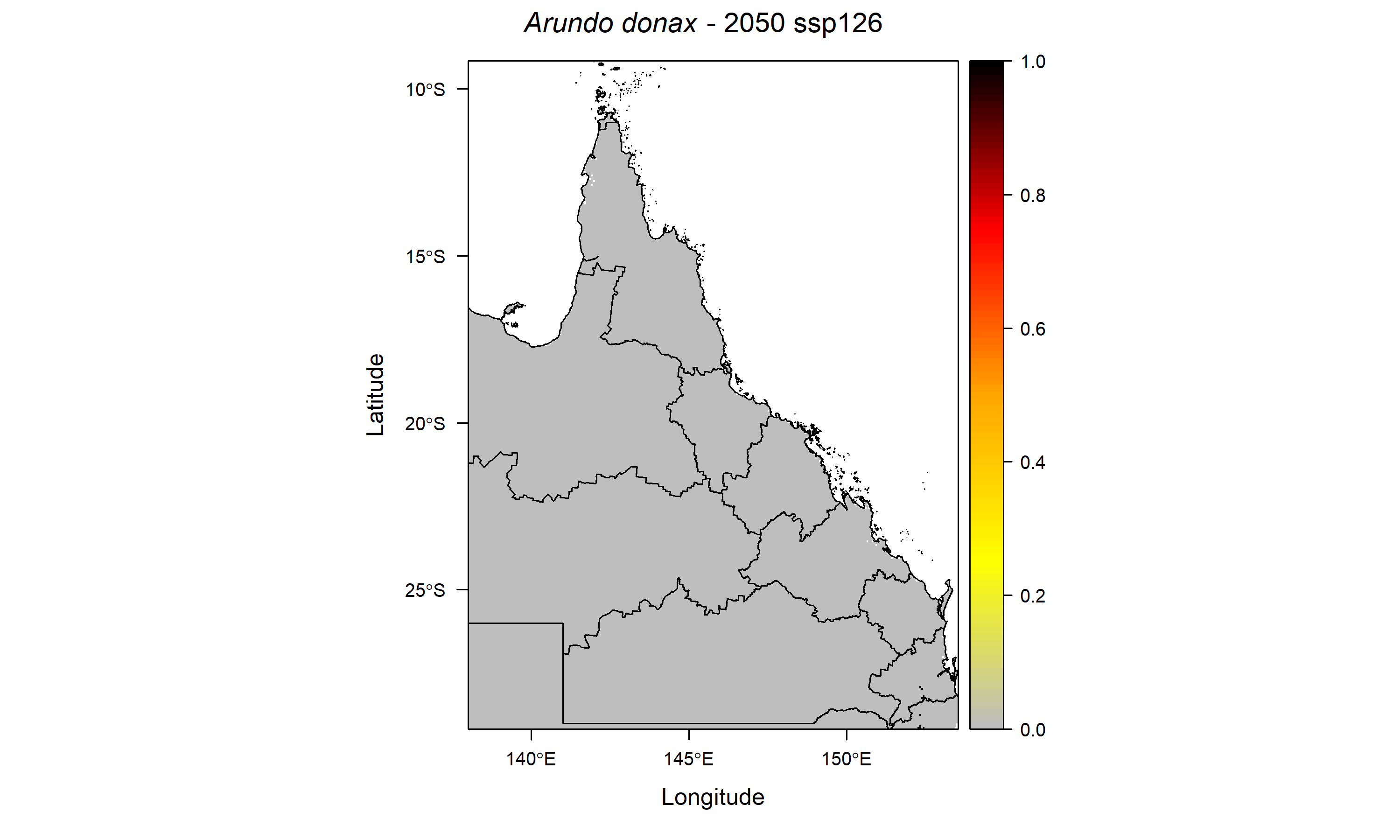

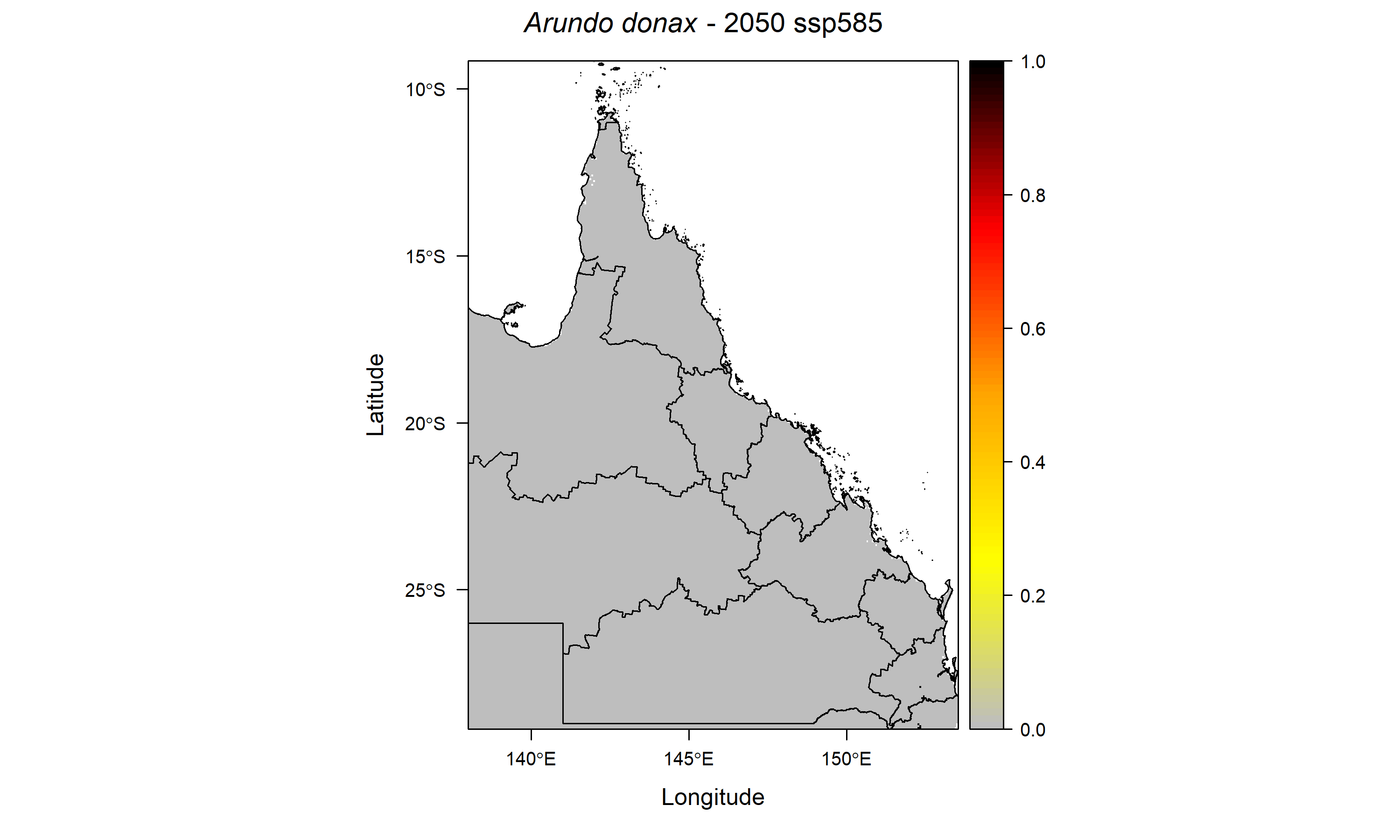

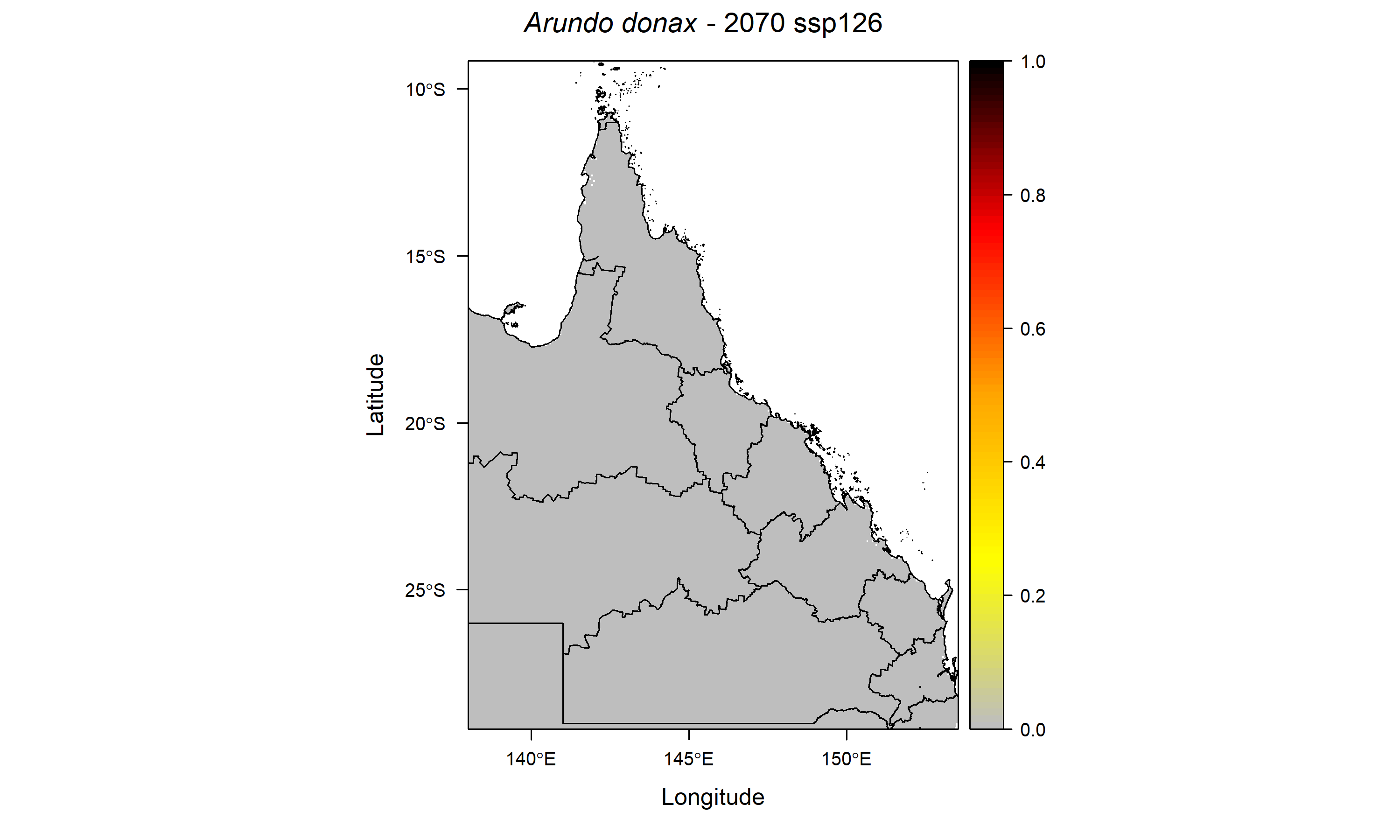

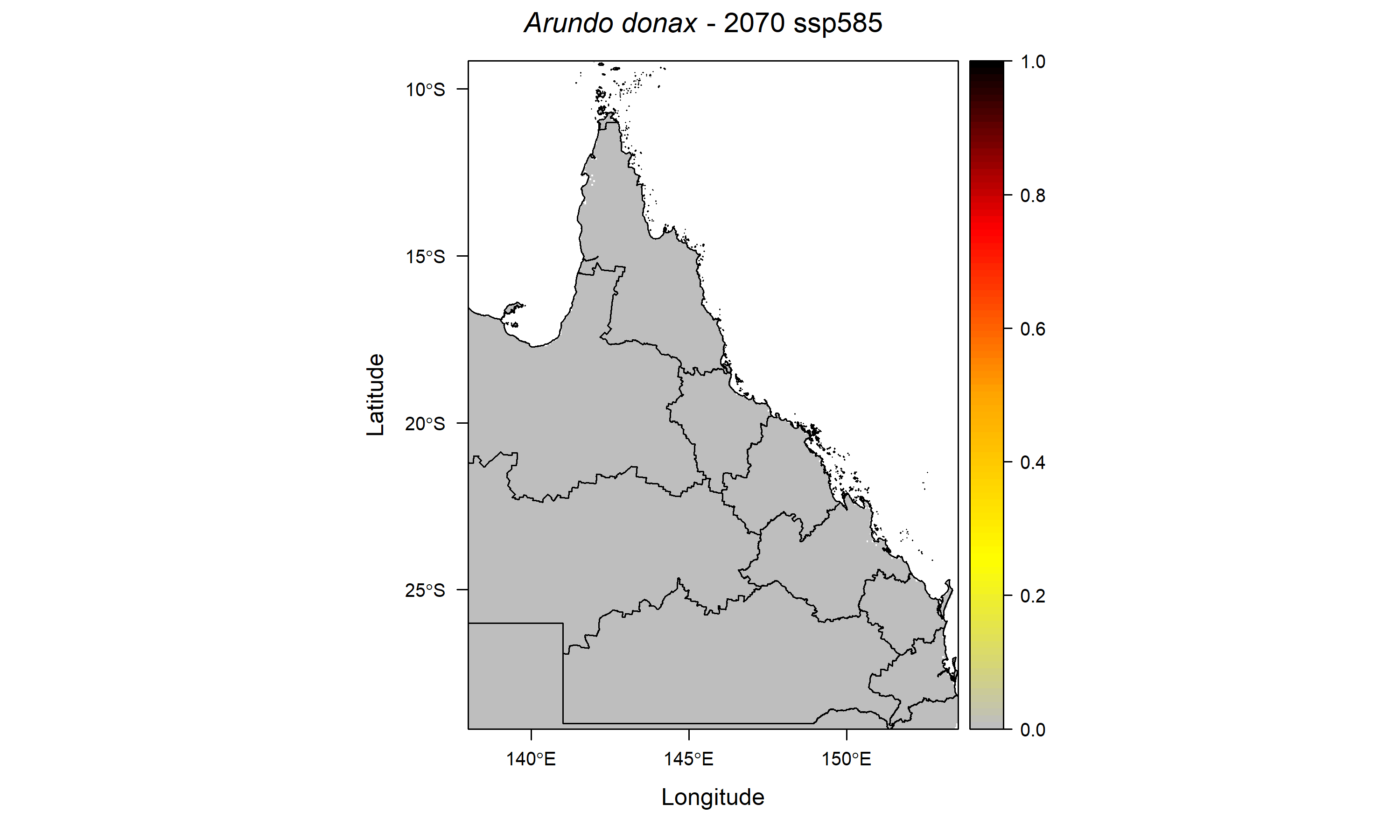


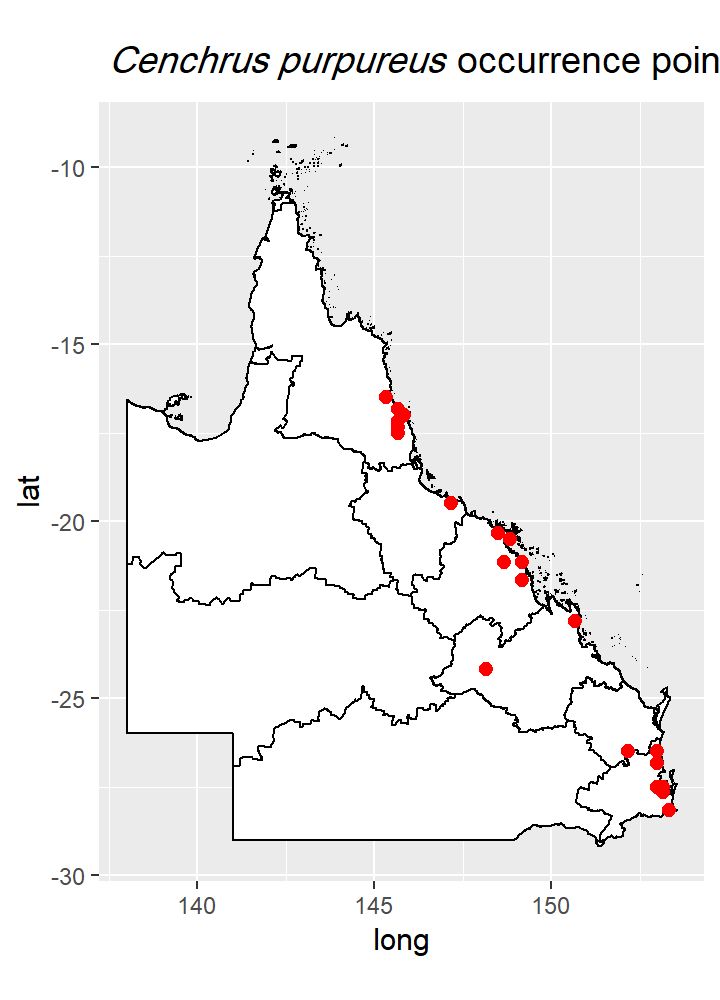

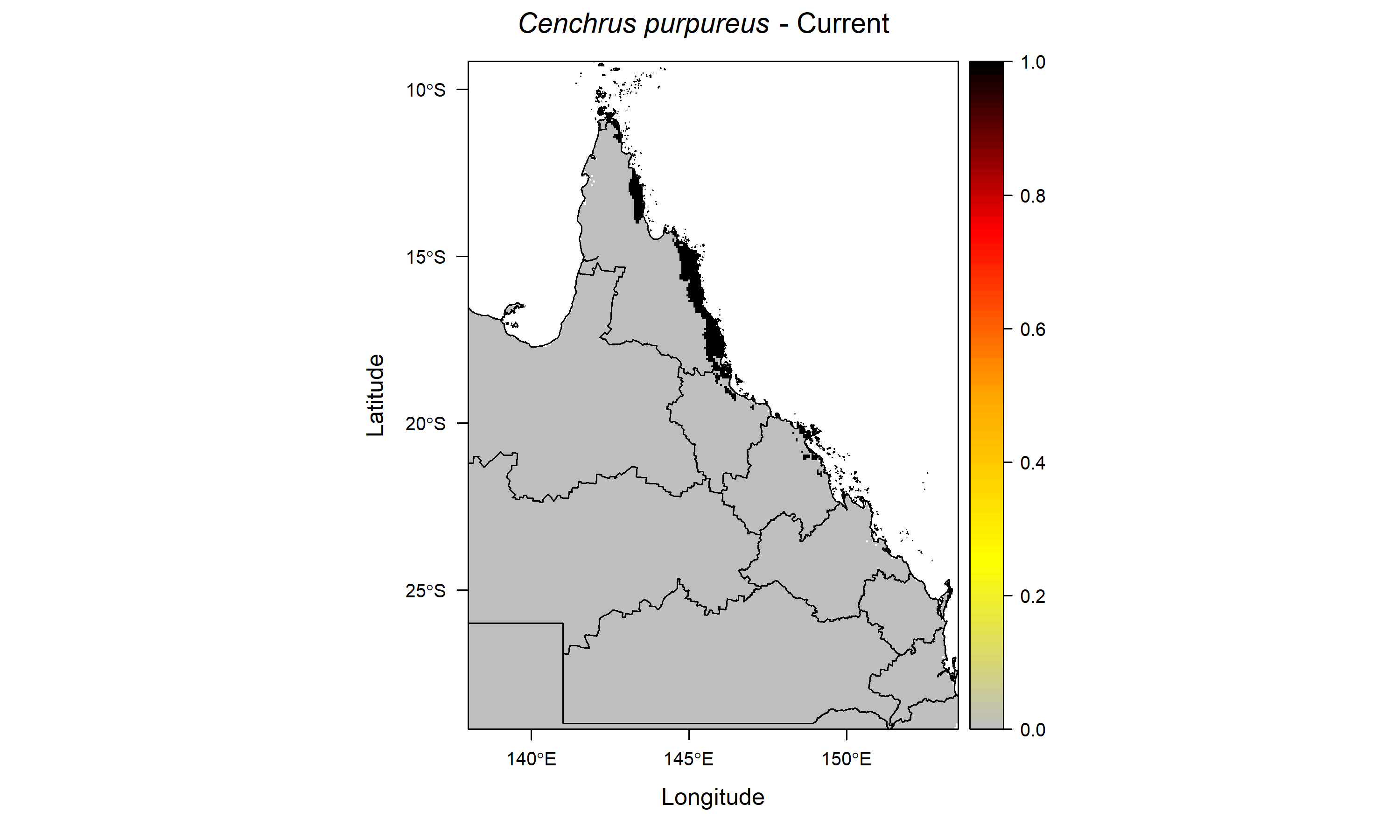

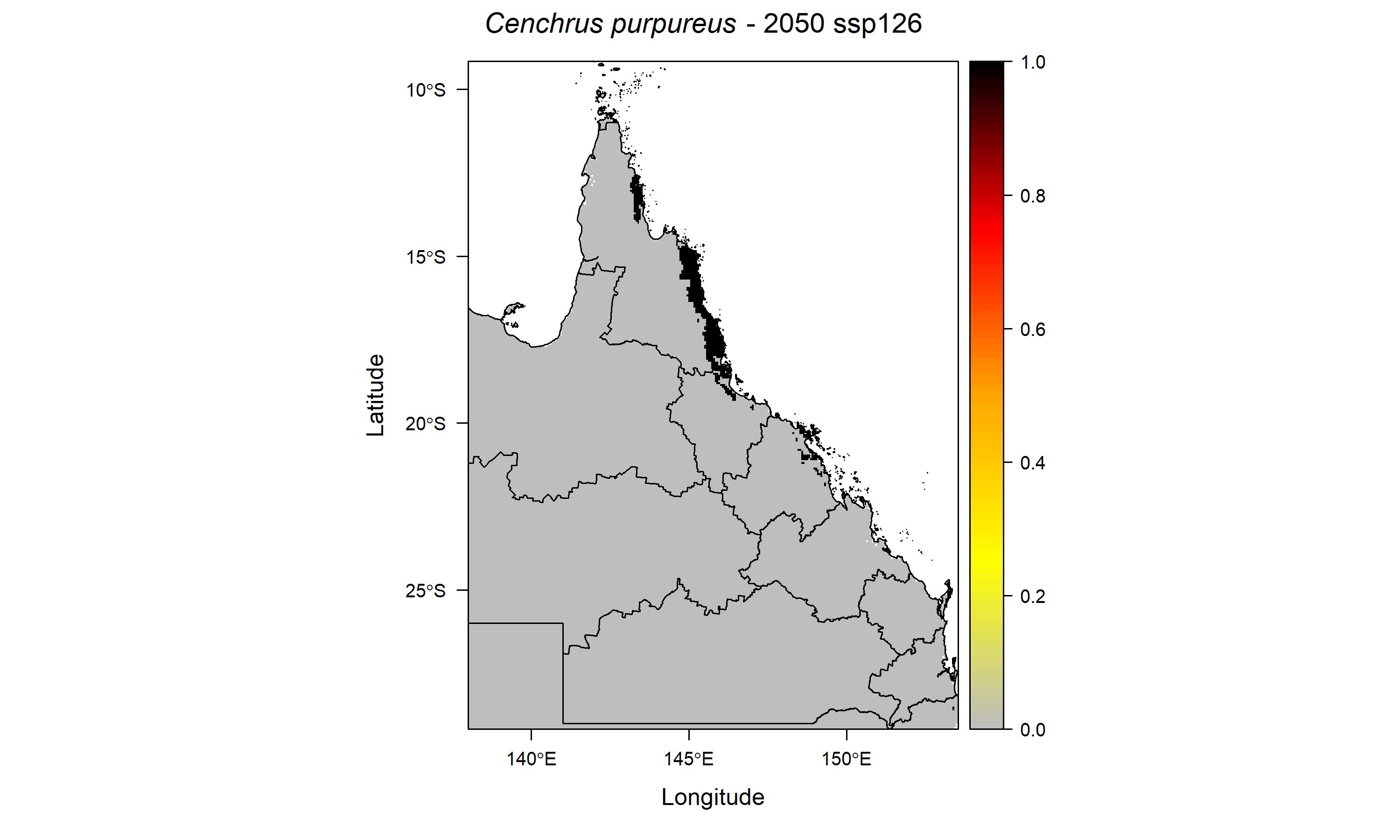

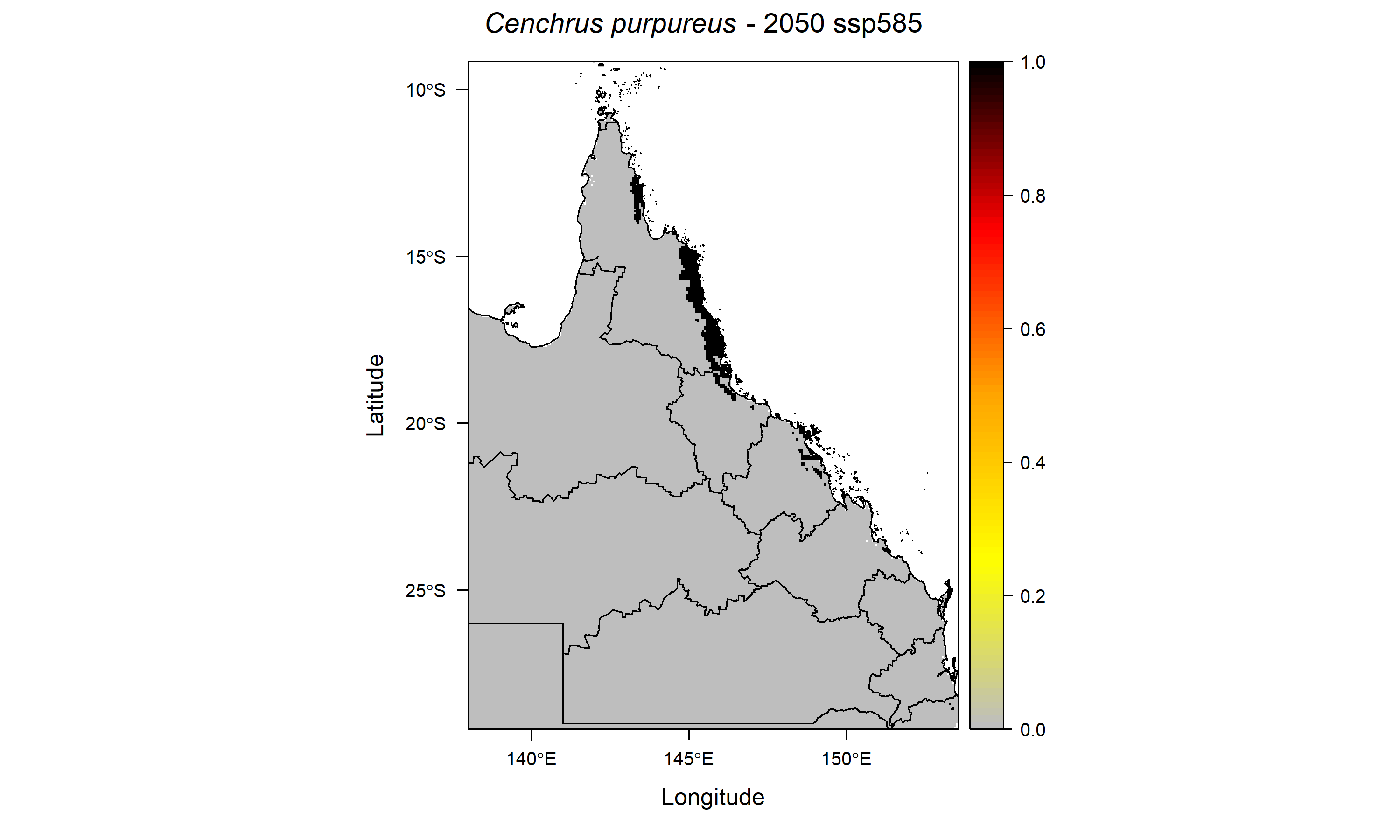

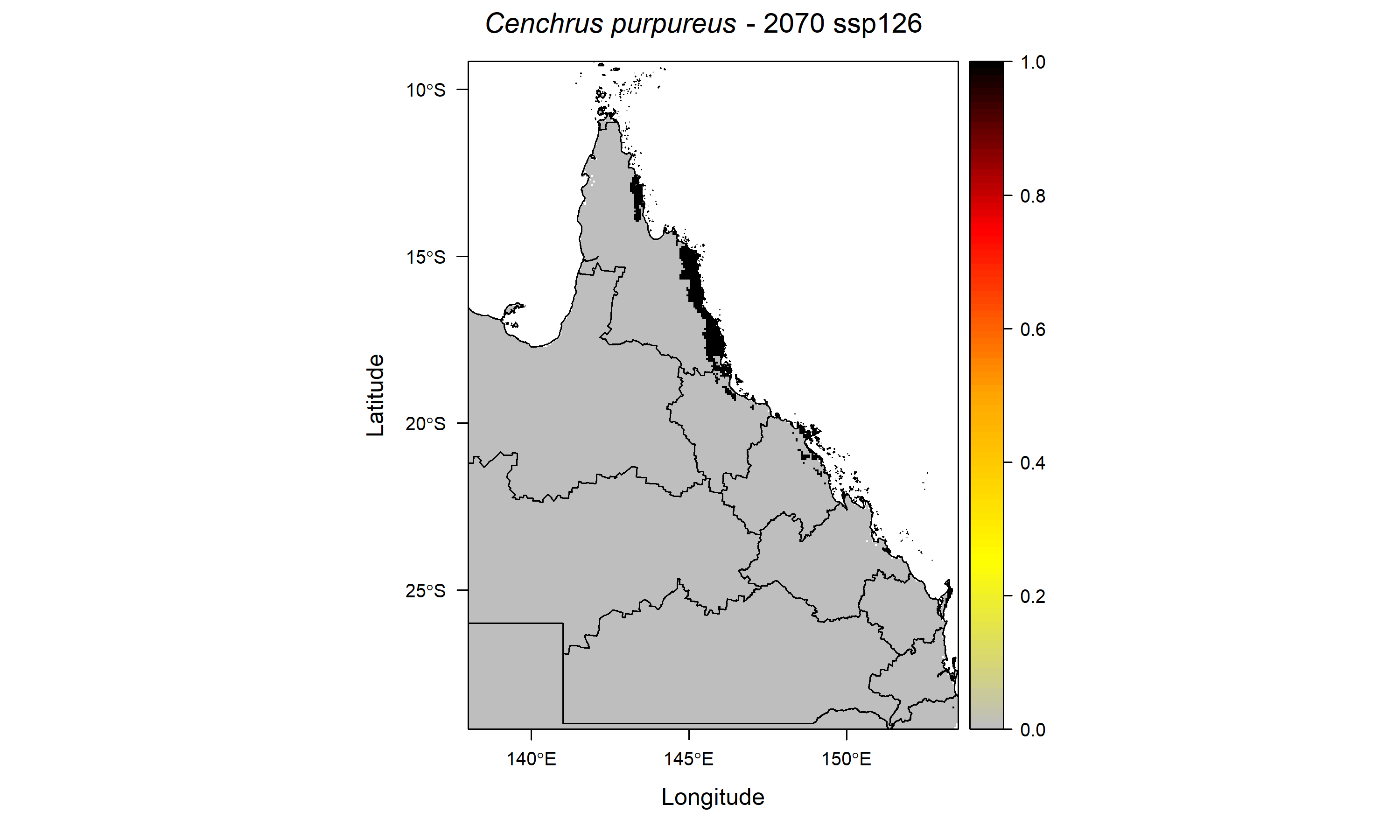

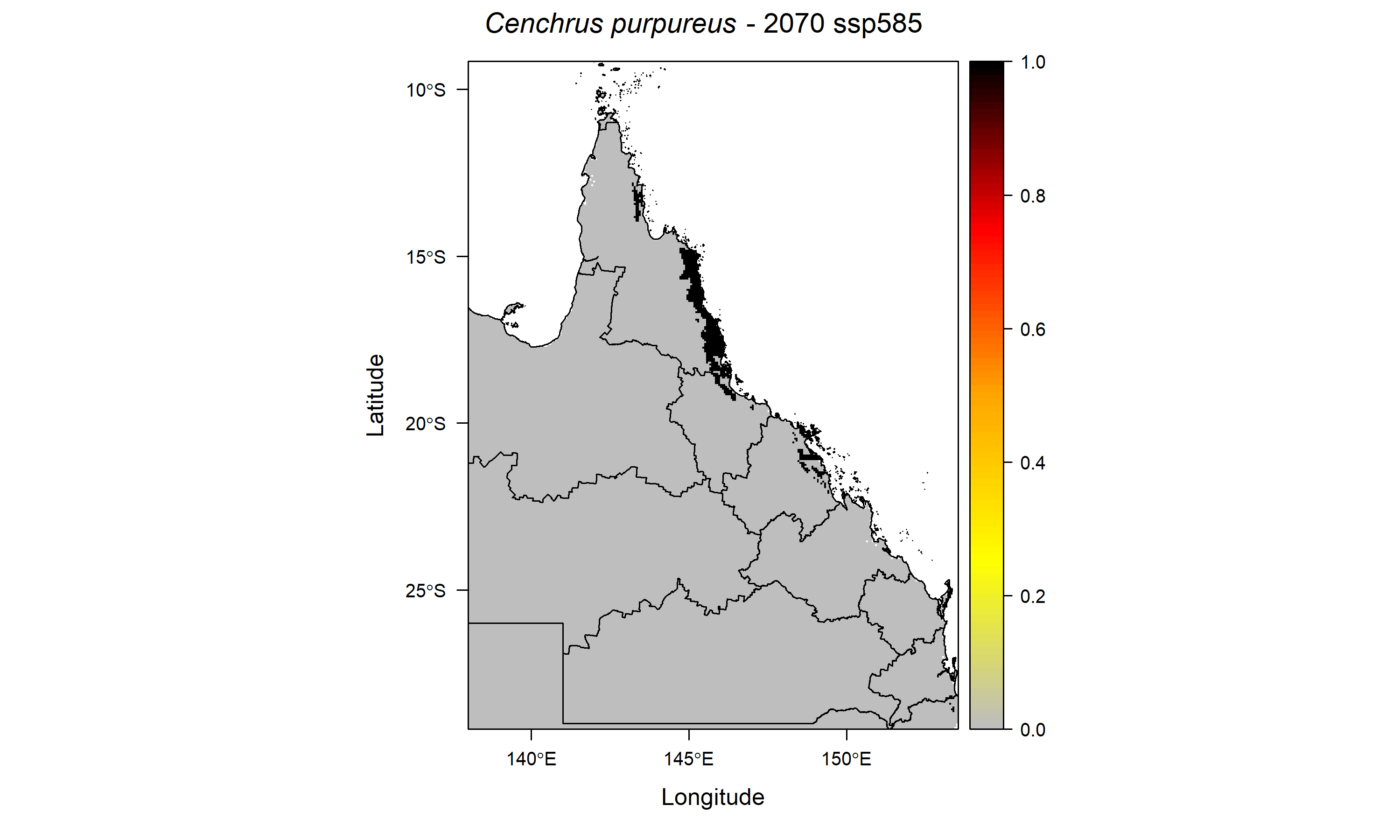


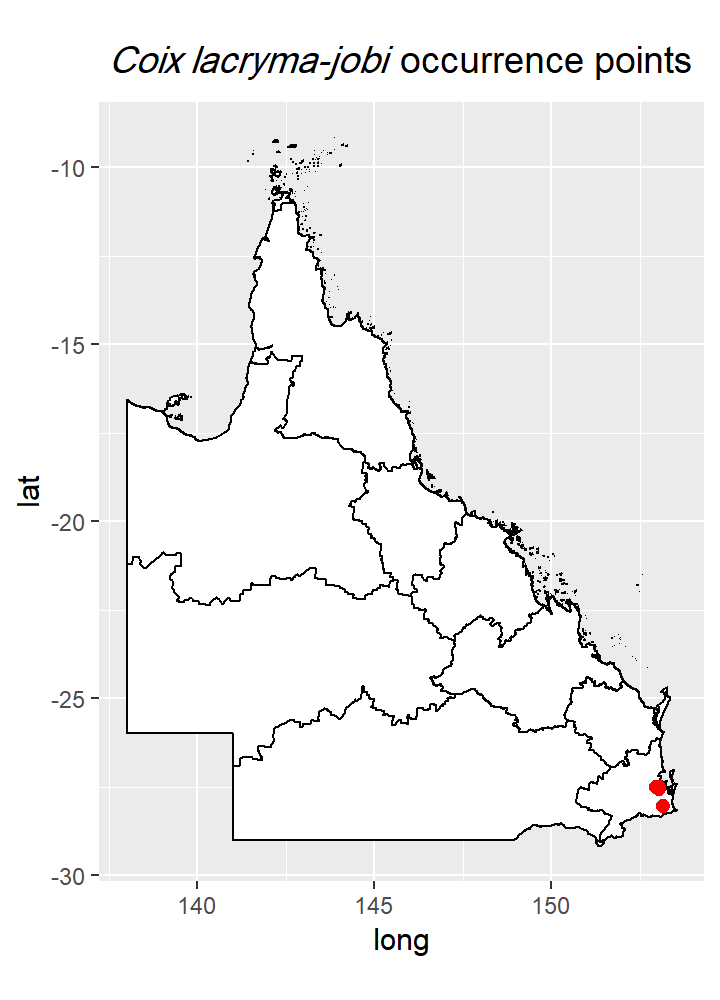

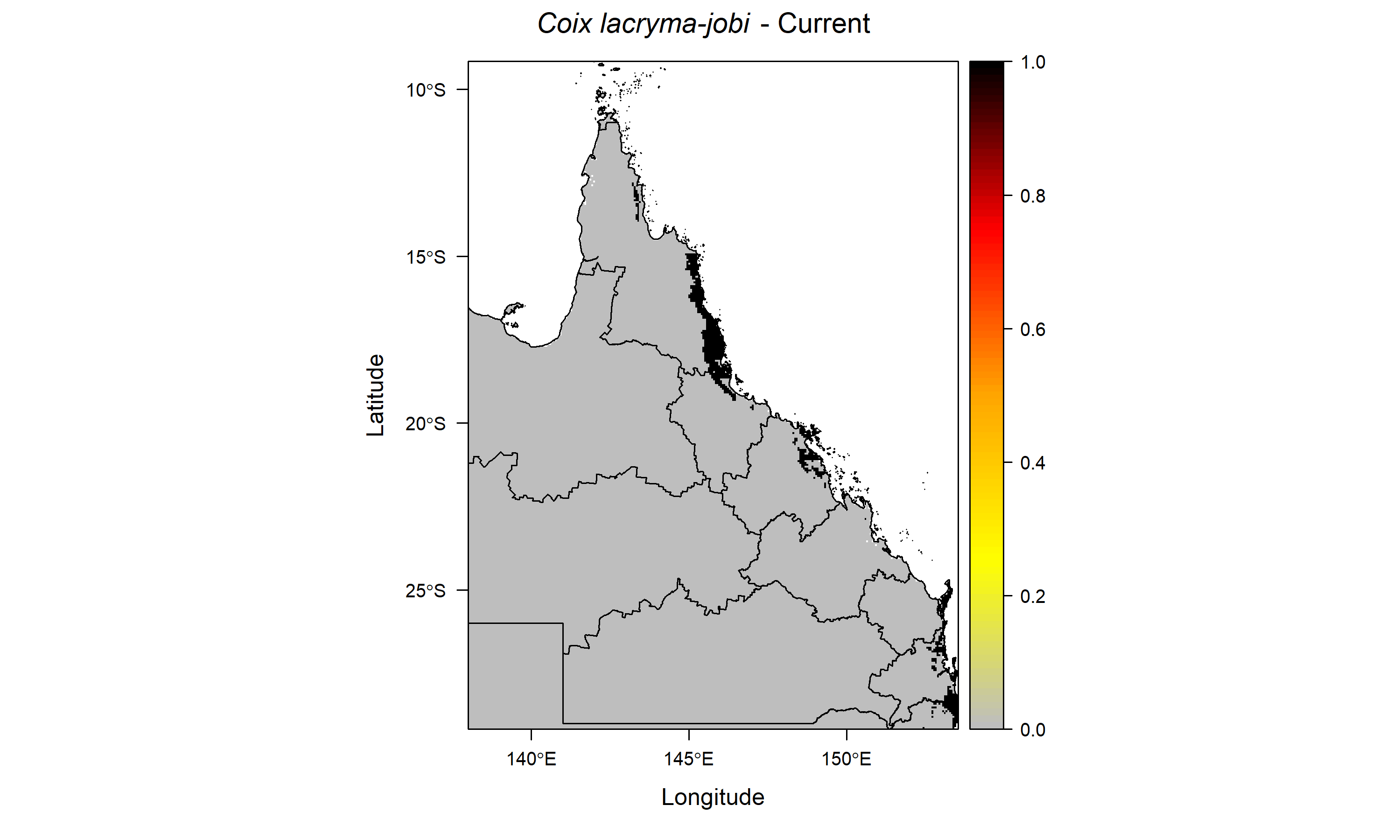

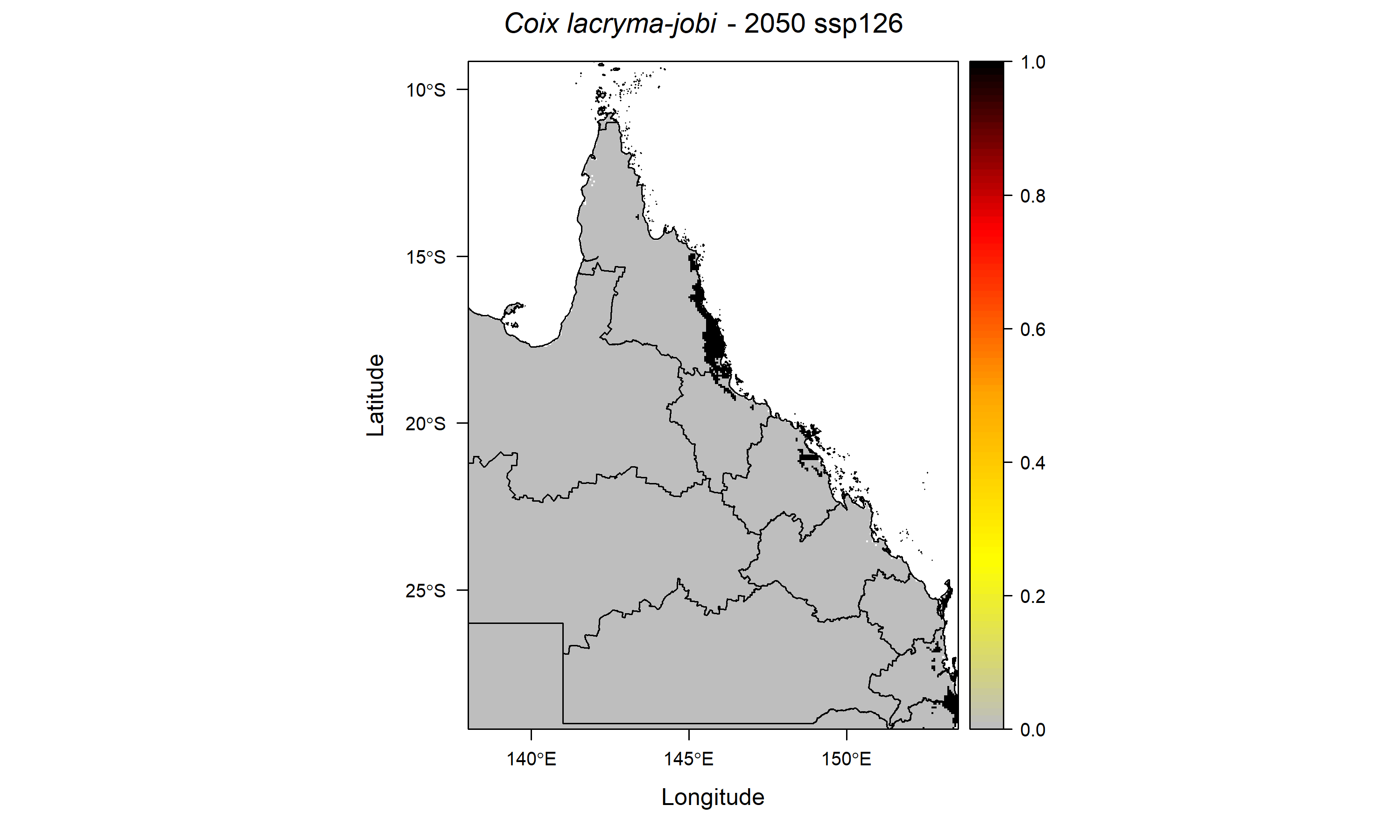

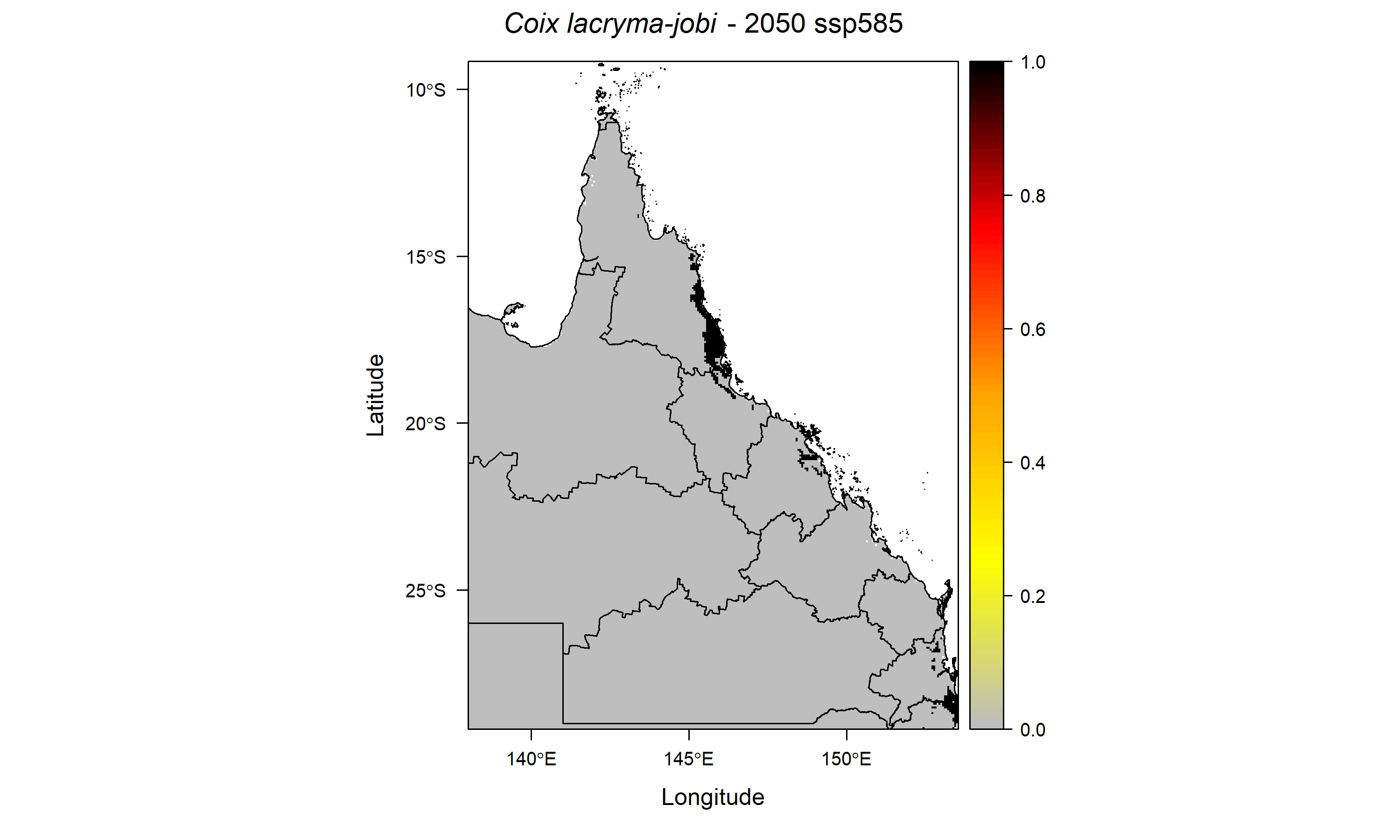

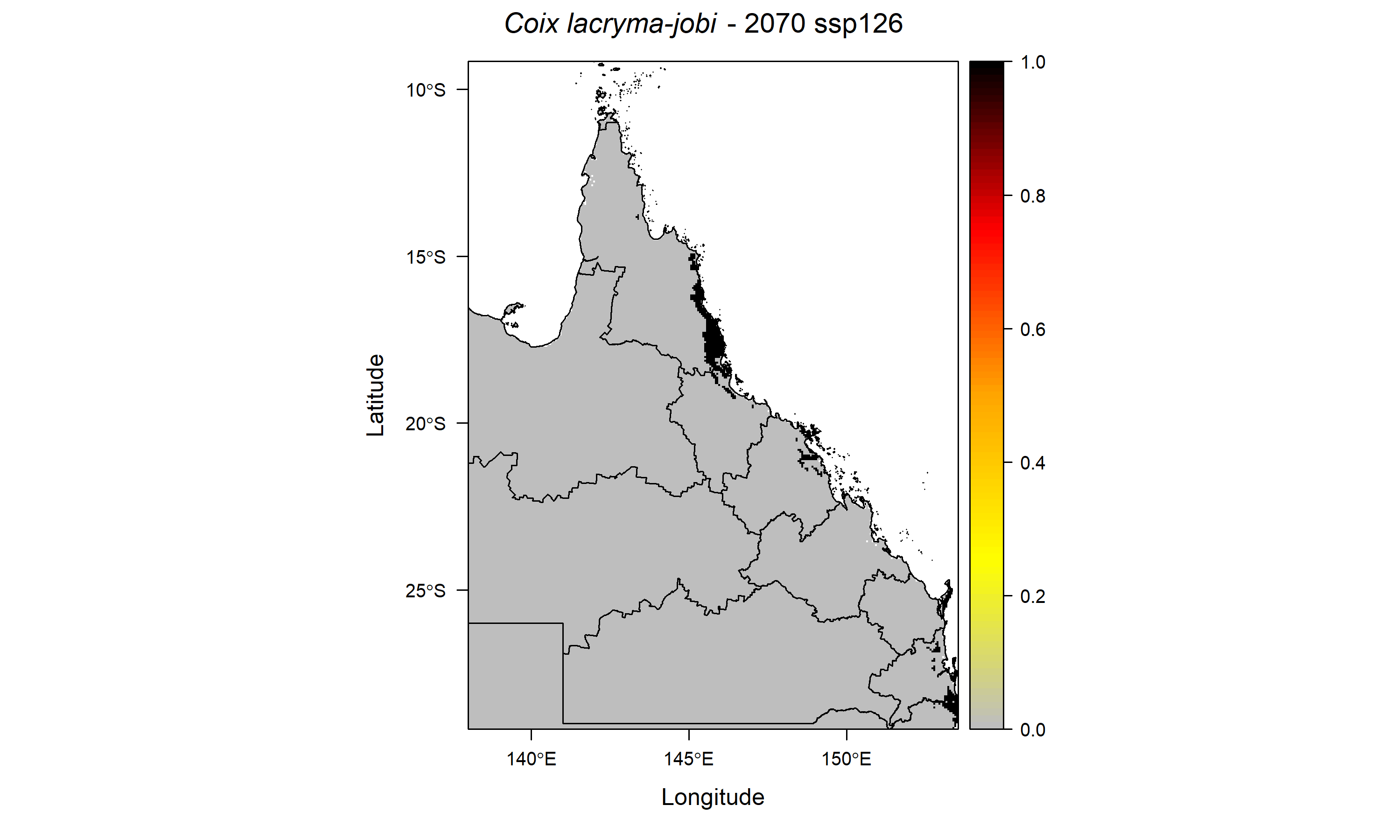

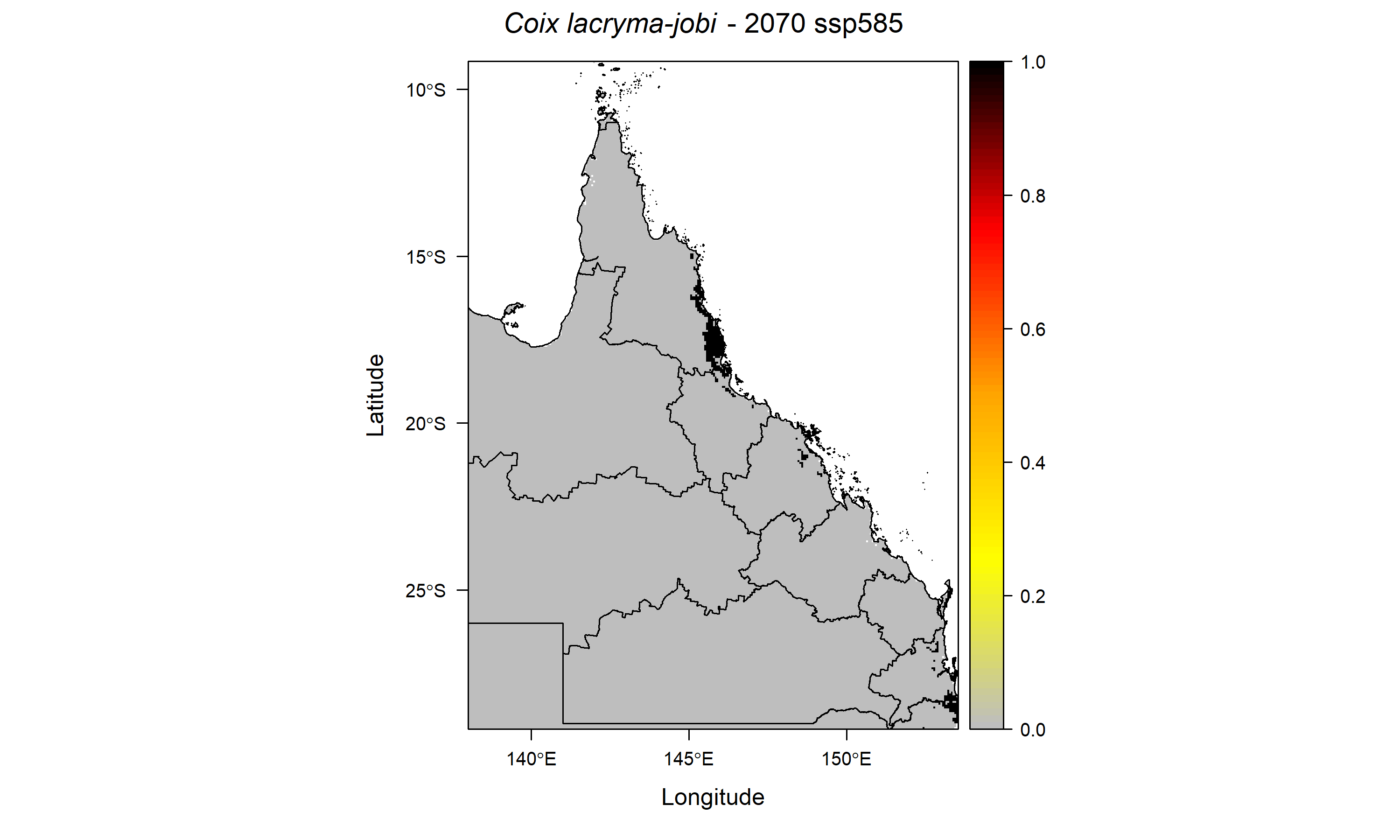


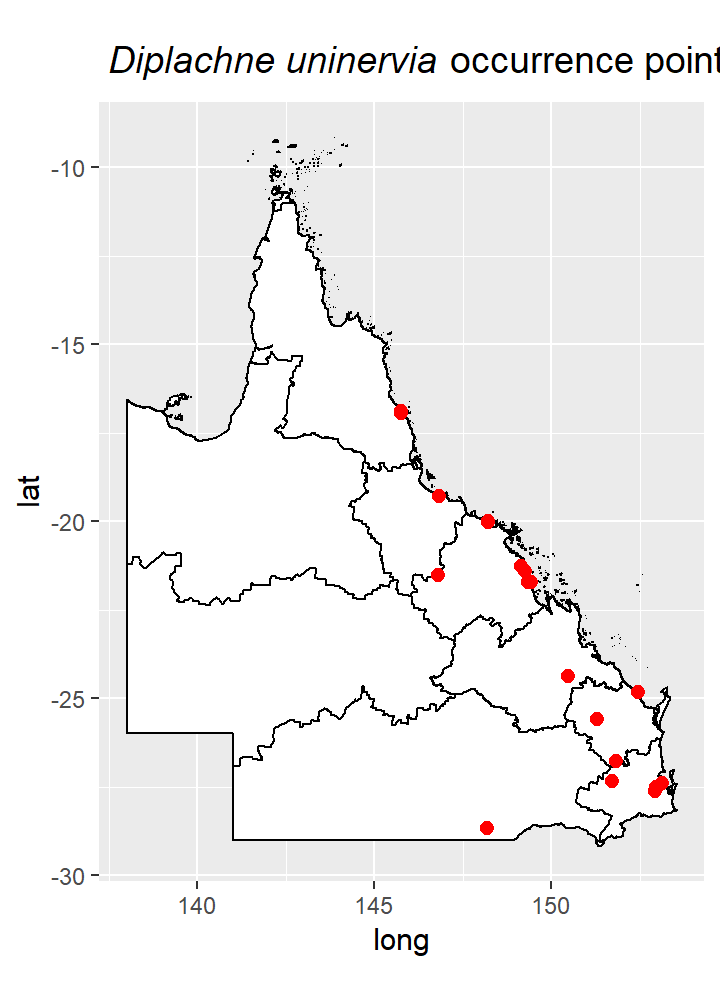

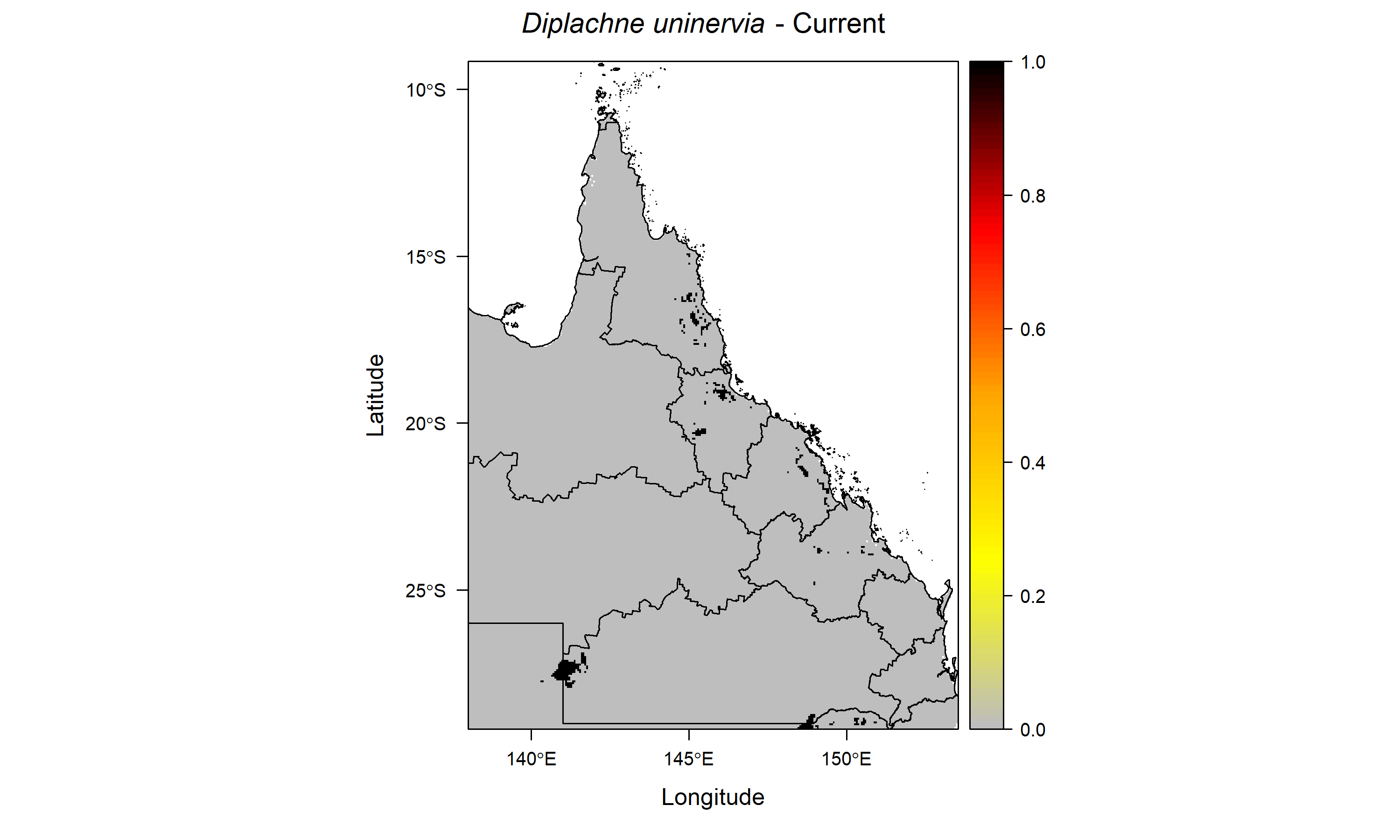

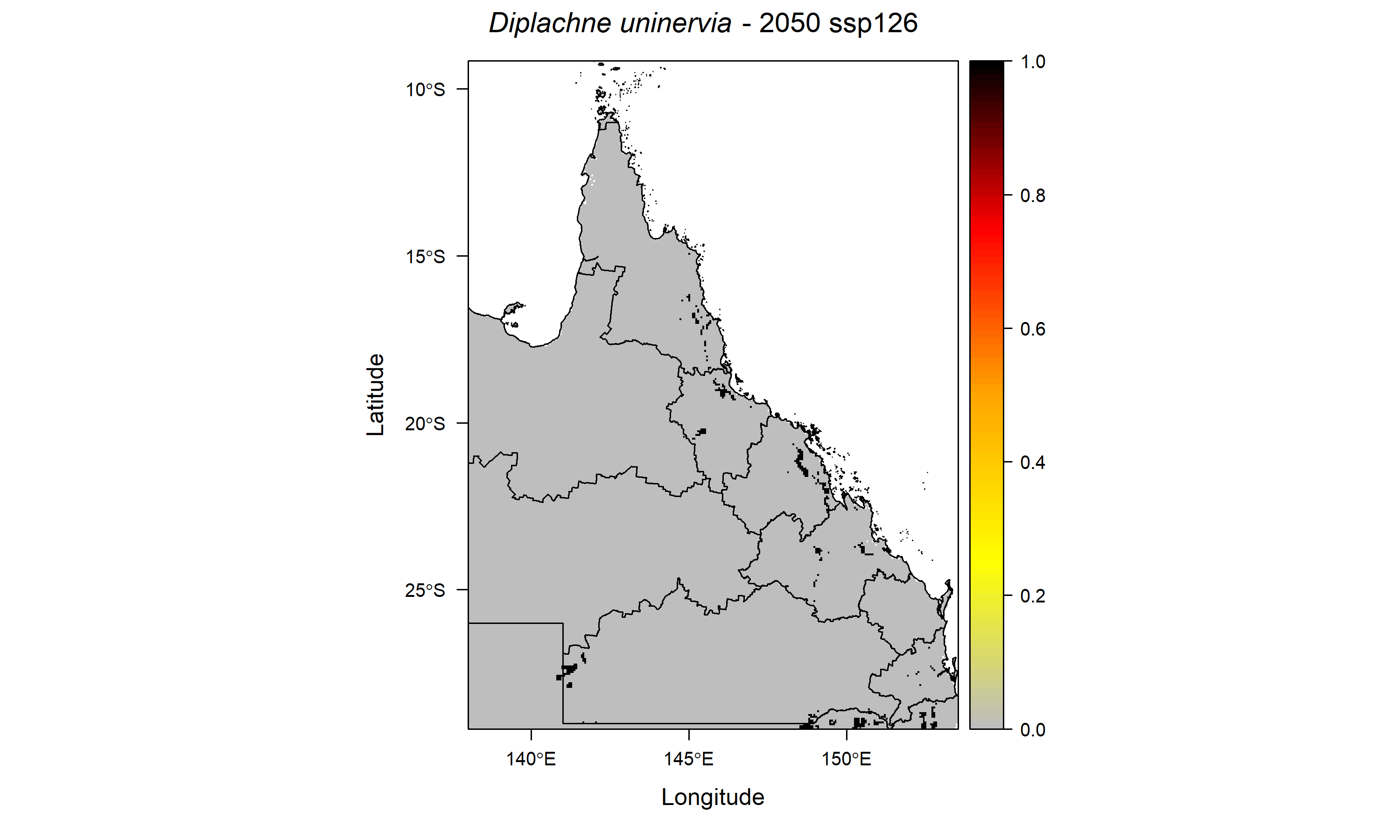

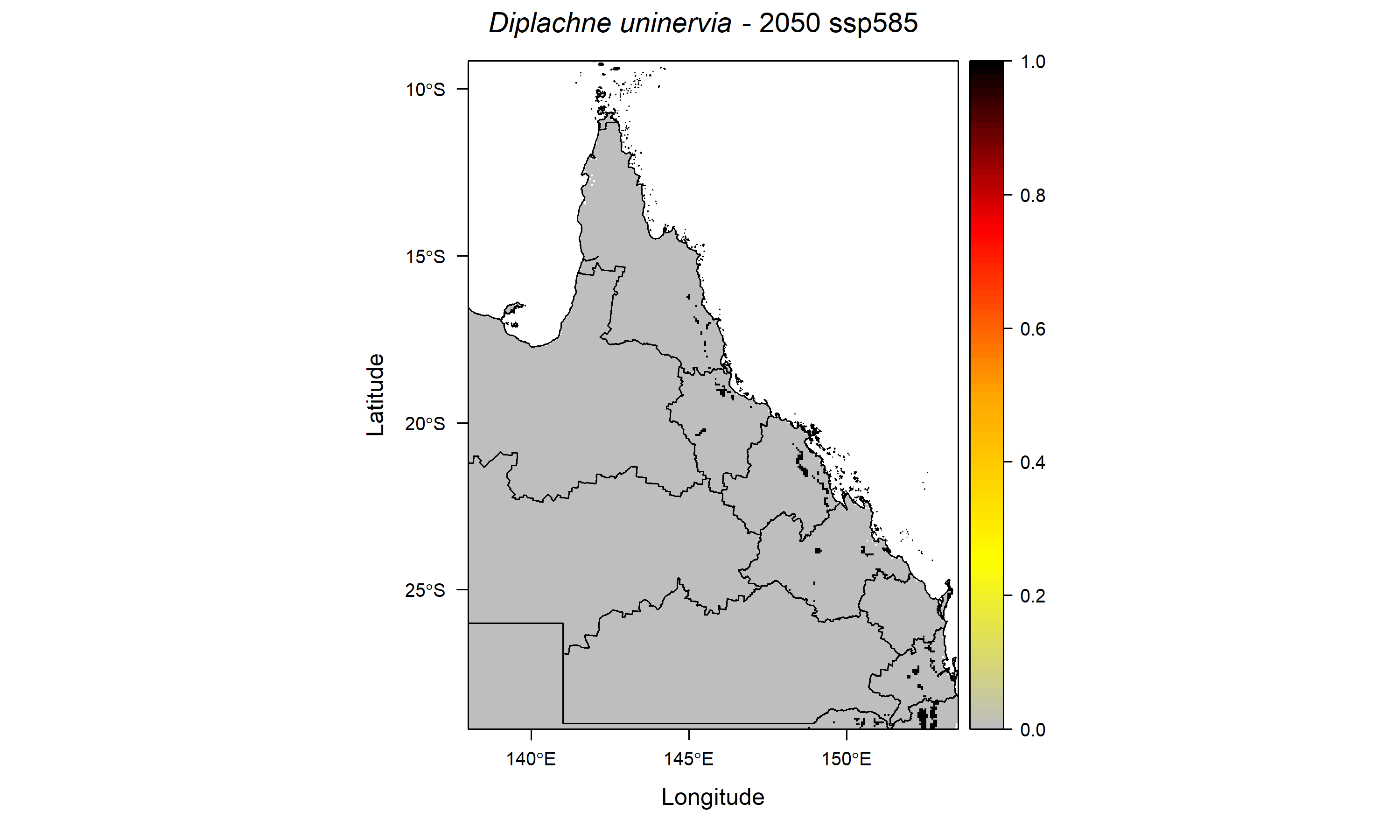

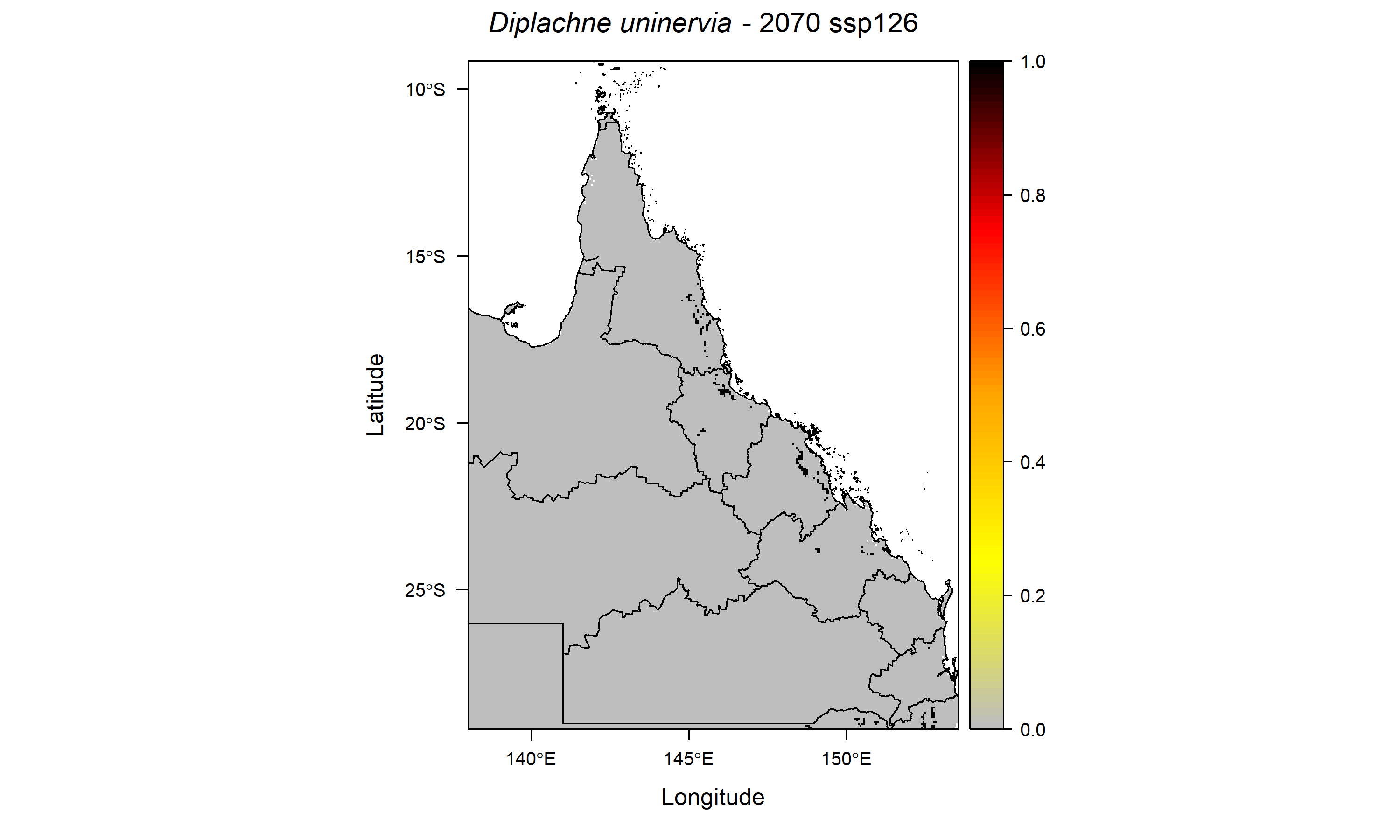

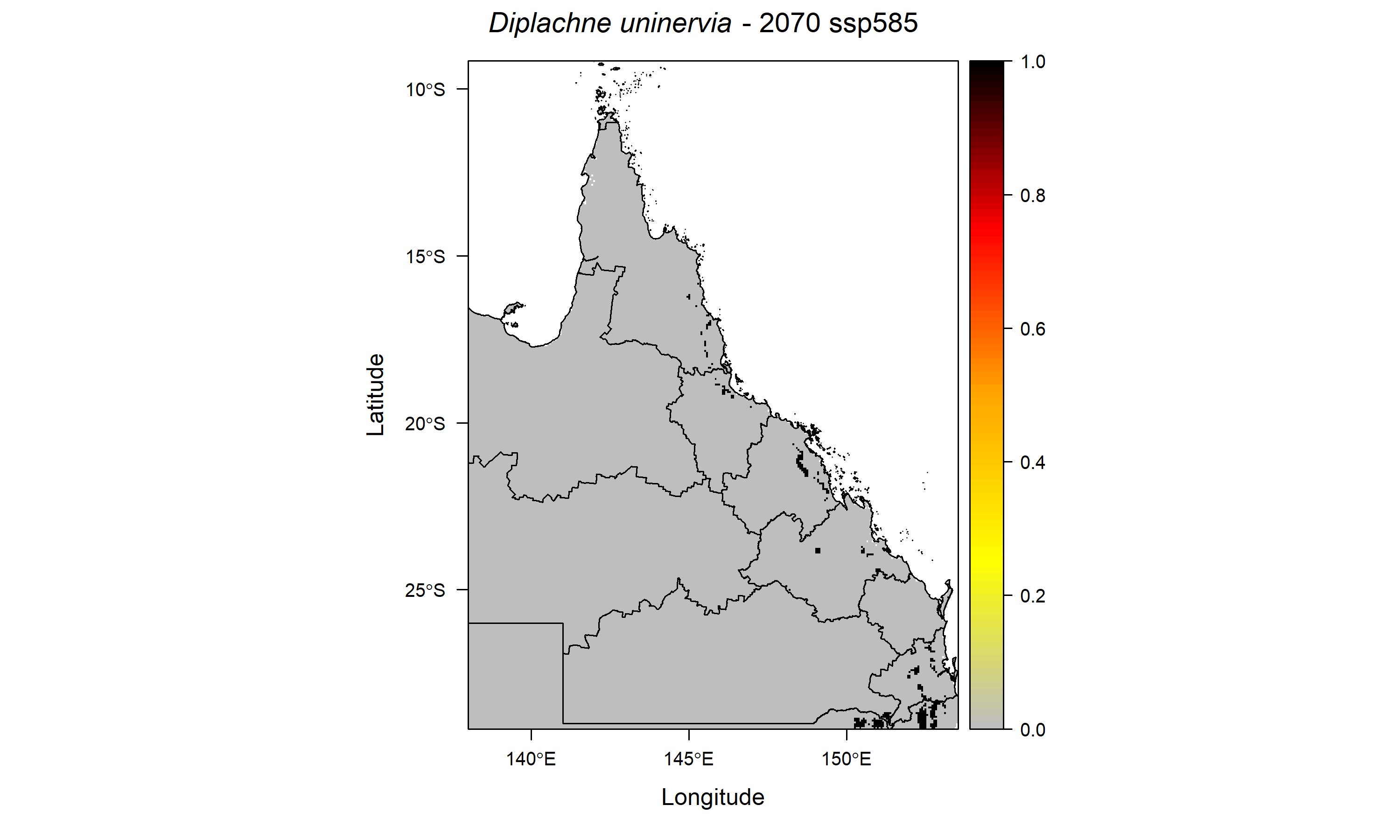


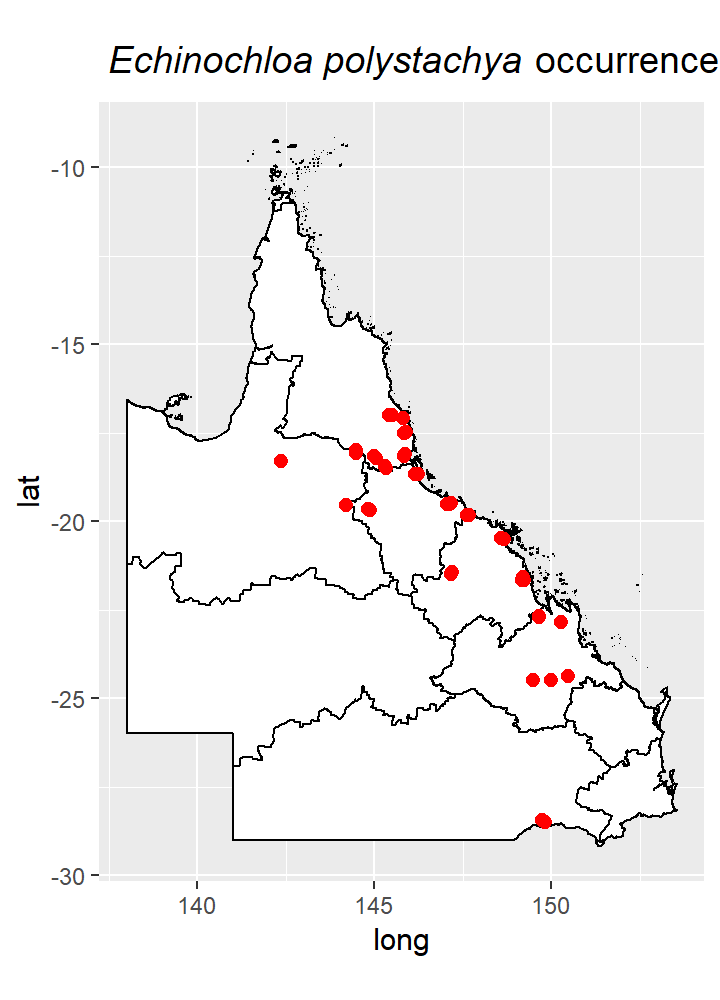

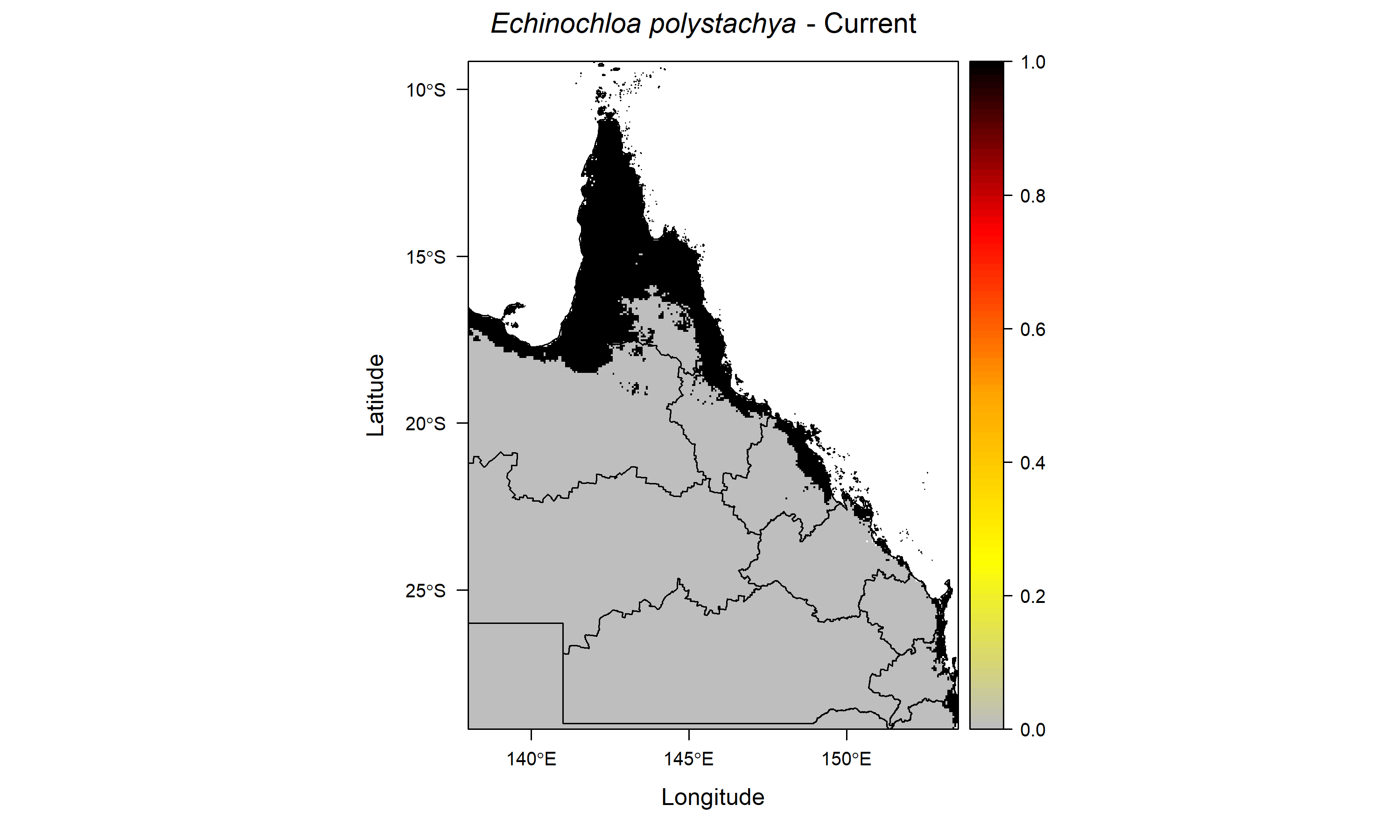

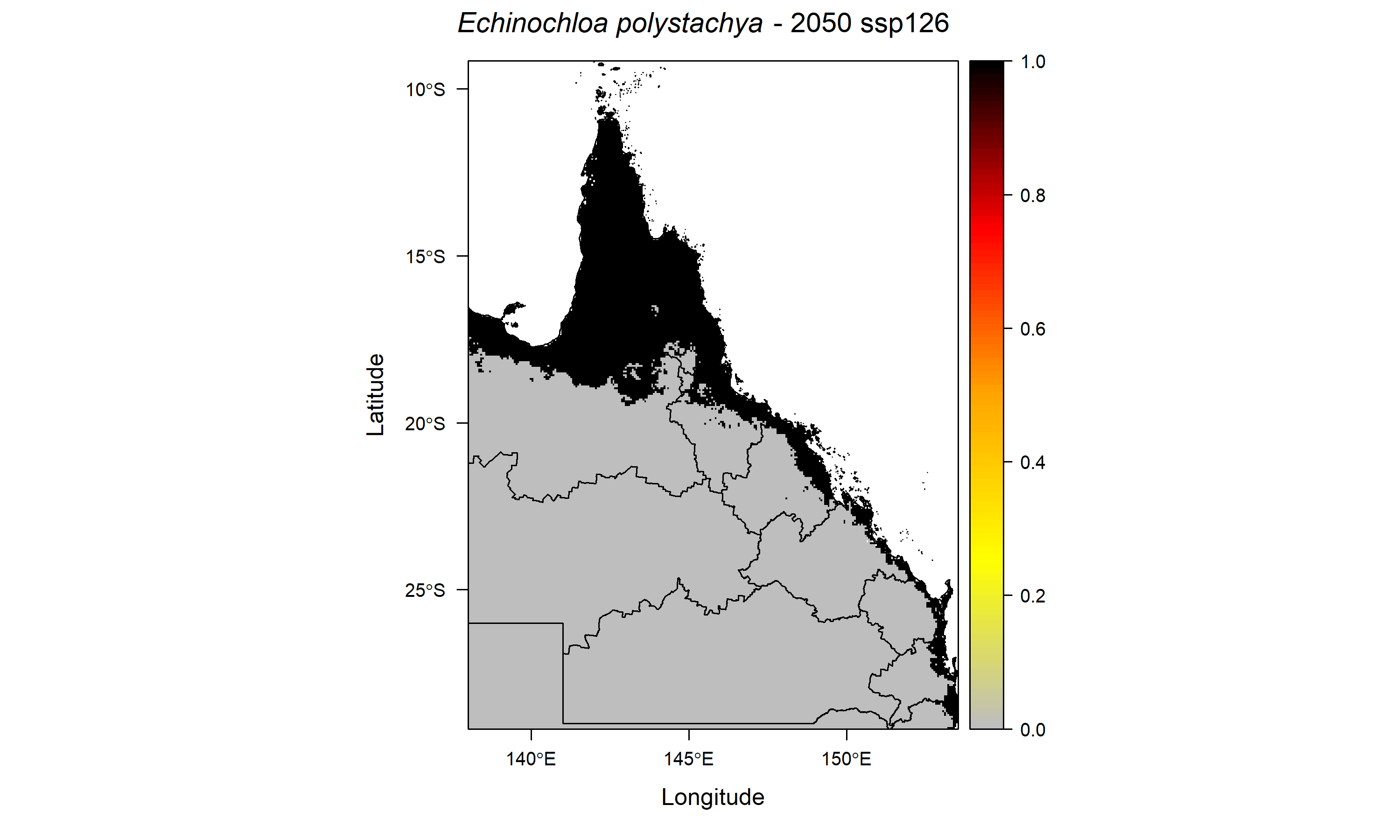

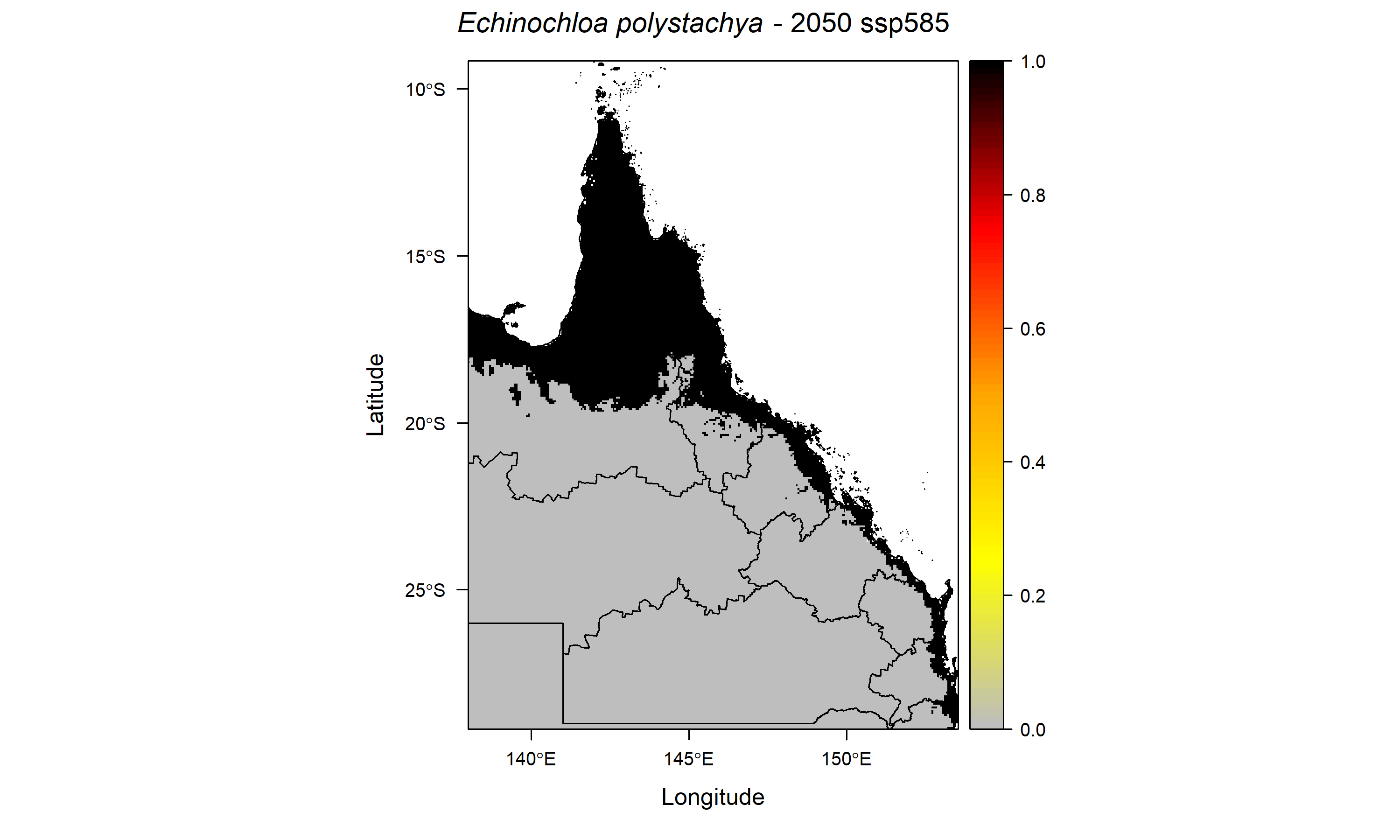

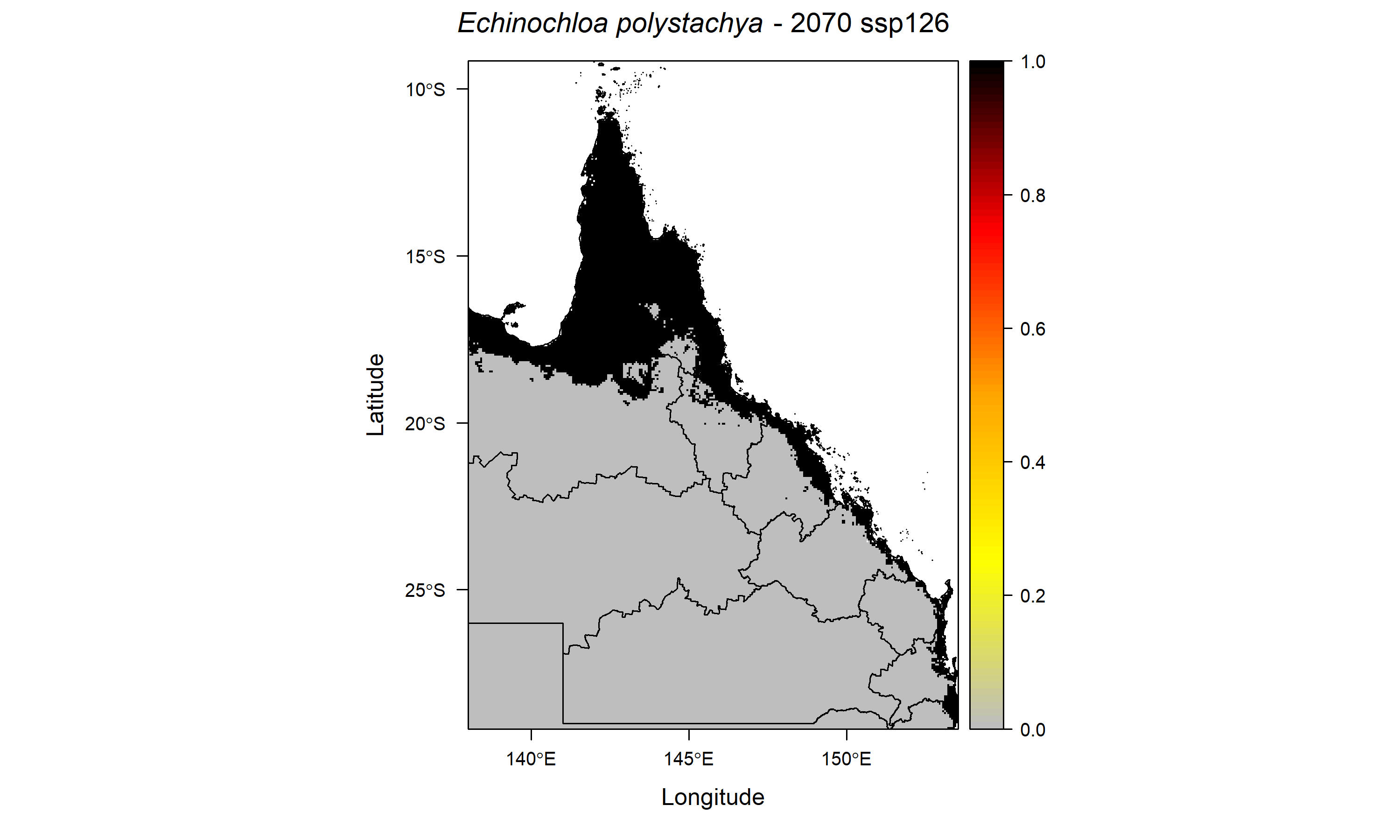

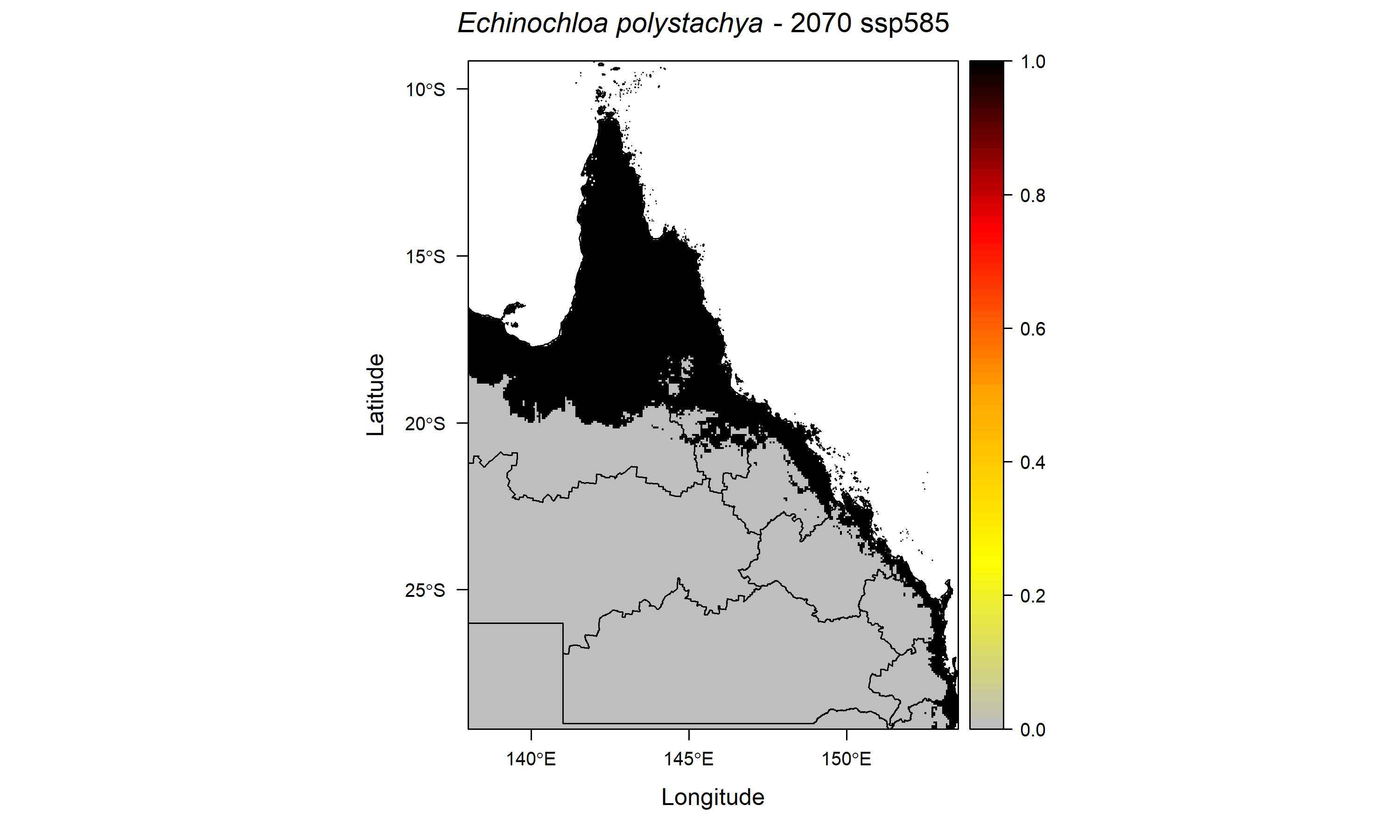


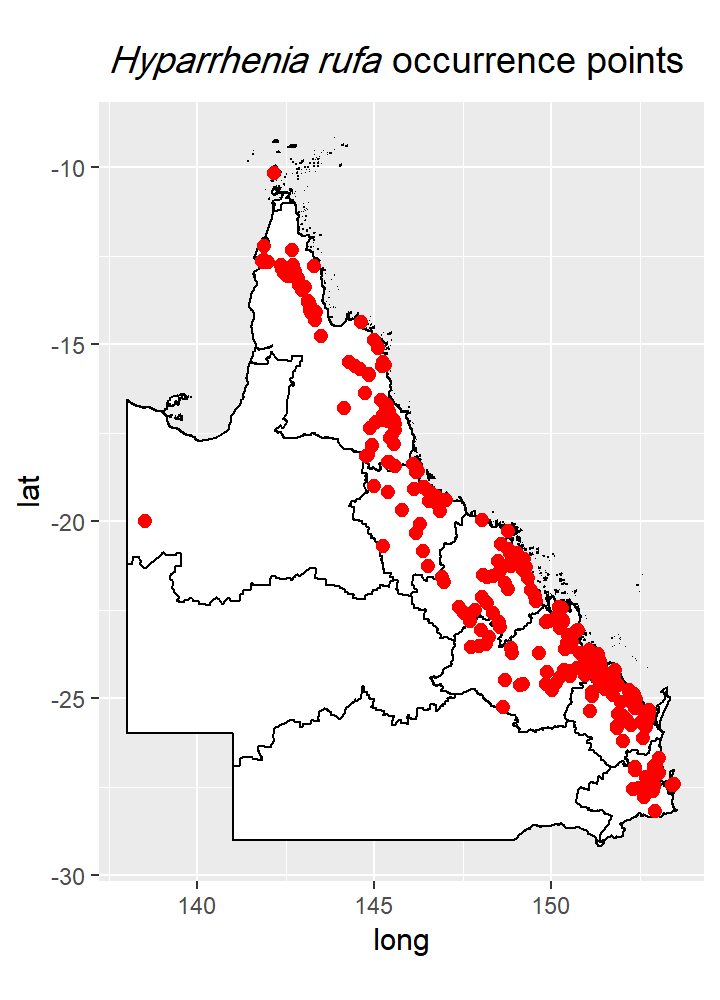

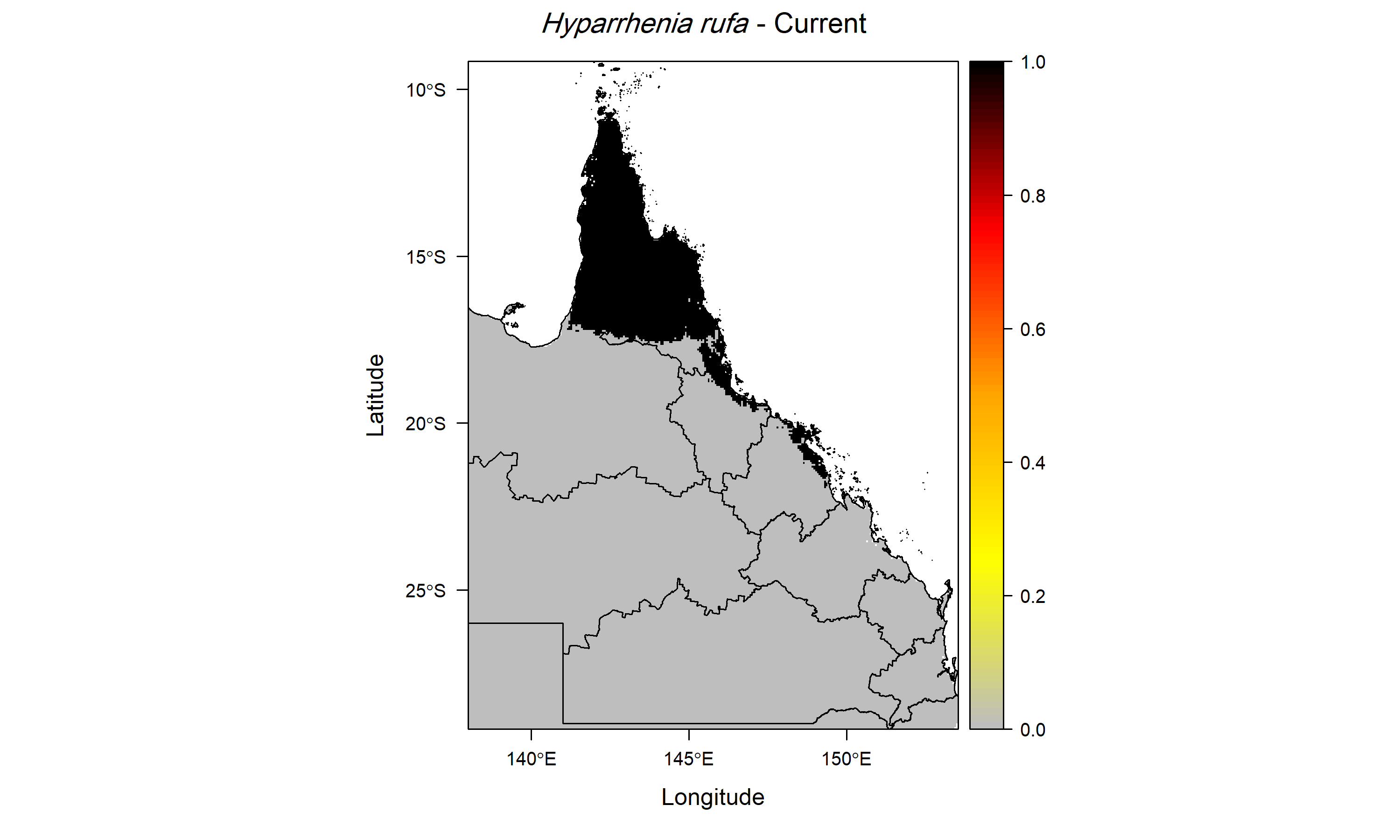

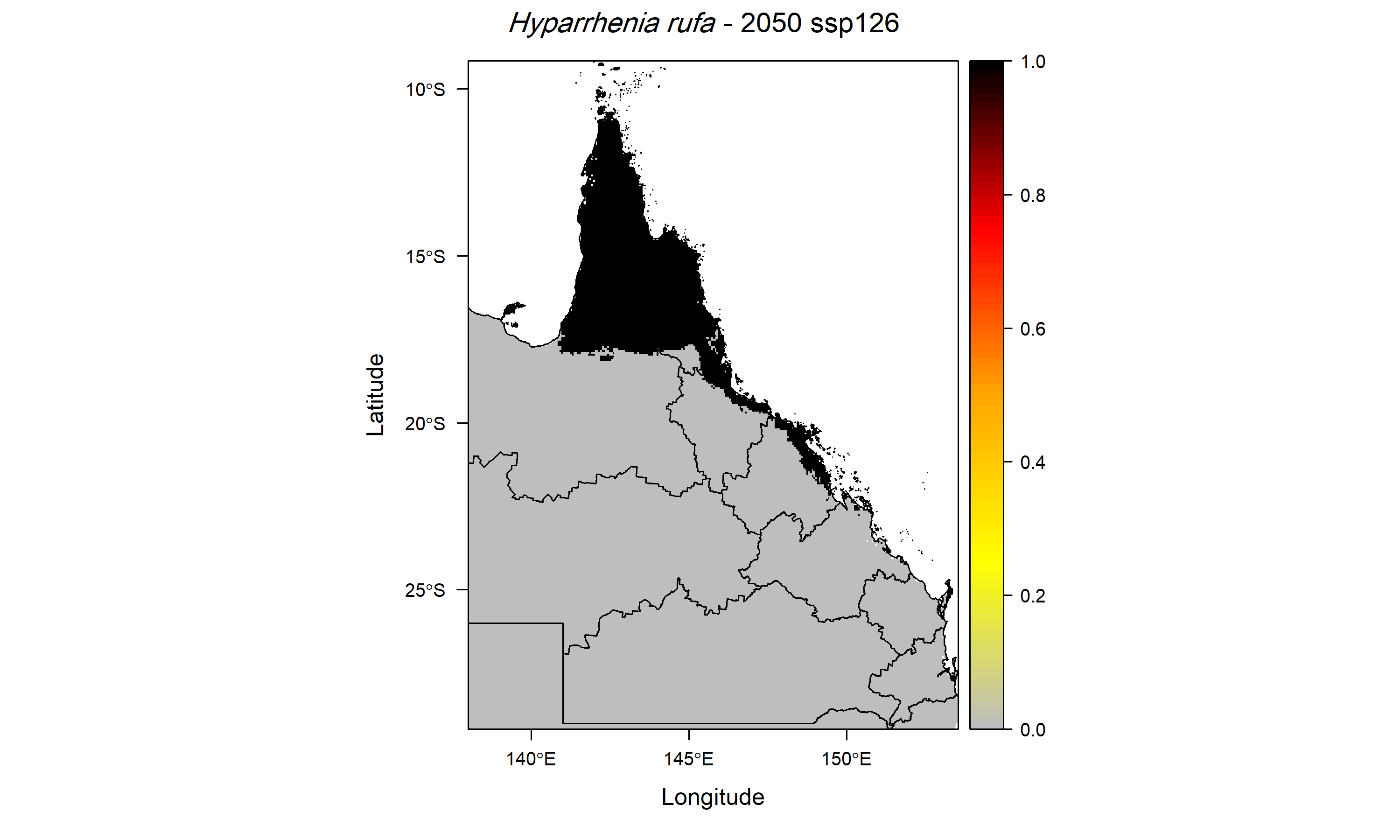

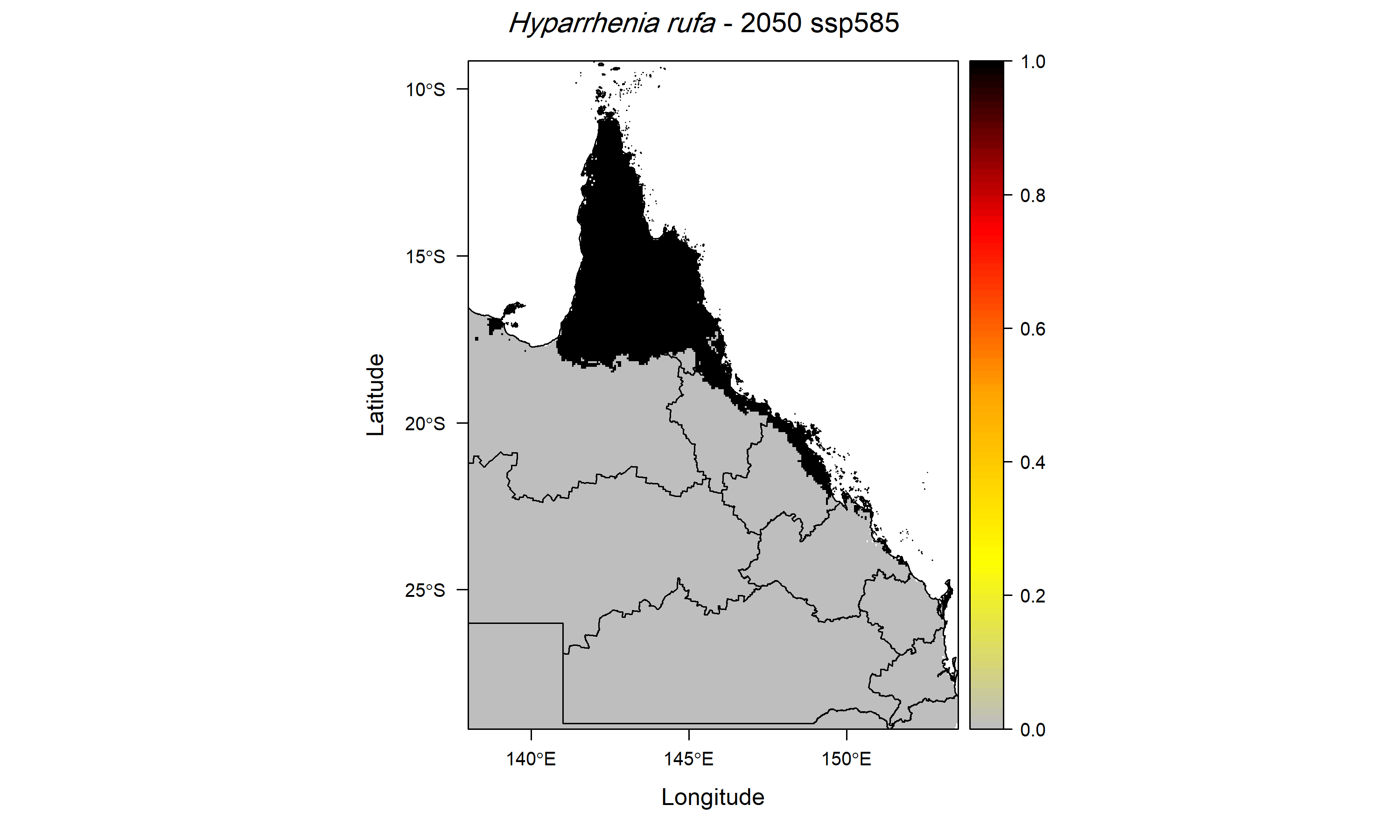

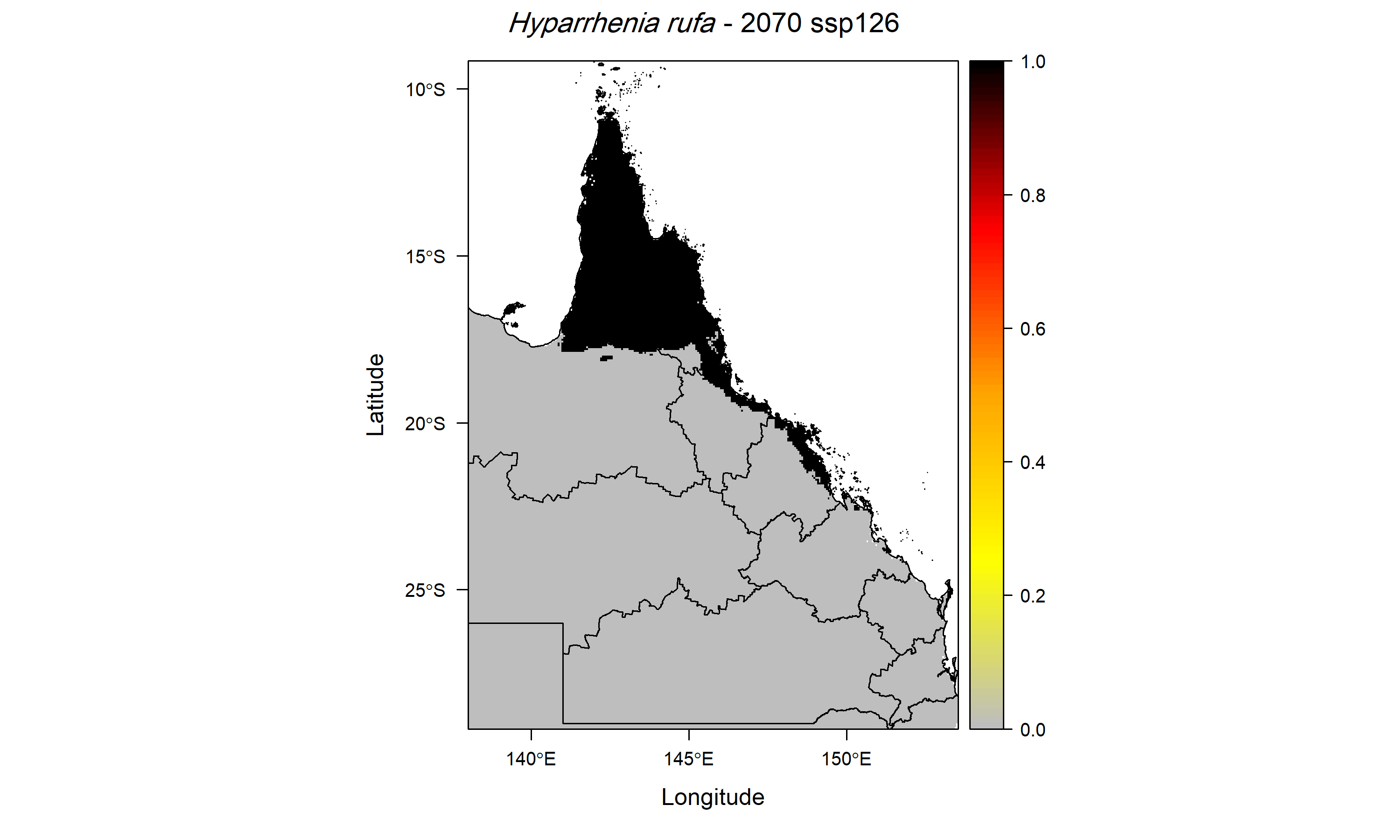

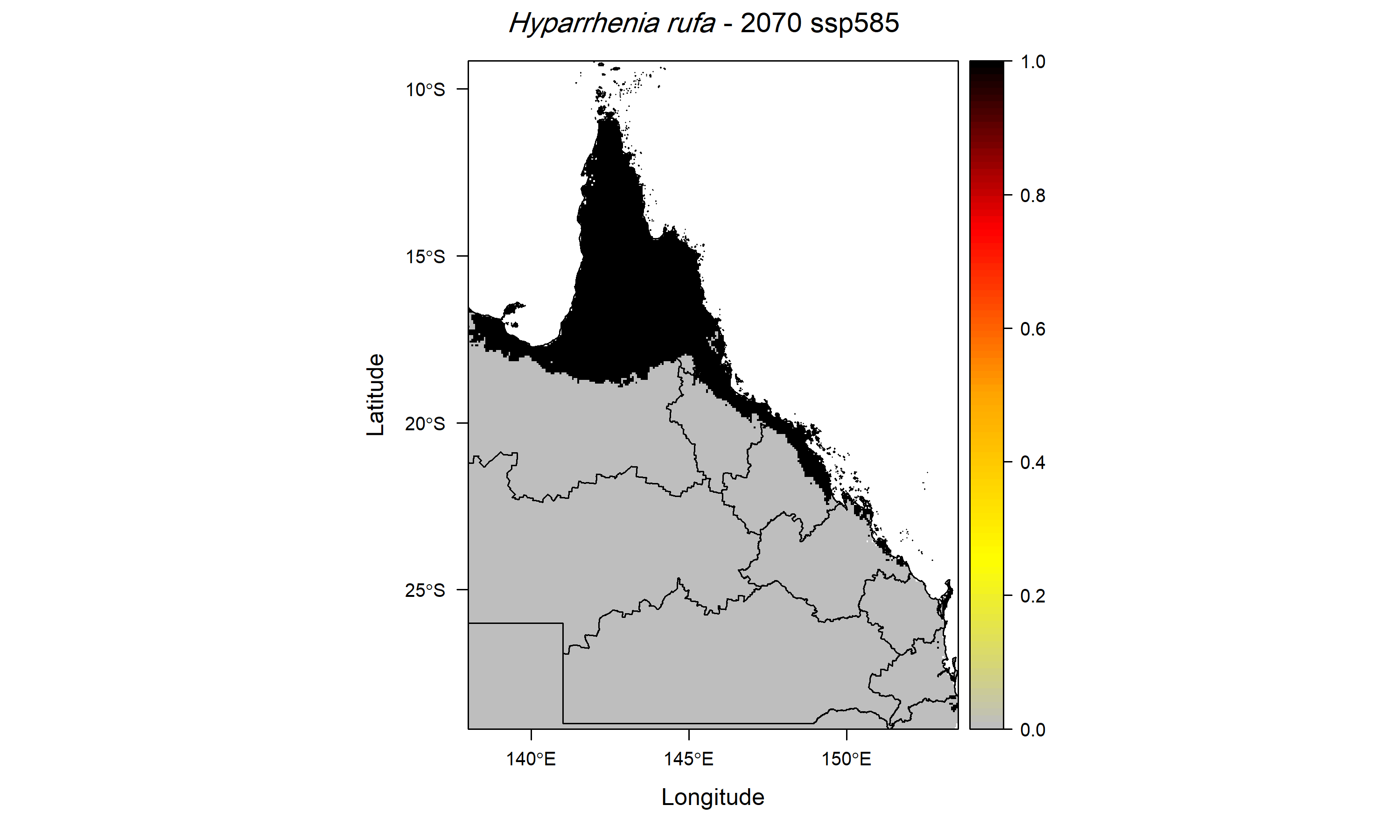


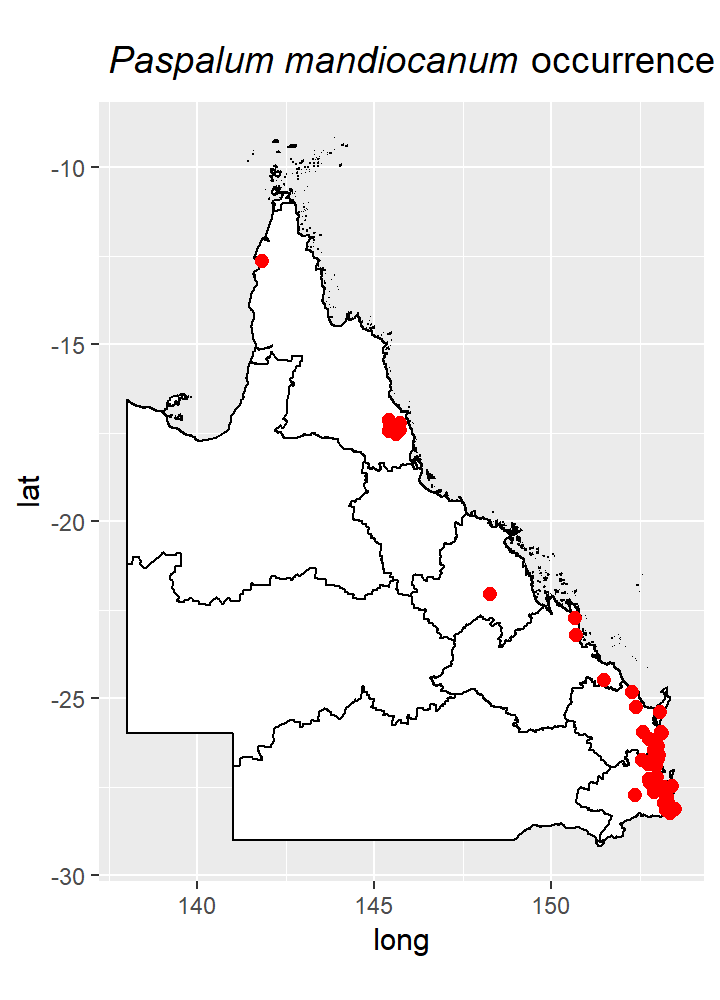

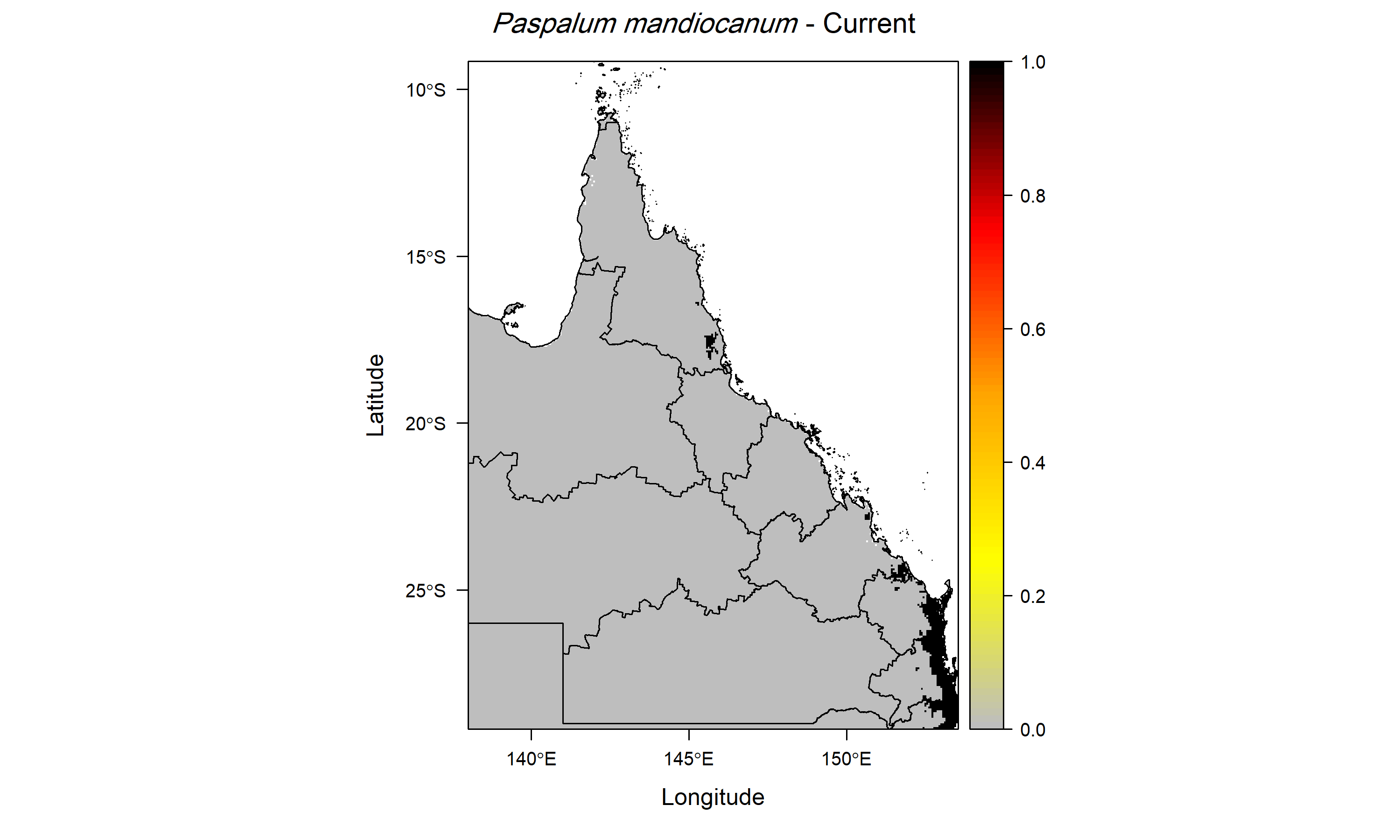

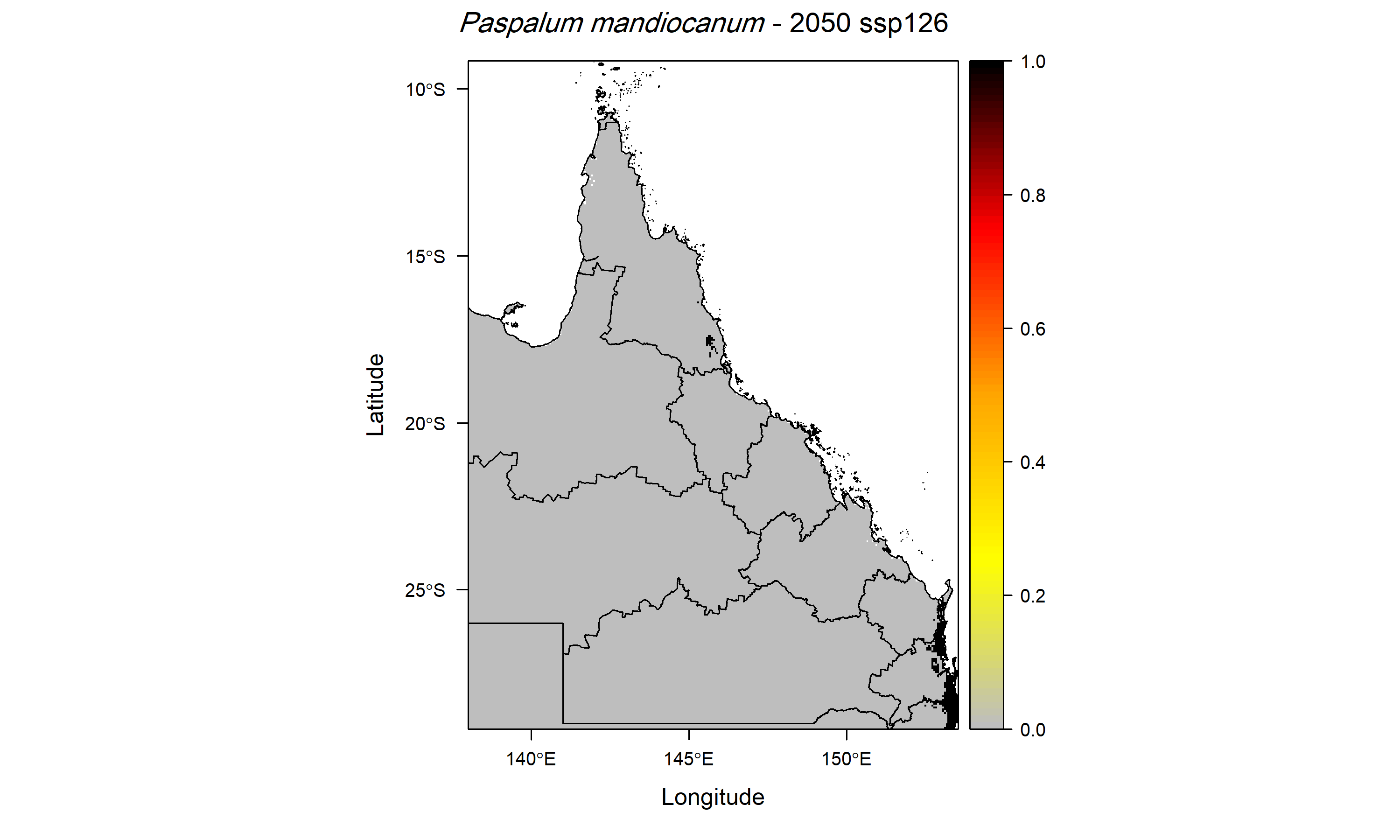

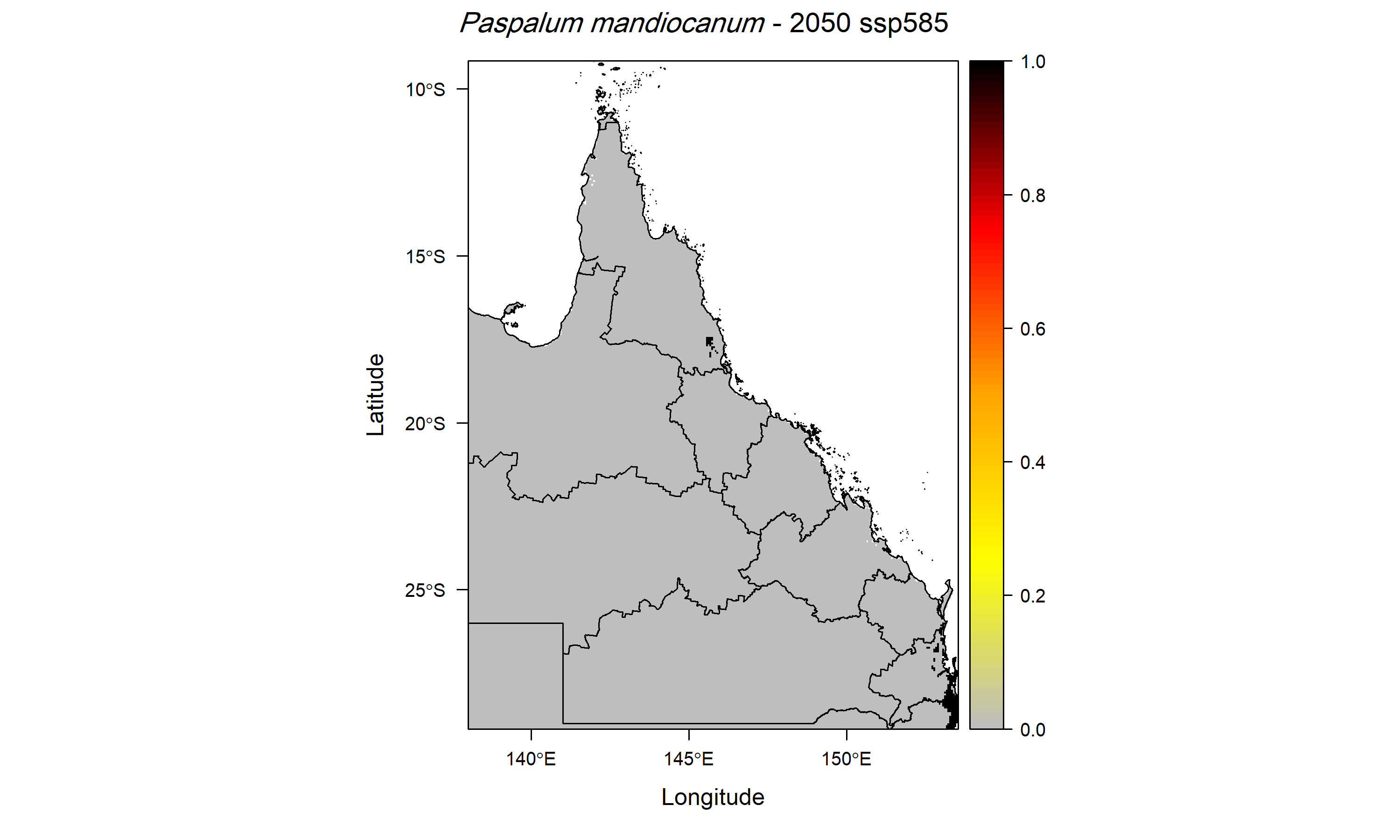

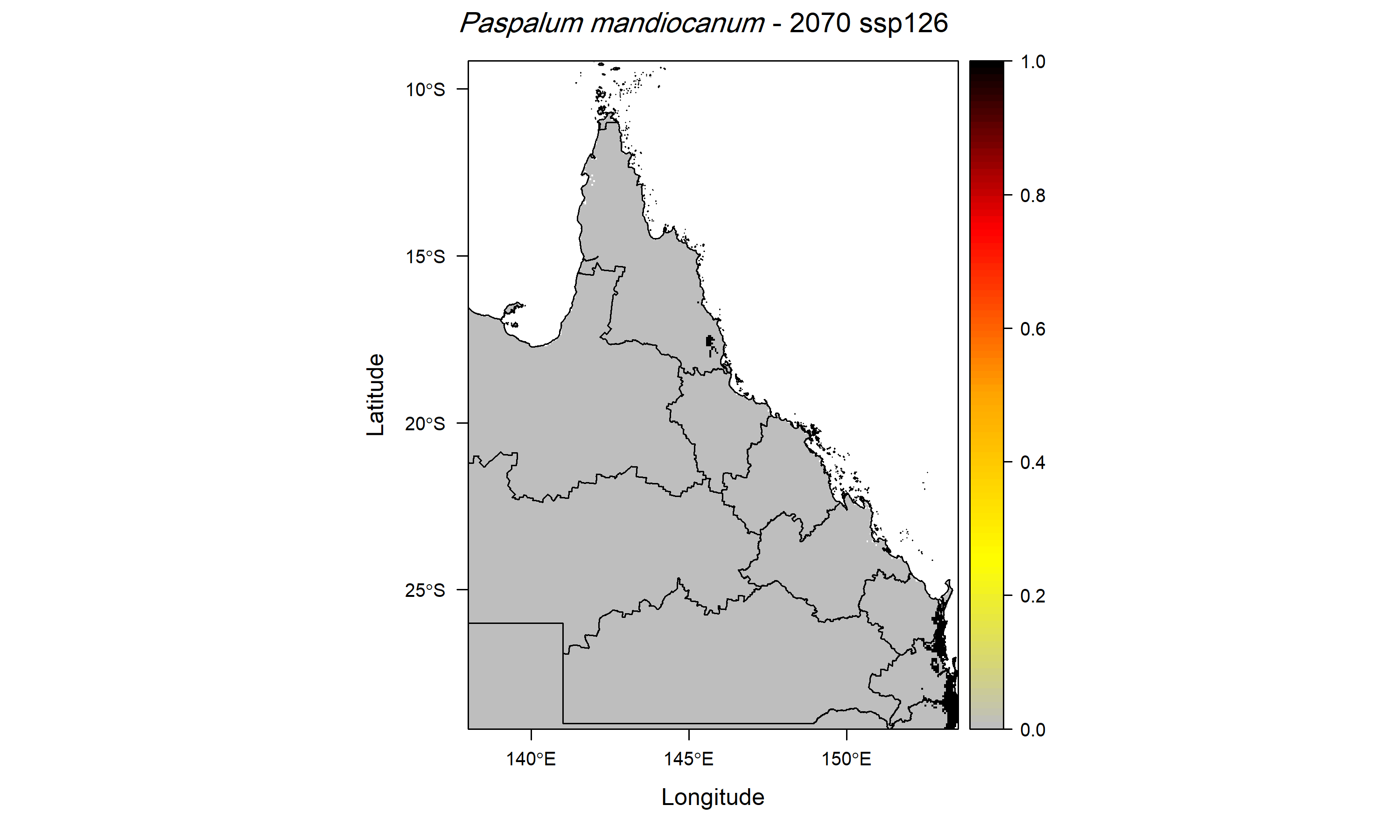

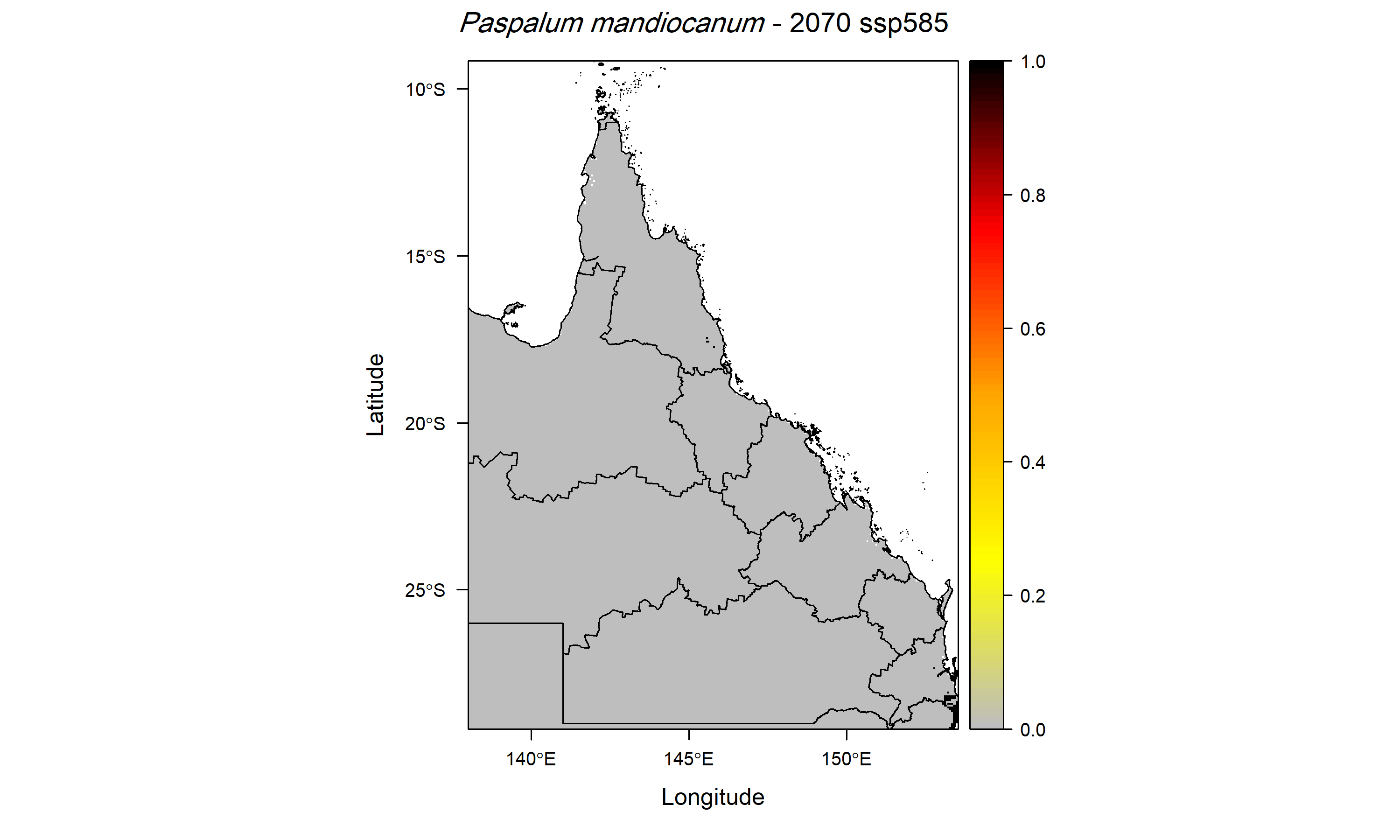


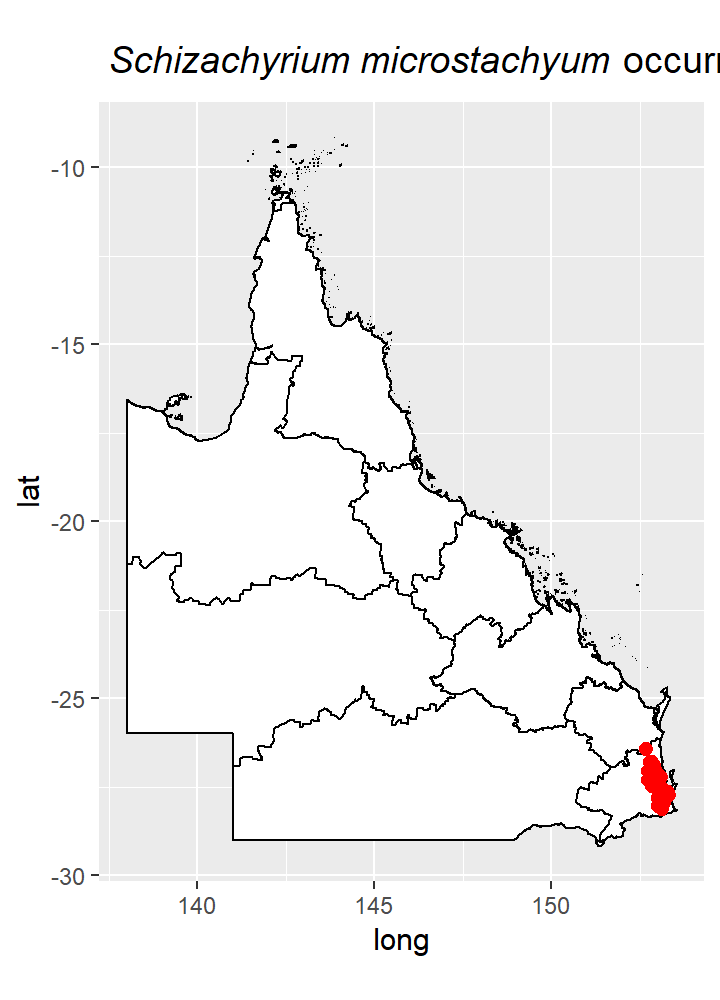

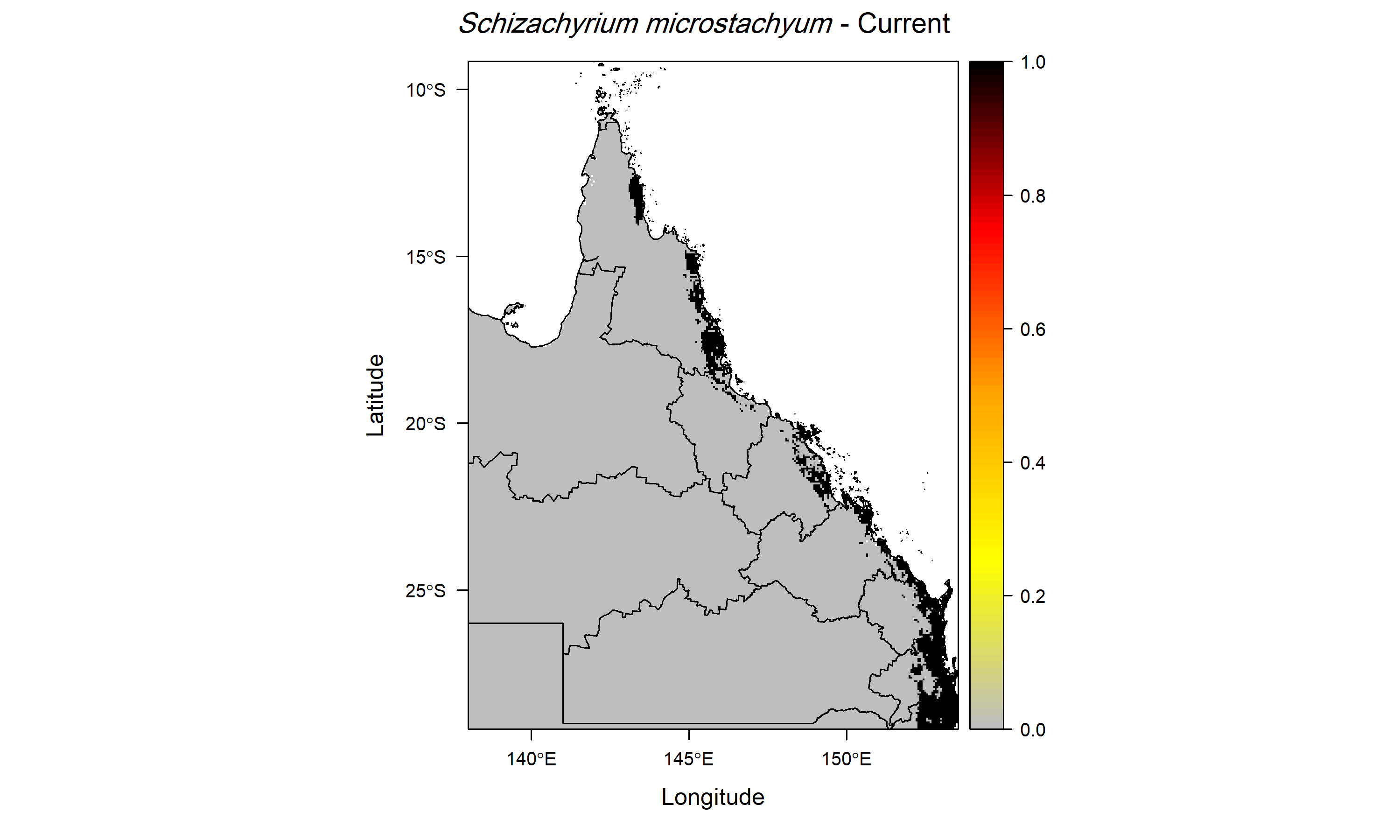

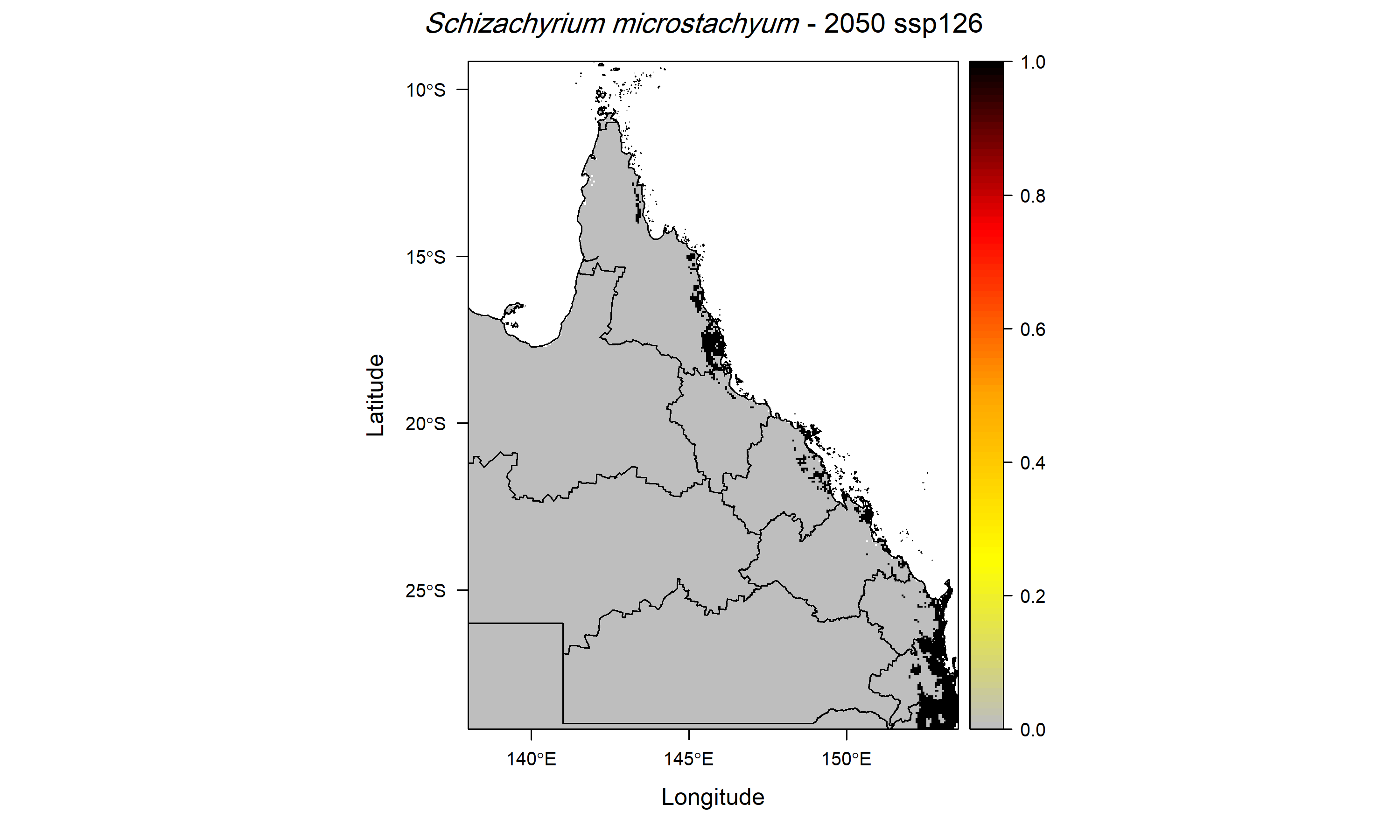

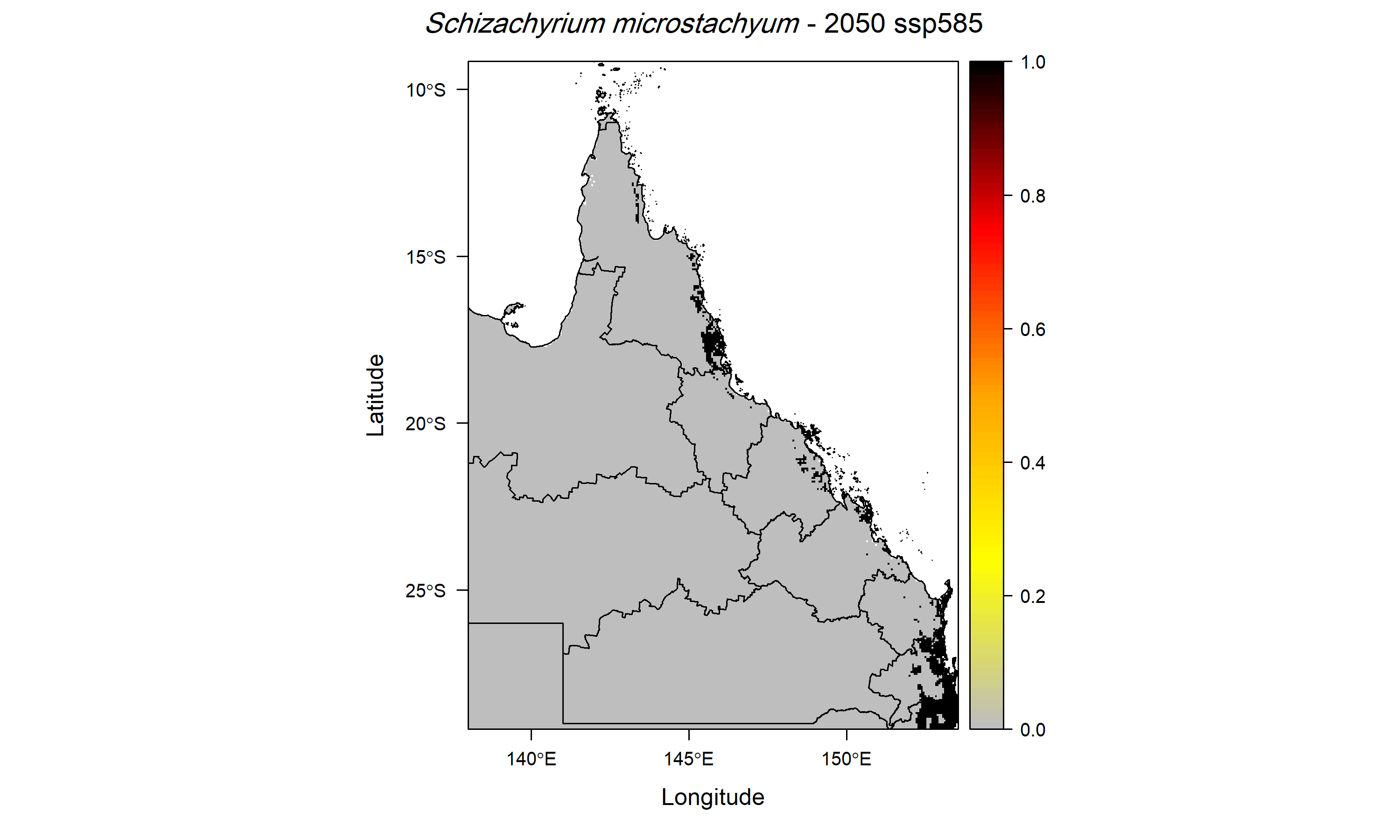

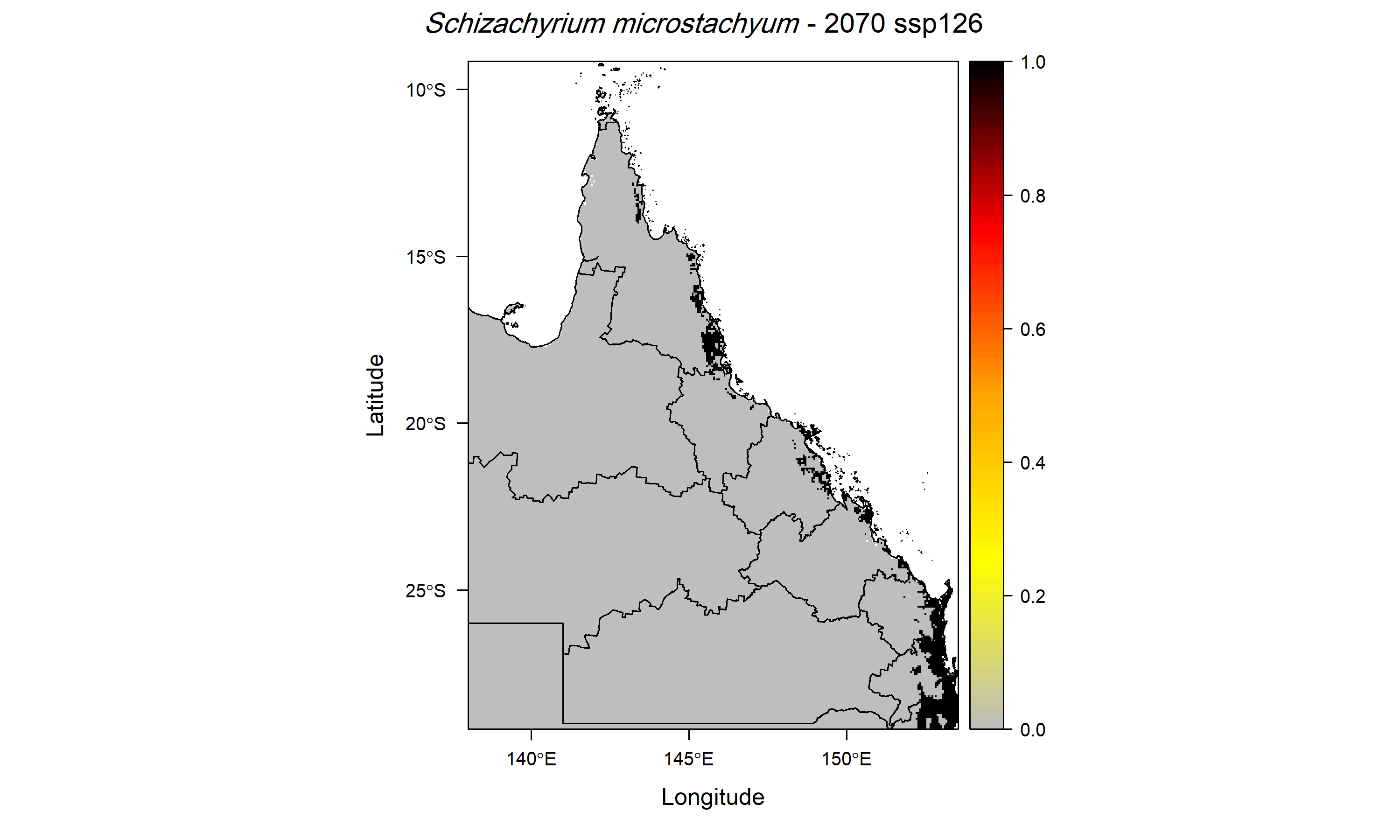

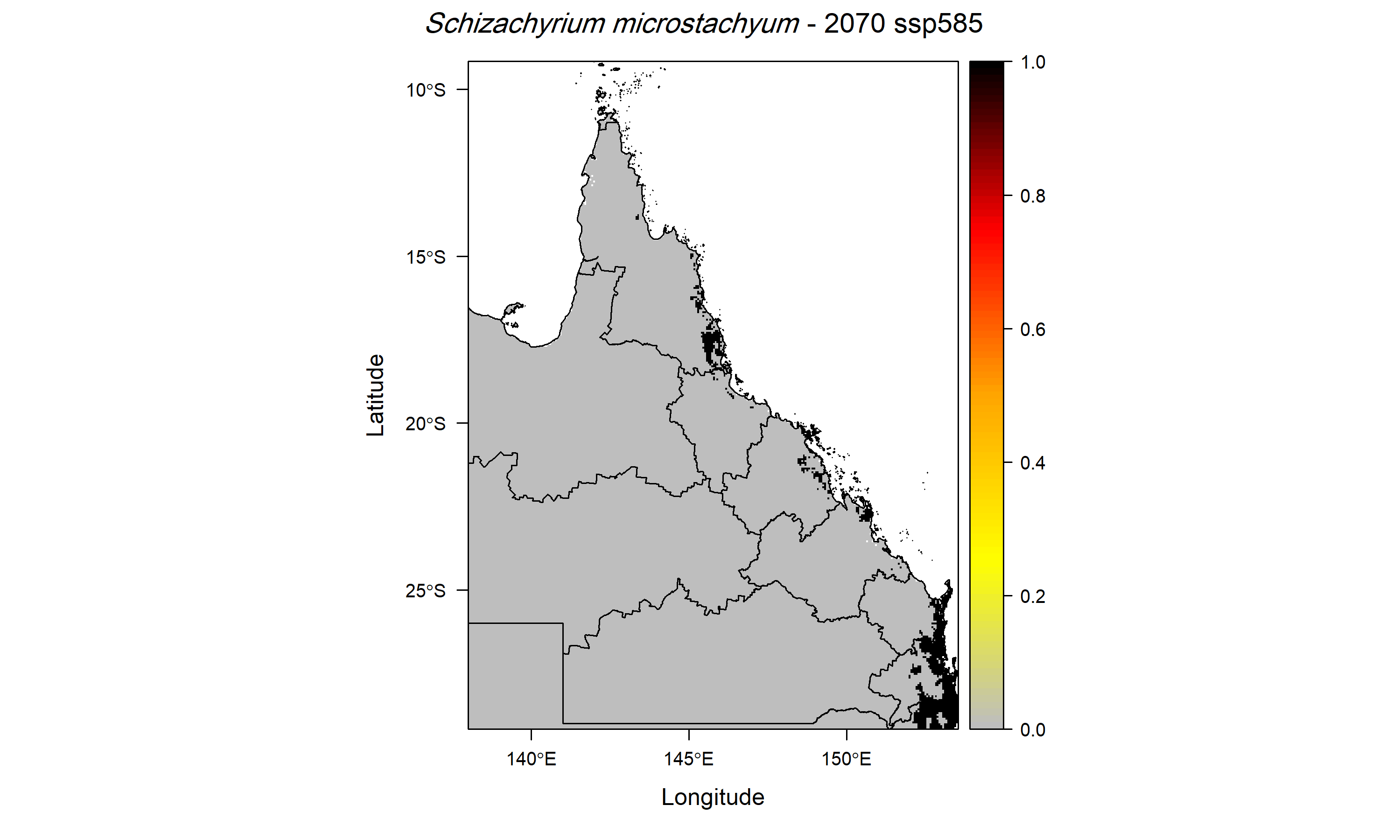


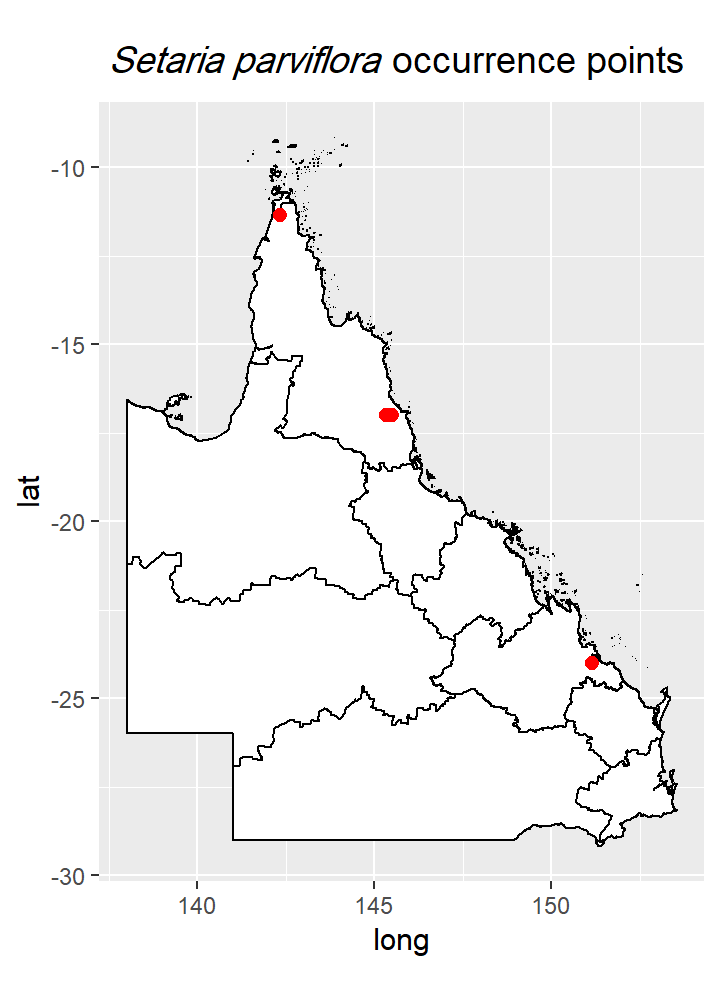

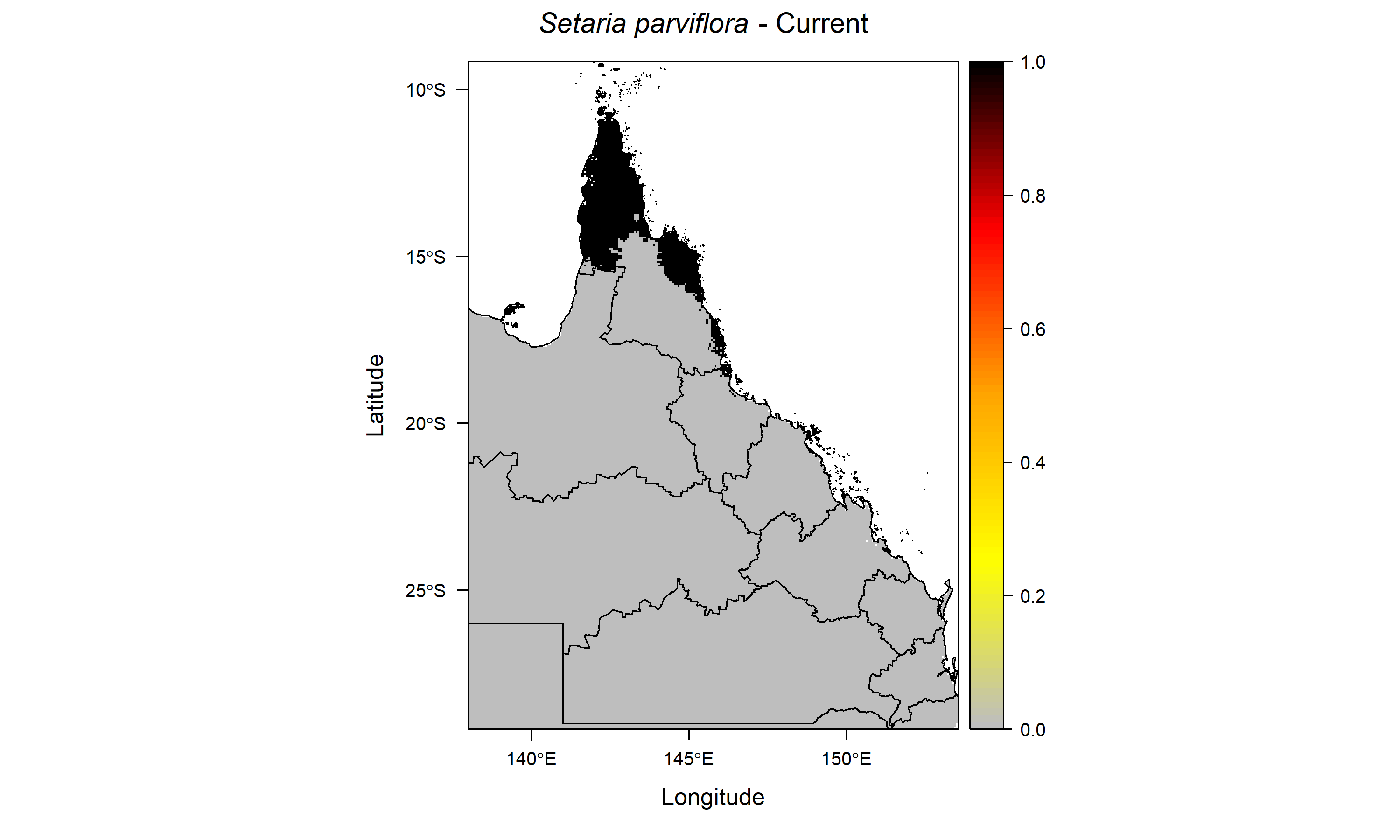

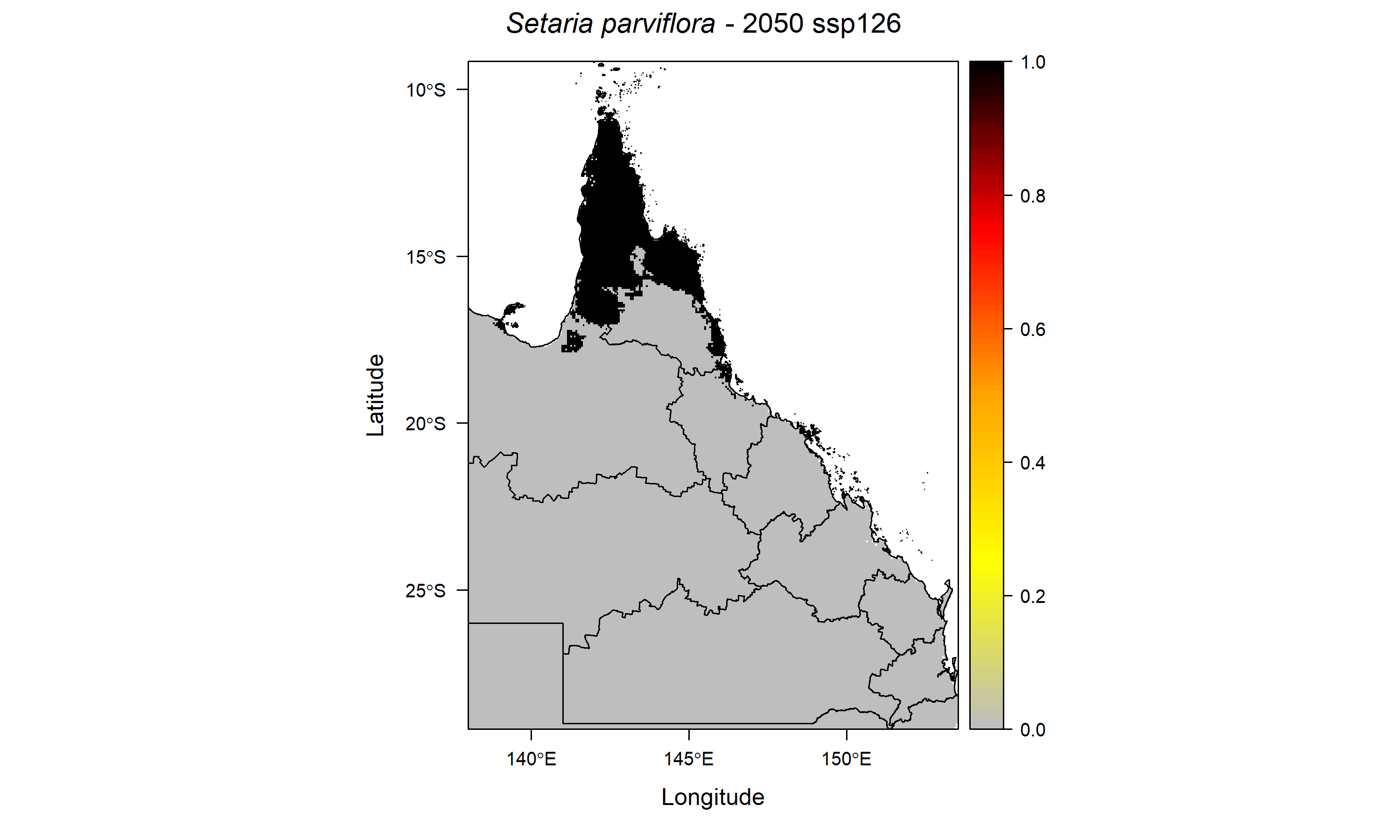

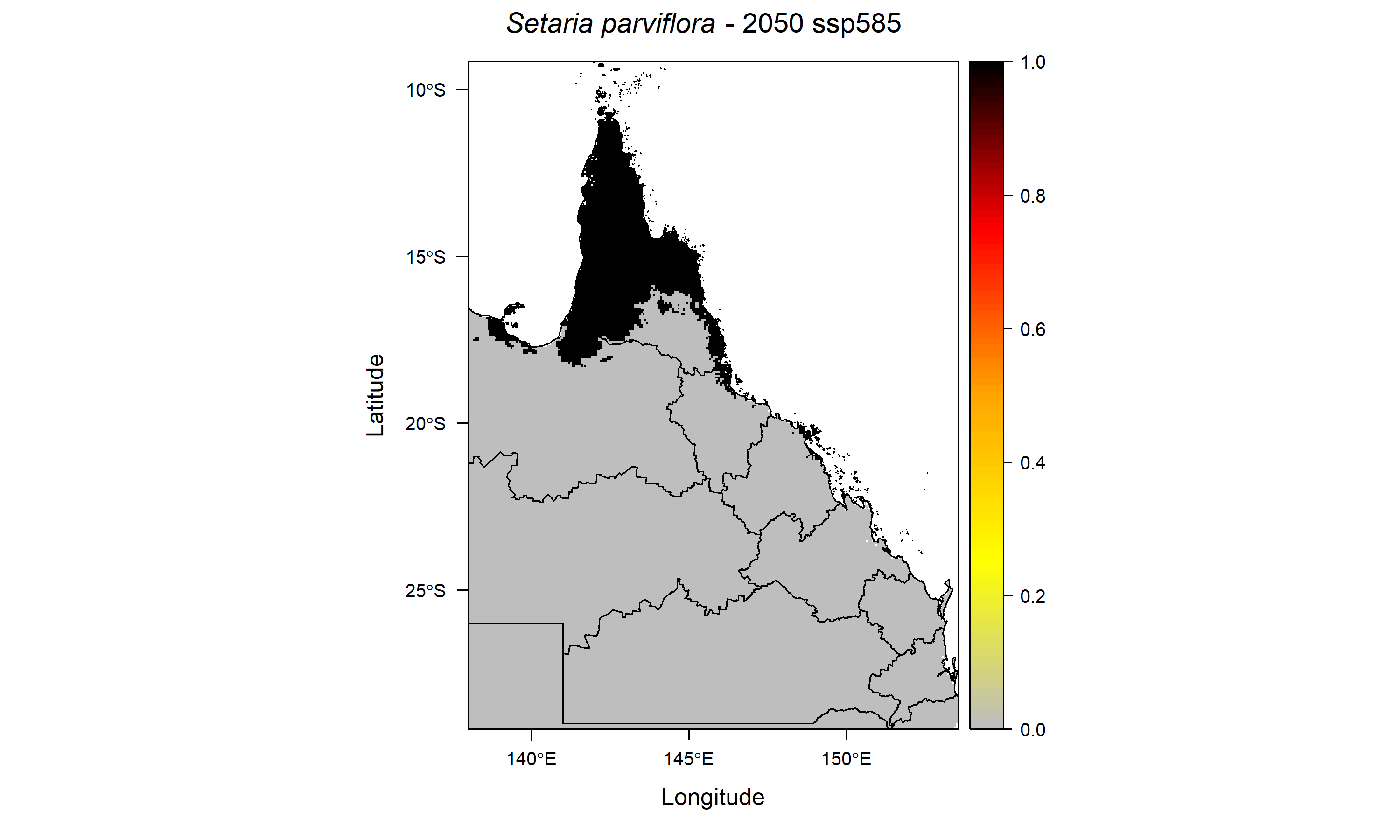

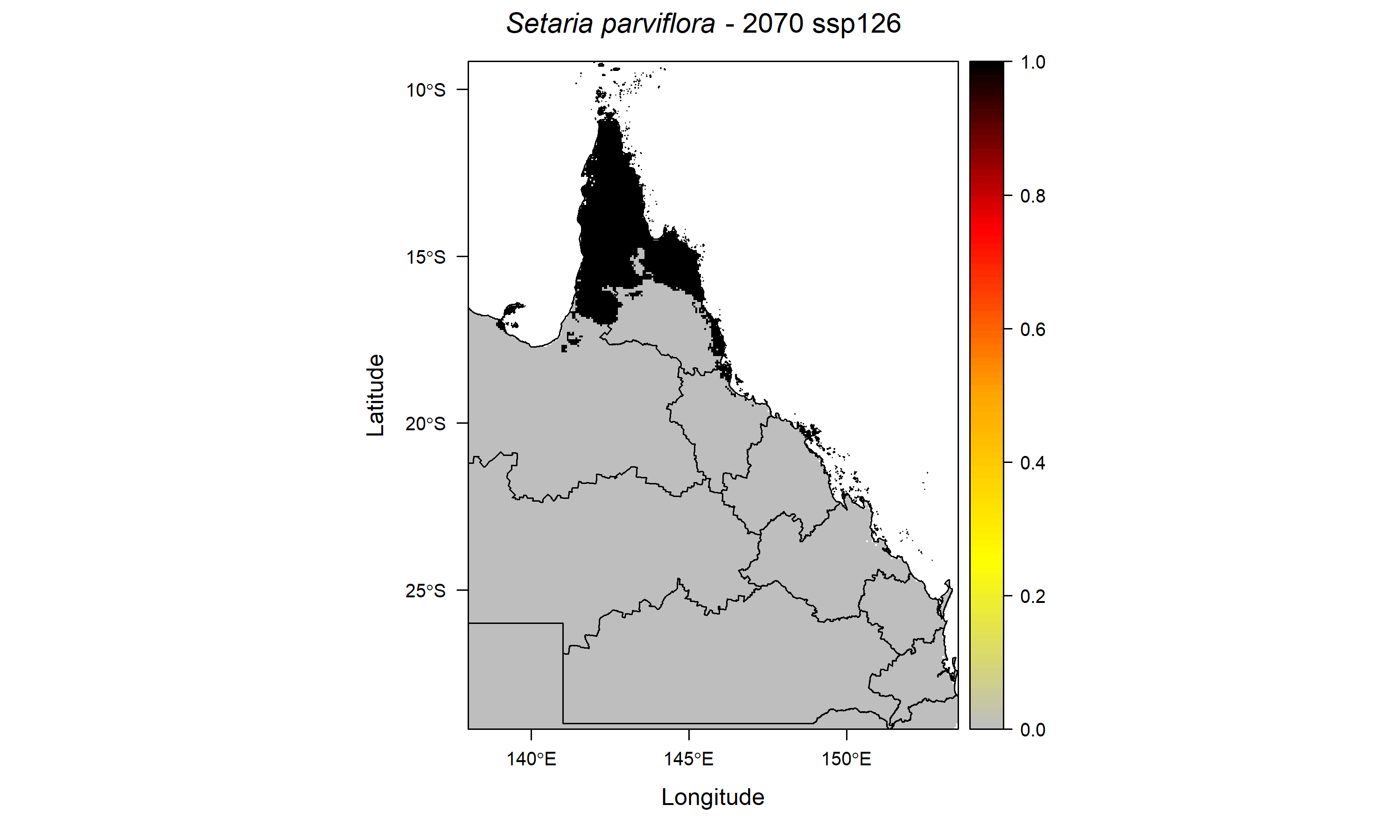

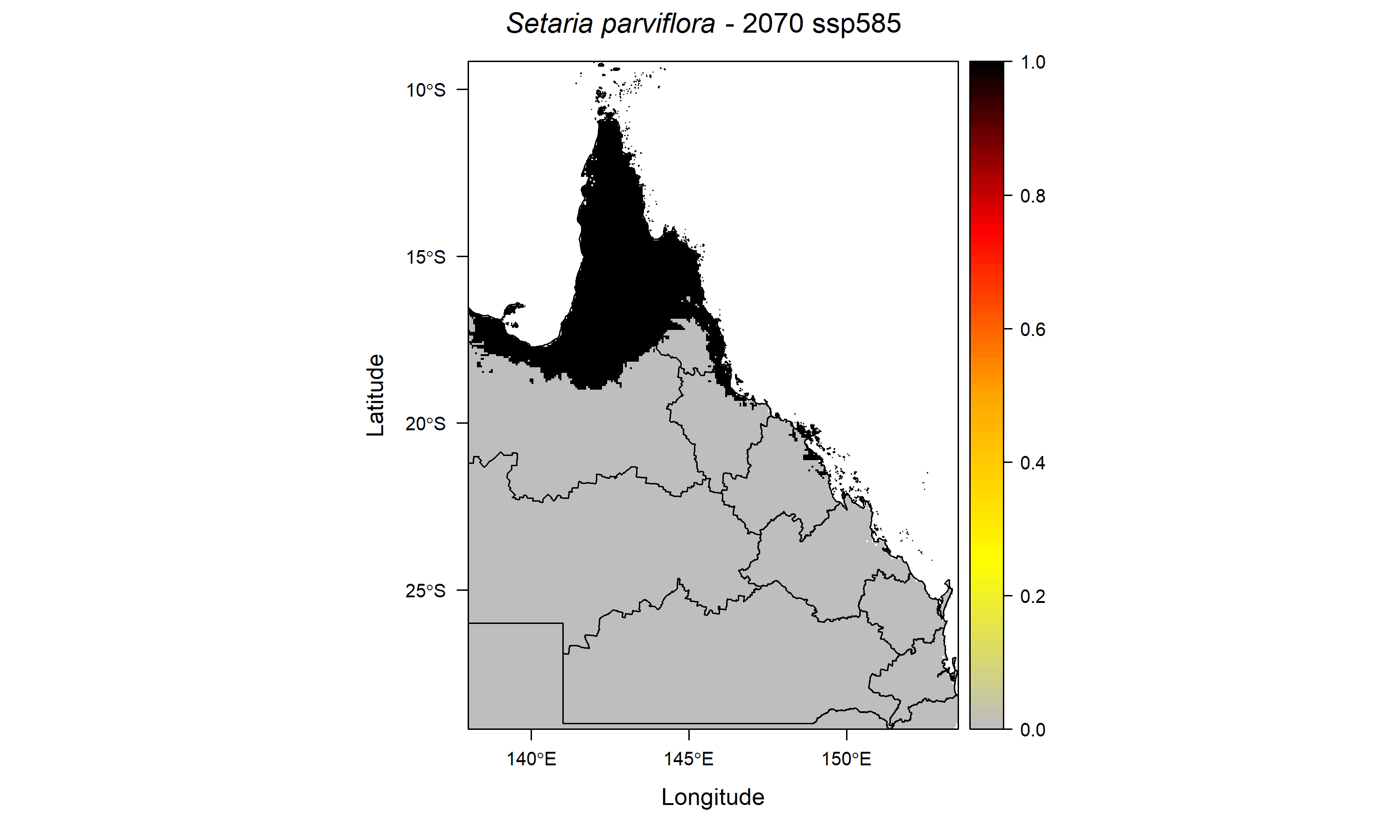


Appendix S2: Spatial distribution of 54 emerging weed species in QLD based on (i) actual occurrence points (ii) potential range, (iii, iv) range response to climate change scenarios in 2050 and (v, vi) range response to climate change scenarios in 2070. Note that species are organised by plant growth form – Grass (9)-Herb (14)-Shrub (8)-Tree (10)-Succulent (6)-Vine (7)

(B) HERB- 14 species

1. (ii) (iii) (iv) (v) (vi)


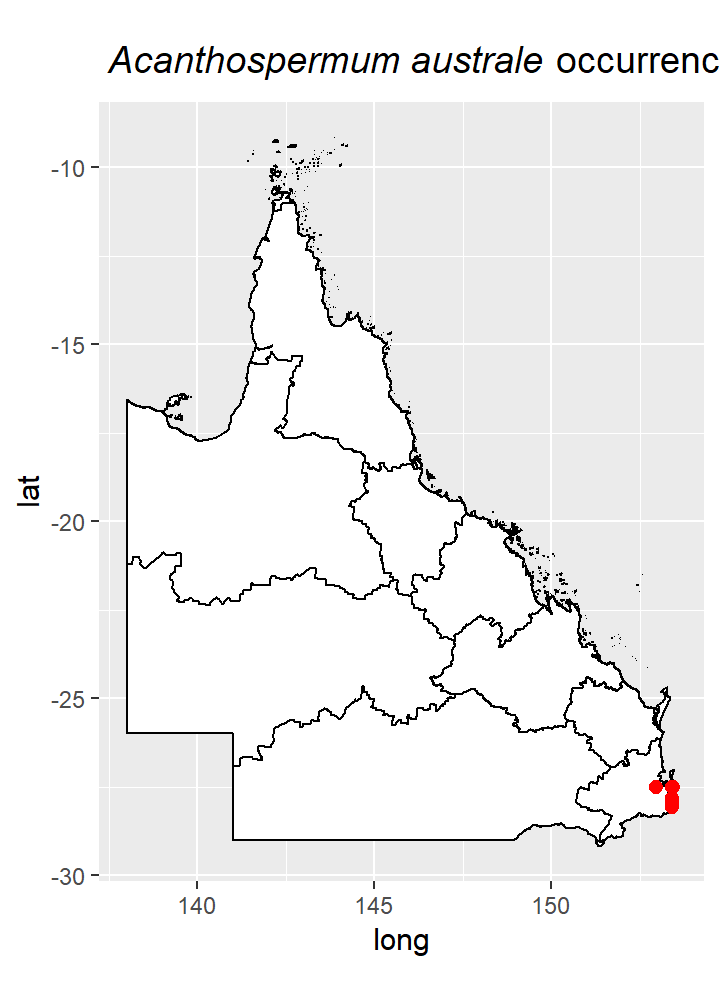

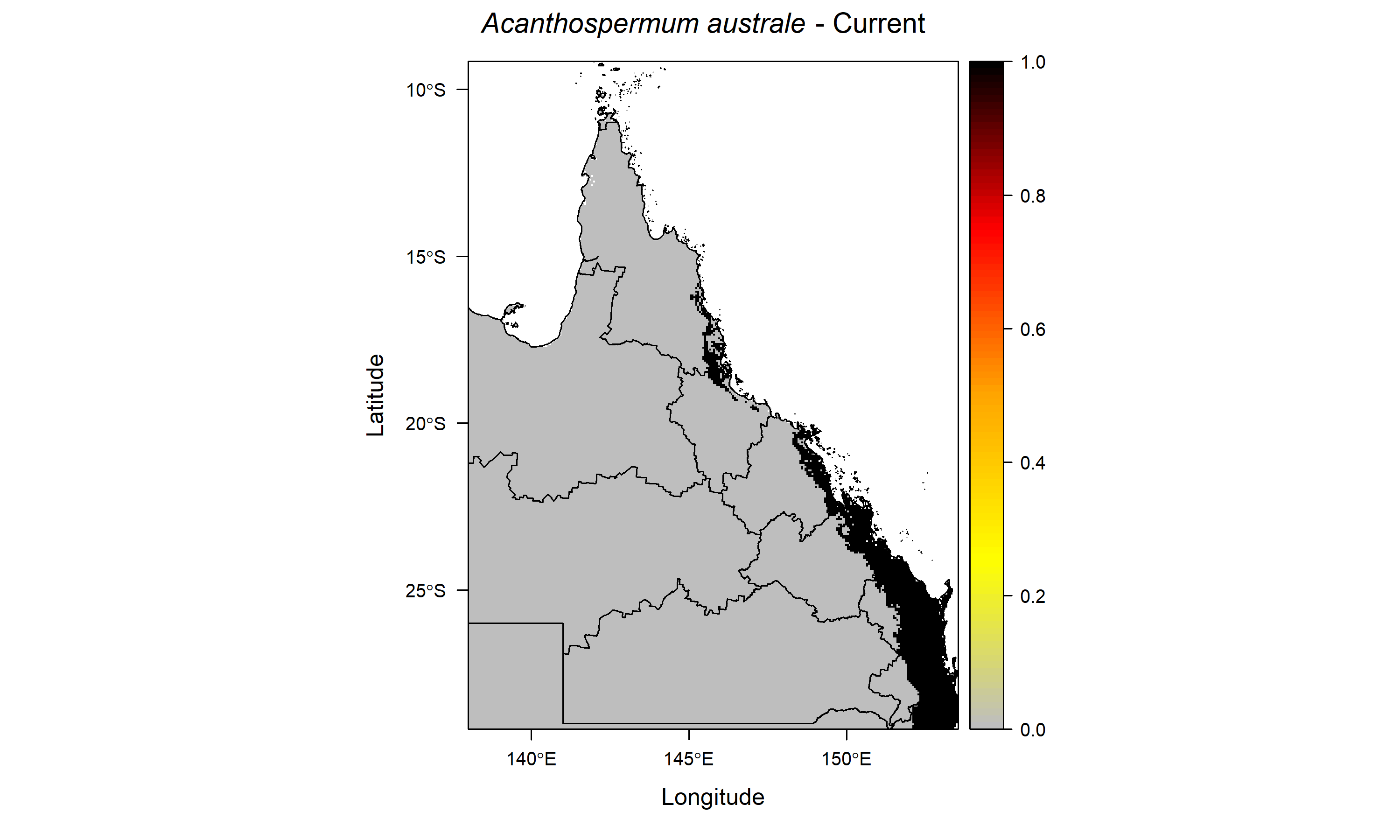

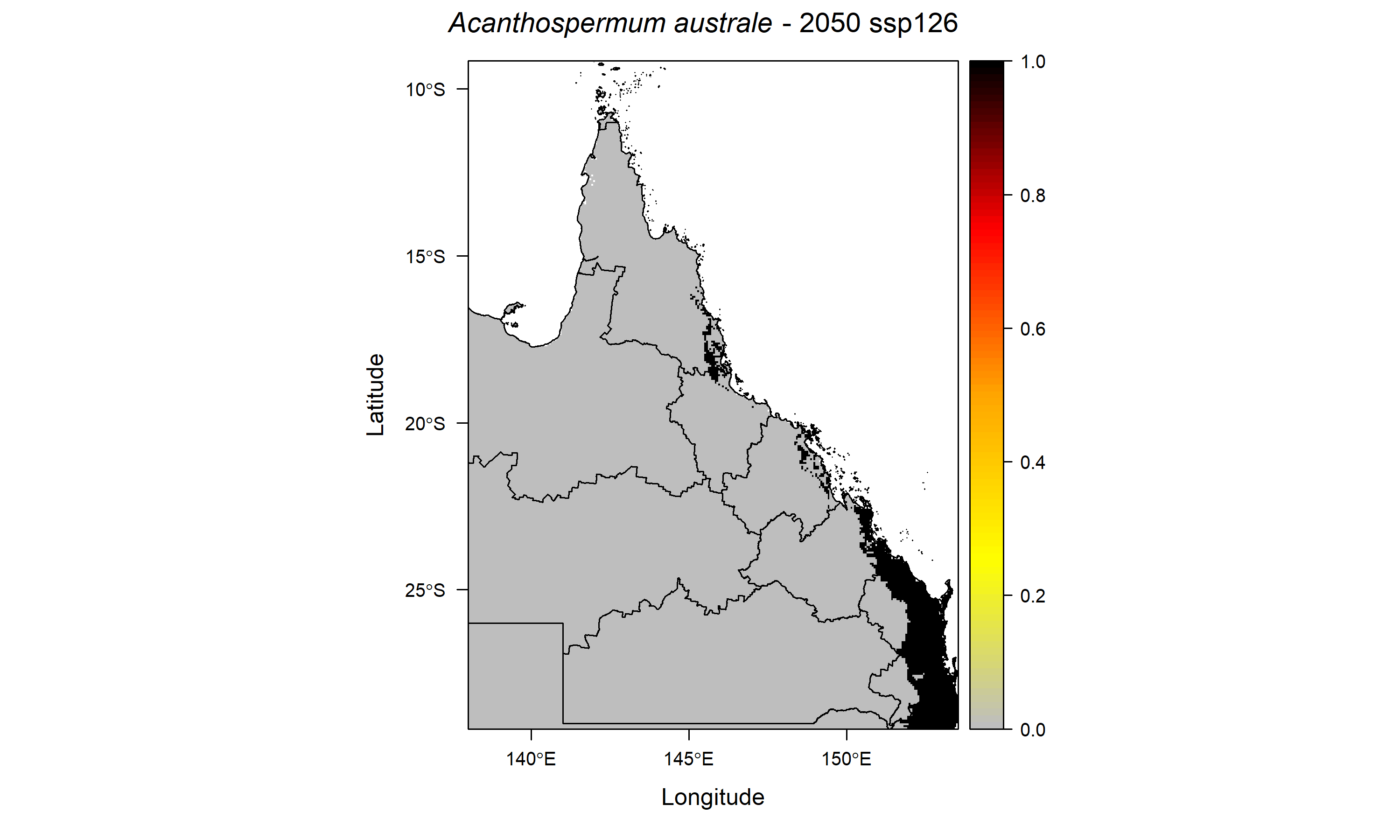

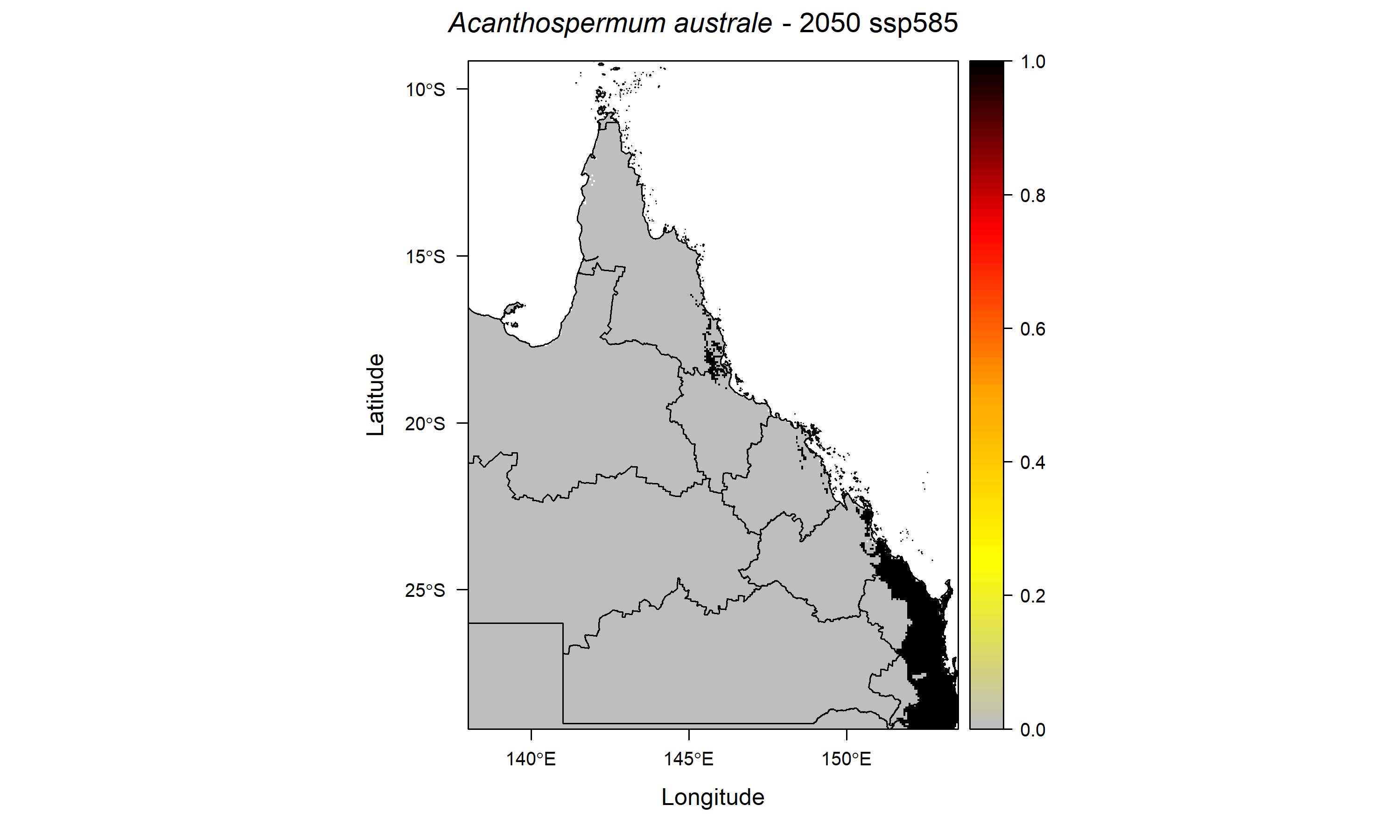

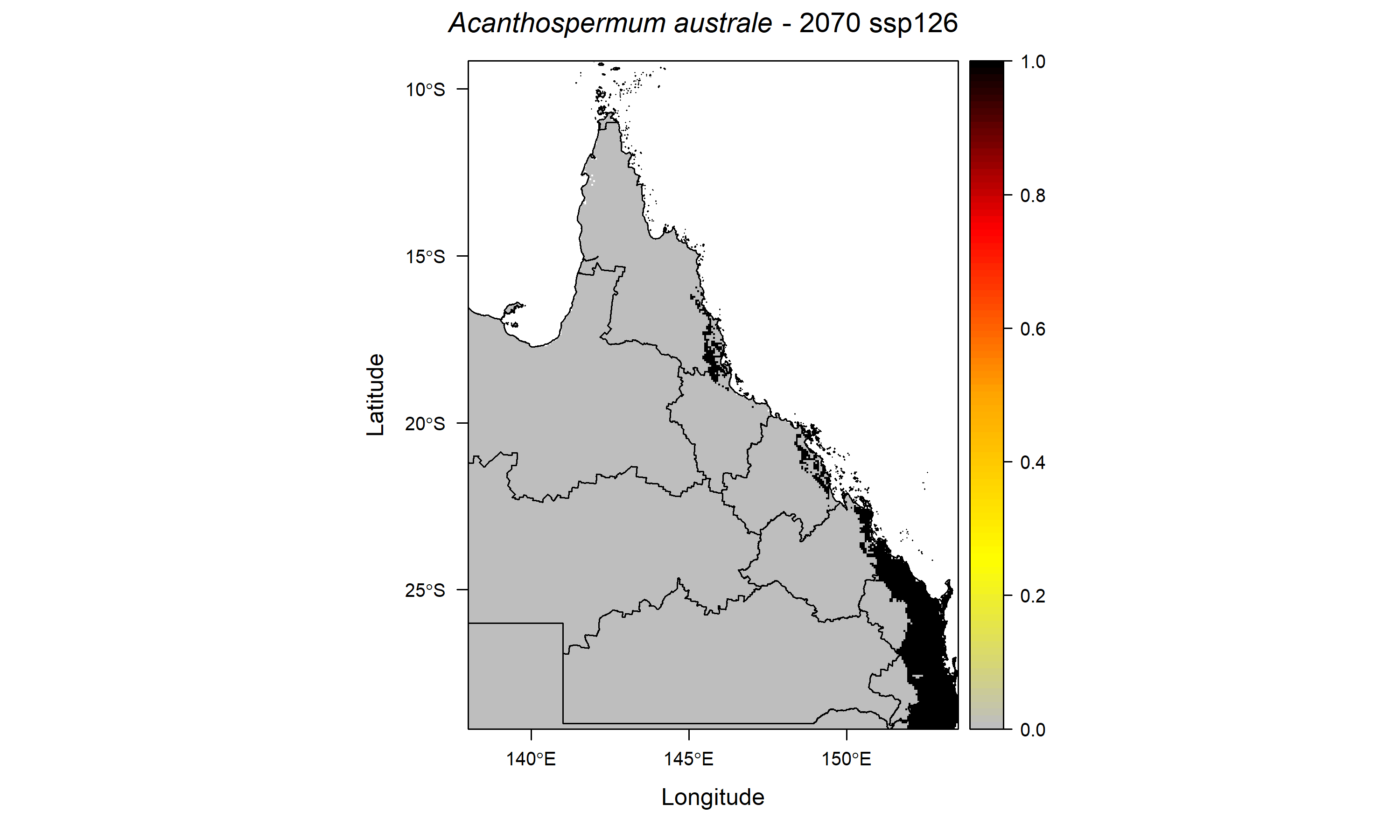

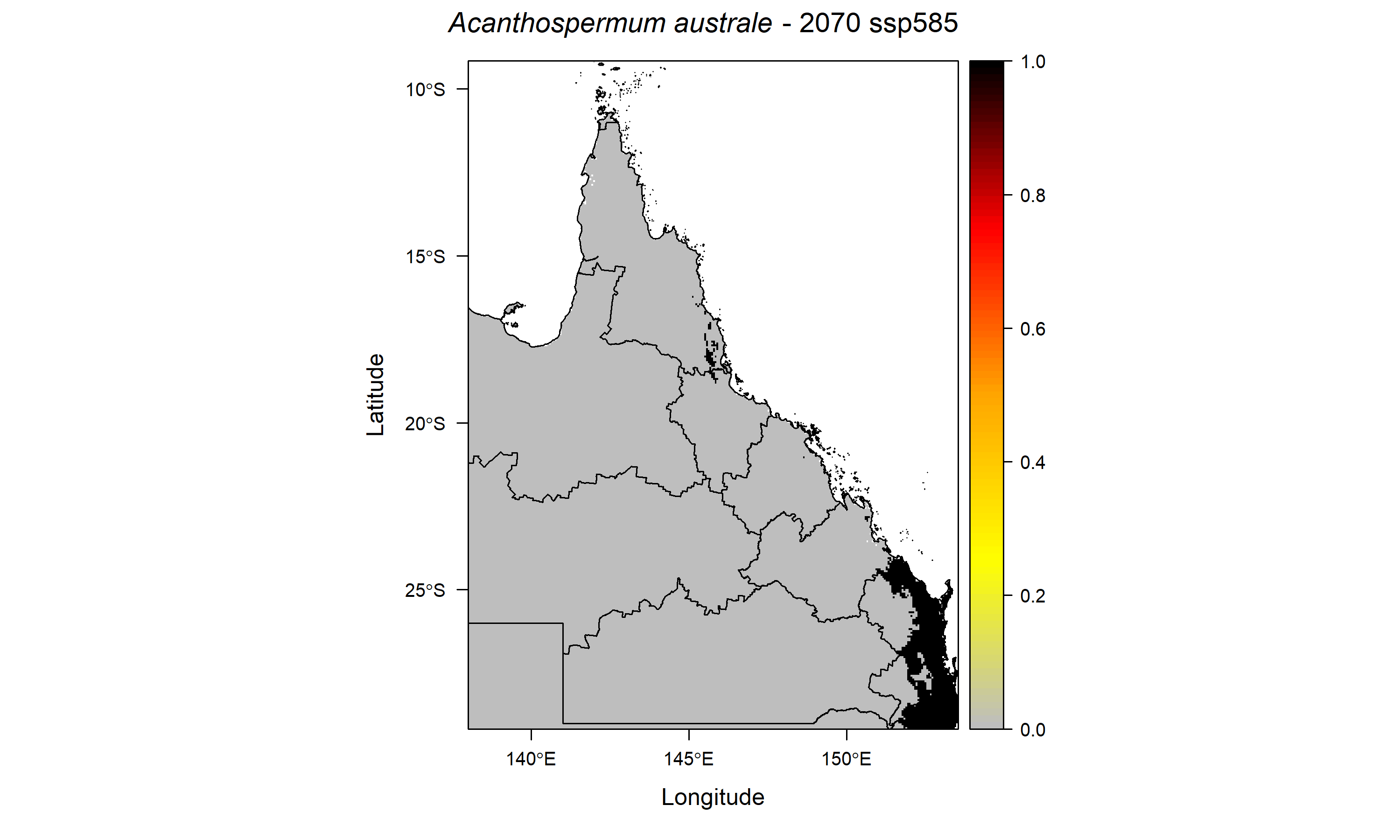


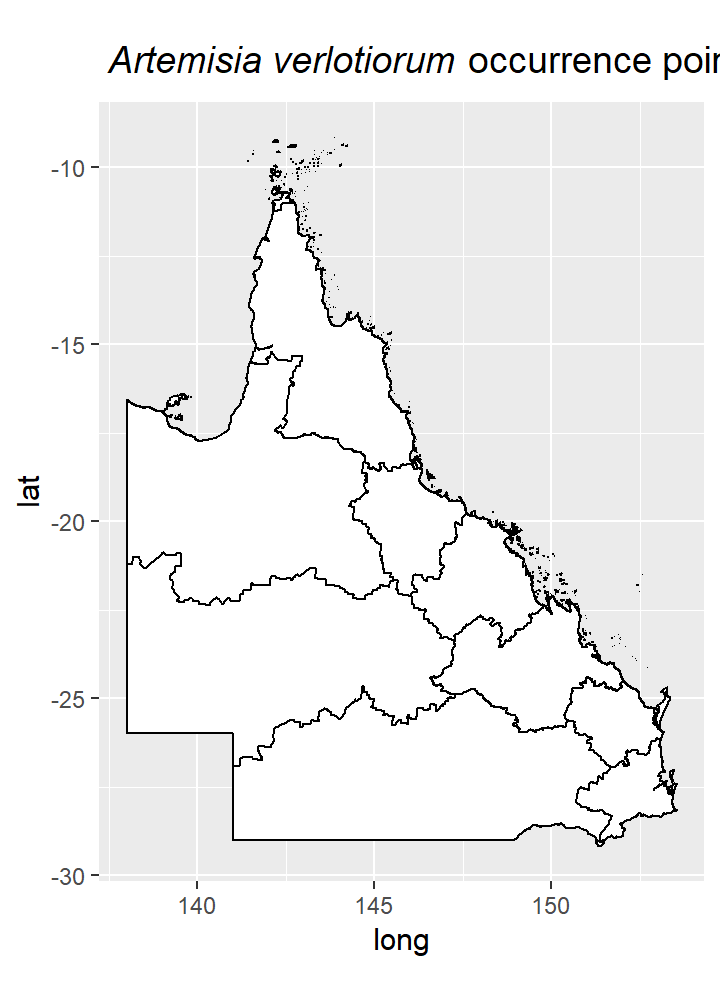

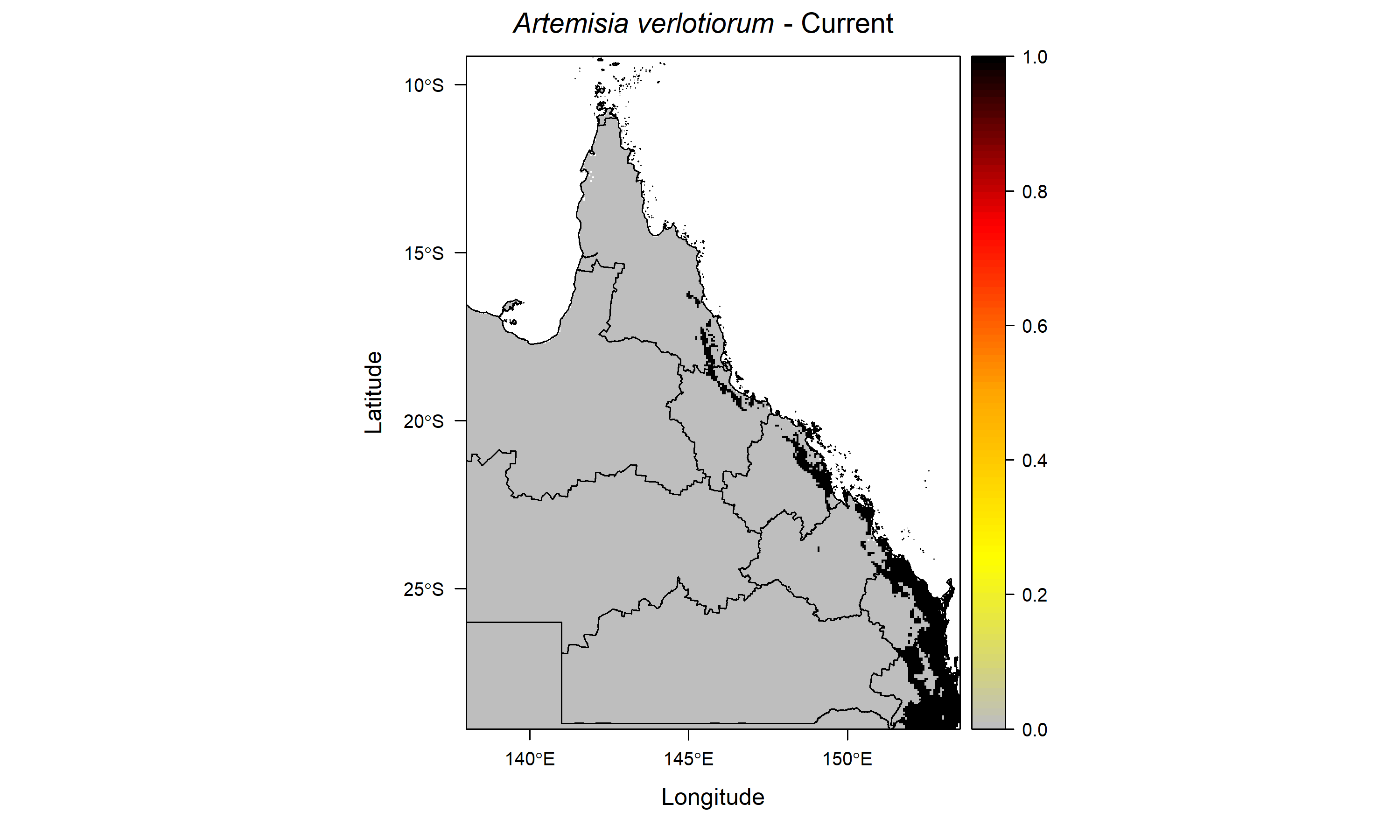

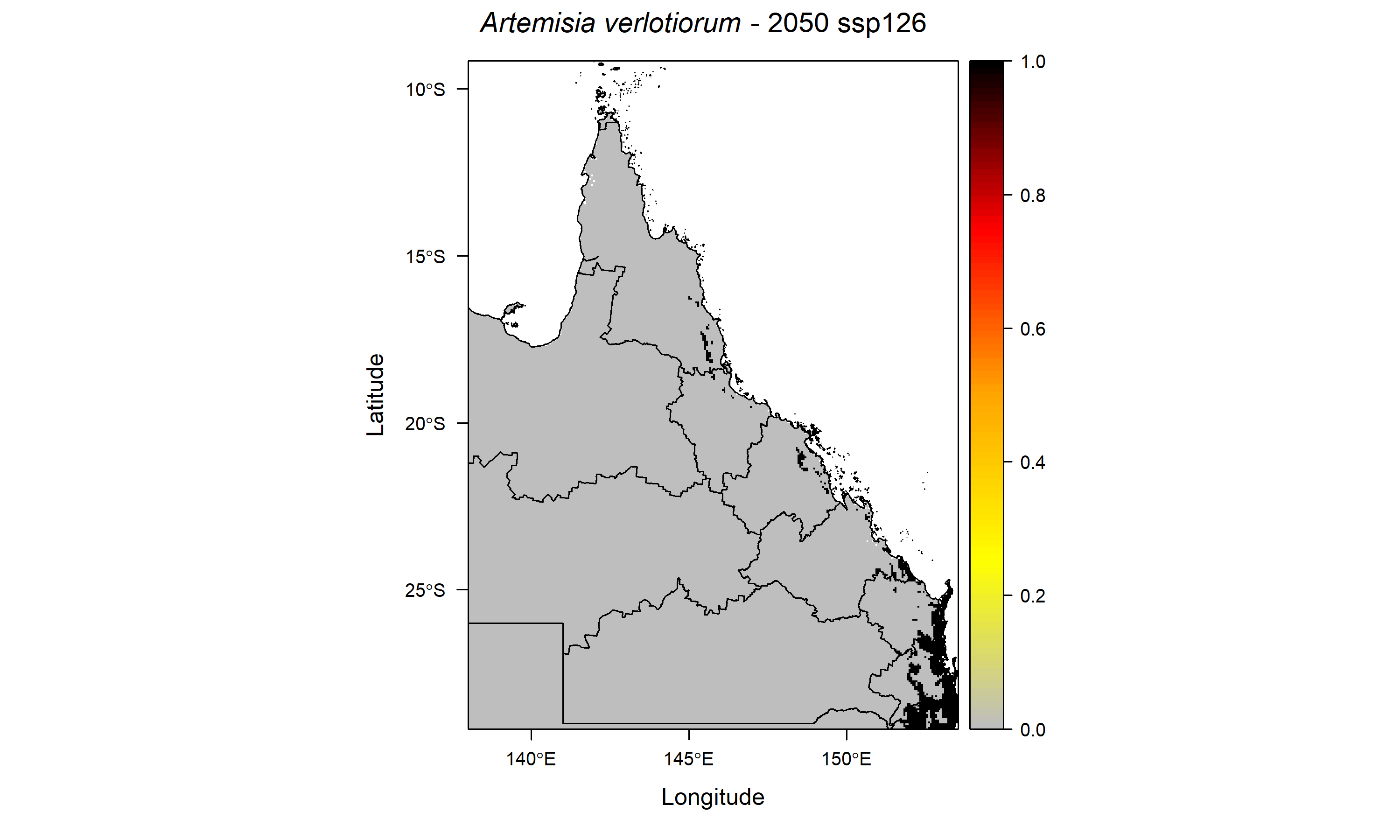

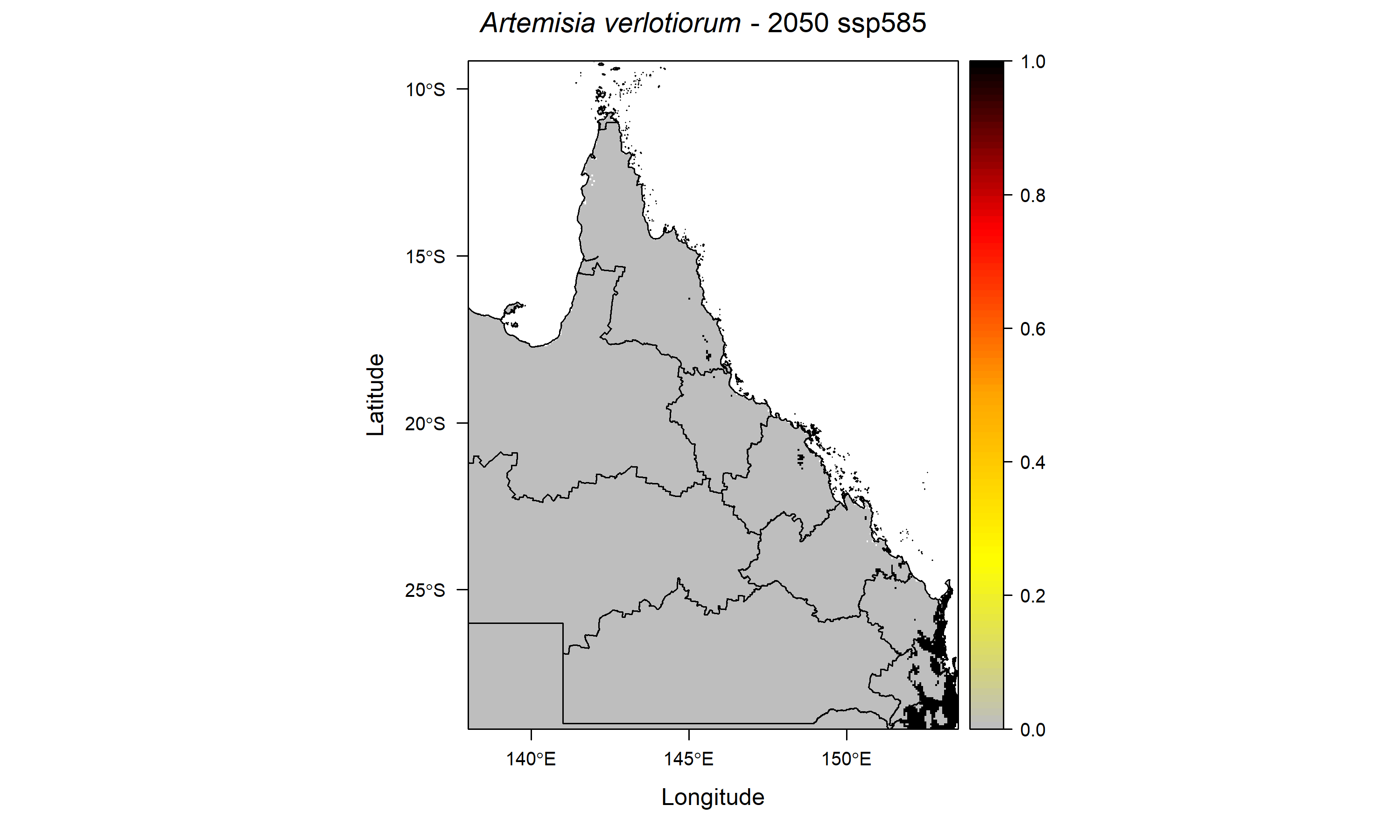

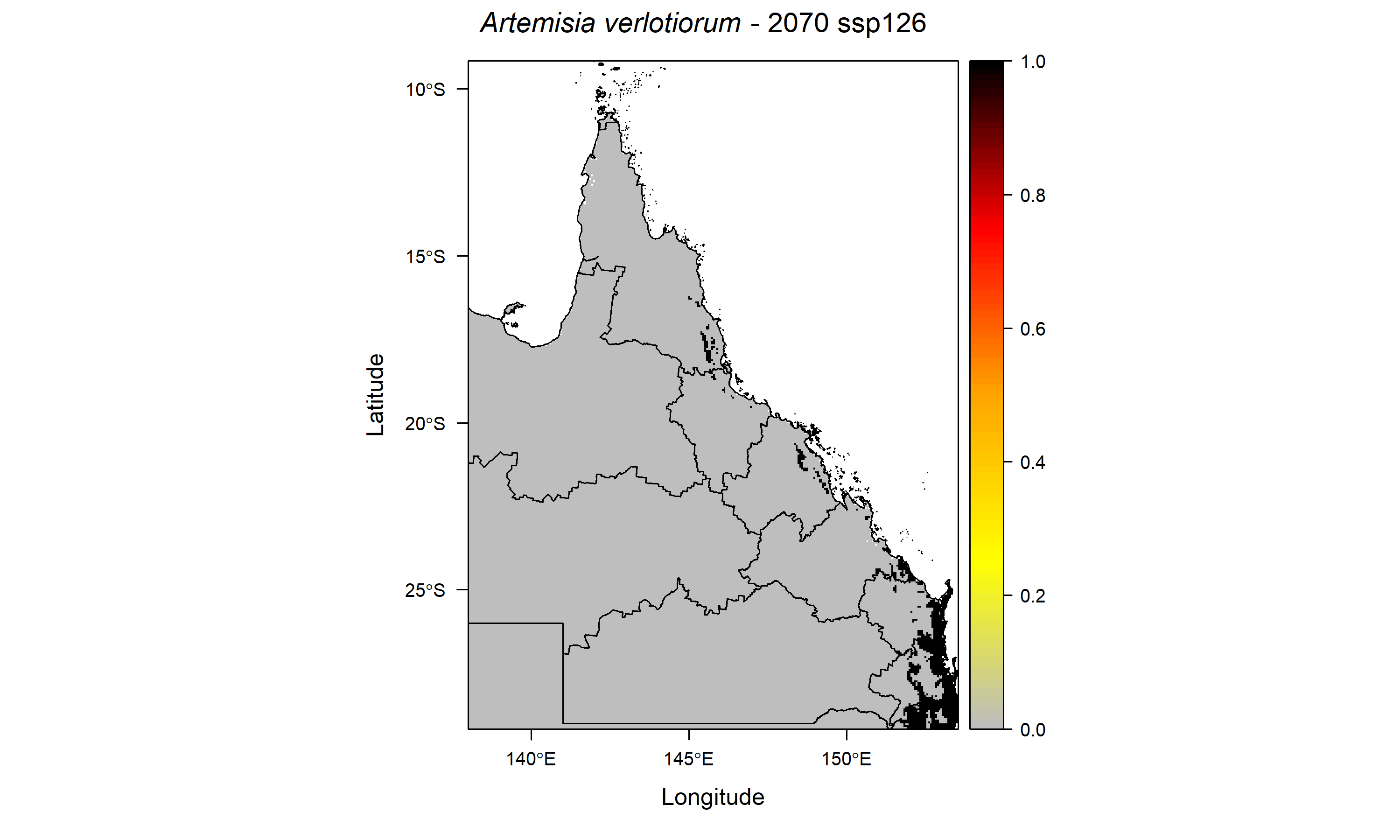

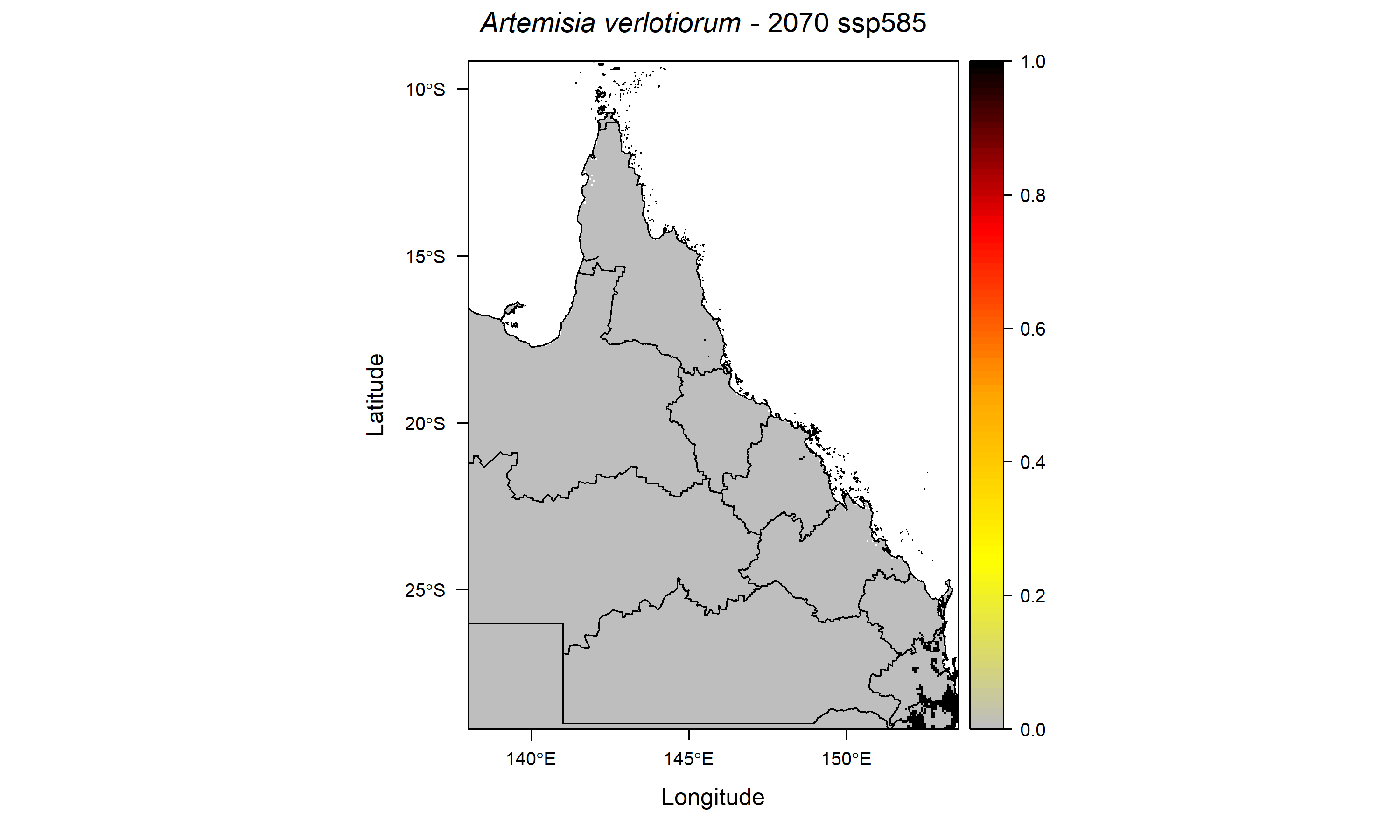


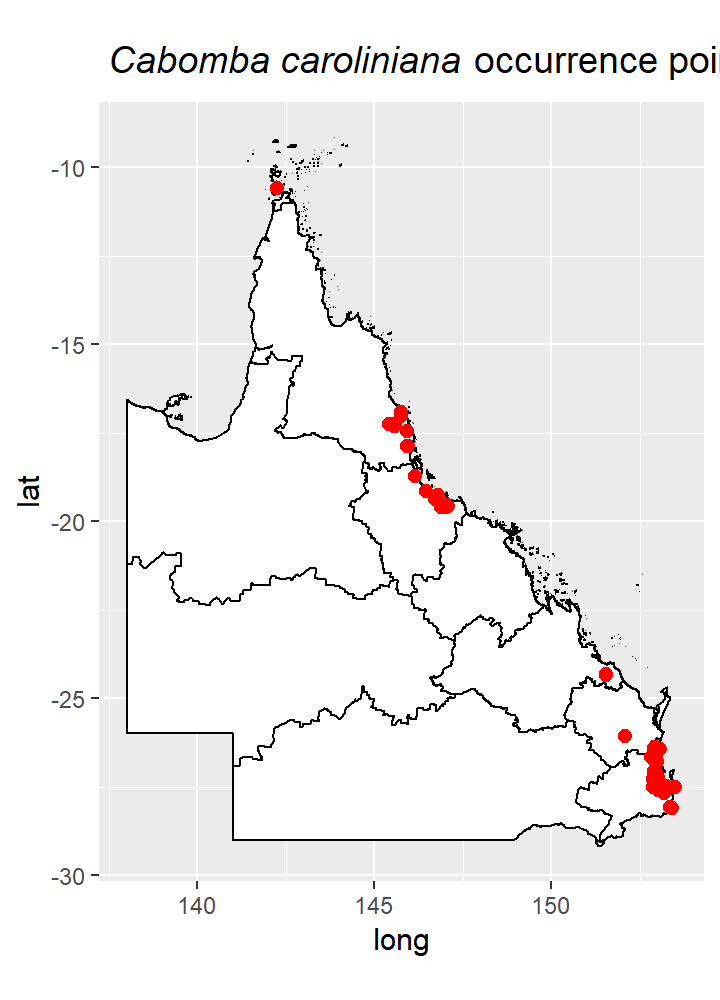

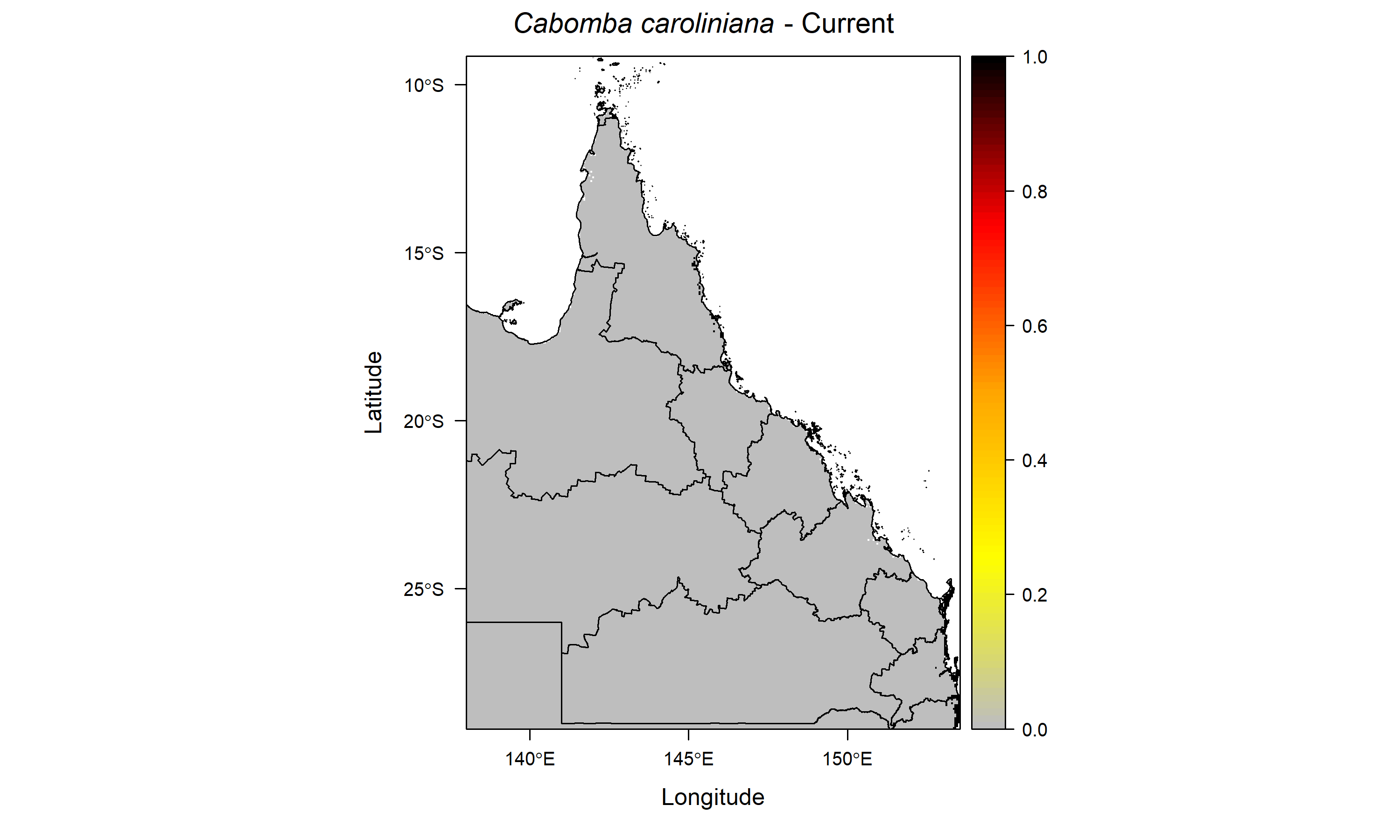

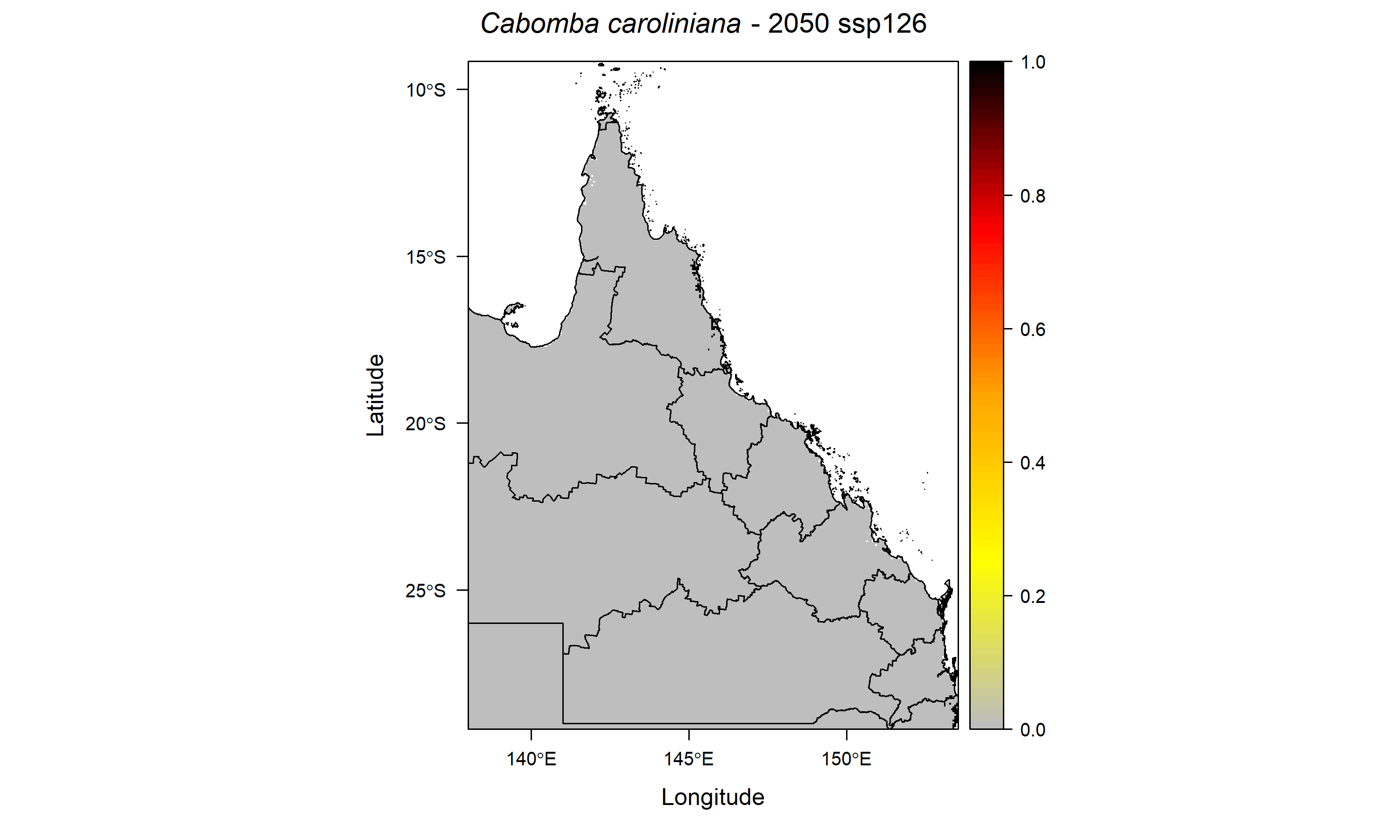

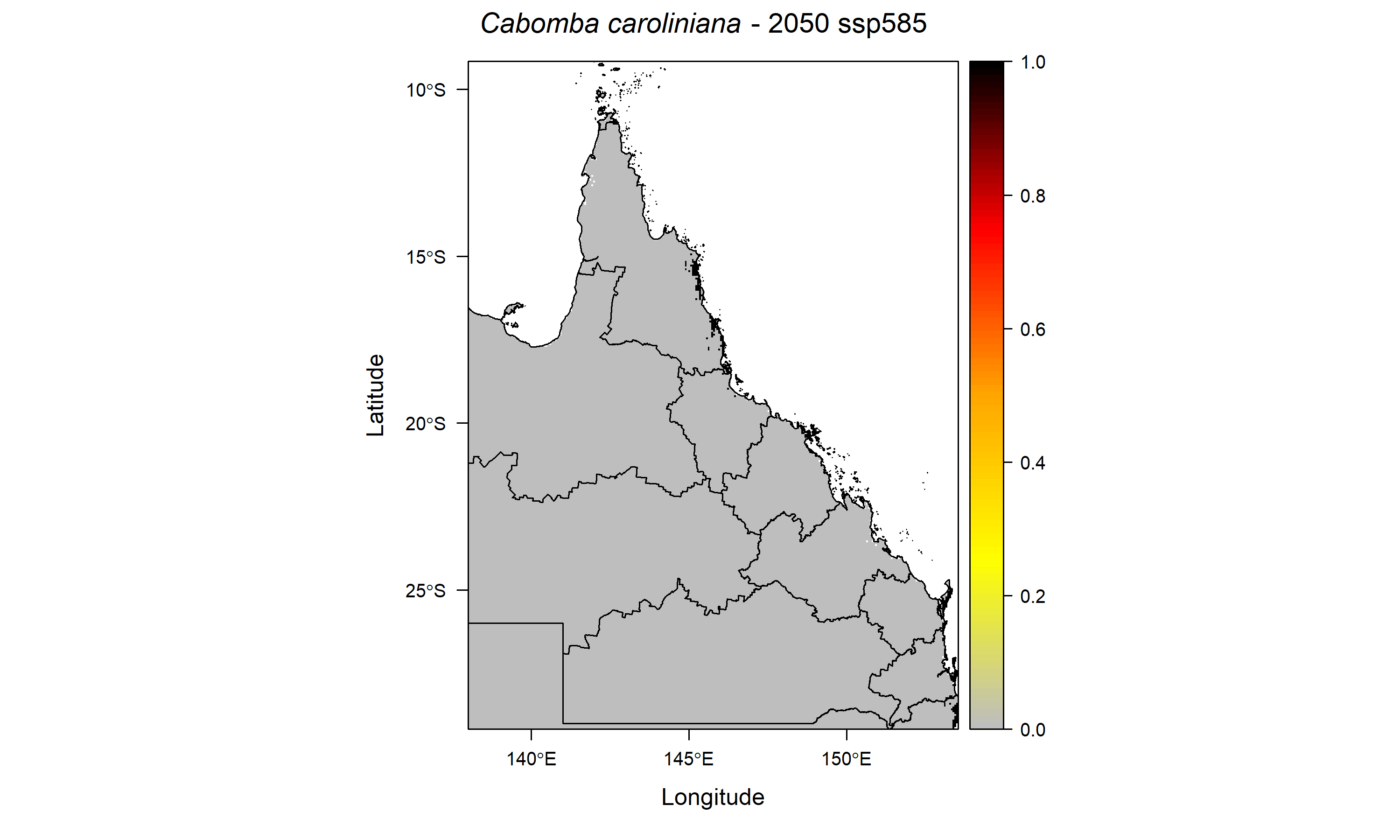

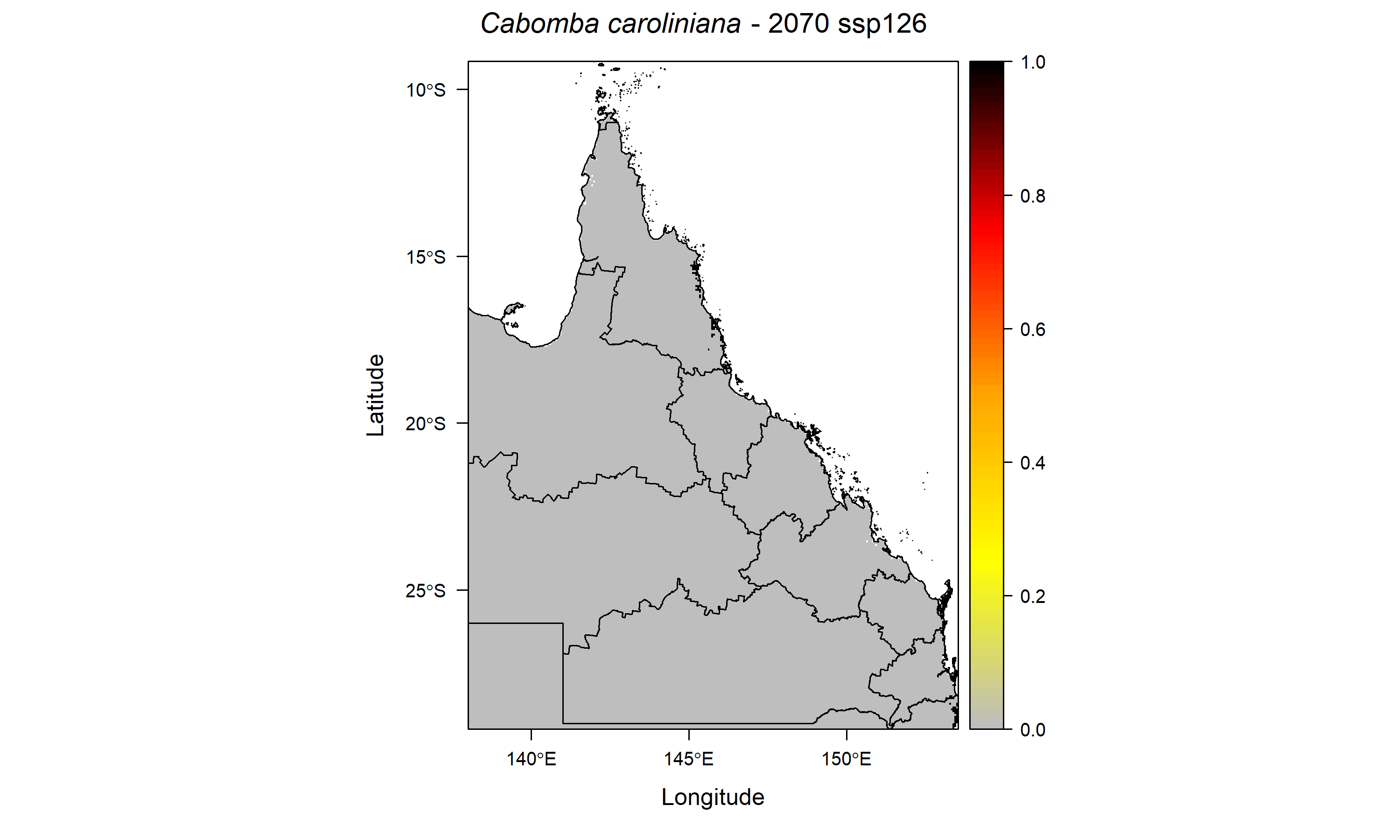

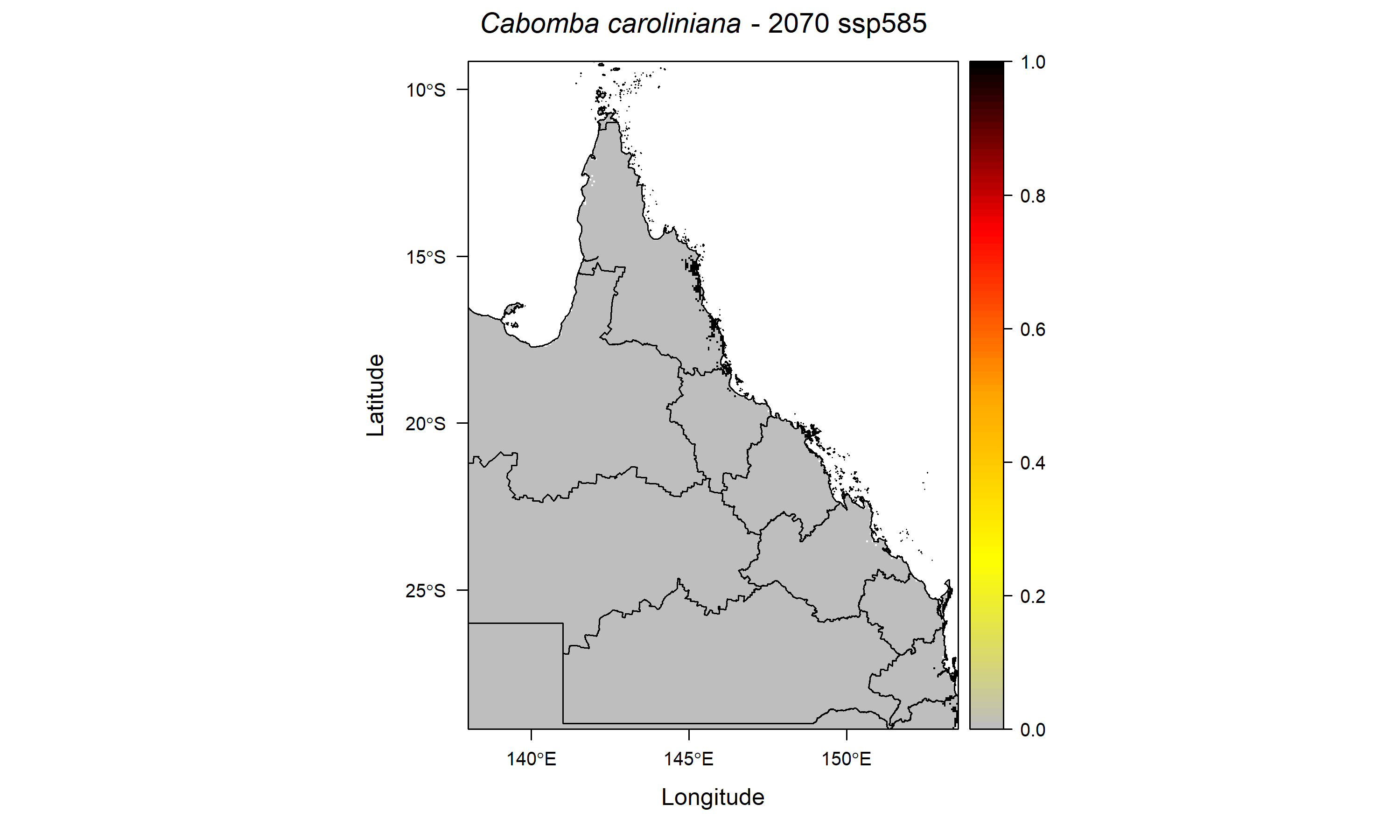


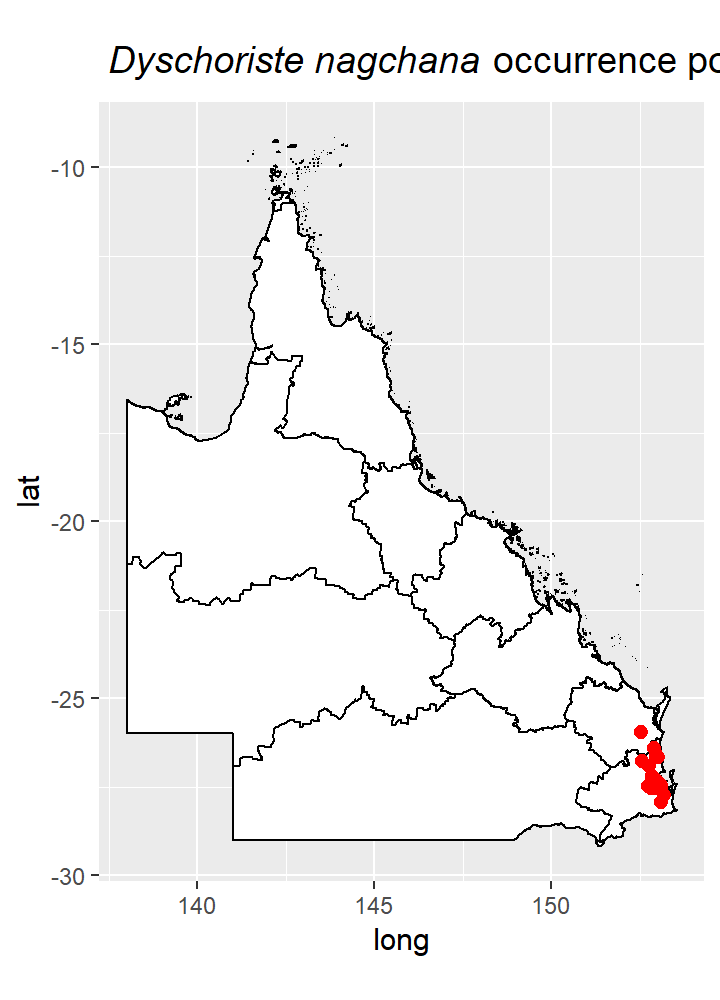

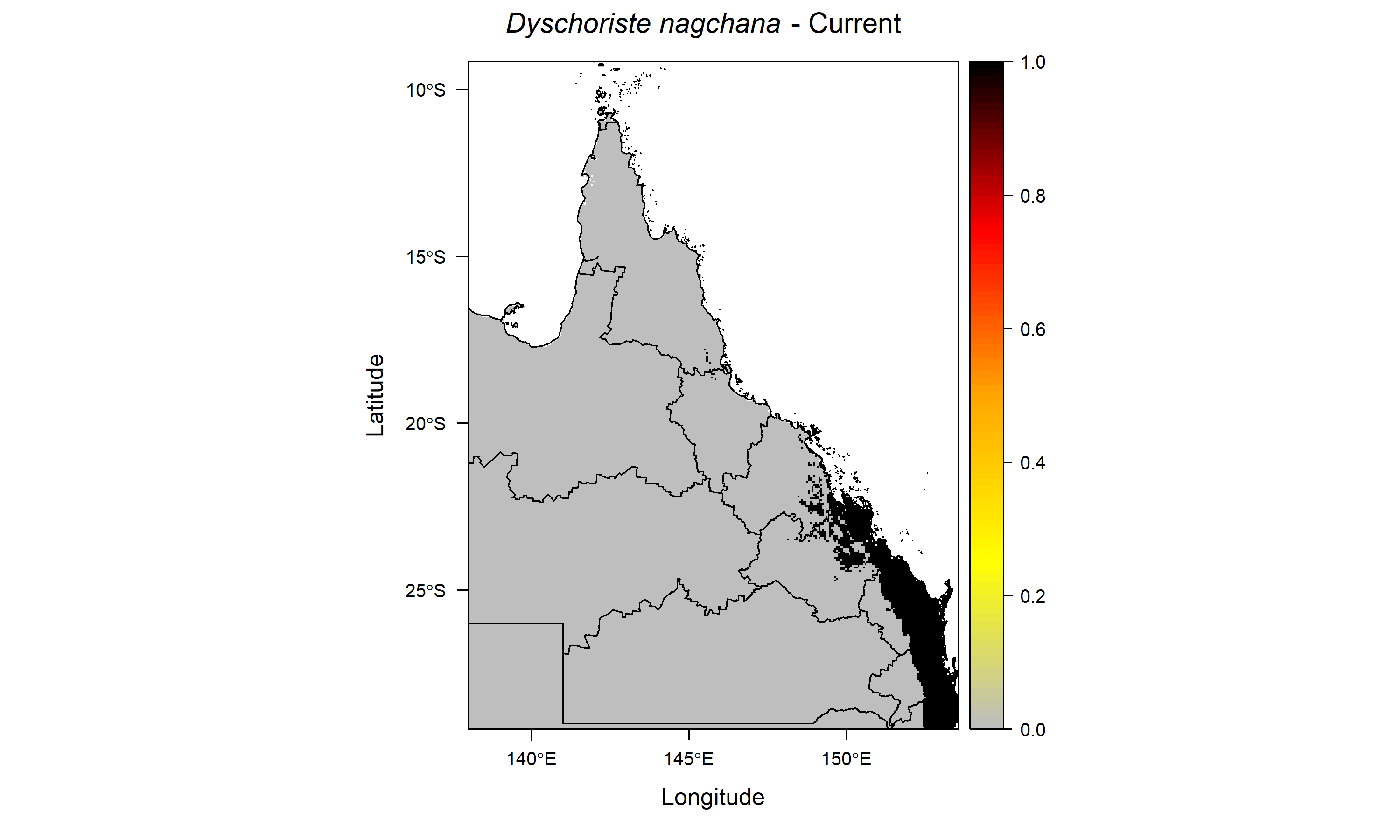

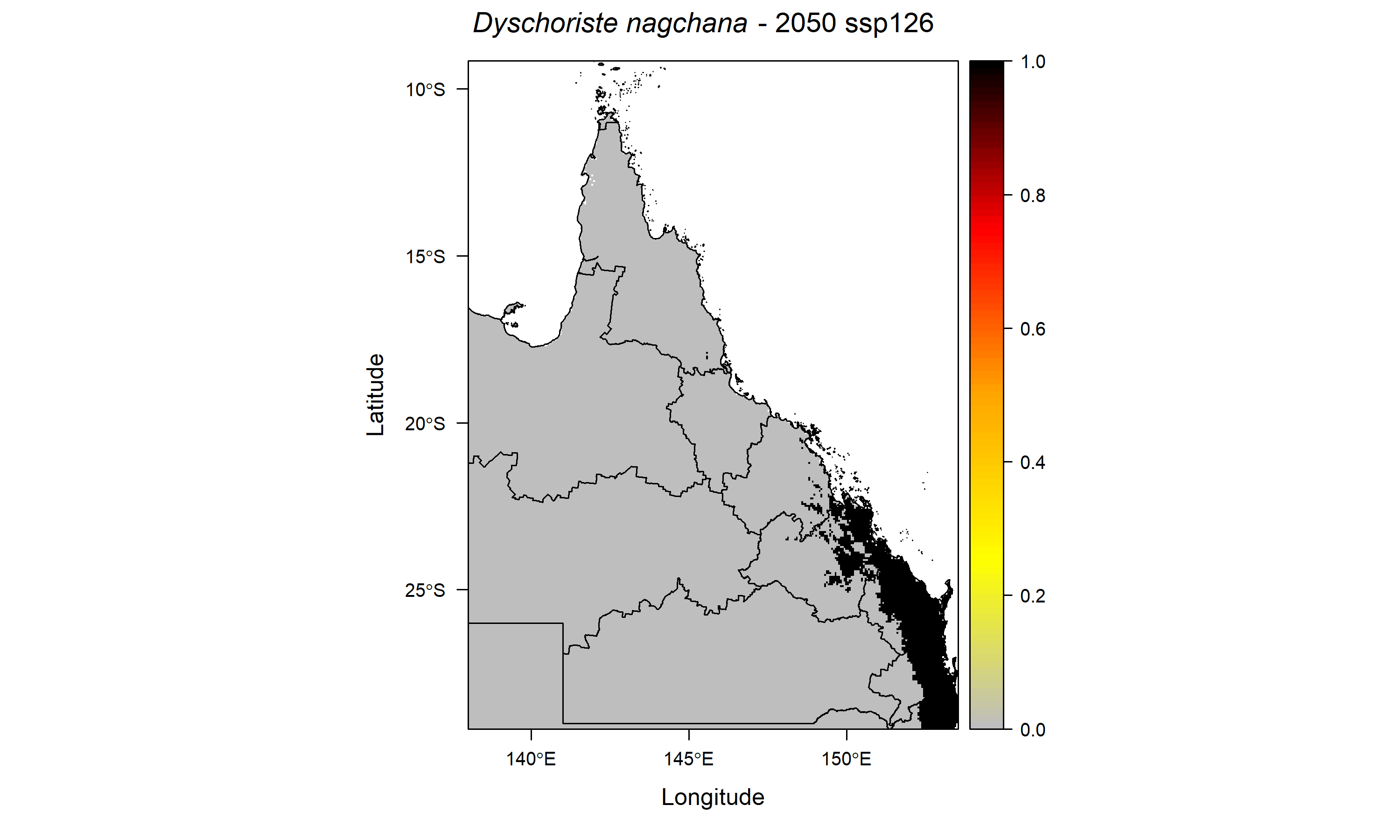

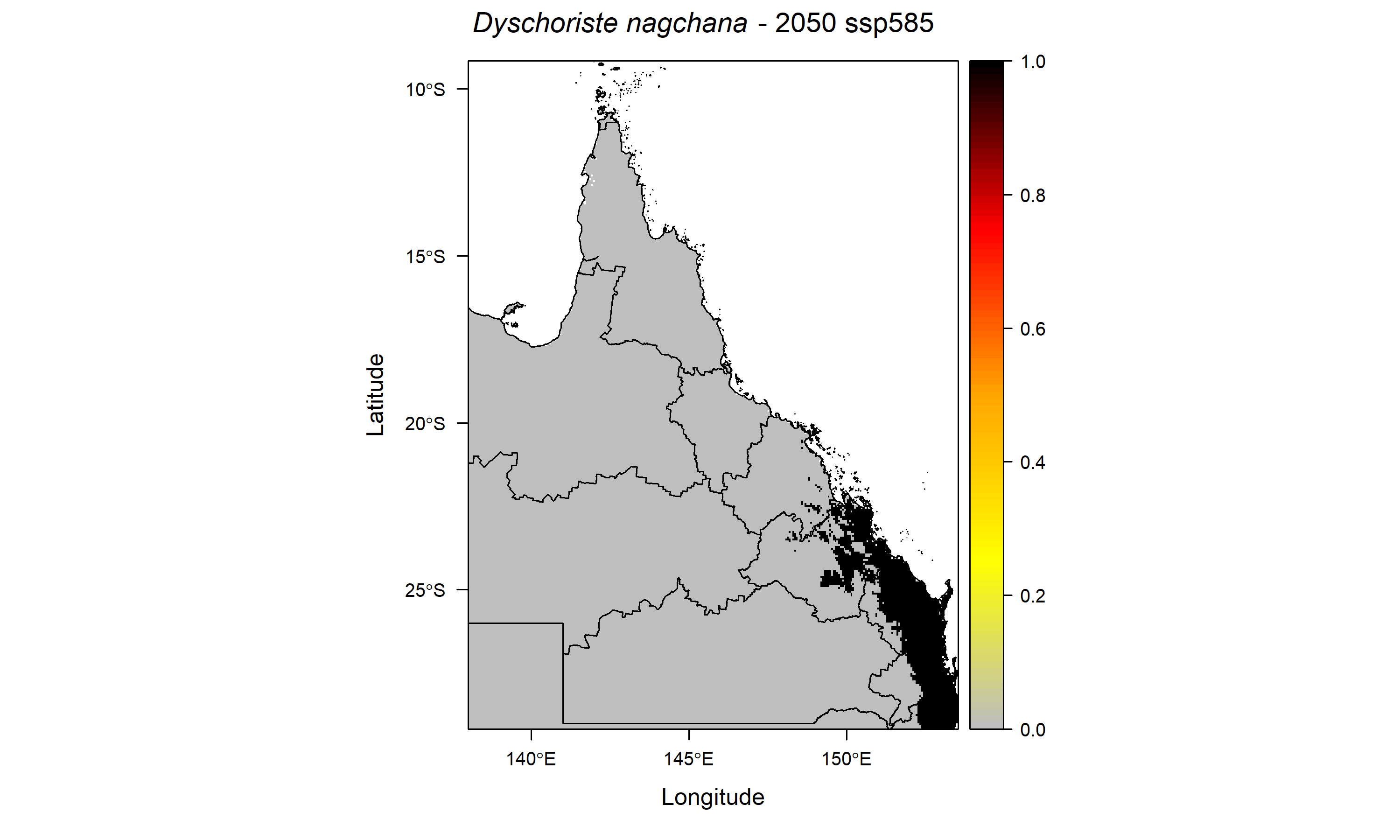

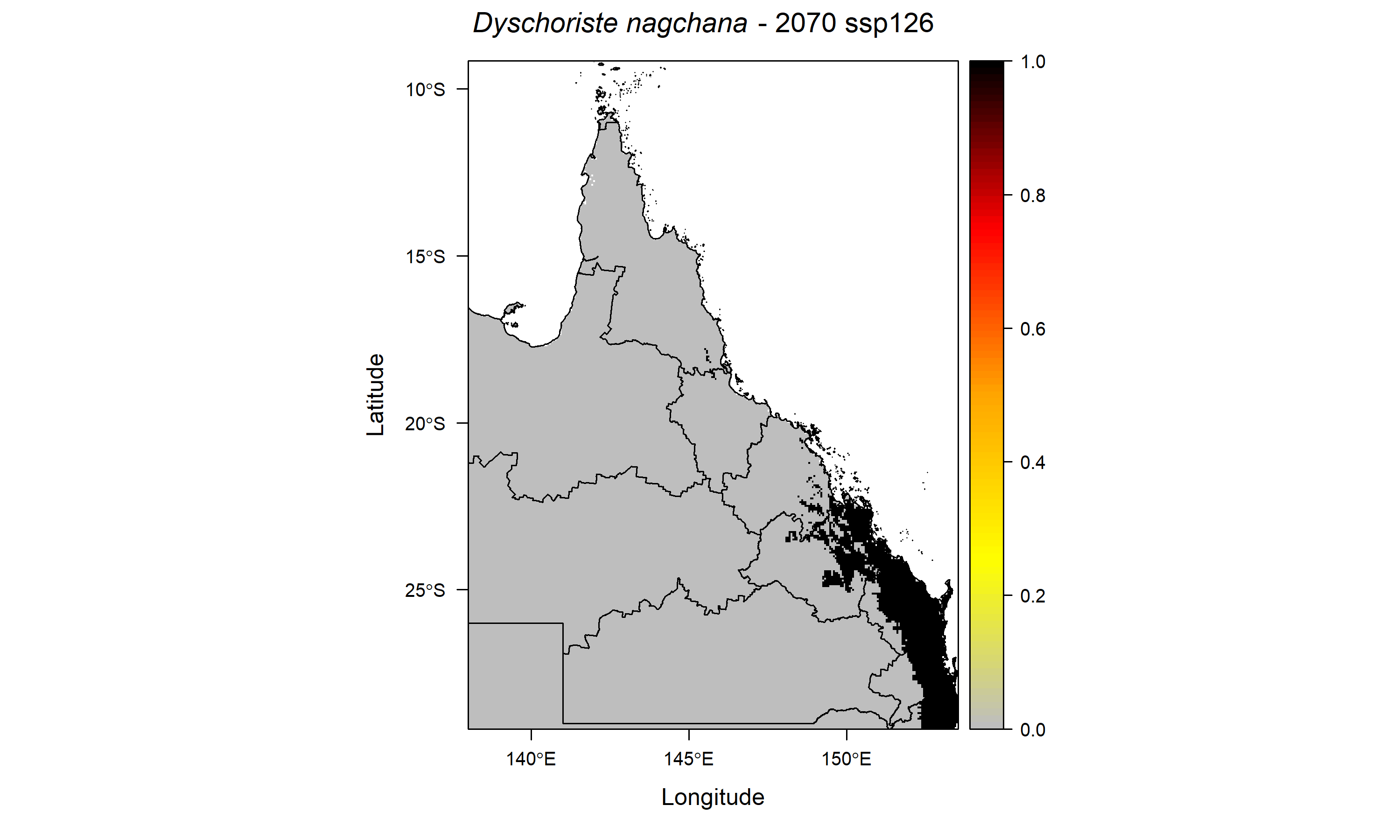

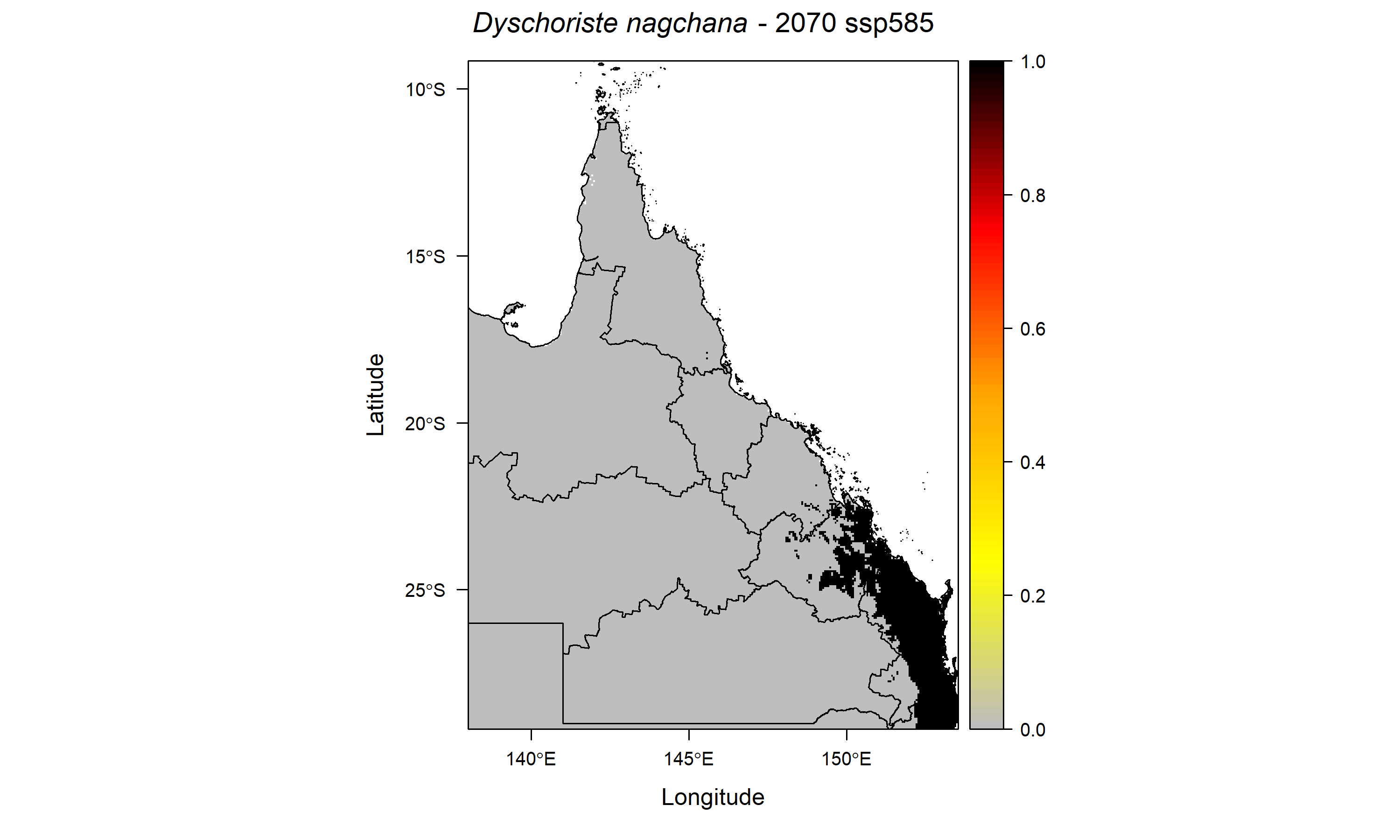


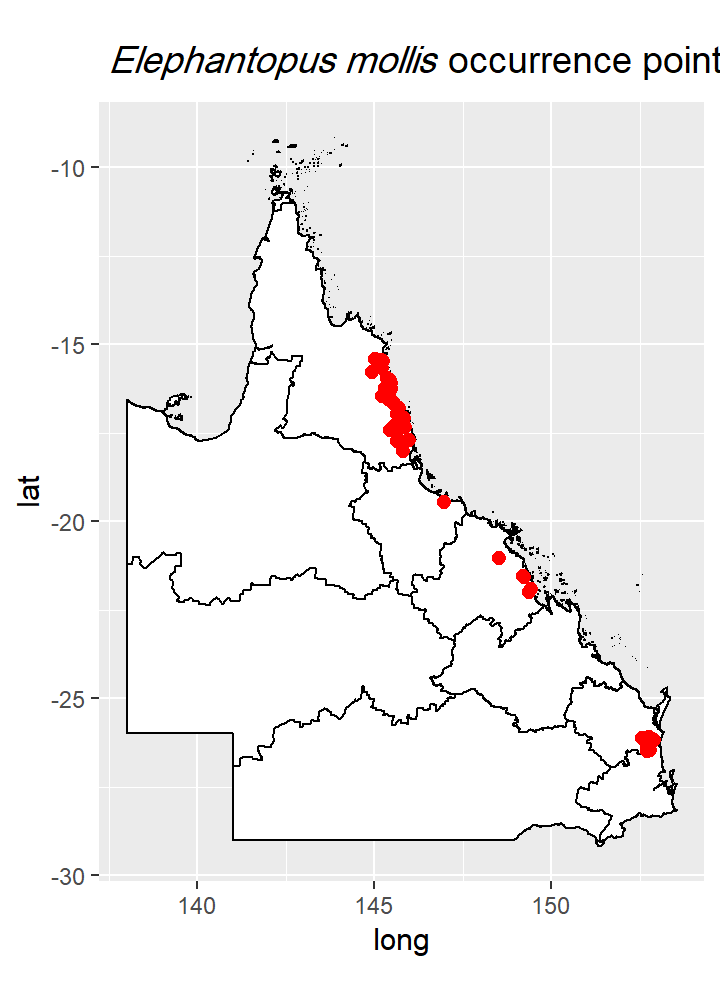

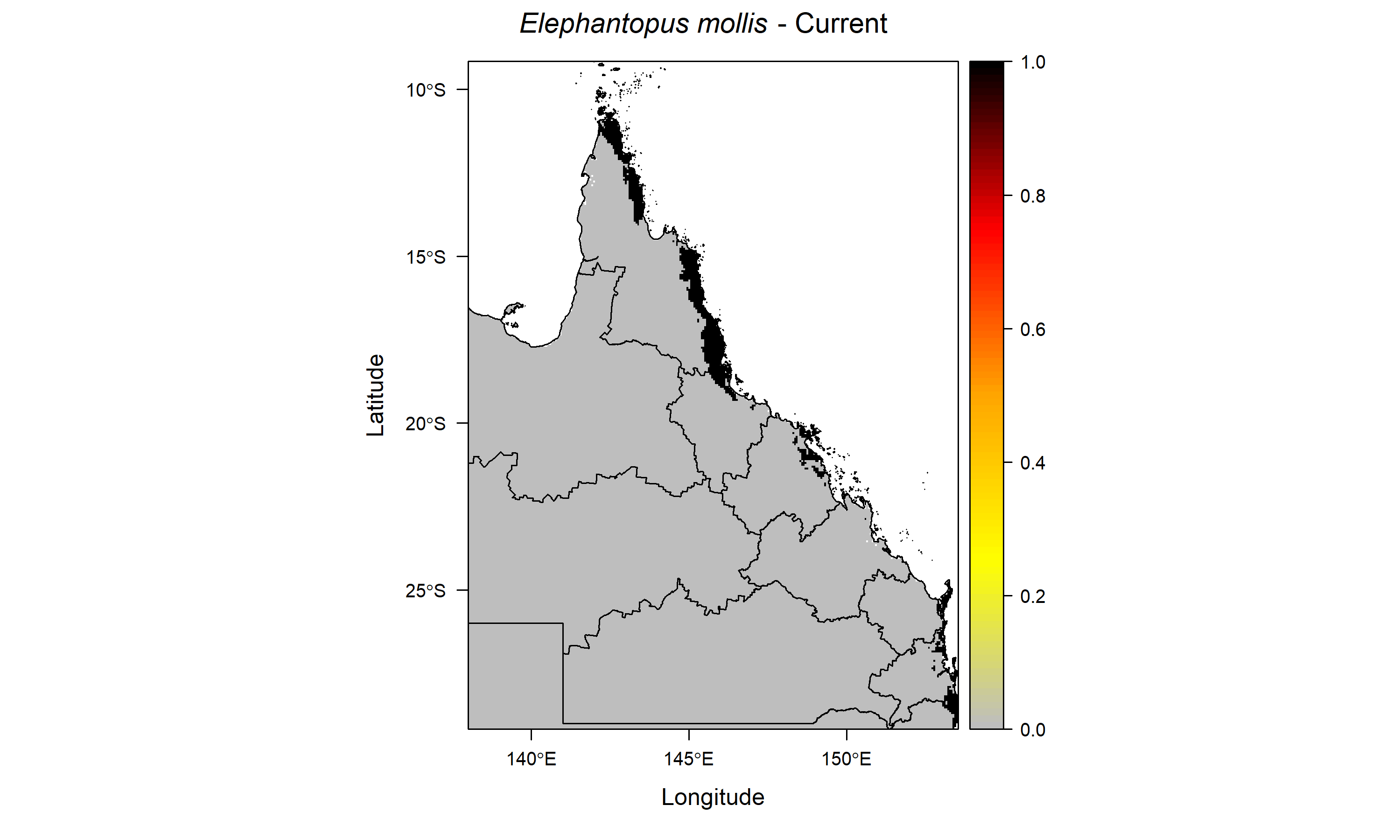

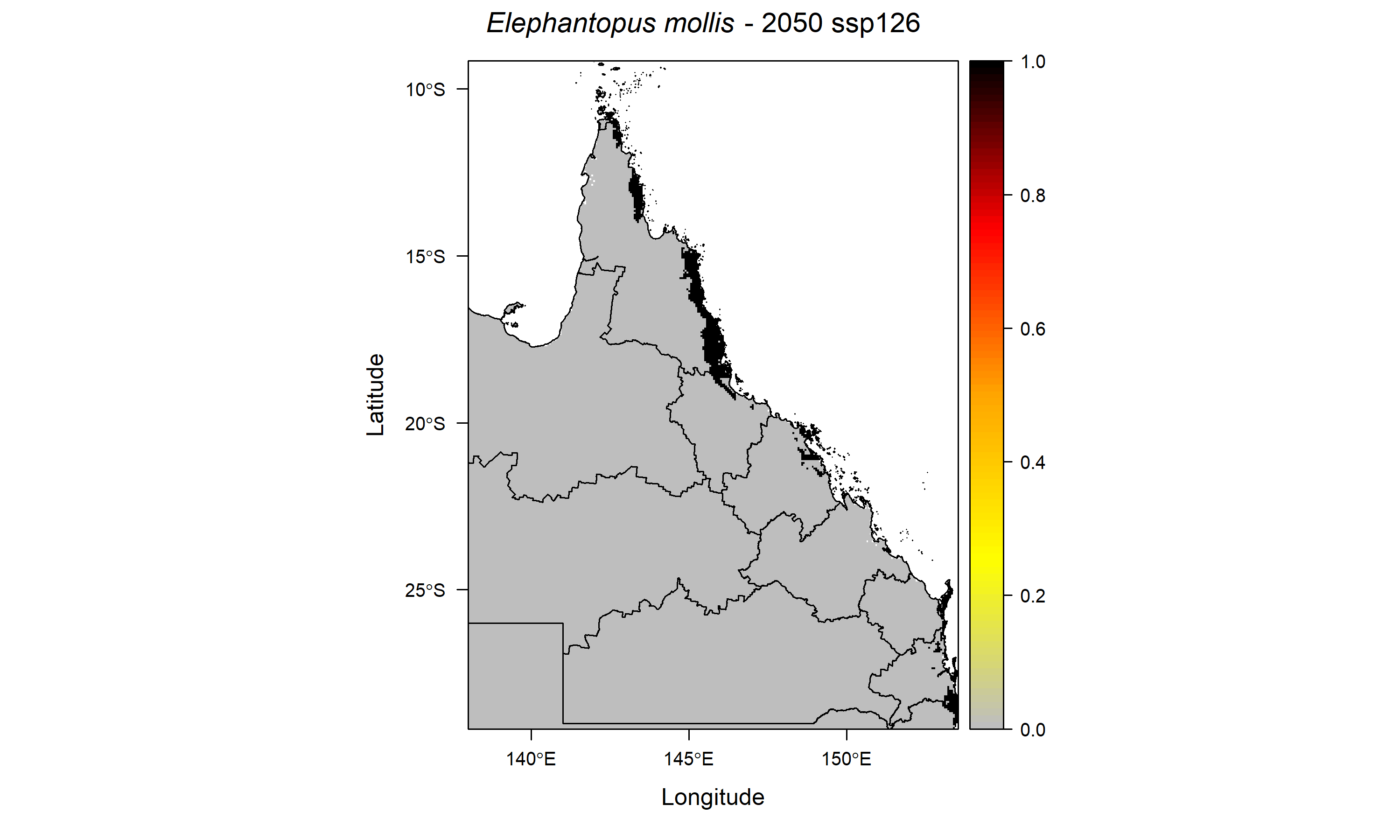

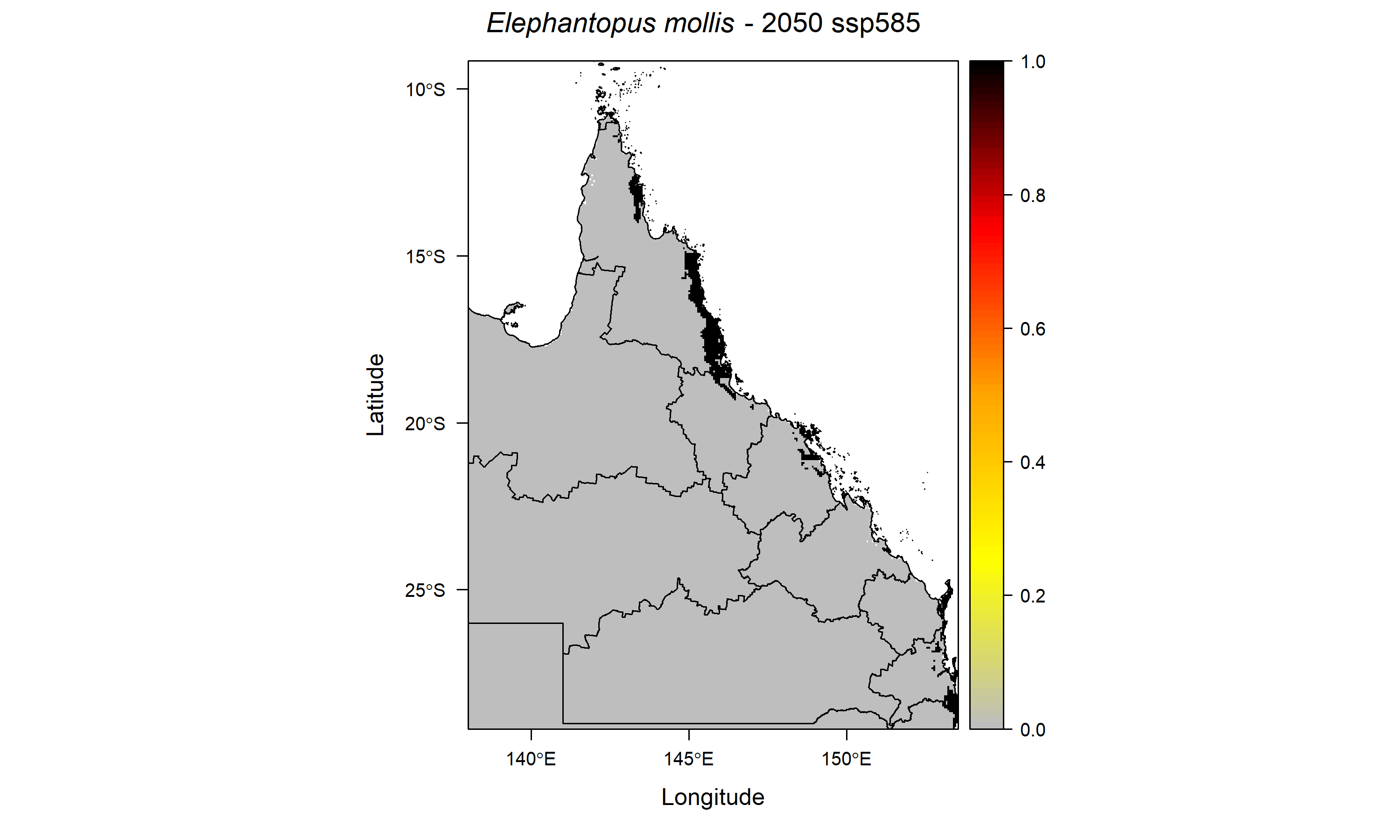

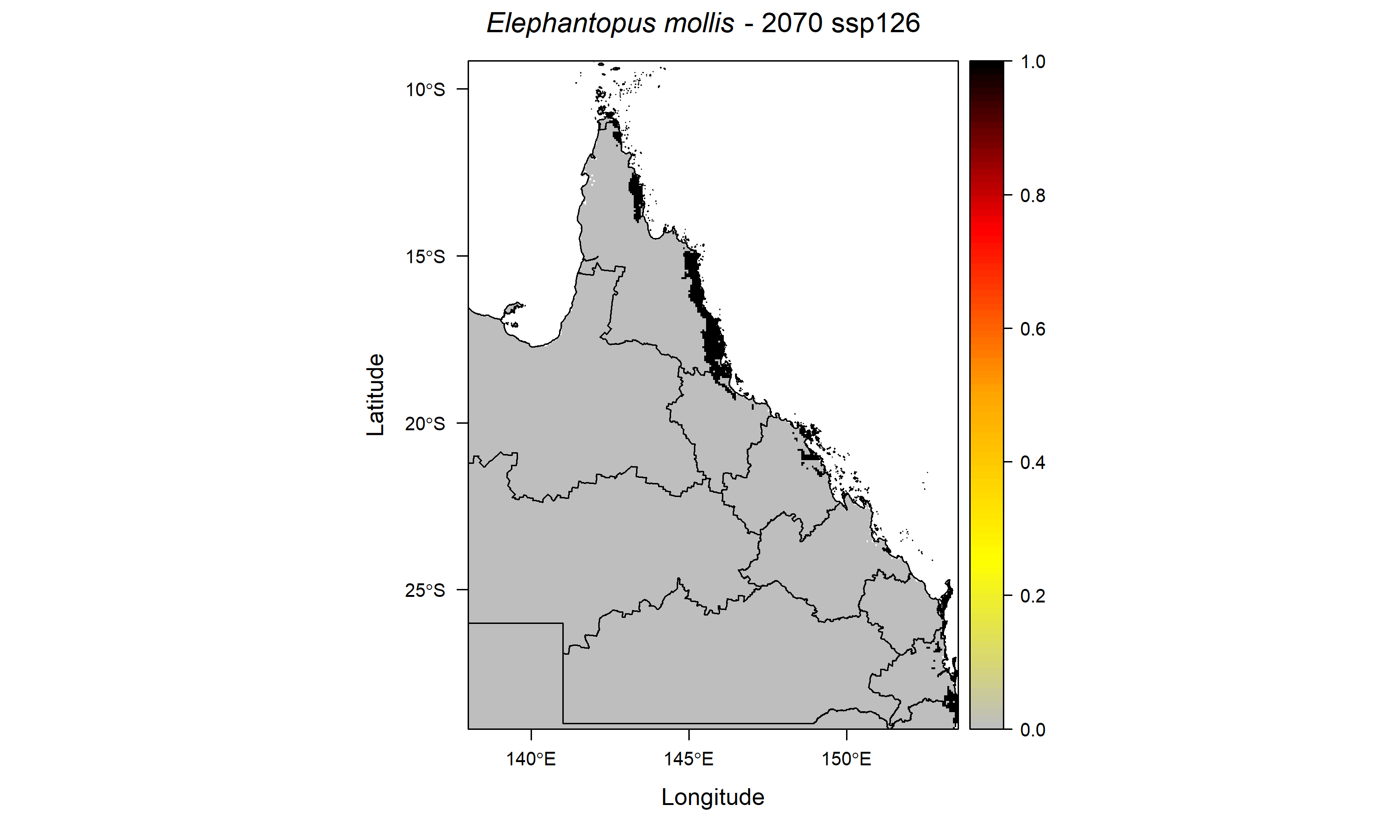

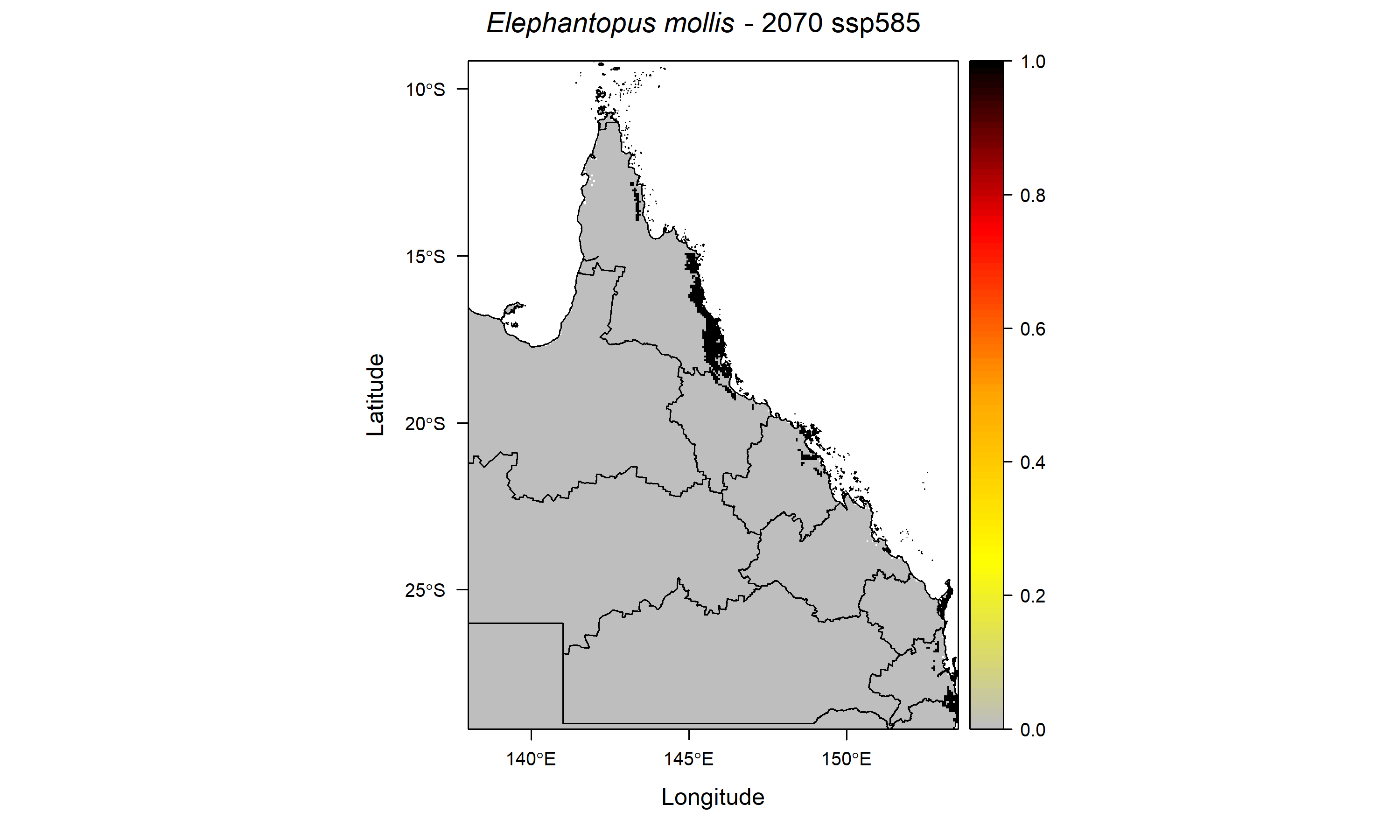


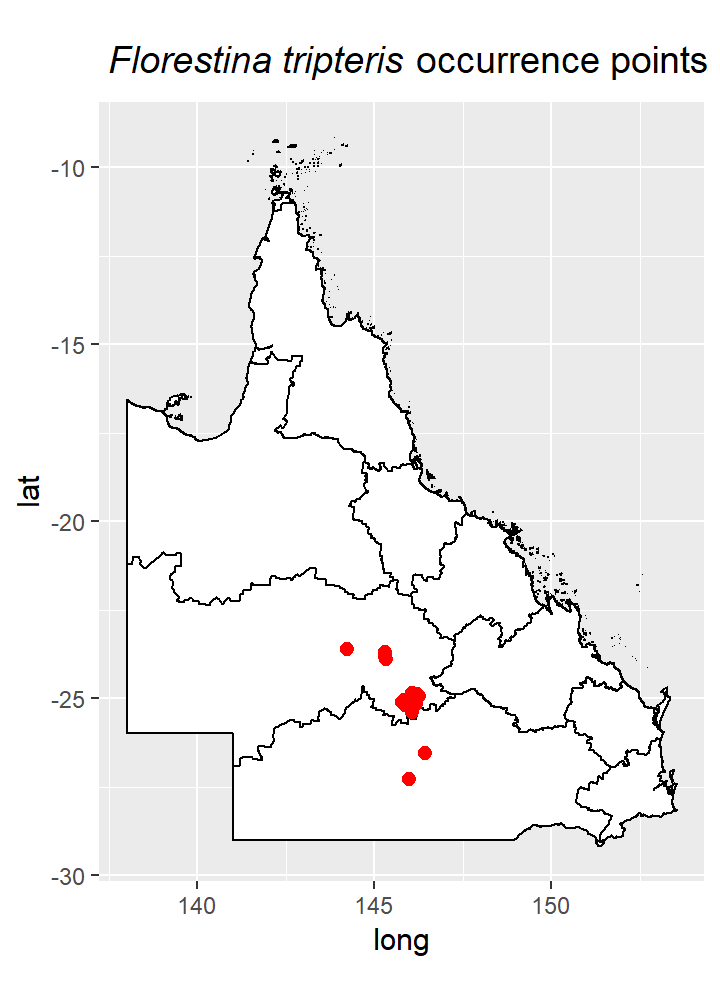

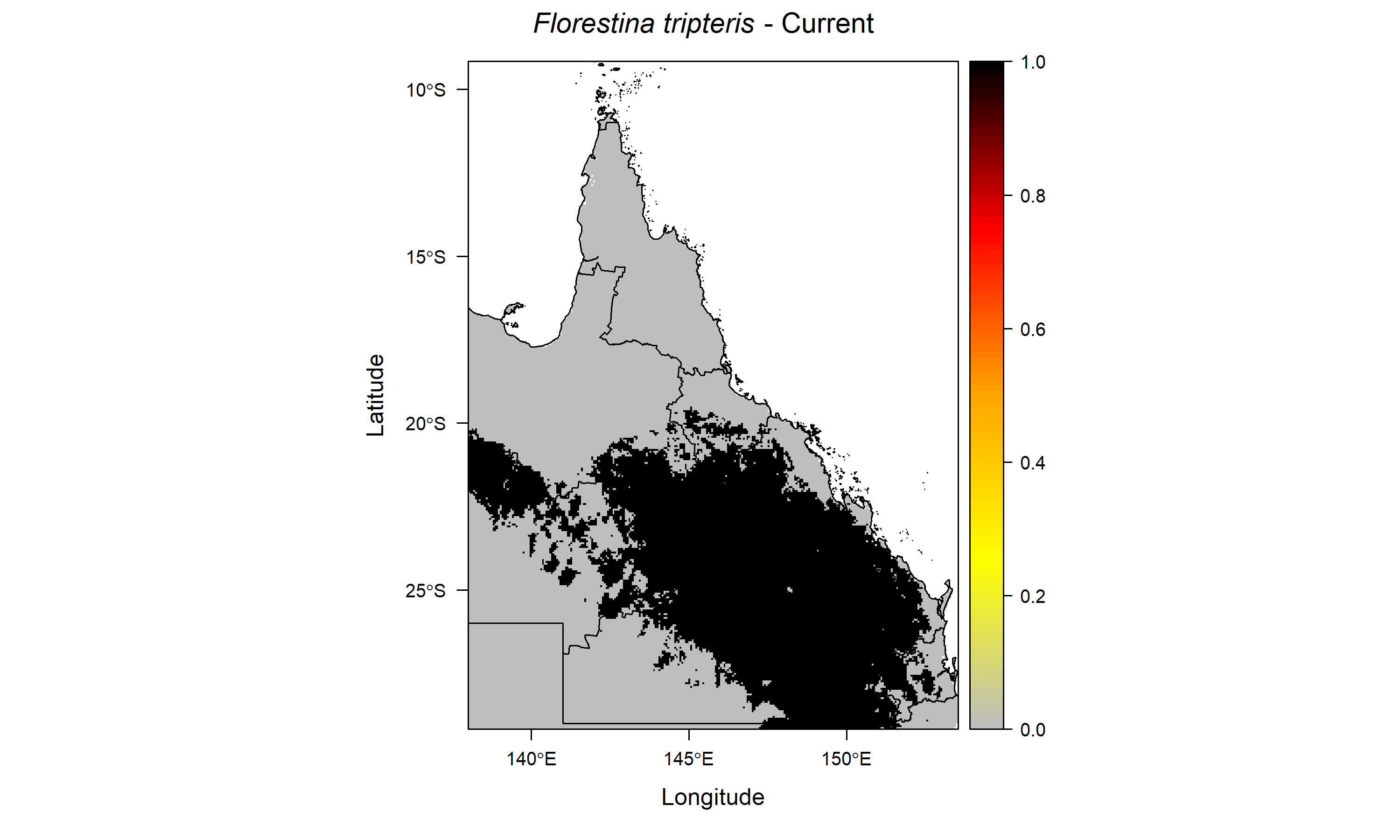

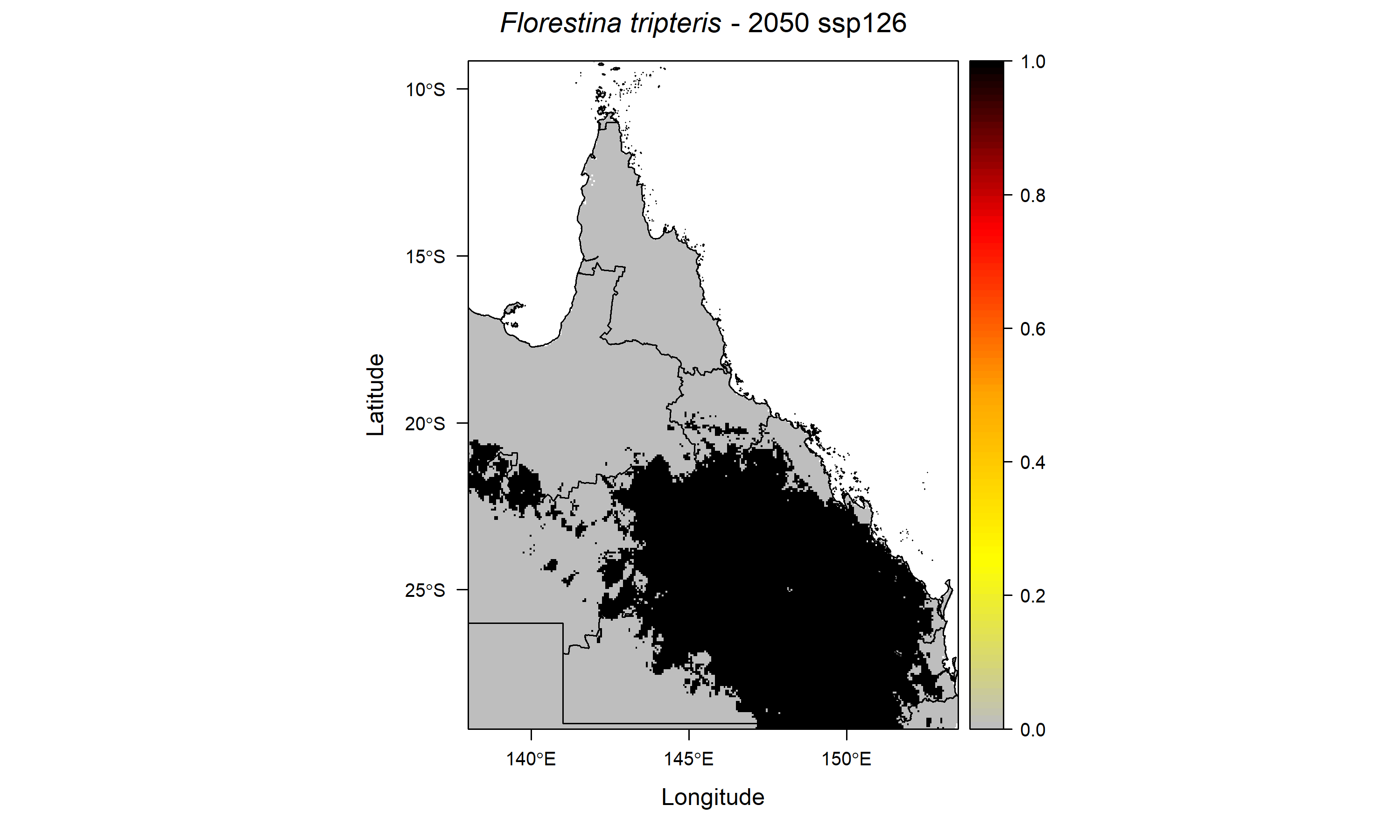

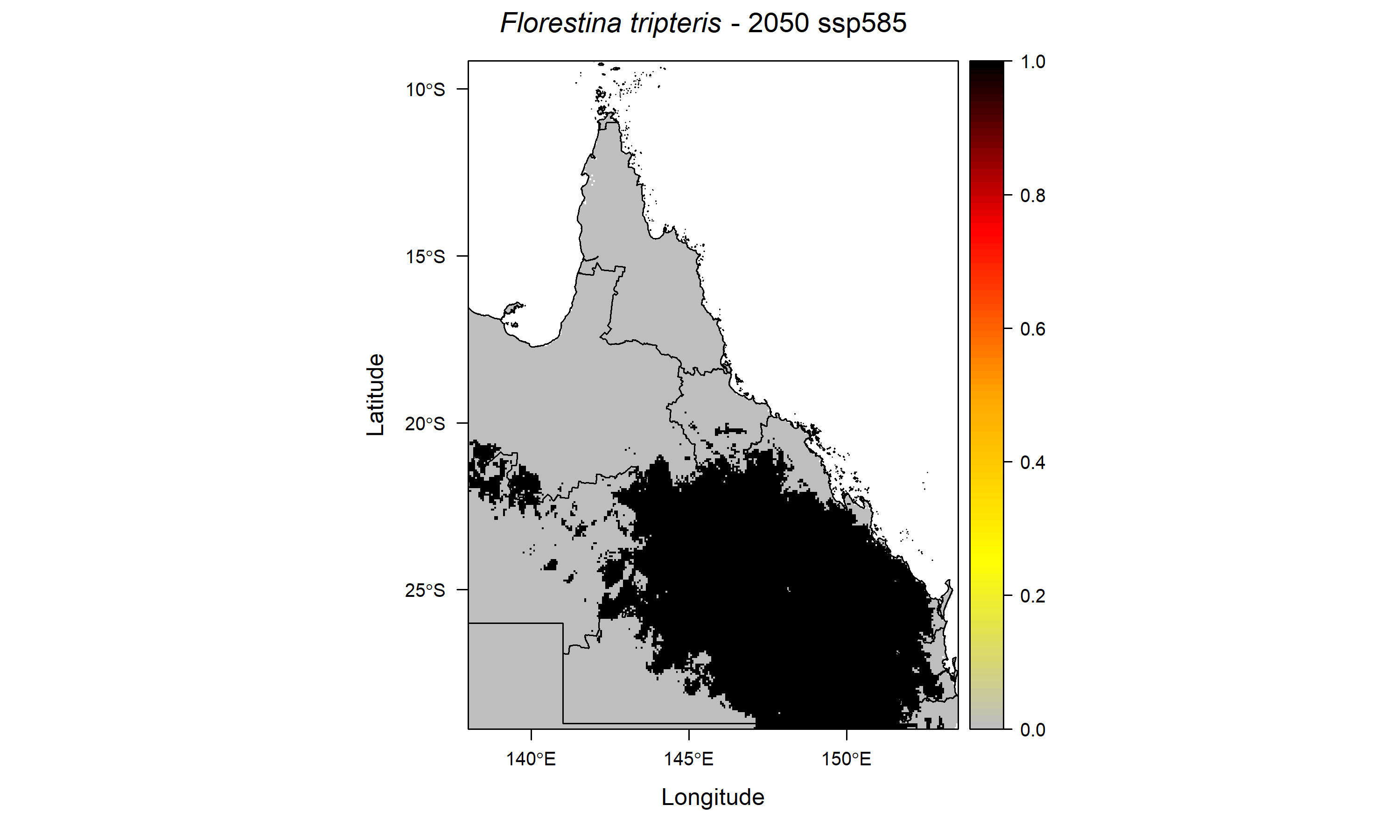

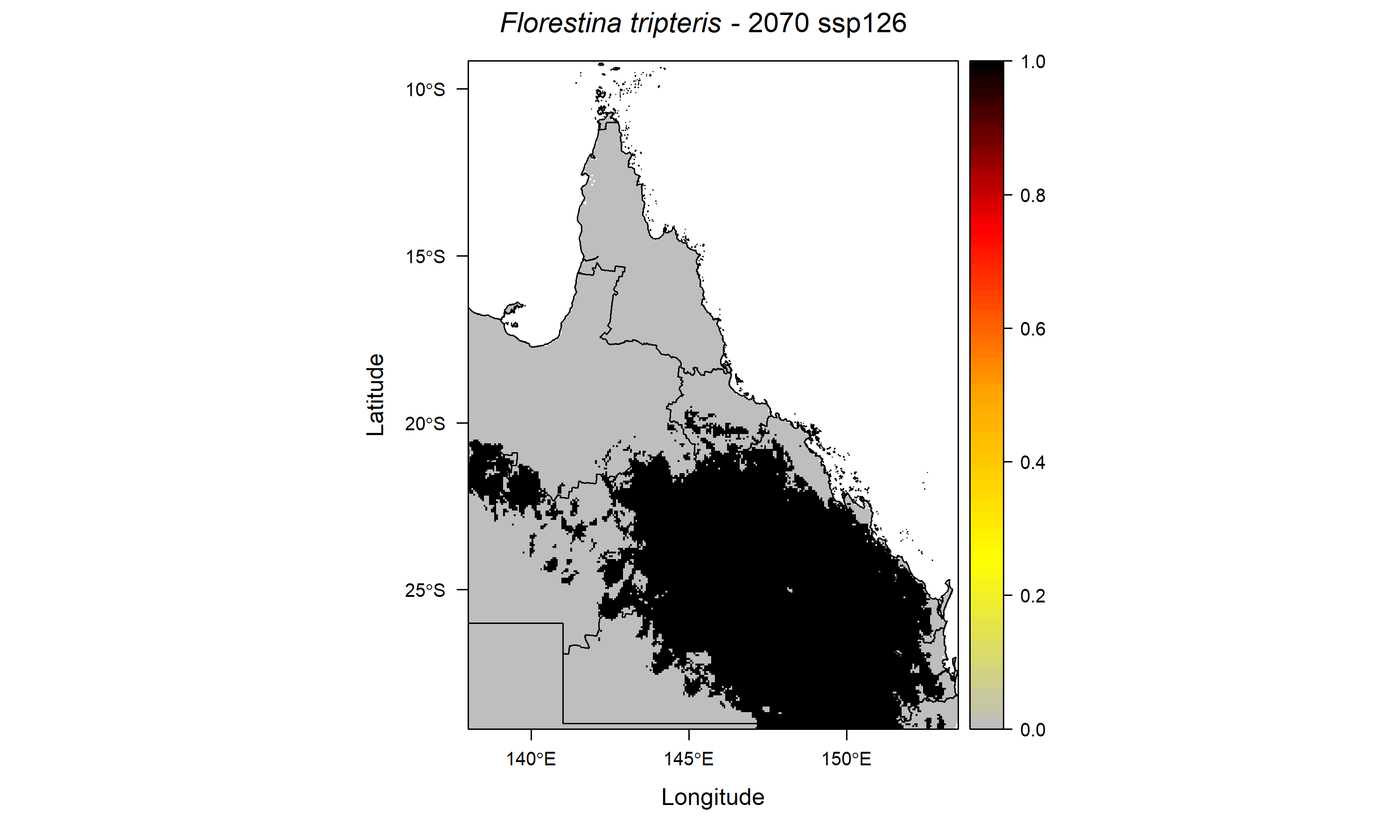

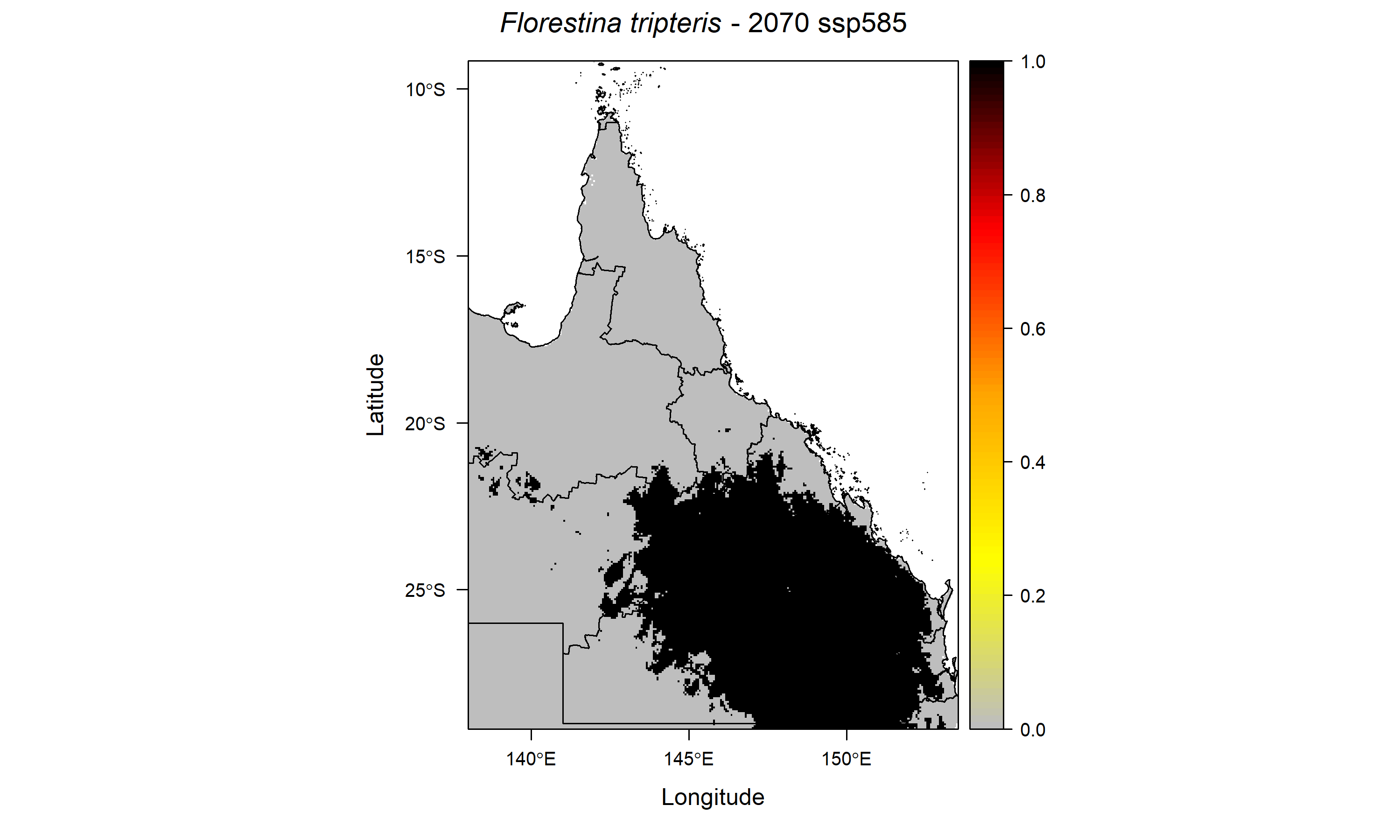

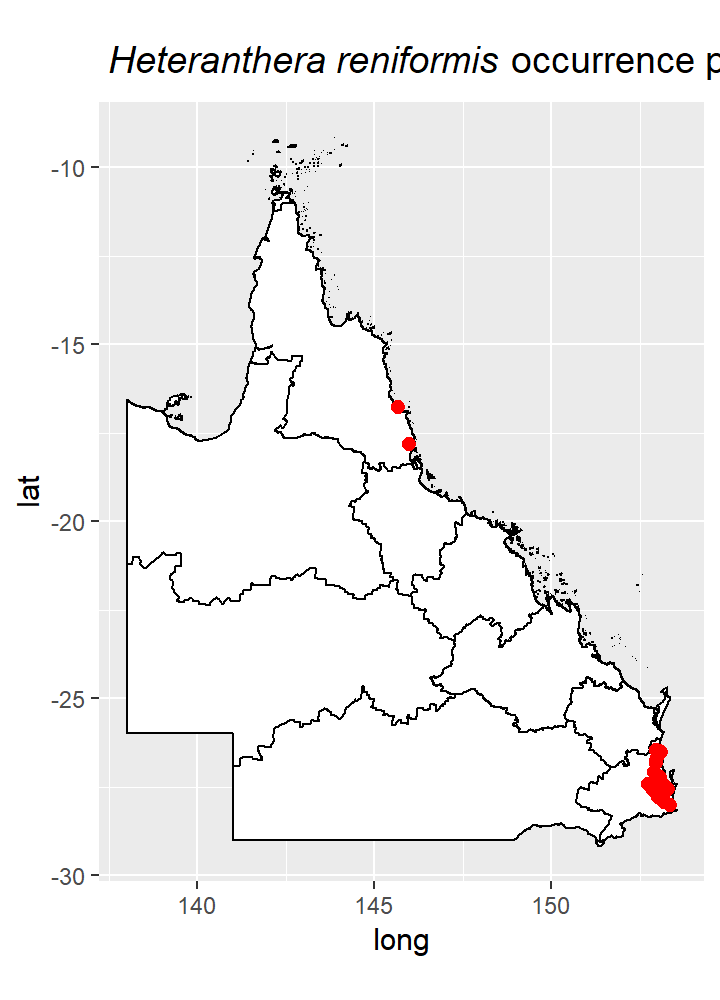

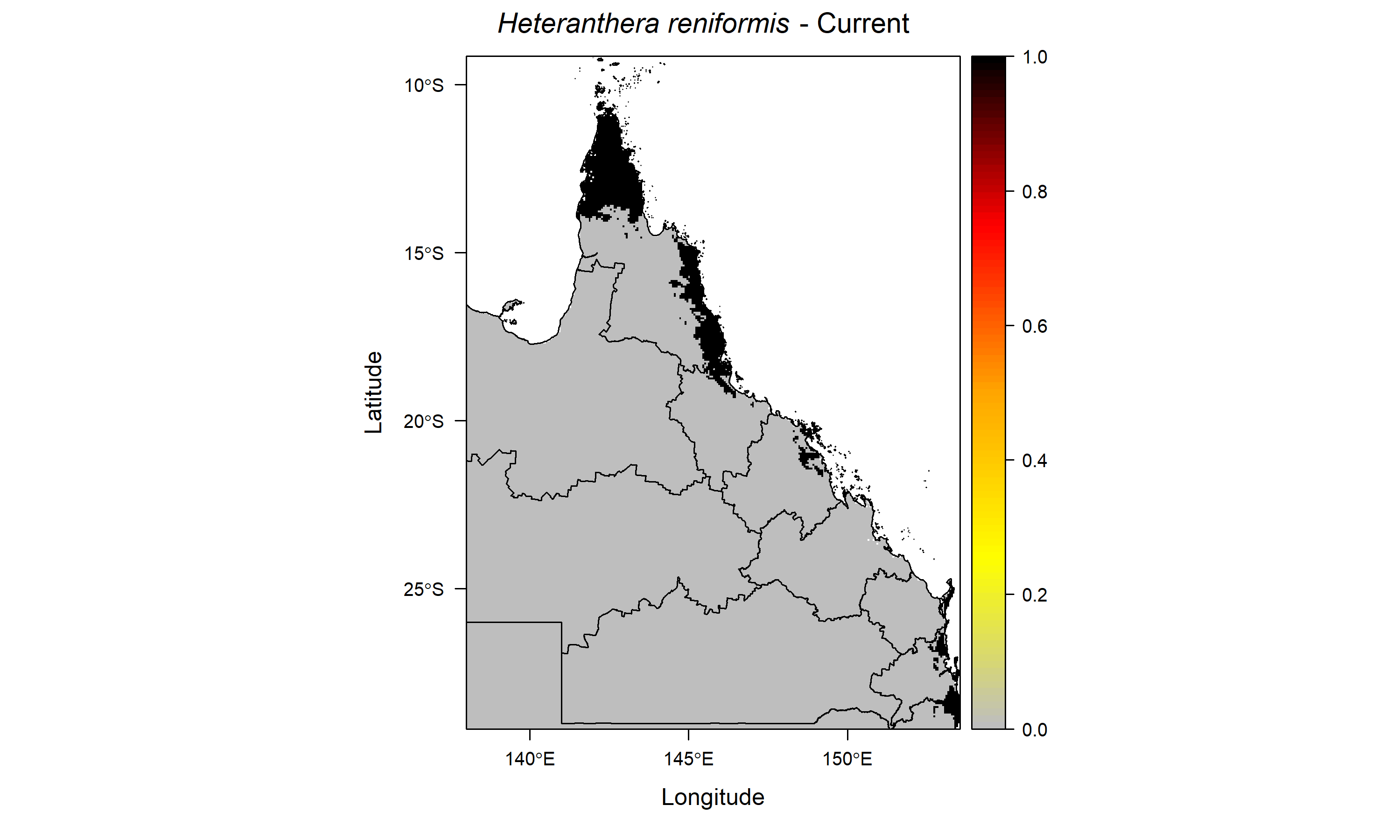

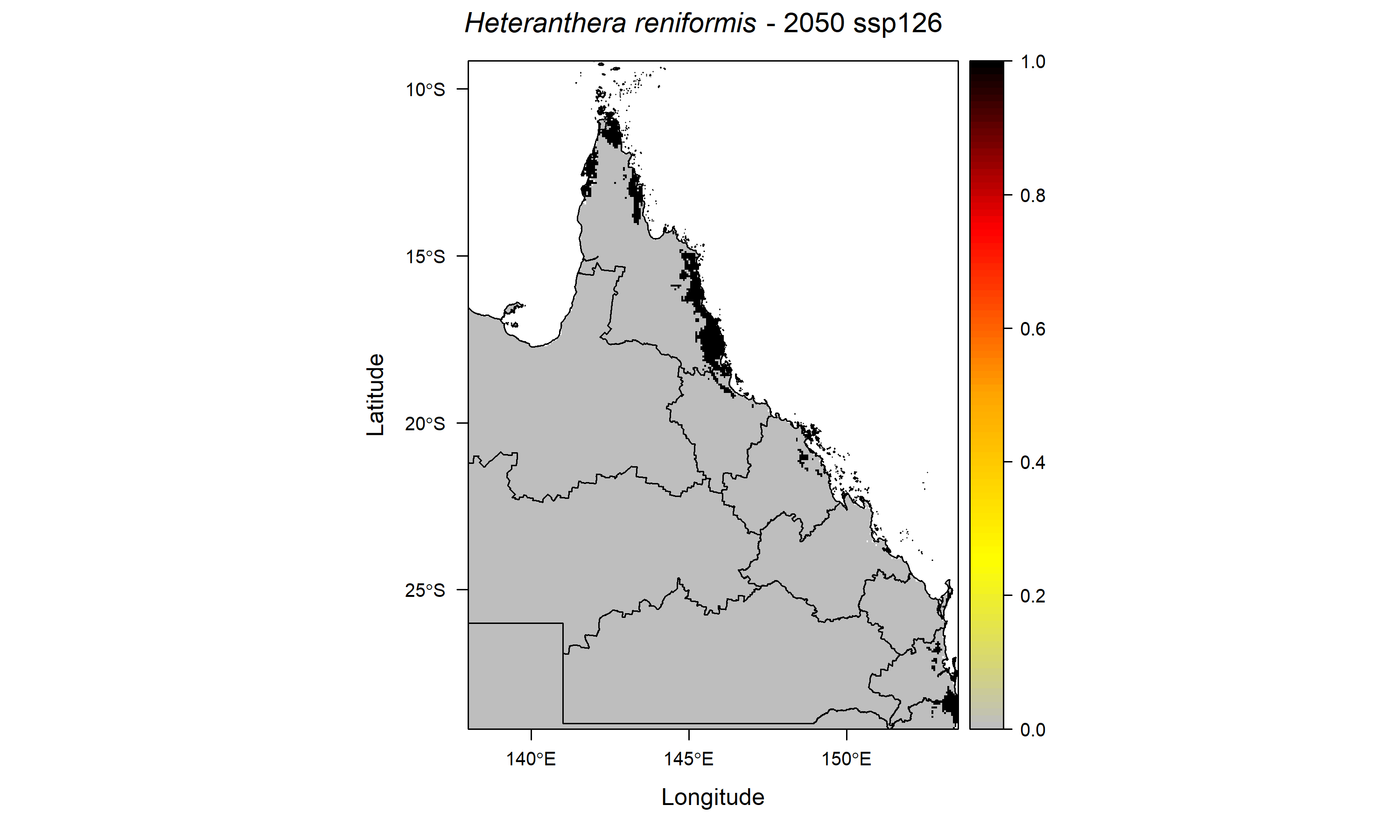

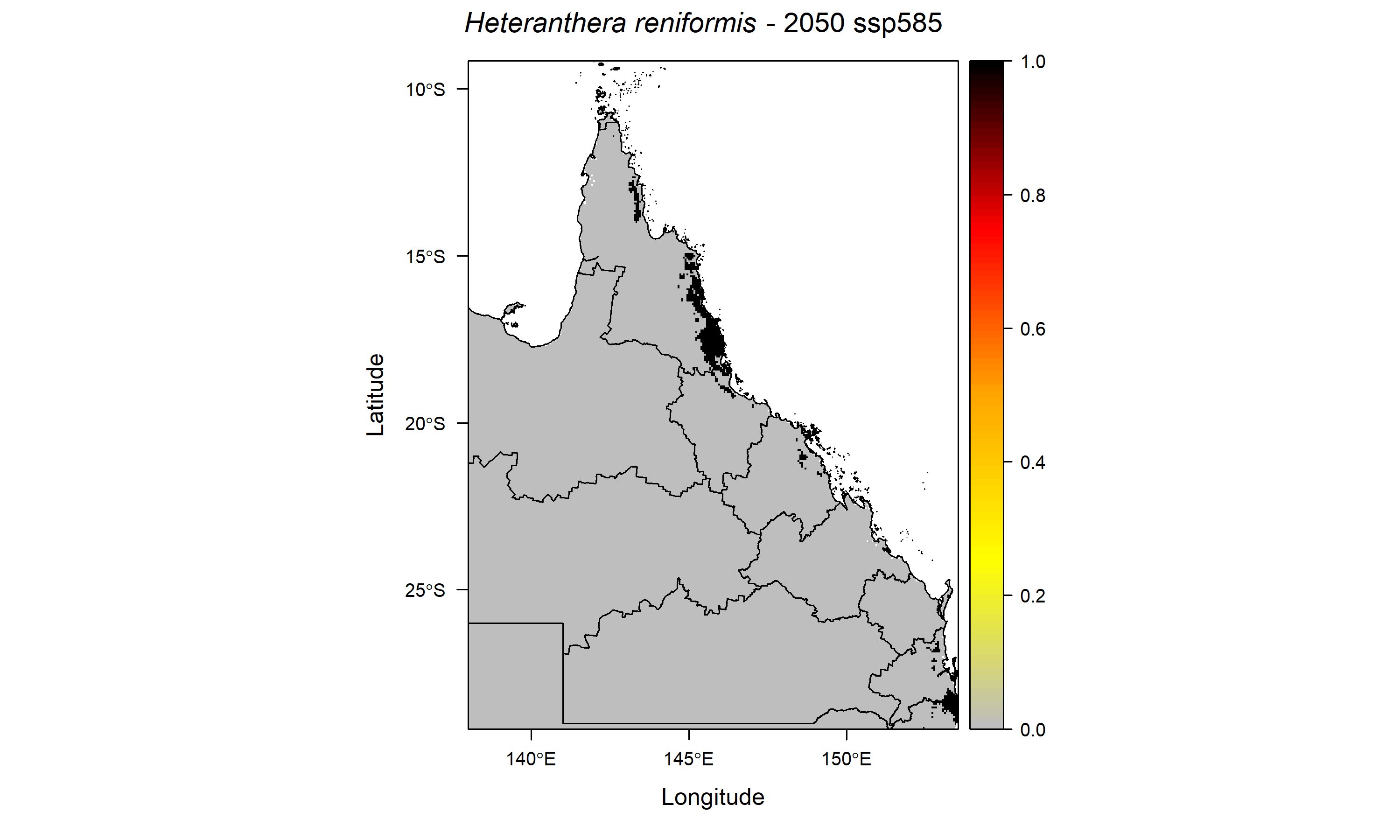

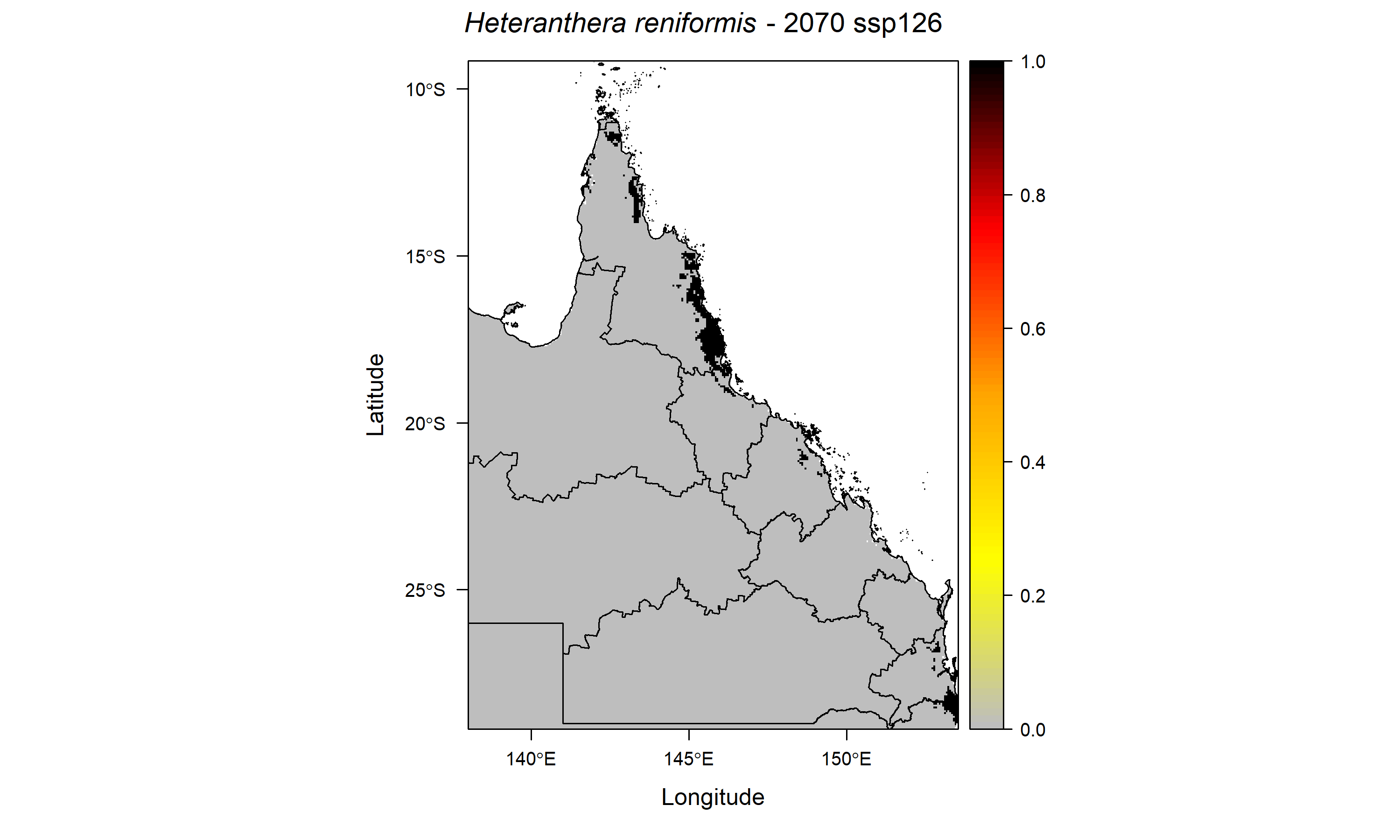

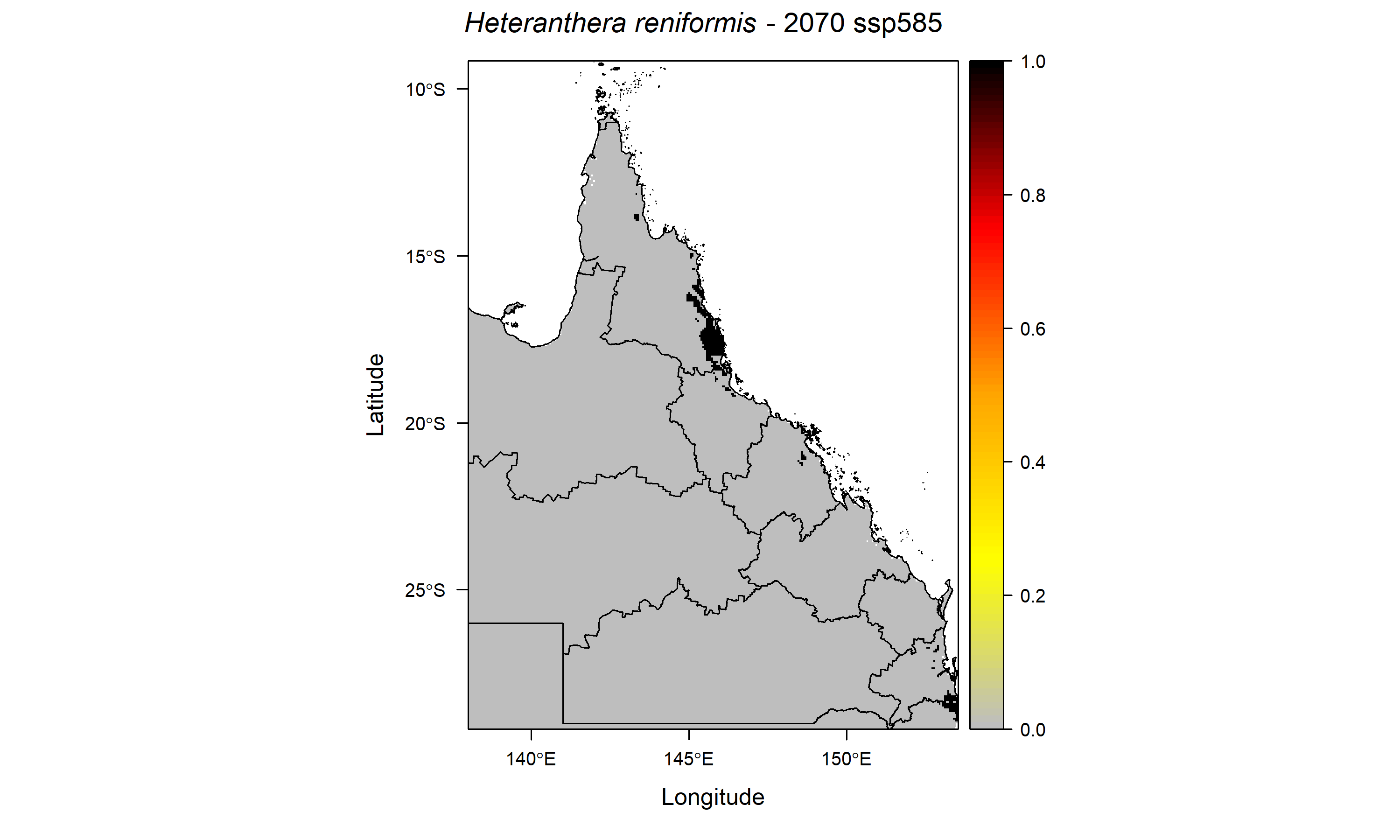


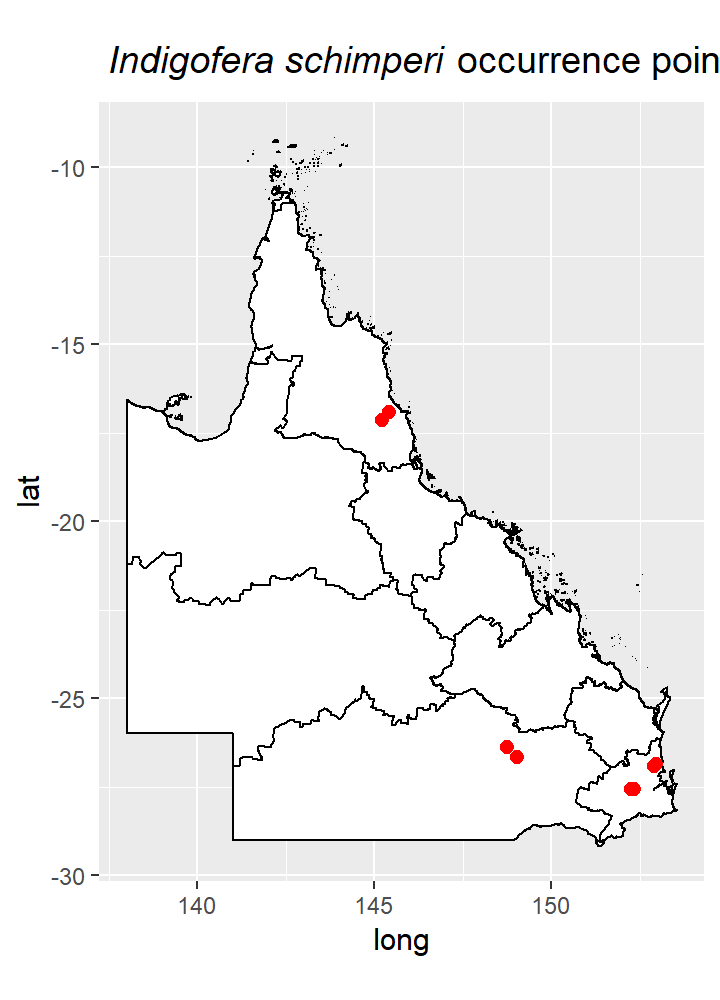

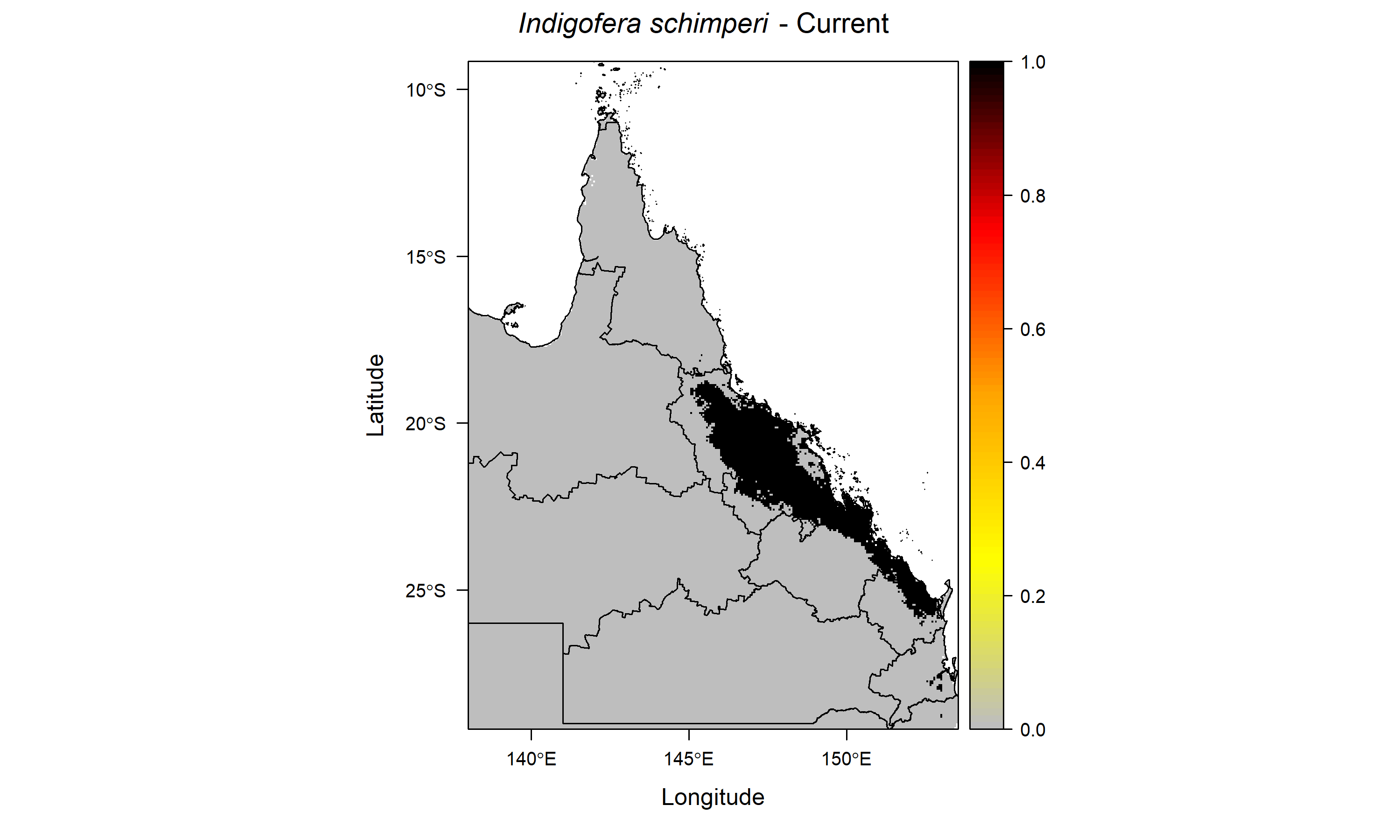

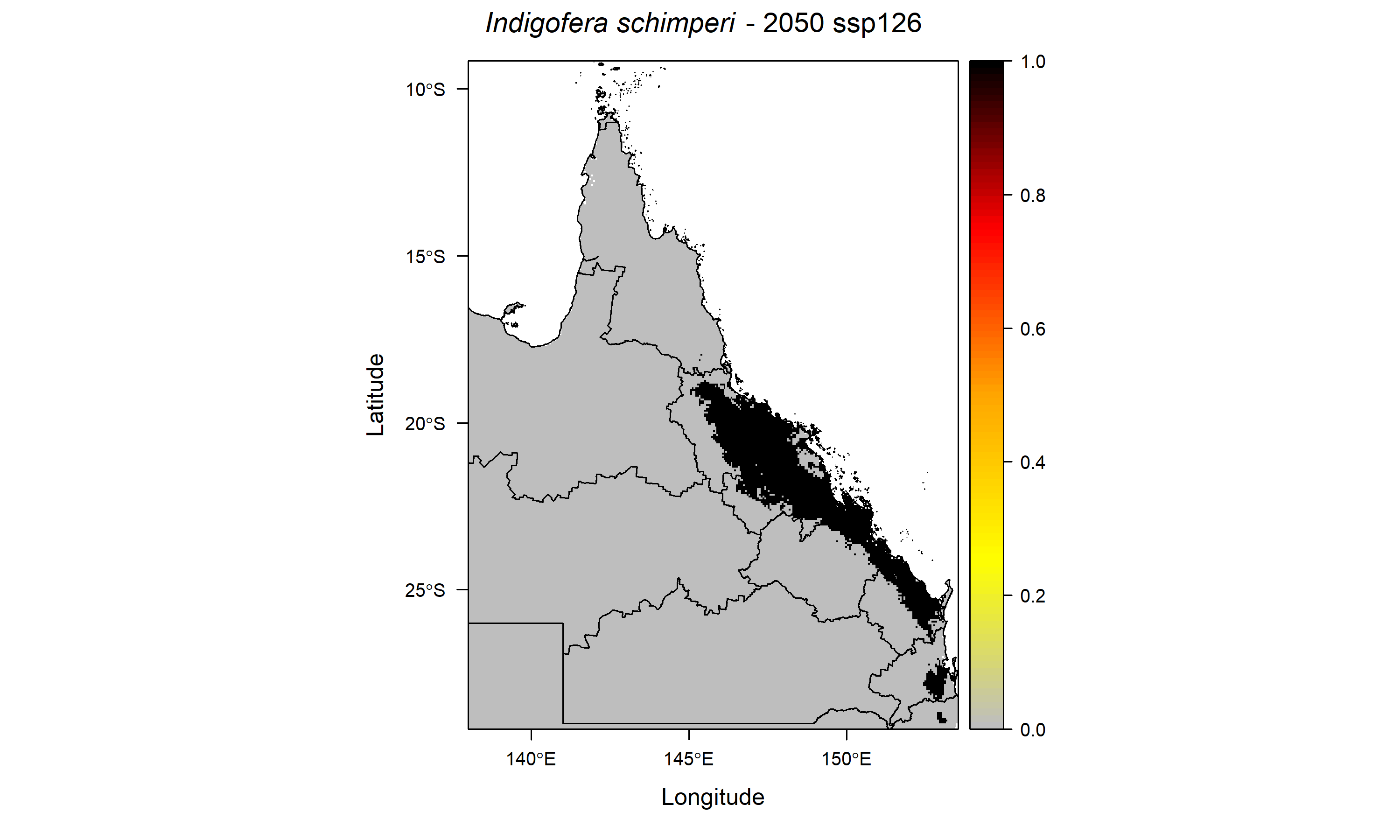

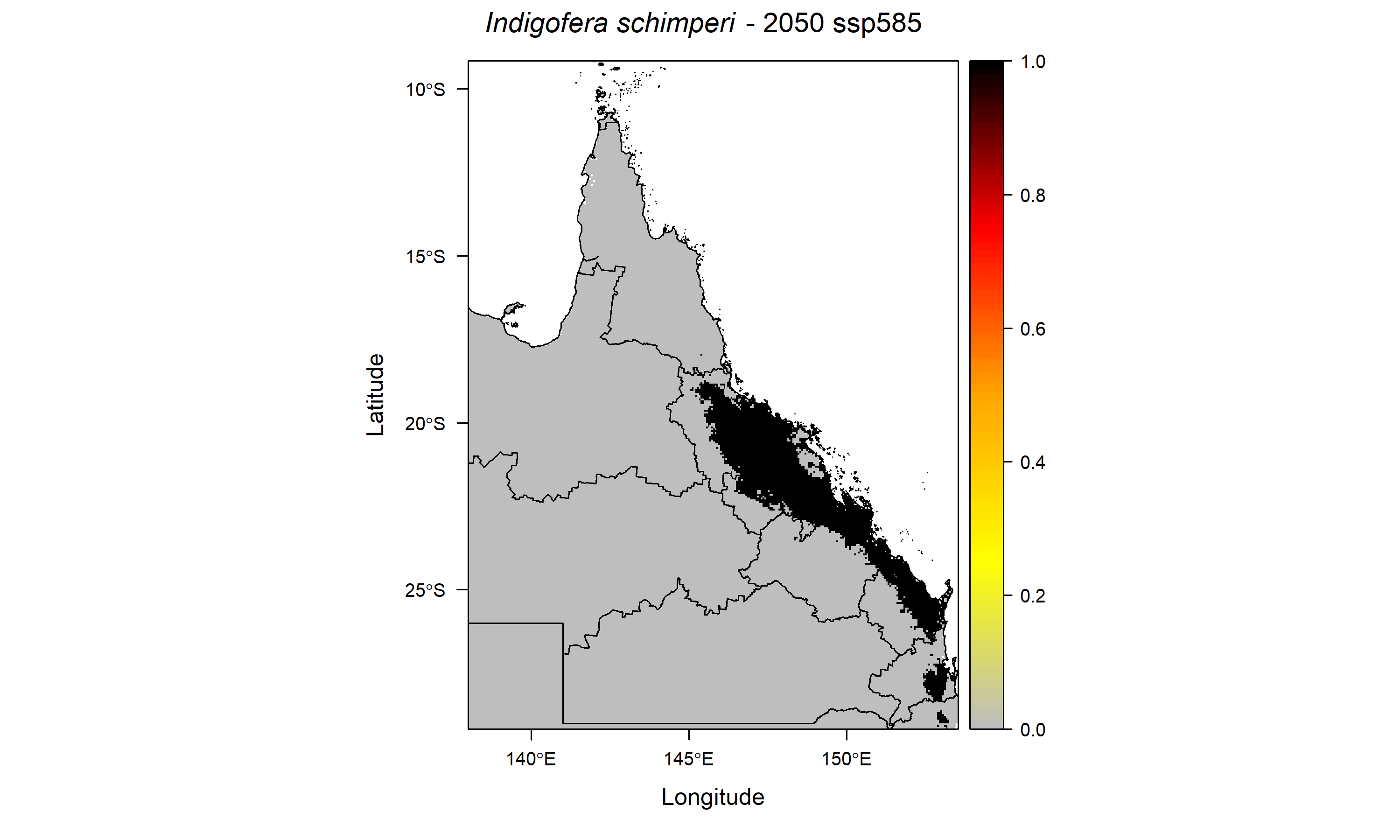


Appendix S2: Spatial distribution of 54 emerging weed species in QLD based on (i) actual occurrence points, (ii) potential range, (iii, iv) range response to climate change scenarios in 2050, and (v, vi) range response to climate change scenarios in 2070. Species are organised by plant growth forms – Grass (9)-Herb (14)-Shrub (8)-Tree (10)-Succulent (6)-Vine (7)

1. SHRUB- 8 species
2. (ii) (iii) (iv) (v) (vi)

Appendix S2: Spatial distribution of 54 emerging weed species in QLD based on (i) actual occurrence points, (ii) potential range, (iii, iv) range response to climate change scenarios in 2050 and (v, vi) range response to climate change scenarios in 2070. Species are organised by plant growth forms – Grass (9)-Herb (14)-Shrub (8)-Tree (10)-Succulent (6)-Vine (7)

SUCCULENT- 6 species.

1. (ii) (iii) (iv) (v) (vi)

Appendix S2: Spatial distribution of 54 emerging weed species in QLD based on (i) actual occurrence points, (ii) potential range, (iii, iv) response to climate change scenarios in 2050 and (v, vi) response to climate change scenarios in 2070. Species are organised by plant growth forms – Grass (9)-Herb (14)-Shrub (8)-Tree (10)-Succulent (6)-Vine (7)

TREE: 10 species (i) (ii) (iii) (iv) (v) (vi)

Appendix S2: Spatial distribution of 54 emerging weed species in QLD based on (i) actual occurrence points, (ii) potential range, (iii, iv) response to climate change scenarios in 2050 and (v, vi) response to climate change scenarios in 2070. Species are organised by plant growth forms – Grass (9)-Herb (14)-Shrub (8)-Tree (10)-Succulent (6)-Vine (7)

1. VINE- 7 species
2. (ii) (iii) (iv) (v) (vi)

|  | **Appendix S3**: Occurrence points, global number of training and test samples, true skill statistics (TSS), area under the curve (AUC) values, and relative contribution of eight chosen climatic variables influencing MaxEnt predictions of habitat suitability for 54 emerging weed species of QLD. The most influential climatic variables are indicated in heavy, underlined italic font. Bioclimatic abbreviations are explained in the texts of the manuscript. | | | | | | | | | | | | | | |
| --- | --- | --- | --- | --- | --- | --- | --- | --- | --- | --- | --- | --- | --- | --- | --- |
|  |  |  |  |  |  |  |  |  |  |  |  |  |  |  |  |
|  |  |  |  |  |  |  |  | Climatic variable contribution (%) | | | | | | | |
| No | Species | Life form | No of occurrence points in QLD | No. of occurrence points used in MaxEnt | TSS | AUC |  | bio1 | bio12 | bio13 | bio14 | bio15 | bio4 | bio5 | bio6 |
| 1 | *Acaciella glauca* | Tree | 14 | 23 | 0.723 | 0.921 |  | 15.20 | 12.49 | 2.12 | 2.23 | 0.43 | ***35.98*** | 26.15 | 5.40 |
| 2 | *Acanthospermum australe* | Herb | 4 | 843 | 0.622 | 0.856 |  | 12.39 | 1.62 | 0.22 | 14.68 | 9.98 | ***39.24*** | 4.99 | 16.88 |
| 3 | *Amphilophium crucigerum* syn *Pithecoctenium crucigerum* | Vine | 12 | 720 | 0.505 | 0.814 |  | 2.47 | 2.53 | 9.35 | 2.92 | 8.82 | ***61.34*** | 1.27 | 11.32 |
| 4 | *Artemisia verlotiorum* | Herb | 0 | 34 | 0.406 | 0.740 |  | 10.88 | 2.88 | 2.33 | ***30.96*** | 4.49 | 2.72 | 13.44 | ***32.30*** |
| 5 | *Arundo donax* | Grass | 47 | 7996 | 0.658 | 0.817 |  | 4.09 | 2.29 | 0.01 | 1.09 | 4.36 | 11.18 | 4.26 | ***72.72*** |
| 6 | *Asparagus retrofractus* | Vine | 2 | 148 | 0.75 | 0.927 |  | 7.73 | 4.19 | 13.77 | 2.44 | 16.23 | ***46.18*** | 1.11 | 8.34 |
| 7 | *Barleria repens* | Shrub | 54 | 239 | 0.782 | 0.938 |  | 4.67 | 6.82 | 0.59 | 2.83 | 27.27 | ***30.26*** | 13.99 | 13.58 |
| 8 | *Bignonia magnifica* syn *Saritae magnifica* | Vine | 17 | 55 | 0.639 | 0.879 |  | 4.98 | 6.24 | 5.35 | 10.96 | 8.70 | 22.28 | ***31.83*** | 9.67 |
| 9 | *Cabomba caroliana* | Herb | 58 | 761 | 0.699 | 0.881 |  | 10.75 | 2.05 | 0.70 | 28.47 | 3.12 | ***31.31*** | 2.75 | 20.85 |
| 10 | *Cenchrus pennisetumn* syn *C. purpureum* | Grass | 25 | 929 | 0.695 | 0.926 |  | 0.99 | 15.52 | 10.20 | 4.64 | 10.11 | 22.39 | 5.64 | ***30.50*** |
| 11 | *Cereus hildmannianus* | Succulent | 28 | 46 | 0.911 | 0.984 |  | 16.93 | 17.91 | 4.37 | 3.93 | 18.15 | 14.04 | 2.23 | ***22.45*** |
| 12 | *Ceropegia gigantea* syn *Stapelia gigantea* | Succulent | 11 | 181 | 0.728 | 0.902 |  | 16.96 | 8.42 | 1.50 | 0.23 | 13.47 | ***28.30*** | 21.37 | 9.74 |
| 13 | *Chromolaeana odoratumn* | Shrub | 229 | 4315 | 0.603 | 0.829 |  | 1.09 | 5.47 | 3.92 | 3.40 | 3.55 | ***41.37*** | 1.29 | ***39.92*** |
| 14 | *Coffee arabica* | Shrub | 37 | 1952 | 0.715 | 0.906 |  | 2.18 | 2.41 | 1.00 | 7.53 | 4.21 | ***66.52*** | 9.96 | 6.19 |
| 15 | *Coix lacryma-jobi* | Grass | 5 | 964 | 0.638 | 0.889 |  | 3.04 | 14.28 | 3.45 | 19.35 | 18.17 | ***18.52*** | 6.67 | 16.53 |
| 16 | *Cylindropuntia fulgida* var. *mamillata* | Succulent | 59 | 197 | 0.561 | 0.863 |  | 34.50 | 2.42 | 2.36 | 1.22 | 4.41 | ***39.41*** | 3.83 | 11.86 |
| 17 | *Dalbergia sissoo* | Tree | 22 | 248 | 0.701 | 0.919 |  | 22.11 | 2.71 | 11.60 | 0.49 | 17.02 | 14.83 | ***24.06*** | 7.18 |
| 18 | *Diplachne uninervia* | Grass | 15 | 819 | 0.565 | 0.836 |  | 16.31 | 2.30 | 1.43 | 0.57 | 9.53 | 24.57 | 11.81 | ***33.49*** |
| 19 | *Dyschoriste nagchana* | Herb | 27 | 55 | 0.708 | 0.930 |  | 14.22 | 9.14 | 2.88 | 8.56 | ***25.96*** | 21.39 | 2.91 | 14.94 |
| 20 | *Echinochloa polystachya* | Grass | 36 | 537 | 0.447 | 0.784 |  | 3.62 | 2.35 | 15.87 | 1.51 | 4.76 | ***36.31*** | 3.91 | 31.67 |
| 21 | *Elephantopus mollis* | Shrub | 56 | 1670 | 0.698 | 0.905 |  | 2.08 | 10.01 | 4.04 | 7.62 | 5.17 | ***59.84*** | 6.99 | 4.26 |
| 22 | *Florestina tripteris* | Herb | 16 | 197 | 0.862 | 0.979 |  | 4.89 | 20.77 | 1.53 | 5.87 | 10.54 | 16.91 | 3.29 | ***36.19*** |
| 23 | *Gliricidia sepium* | Tree | 4 | 1167 | 0.698 | 0.879 |  | 0.97 | 1.15 | 1.81 | 1.97 | 3.48 | ***72.31*** | 1.85 | 16.46 |
| 24 | *Gmelina arborea* | Tree | 6 | 915 | 0.541 | 0.909 |  | 2.71 | 2.57 | 3.93 | 9.46 | 19.63 | ***44.11*** | 1.98 | 15.62 |
| 25 | *Heteranthera reniformis* | Herb | 34 | 1020 | 0.509 | 0.808 |  | 16.52 | 3.40 | 11.13 | 5.51 | 17.98 | ***33.09*** | 2.07 | 10.29 |
| 26 | *Hyparrhenia rufa* | Grass | 308 | 2822 | 0.65 | 0.855 |  | 27.37 | 3.64 | 0.46 | 3.79 | 3.59 | ***51.79*** | 3.10 | 6.25 |
| 27 | *Indigofera schimperi* | Herb | 7 | 156 | 0.612 | 0.873 |  | 5.50 | ***30.32*** | 7.02 | 5.13 | 18.60 | 26.51 | 3.53 | 3.38 |
| 28 | *Ipomoea alba* | Vine | 20 | 1343 | 0.606 | 0.867 |  | 1.18 | 6.43 | 1.86 | 17.64 | 7.02 | ***49.16*** | 0.80 | 15.92 |
| 29 | *Jatropha curcas* | Shrub | 14 | 1420 | 0.632 | 0.862 |  | 1.17 | 4.87 | 1.92 | 0.82 | 11.05 | ***66.40*** | 0.85 | 12.93 |
| 30 | *Khaya senegalensis* | Tree | 10 | 1107 | 0.751 | 0.893 |  | ***25.01*** | 6.38 | 3.36 | 14.27 | 13.20 | 10.40 | 2.38 | ***25.00*** |
| 31 | *Leonotis nepetifolia* | Herb | 47 | 1814 | 0.631 | 0.873 |  | 2.26 | 12.78 | 2.29 | 1.42 | 4.35 | ***71.16*** | 2.78 | 2.96 |
| 32 | *Manihot glaziovii* | Shrub | 4 | 244 | 0.574 | 0.861 |  | 1.03 | 9.91 | 1.80 | 1.92 | 6.31 | 24.29 | 21.35 | ***33.40*** |
| 33 | *Mesosphaerum pectinatum* | Herb | 22 | 596 | 0.746 | 0.847 |  | 6.70 | 1.64 | 1.97 | 2.11 | 2.50 | ***37.12*** | 19.48 | 28.48 |
| 34 | *Miconia racemosa* | Tree | 1 | 163 | 0.731 | 0.919 |  | 2.48 | 3.36 | 1.38 | 21.90 | 26.37 | ***32.82*** | 3.58 | 8.11 |
| 35 | *Mimosa pigra* | Shrub | 5 | 2442 | 0.65 | 0.868 |  | 6.63 | 5.51 | 2.40 | 2.37 | 3.58 | ***53.27*** | 3.21 | **23.04** |
| 36 | *Murraya koenigii* | Tree | 26 | 555 | 0.607 | 0.868 |  | ***37.77*** | 3.03 | 17.28 | 1.24 | 15.57 | 15.43 | 4.33 | 5.36 |
| 37 | *Neptunia plena* | Herb | 9 | 533 | 0.634 | 0.864 |  | 20.07 | 22.56 | 0.81 | 2.41 | 9.73 | 15.24 | 4.73 | ***24.45*** |
| 38 | *Opuntia dejecta* | Succulent | 1 | 74 | 0.793 | 0.938 |  | 0.32 | 6.98 | 2.07 | 3.34 | 22.59 | ***58.33*** | 0.17 | 6.22 |
| 39 | *Opuntia elata* | Succulent | 15 | 180 | 0.644 | 0.880 |  | 3.49 | 6.23 | 5.92 | 9.37 | 17.42 | ***32.43*** | 7.32 | 17.81 |
| 40 | *Opuntia sulphurea* | Succulent | 5 | 235 | 0.671 | 0.898 |  | 26.73 | 0.95 | 1.49 | 8.64 | 6.59 | 12.95 | 2.96 | ***39.71*** |
| 41 | *Paspalum mandiocanum* | Grass | 44 | 402 | 0.819 | 0.947 |  | 6.10 | 4.15 | 2.70 | 3.31 | 12.74 | ***39.11*** | **13.51** | 18.38 |
| 42 | *Pithecellobium dulce* | Tree | 19 | 1515 | 0.653 | 0.869 |  | 3.61 | 7.61 | 0.67 | 6.26 | 11.81 | ***59.40*** | 1.05 | 9.59 |
| 43 | *Praxelis clematidea* | Herb | 327 | 1080 | 0.61 | 0.853 |  | 2.74 | 9.26 | 2.34 | 6.54 | 5.01 | ***39.65*** | 12.66 | 21.79 |
| 44 | *Rhodomyrtus tomentosa* | Shrub | 9 | 427 | 0.713 | 0.910 |  | 4.01 | 5.46 | 2.08 | 18.14 | 19.73 | ***26.18*** | 2.31 | 22.09 |
| 45 | *Rotala rotundifolia* | Herb | 15 | 335 | 0.724 | 0.906 |  | 4.16 | 17.67 | 2.92 | 15.64 | 6.88 | ***23.65*** | 10.43 | 18.64 |
| 46 | *Schizachyrium microstachyum* | Grass | 22 | 700 | 0.565 | 0.836 |  | 2.61 | 26.46 | 2.23 | 1.35 | 2.08 | ***48.64*** | 4.42 | 12.21 |
| 47 | *Setaria parviflora* | Grass | 4 | 752 | 0.742 | 0.942 |  | 1.63 | 8.89 | 5.90 | 4.82 | 8.66 | 6.71 | 1.55 | ***61.86*** |
| 48 | *Sieruela rutidosperma* | Herb | 2 | 1163 | 0.794 | 0.944 |  | 1.87 | 25.93 | 8.57 | 0.31 | 1.39 | 9.25 | 3.60 | ***49.07*** |
| 49 | *Spathodea campanulata* | Tree | 89 | 1886 | 0.654 | 0.881 |  | 0.29 | 4.80 | 0.47 | 3.95 | 1.81 | ***74.62*** | 6.25 | 7.81 |
| 50 | *Stigmaphyllon ciliatum* | Vine | 6 | 136 | 0.8 | 0.939 |  | 0.82 | 3.07 | 13.75 | 12.04 | 19.21 | ***29.44*** | 7.58 | 14.09 |
| 51 | *Syzygium jambos* | Tree | 12 | 1292 | 0.704 | 0.910 |  | 1.49 | 7.83 | 3.32 | 8.07 | 8.19 | ***52.15*** | 9.02 | 9.92 |
| 52 | *Thunbergia fragrans* | Vine | 62 | 588 | 0.691 | 0.914 |  | 1.79 | 12.94 | 5.04 | 6.18 | 8.56 | ***48.60*** | 5.40 | 11.49 |
| 53 | *Toxicodendron radicans* | Vine | 2 | 10639 | 0.793 | 0.817 |  | 3.88 | 0.08 | 0.04 | ***30.70*** | 8.89 | 18.18 | 29.72 | 8.50 |
| 54 | *Ziziphus mucronata* | Tree | 0 | 1315 | 0.585 | 0.834 |  | 1.51 | ***52.30*** | 2.25 | 0.36 | 4.07 | 30.07 | 2.35 | 7.09 |
|  |  |  |  |  |  |  |  |  |  |  |  |  |  |  |  |
|  | Average |  | 35.65 | 1147 | 0.669 | 0.883 |  | 8.08 | 8.76 | 4.07 | 7.27 | 10.32 | ***35.14*** | 7.41 | 18.96 |
|  |  |  |  |  |  |  |  |  |  |  |  |  |  |  |  |

| **Appendix S4:** Mean climatic variables by plant growth form. Bioclimatic abbreviations are explained in the texts of the manuscript. | | | | | |  |
| --- | --- | --- | --- | --- | --- | --- |
|  |  |  |  |  |  |  |
|  |  |  |  | 95% Confidence Interval | |  |
| Climatic variable | Life form | Mean | Std. Error | Lower Bound | Upper Bound |  |
| bio1_Ann Mean_temp | Grass | 21.36 | 0.42 | 20.52 | 22.19 |  |
|  | Herb | 21.08 | 0.35 | 20.38 | 21.77 |  |
|  | Shrub | 23.42 | 0.42 | 22.59 | 24.26 |  |
|  | Succulent | 19.56 | 0.52 | 18.54 | 20.58 |  |
|  | Tree | 24.15 | 0.40 | 23.36 | 24.95 |  |
|  | Vine | 20.46 | 0.48 | 19.51 | 21.41 |  |
|  |  |  |  |  |  |  |
| bio12_Ann_Precipitn | Grass | 1415.76 | 61.33 | 1294.87 | 1536.66 |  |
|  | Herb | 1304.84 | 51.03 | 1204.25 | 1405.43 |  |
|  | Shrub | 1643.55 | 61.33 | 1522.65 | 1764.44 |  |
|  | Succulent | 686.85 | 75.11 | 538.79 | 834.91 |  |
|  | Tree | 1356.47 | 58.18 | 1241.78 | 1471.16 |  |
|  | Vine | 1352.44 | 69.54 | 1215.36 | 1489.52 |  |
|  |  |  |  |  |  |  |
| bio13_Precip_Wettest_Mth | Grass | 236.23 | 11.10 | 214.34 | 258.12 |  |
|  | Herb | 222.57 | 9.24 | 204.35 | 240.78 |  |
|  | Shrub | 277.34 | 11.10 | 255.45 | 299.23 |  |
|  | Succulent | 117.28 | 13.60 | 90.47 | 144.09 |  |
|  | Tree | 271.94 | 10.53 | 251.17 | 292.70 |  |
|  | Vine | 212.91 | 12.59 | 188.09 | 237.73 |  |
|  |  |  |  |  |  |  |
| bio14_Precip_Driest_Mth | Grass | 35.45 | 2.45 | 30.63 | 40.28 |  |
|  | Herb | 32.77 | 2.04 | 28.75 | 36.78 |  |
|  | Shrub | 41.13 | 2.45 | 36.30 | 45.95 |  |
|  | Succulent | 18.13 | 3.00 | 12.22 | 24.04 |  |
|  | Tree | 22.39 | 2.32 | 17.81 | 26.97 |  |
|  | Vine | 43.82 | 2.78 | 38.35 | 49.29 |  |
|  |  |  |  |  |  |  |
| bio15_Precipt_Seasonality | Grass | 62.71 | 2.39 | 58.01 | 67.42 |  |
|  | Herb | 64.19 | 1.99 | 60.27 | 68.10 |  |
|  | Shrub | 64.07 | 2.39 | 59.36 | 68.77 |  |
|  | Succulent | 61.14 | 2.92 | 55.38 | 66.91 |  |
|  | Tree | 84.47 | 2.26 | 80.00 | 88.93 |  |
|  | Vine | 52.35 | 2.71 | 47.02 | 57.69 |  |
|  |  |  |  |  |  |  |
| bio4_Temp_seasonality | Grass | 320.90 | 22.82 | 275.91 | 365.89 |  |
|  | Herb | 343.52 | 18.99 | 306.08 | 380.95 |  |
|  | Shrub | 201.17 | 22.82 | 156.18 | 246.16 |  |
|  | Succulent | 440.51 | 27.95 | 385.41 | 495.62 |  |
|  | Tree | 246.09 | 21.65 | 203.41 | 288.78 |  |
|  | Vine | 339.75 | 25.88 | 288.73 | 390.77 |  |
|  |  |  |  |  |  |  |
| bio5_Max_Temp_Warmest_Mth | Grass | 31.08 | 0.31 | 30.48 | 31.69 |  |
|  | Herb | 30.84 | 0.25 | 30.34 | 31.34 |  |
|  | Shrub | 31.01 | 0.31 | 30.41 | 31.61 |  |
|  | Succulent | 31.77 | 0.37 | 31.03 | 32.50 |  |
|  | Tree | 33.03 | 0.29 | 32.46 | 33.60 |  |
|  | Vine | 30.40 | 0.35 | 29.72 | 31.08 |  |
|  |  |  |  |  |  |  |
| bio6_Min_Temp_Colodest_Mth | Grass | 11.49 | 0.71 | 10.09 | 12.90 |  |
|  | Herb | 10.36 | 0.59 | 9.20 | 11.53 |  |
|  | Shrub | 15.34 | 0.71 | 13.94 | 16.74 |  |
|  | Succulent | 6.67 | 0.87 | 4.95 | 8.38 |  |
|  | Tree | 14.54 | 0.67 | 13.21 | 15.87 |  |
|  | Vine | 10.11 | 0.81 | 8.52 | 11.70 |  |
|  |  |  |  |  |  |  |
|  |  |  |  |  |  |  |
